# ZC3H15 regulates the ubiquitination of PTEN via recruitment of TRIM56 and promotes malignant progression of non-small cell lung cancer

**DOI:** 10.1038/s41419-025-08138-2

**Published:** 2026-01-09

**Authors:** Peihong Wu, Peifeng Yao, Mingfang Zhao, Ming Cheng

**Affiliations:** 1 Department of Medical Oncology, The First Hospital of China Medical University, No. 155 NanjingBei Street, Heping District, Shenyang, 110000 Liaoning Province PR China; 2 Department of Hand Surgery, Central Hospital affiliated to Shenyang Medical College, 110000 Shenyang, Liaoning Province PR China; 3 Department of Pathology, Shengjing Hospital of China Medical University, No. 36 Sanhao Street, Heping District, 110000 Shenyang, Liaoning Province PR China

**Keywords:** Non-small-cell lung cancer, Oncogenes

## Abstract

Lung cancer is one of the most common cancers worldwide and the leading cause of cancer-related deaths. Non-small cell lung cancer (NSCLC) accounts for 85% of lung cancer cases and has a 5-year survival rate of ~19%. Since more than half of NSCLC patients present with metastatic disease at the time of diagnosis, early diagnosis is crucial for providing patients with the most effective treatment strategy. This study integrated transcriptome data between cancer and adjacent tissues from GEO and TCGA databases through bioinformatics analysis, and screened zinc finger CCCH-type containing 15 (ZC3H15) as a key differentially expressed gene in NSCLC. ZC3H15 expression levels were found to be significantly higher in NSCLC tissue than normal tissue and correlated with tumor size, TNM stage, lymph node metastasis and poor prognosis of patients. Overexpression of ZC3H15 promoted the proliferation, migration and invasion of NSCLC cells through activation of the AKT-mTOR signaling pathway. To elucidate the underlying molecular mechanism, we determined that ZC3H15 could bind to PTEN through its DFRP structural domain and recruited the E3 ligase TRIM56 to promote PTEN ubiquitination. In addition, overexpression of ZC3H15 increased the resistance of NSCLC cells to cisplatin. Therefore, ZC3H15 promotes the malignant phenotype of NSCLC through recruitment of TRIM56 to ubiquitinate PTEN, decreasing its expression and driving increased AKT-mTOR signaling pathway and cisplatin resistance. These findings provide a scientific basis for the development of targeted therapies against ZC3H15, which may lead to new therapeutic strategies for NSCLC patients.

## Introduction

Lung cancer is one of the most common cancers and the leading cause of cancer-related deaths worldwide [1] with an estimated 2 million new cases and 1.76 million deaths annually. There are two main histologic types of lung cancer, with non-small cell lung cancer (NSCLC) patients accounting for 85% of all lung cancer patients and small cell lung cancer patients accounting for 15%. The 5-year survival rate for NSCLC patients is ~19% [2]. More than 50% of lung cancer patients have a low survival rate due to the fact that at the time of diagnosis many NSCLC patients present with metastatic disease. Thus, early diagnosis of the disease is critical to ensure that each patient receives the most effective treatment [3, 4].

Zinc finger CCCH-type containing 15 (ZC3H15), a member of the CCCH zinc finger family of proteins, is a highly conserved eukaryotic protein involved in various cellular processes associated with tumorigenesis [5]. The ZC3H15 gene, also known as LEREPO4 or DFRP1, is located on chromosome 2q32.1, and regulates developmentally-regulated GTP binding protein 1 (DRG1), which plays an important role in cellular signaling [6, 7]. In addition to the CCCH zinc finger domain, ZC3H15 also contains a conserved DRG family regulatory protein (DFRP) domain, which is required for the association of DFRP1/DFRP2 with DRG. DFRP proteins have been shown to stabilize the expression of their corresponding DRG proteins, as well as stabilize GTPases [8]. DFRP1 reportedly stimulates the GTPase activity of DRG1 by increasing its affinity for potassium ions [9]. In addition, the binding of DFRP1 to DRG1 prevents DRG1 degradation, most likely via ubiquitination and proteasomal degradation [10].

Recently, ZC3H15 has been shown to be highly expressed in glioblastoma (GBM) and is associated with poor prognosis. ZC3H15 promotes proliferation, migration, invasion and tumorigenesis of GBM cells by inhibiting epidermal growth factor receptor (EGFR) ubiquitination and degradation [11]. In addition, ZC3H15 has been shown to inhibit Casitas B-lineage lymphoma (CBL) transcription and promote EGFR protein stability. High ZC3H15 expression has also been reported in gastric cancer and is associated with poor prognosis. ZC3H15 has been shown to regulate c-Myc protein stability by inhibiting the transcription of FBXW7, which is responsible for c-Myc degradation. The subsequent increase in c-Myc expression levels promotes the proliferation, migration and invasion ability of gastric cancer cells [12].

To date, a role for ZC3H15 in NSCLC has not been reported. Thus, the aim of the current study was to investigate the expression of ZC3H15 protein in NSCLC and its correlation with clinical features, examine the effects of ZC3H15 on the biological behavior of NSCLC cells, and identify potential molecular mechanisms of action in order to determine the potential of ZC3H15 as a new target for the treatment of NSCLC.

## Results

### ZC3H15 plays a key role in the progression and prognosis of NSCLC

**Fig. 1:**
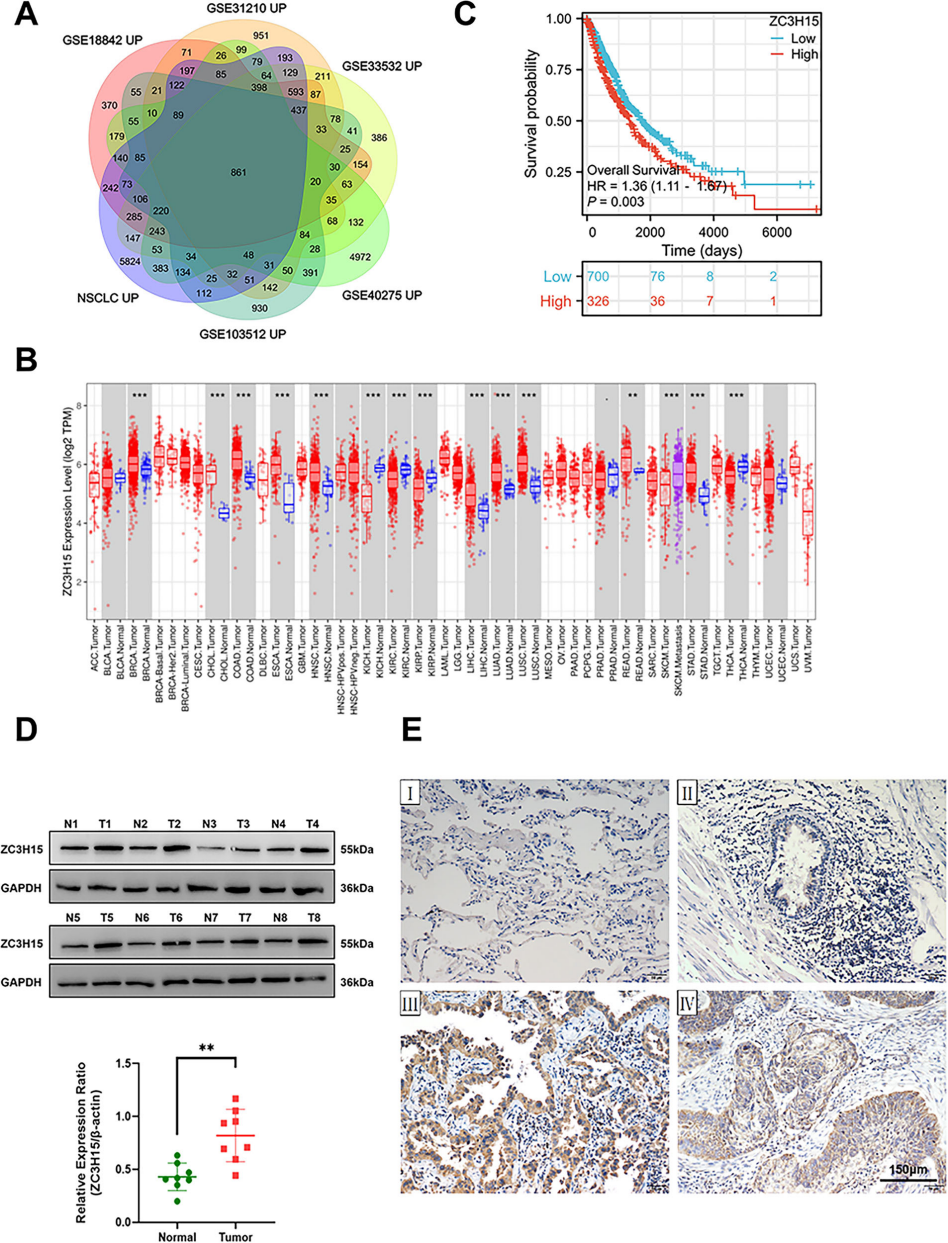
ZC3H15 plays a key role in the progression and prognosis of NSCLC. **A** Venn diagrams showing overlaps between five GEO datasets and the data from TCGA database. **B** ZC3H15 mRNA expression was investigated in the TCGA database and compared among 33 kinds of tumors. **C** The analysis of overall survival in all NSCLC patients. **D** Western blotting analyses of ZC3H15 levels in eight lung cancer tissues and matched normal tissues. Student’s *t* test. Mean ± SD, *n* = 3. **P < 0.01. **E** ZC3H15 levels in normal alveolar(Ⅰ) and bronchial epithelial cell(Ⅱ), adenocarcinoma(Ⅲ),and squamous cell carcinoma(Ⅳ) using immunohistochemistry. Magnification: ×200.

Genes differentially expressed in NSCLC were identified by mining five datasets from the GEO database and lung adenocarcinoma (LUAD) & lung squamous cell carcinoma (LUSC) samples from the Cancer Genome Atlas (TCGA) database using RNA-sequencing (RNA-seq) analysis. We found 861 upregulated genes [*P* < 0.05, |logFC|>0.1] in NSCLC samples (Fig. 1A). UniCOX analysis was then performed on the 861 differentially upregulated genes with a screening criteria of *P* < 0.001, and a total of 43 independent prognostic factors were obtained (Supplementary Fig. 1A). Next, survival analysis was performed on 42 genes from previous microarray data (GSE31210) and TCGA lung cancer samples. We found that ZC3H15 was highly expressed and correlated with poor prognosis in both sets of data (Supplementary Fig. 1B, C). In contrast, other top candidates (e.g., E2F7, KIF18A, and CDC25A) exhibited weaker or inconsistent clinical associations (Supplementary Fig. 1D–F). Therefore, we selected ZC3H15 for further analysis.

Analysis of data from the online bioinformatics analysis site TIMER (https://cistrome.shinyapps.io/timer) revealed that ZC3H15 mRNA is highly expressed in various tumor tissues, including LUAD and LUSC (Fig. 1B). Our analysis of 1149 TCGA and matched samples showed that ZC3H15 mRNA levels were significantly increased in lung cancer tissues [*P* < 0.001] (Supplementary Fig. 1G, H). Data analysis of the online bioinformatics analysis site Xiantao Academic (https://www.xiantaozi.com/) revealed that ZC3H15 was associated with tumor size and lymph node metastasis (Supplementary Fig. 1I, J). Furthermore, high expression of ZC3H15 was found to be a predictor of poor prognosis in lung cancer patients [*P* = 0.003] (Fig. 1C).

Next, we used western blot analysis to examine ZC3H15 expression levels in eight lung cancer tissues and normal tissues (Fig. 1D). ZC3H15 protein expression levels were found to be higher in lung cancer tissue than the corresponding adjacent tissue [*P* < 0.01] and normal tissue (Fig. 1E). In addition, clinical case-related factor analysis showed that high ZC3H15 expression levels correlated with tumor size [*P* = 0.006], TNM stage [*P* = 0.012] and lymph node metastasis [*P* = 0.003] (Table 1). Overall, our results suggest that ZC3H15 is highly expressed in NSCLC and may therefore serve as a potential therapeutic target for NSCLC.

**Table 1:** Association of ZC3H15 expression with clinical and pathological characteristics of patients.

Factors	Number of samples	ZC3H15Negative	ZC3H15Positive	*P* value
Gender				0.963
Male	92	54 (32.9%)	38 (23.2%)	
Female	72	42 (25.6%)	30 (18.3%)	
Age(years)				0.196
≤60	77	41 (25.0%)	36 (22.0%)	
å 60	87	55 (33.5%)	32 (19.5%)	
Tumor size(cm)				**0.006**
≤3	81	56 (34.1%)	25 (15.2%)	
å 3	83	40 (24.4%)	43 (26.2%)	
Differentiation				0.060
Well	89	58 (35.4%)	31 (18.9%)	
Poor-moderate	75	38 (23.2%)	37 (22.6%)	
Histological type				0.469
Adenocarcinoma	91	51 (31.1%)	40 (24.4%)	
Squamous carcinoma	73	45 (27.4%)	28 (17.1%)	
TNM stage				**0.012**
Ⅰ	96	64 (39.0%)	32 (19.5%)	
Ⅱ-Ⅲ	68	32 (19.5%)	36 (22.0%)	
Lymph node status				**0.003**
Negative	85	59 (36.0%)	26 (15.9%)	
Positive	79	37 (22.6%)	42 (25.6%)	

TNM Eighth Edition of tumor-node-metastasis classification.

The bold and underlined values were considered statistically significant.

### Overexpression of ZC3H15 promotes the malignant progression of NSCLC

**Fig. 2:**
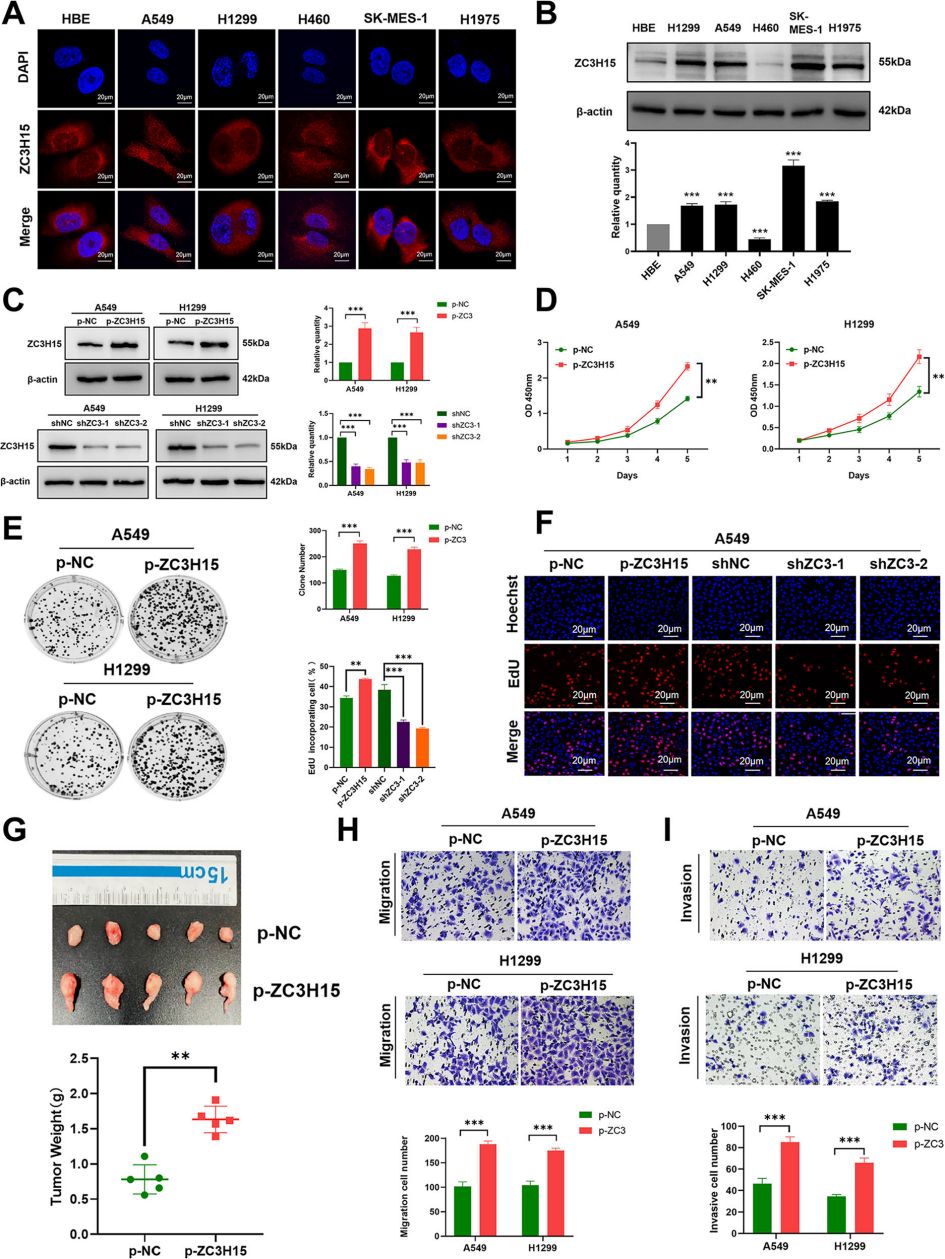
Overexpression of ZC3H15 promotes the malignant progression of NSCLC. **A** Immunofluorescence was performed to detect ZC3H15 localization in cell lines. **B** Western blotting analyses of ZC3H15 expression in lung epithelial cell line, HBE and six NSCLC cell lines. Student’s *t* test. Mean ± SD, *n* = 3. ***P < 0.001. **C** Western blotting analyzing the expression of ZC3H15 in the indicated cells. Multiple *t* tests. Mean ± SD, *n* = 3. ***P < 0.001. **D** Cell viability was analyzed by CCK8. Multiple *t* tests. Mean ± SD, *n* = 3. **P < 0.01. **E** Cell growth was determined by colony formation. Mean ± SD, *n* = 3. **P < 0.01, ***P < 0.001. **F** DNA replication of A549 cells was determined by EDU staining. Scale bar: 20 μm. Mean ± SD, *n* = 3. ***P < 0.001. **G** The xenograft tumor model in nude mice established by A549 cells with ZC3H15 overexpression. Student’s *t* test. Mean ± SD, *n* = 3. ***P < 0.001. Cell migration (**H**) and invasion (**I**) was evaluated by the Transwell migration assay; cells that migrated to the lower chamber were stained with hematoxylin and counted. Mean ± SD, *n* = 3. ***P < 0.001.

Subsequently, we sought to determine the biological function of ZC3H15 in NSCLC using lung cancer cell lines. Immunofluorescence experiments revealed that ZC3H15 was mainly localized in the cytoplasm of lung cancer cells (Fig. 2A). Further quantitative analysis showed that ZC3H15 expression levels were higher in NSCLC cell lines including A549, NCI-H1299, NCI-H1975, and SK-MES-1 cells than the normal bronchial epithelial cell line HBE (Fig. 2B). NCI-H460 cells did not display high ZC3H15 expression levels (Fig. 2B). Since intermediate ZC3H15 expression levels were observed in A549 and NCI-H1299 cells, these cell lines were selected for subsequent experiments. We constructed cell lines stably overexpressing and silencing ZC3H15 using lentiviruses. The lentiviral transduction efficiencies of these stable cell lines are shown in Fig. 2C. Based on the potential correlation between ZC3H15 and tumor growth and lymph node metastasis, as well as its association with the cell cycle identified by gene set enrichment analysis (GSEA), we hypothesized that ZC3H15 may play an important role in cell proliferation, migration and invasion (Supplementary Fig. 2A).

Using CCK-8, colony formation and 5-ethynyl-2’-deoxyuridine (EdU) assays, we demonstrated that overexpression of ZC3H15 promoted the proliferative capacity of cells in vitro (Fig.2D–F and Supplementary Fig. 2B) and in vivo (Fig. 2G and Supplementary Fig. 2C). In addition, overexpression of ZC3H15 increased the migration and invasion abilities of lung cancer cells (Fig. 2H, I and Supplementary Fig. 2D). Consistent with these findings, overexpression of ZC3H15 promoted the expression of proliferation, migration, and invasion-related proteins including CDK4, CDK6, Cyclin D1, E-cadherin, N-cadherin, and MMP9 (Supplementary Fig. 2E). In contrast, knockdown of ZC3H15 led to decreased proliferation, migration, and invasion, as well as a corresponding reduction in the expression levels of related proteins (Fig. 2F and Supplementary Figs. 2B and 3). Together, these results confirm that ZC3H15 plays an important role in promoting the proliferation, migration and invasion of lung cancer cells.

### ZC3H15 promotes tumorigenesis in NSCLC cells through the AKT-mTOR pathway

**Fig. 3:**
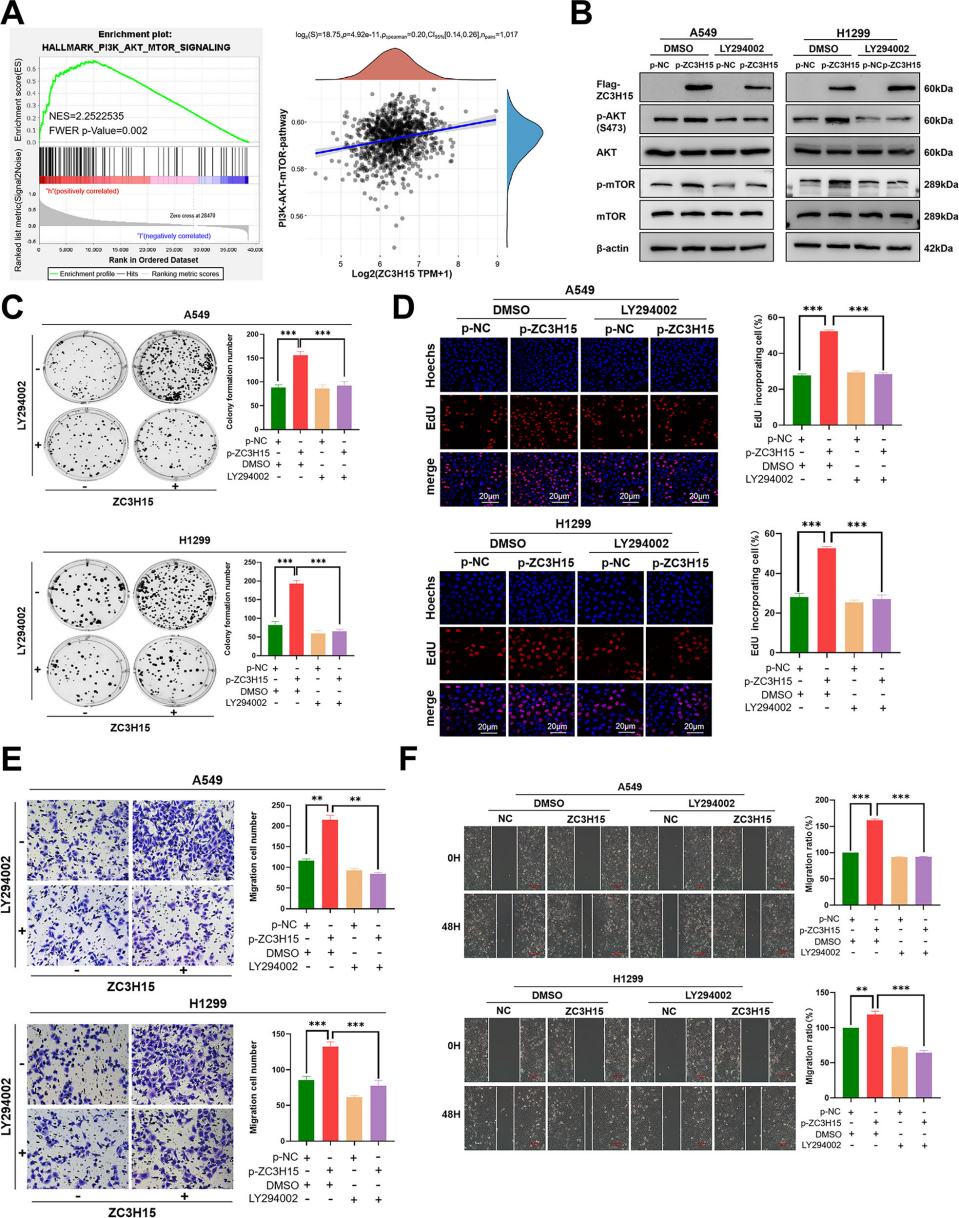
ZC3H15 promotes tumorigenesis in NSCLC cells through the AKT-mTOR pathway. **A** GSEA in the TCGA database of ZC3H15-related enrichment plots were performed. **B** Western blotting analyzing the expression of proteins involved in the AKT-mTOR pathway in A549 and H1299 cells treated with DMSO or the AKT pathway inhibitor LY294002. **C** Cell growth was determined by colony formation. Unpaired *t* test. Mean ± SD, *n* = 3. ***P < 0.001. **D** DNA replication of A549 and H1299 cells treated with DMSO or the AKT pathway inhibitor LY294002 was determined by EDU staining. Scale bar: 200 μm. Mean ± SD, *n* = 3. ***P < 0.001. **E** Cell migration was evaluated by the Transwell migration assay. Mean ± SD, *n* = 3. **P < 0.01, ***P < 0.001. **F** Wound-healing assay showing the LY294002 effect on the ZC3H15 overexpressed A549 and H1299 cell migration. Mean ± SD, *n* = 3. **P < 0.01, ***P < 0.001.

GSEA analysis of TCGA database suggested that ZC3H15 was associated with the AKT-mTOR pathway (Fig. 3A). Thus, we asked whether ZC3H15 exerted its pro-oncogenic effects by activating the AKT-mTOR pathway in NSCLC. The AKT-mTOR pathway, a signaling pathway that is stimulated and activated by cytokines, plays an important role in various biological processes such as proliferation and migration. Western blot analysis revealed that p-AKT and p-mTOR protein expression levels were upregulated following overexpression of ZC3H15 (Supplementary Fig. 4A), while ZC3H15 gene silencing had the opposite effect (Supplementary Fig. 4B). Next, we treated cells with LY294002, an AKT pathway inhibitor, to determine whether ZC3H15 regulates the biological functions of NSCLC cells through the AKT-mTOR signaling pathway. We found that activation of the AKT-mTOR signaling pathway following ZC3H15 overexpression was inhibited in the presence of LY294002 (Fig. 3B). Colony formation, EdU, scratch, and Transwell migration and invasion assays also demonstrated that the promotion of cell proliferation, migration and invasion following ZC3H15 overexpression was inhibited by LY294002 treatment (Fig. 3C–F and Supplementary Fig. 4C, D). Meanwhile, after adding LY294002 to ZC3H15 knockdown cells, the inhibitory effects of ZC3H15 depletion on cell proliferation, migration, and invasion were not further enhanced (Supplementary Figs. 4E, F and 5). Together, our findings suggest that ZC3H15 promotes the proliferation, migration and invasion of lung cancer cells through activation of the AKT-mTOR signaling pathway.

### ZC3H15 is a novel PTEN-interacting protein

**Fig. 4:**
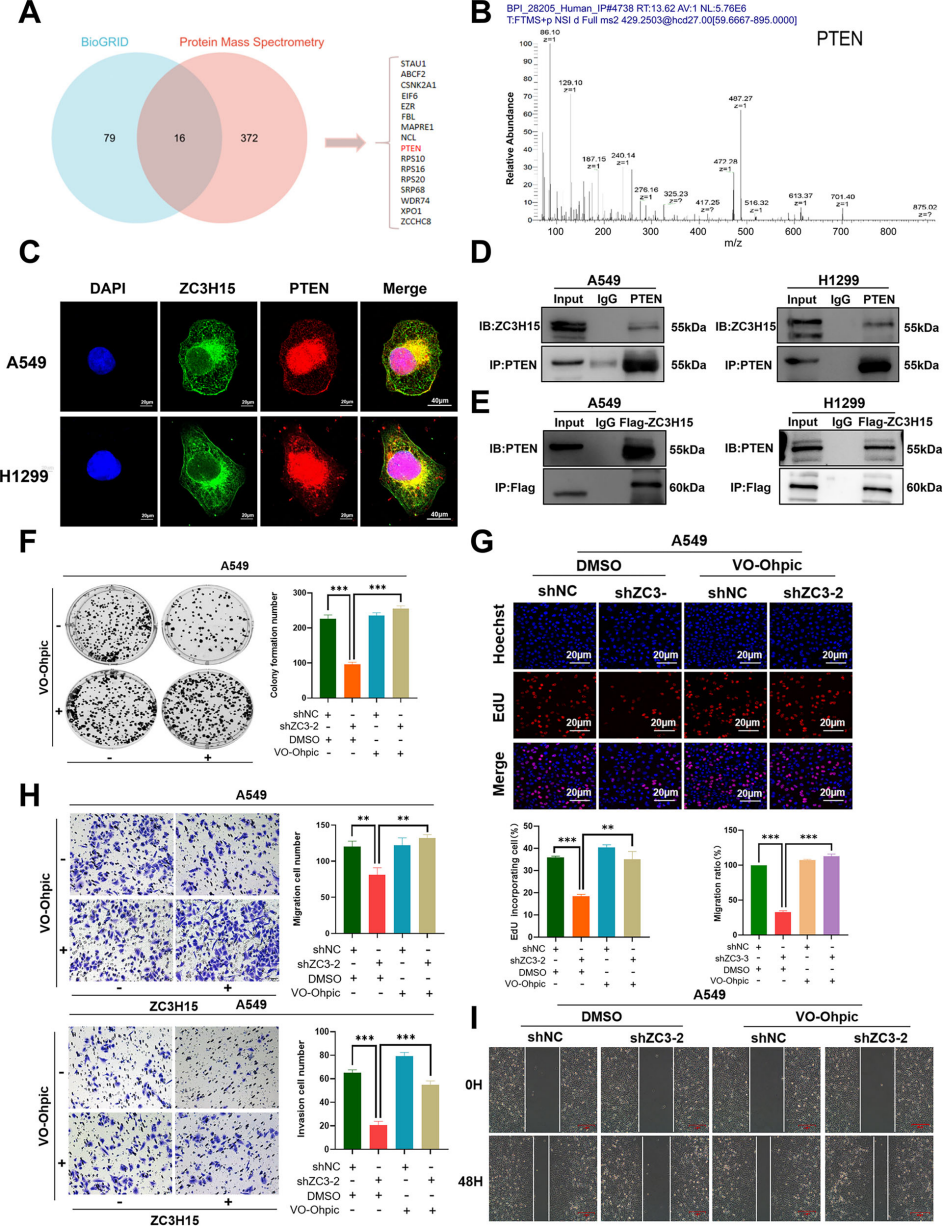
ZC3H15 is a novel PTEN-interacting protein. **A** The Venn diagram shows the overlaps between protein mass spectrometry analysis results and the data from BioGRID website. **B** The raw MS/MS spectrum of PTEN. **C** Immunofluorescence assay results indicate that ZC3H15 and PTEN colocalize within the cytoplasm of A549 and H1299 cells. **D**, **E** Interactions between PHF23 and ACTN4 in A549 and H1299 cells measured by co-immunoprecipitation. **F** Cell growth of A549 treated with DMSO or VO-Ohpic was determined by colony formation. Unpaired *t* test. Mean ± SD, *n* = 3. ***P < 0.001. **G** DNA replication of A549 treated with DMSO or VO-Ohpic was determined by EDU staining. Scale bar: 20 μm. Mean ± SD, *n* = 3. **P < 0.01, ***P < 0.001. **H** Cell migration and invasion of A549 was evaluated by the Transwell migration assay. Mean ± SD, *n* = 3. **P < 0.01, ***P < 0.001. **I** The migration of A549 treated with DMSO or VO-Ohpic was evaluated by the wound-healing assay. Mean ± SD, *n* = 3. ***P < 0.001.

To further understand how ZC3H15 interacts with the AKT-mTOR signaling pathway, we performed protein profiling and screened a total of 388 binding proteins. Sixteen binding proteins were obtained by taking the intersection with 95 proteins queried in the BioGRID online database (https://thebiogrid.org) that may interact with ZC3H15, including STAU1, ABCF2, CSNK2A1, EIF6, EZR, FBL, MAPRE1, NCL, PTEN, RPS10, RPS16, RPS20, SRP68, WDR74, XPO1, and ZCCHC8 (Fig. 4A). Since PTEN negatively regulates the AKT signaling pathway and influences tumor prognosis, we selected PTEN as a candidate protein that might interact with ZC3H15 (Fig. 4B).

Immunoprecipitation (IP) assays revealed that ZC3H15 interacts with PTEN, while immunofluorescence (IF) staining showed that ZC3H115 and PTEN co-localize in the cytoplasm (Fig. 4C–E). Addition of VO-Ohpic trihydrate (VO-Ohpic), a potent PTEN inhibitor, to ZC3H15-silenced cells effectively activated the AKT-mTOR signaling pathway. At the same time, the inhibitory effects of ZC3H15 knockdown on cell proliferation, migration and invasion were reversed (Fig. 4F–I and Supplementary Fig. 6). Taken together, our results suggest that ZC3H15 interacts with PTEN, activates the AKT-mTOR signaling pathway and promotes the malignant phenotype of NSCLC cells.

### ZC3H15 binds to PTEN through its DFRP structural domain

**Fig. 5:**
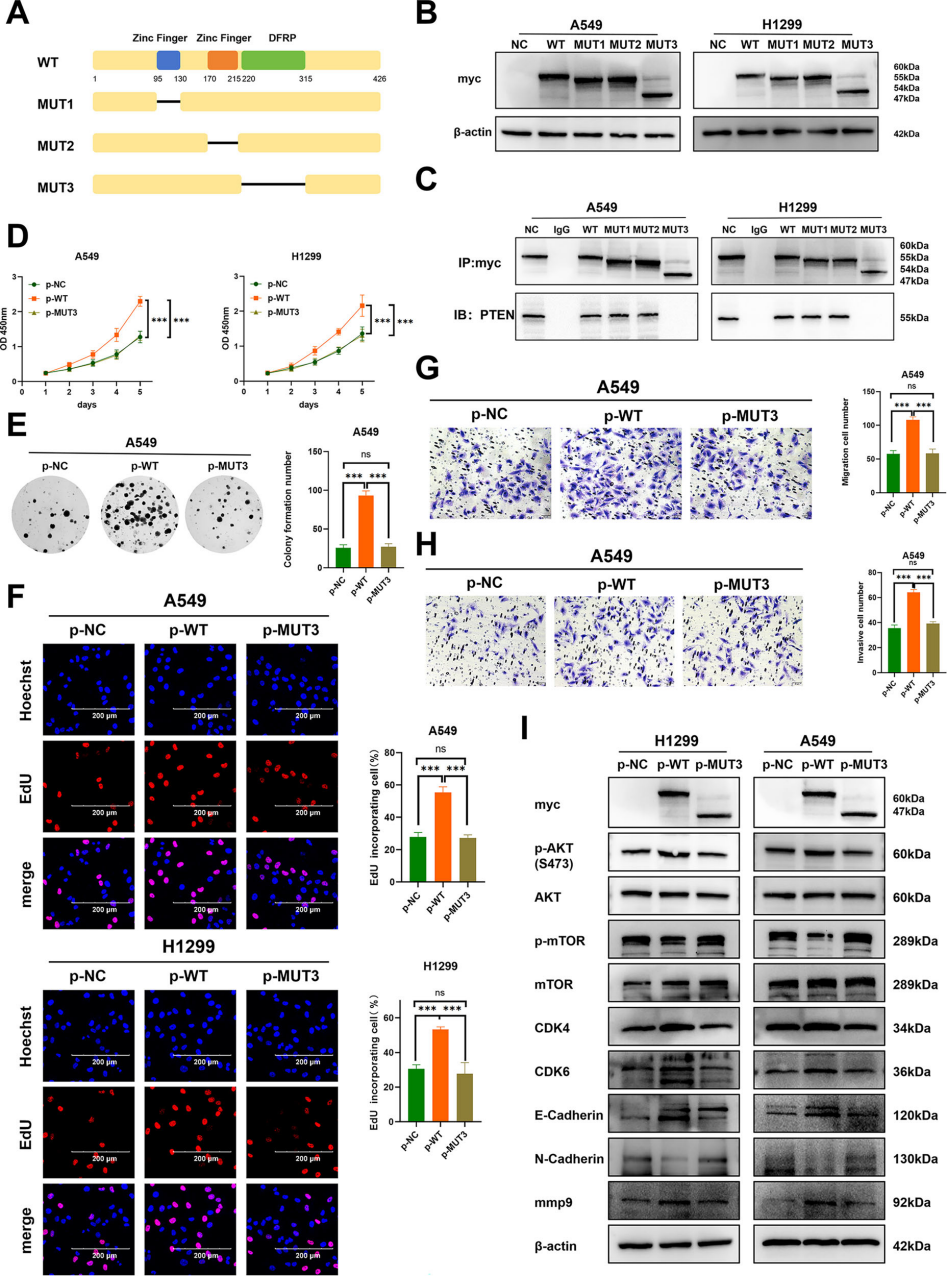
ZC3H15 binds to PTEN through its DFRP structural domain. **A** Schematic diagram of ZC3H15 splicing mutants. **B** The expression of myc-tag after transfected with ZC3H15 splicing mutant cDNA. **C** Interactions between ZC3H15 splicing mutants and PTEN in A549 and H1299 cells measured by co-immunoprecipitation. **D** Cell viability of A549 and H1299 transfected with ZC3H15 cDNA or ZC3H15 MUT3 cDNA was analyzed by CCK8. Unpaired *t* test. Mean ± SD, *n* = 3. ***P < 0.001. **E** Cell growth of A549 transfected with ZC3H15 cDNA or ZC3H15 MUT3 cDNA was determined by colony formation. Mean ± SD, *n* = 3. ***P < 0.001. **F** DNA replication of A549 transfected with ZC3H15 cDNA or ZC3H15 MUT3 cDNA was determined by EDU staining. Scale bar: 20 μm. Mean ± SD, *n* = 3. ***P < 0.001. The migration (**G**) and invasion (**H**) of A549 transfected with ZC3H15 cDNA or ZC3H15 MUT3 cDNA was evaluated by the Transwell migration assay. Mean ± SD, *n* = 3. ***P < 0.001. **I** Western blotting analyzing the expression of proteins involved in the AKT-mTOR pathway and proliferation- and migration-related proteins in A549 and H1299 cells transfected with ZC3H15 cDNA or ZC3H15 MUT3 cDNA.

Previous studies have shown that ZC3H15 contains two CCCH zinc finger structural domains and a conserved DFRP structural domain. To further explore the structural basis of ZC3H15 binding to PTEN, we constructed a series of mutants with deletion of the CCCH and DFRP domains (Fig. 5A). The transfection efficiency is shown in Fig. 5B. IP experiments in H1299 and A549 cells revealed that deletion of the DFRP domain in ZC3H15 eliminated the interaction between ZC3H15 and PTEN (Fig. 5C). In addition, deletion of the DFRP domain significantly inhibited activation of the AKT-mTOR signaling pathway and promoted proliferation, migration and invasion in lung cancer cells (Fig. 5D–I and Supplementary Fig. 7). These results suggest that ZC3H15 binds to PTEN in a DFRP-dependent manner.

### ZC3H15 recruits TRIM56 to promote PTEN ubiquitination

**Fig. 6:**
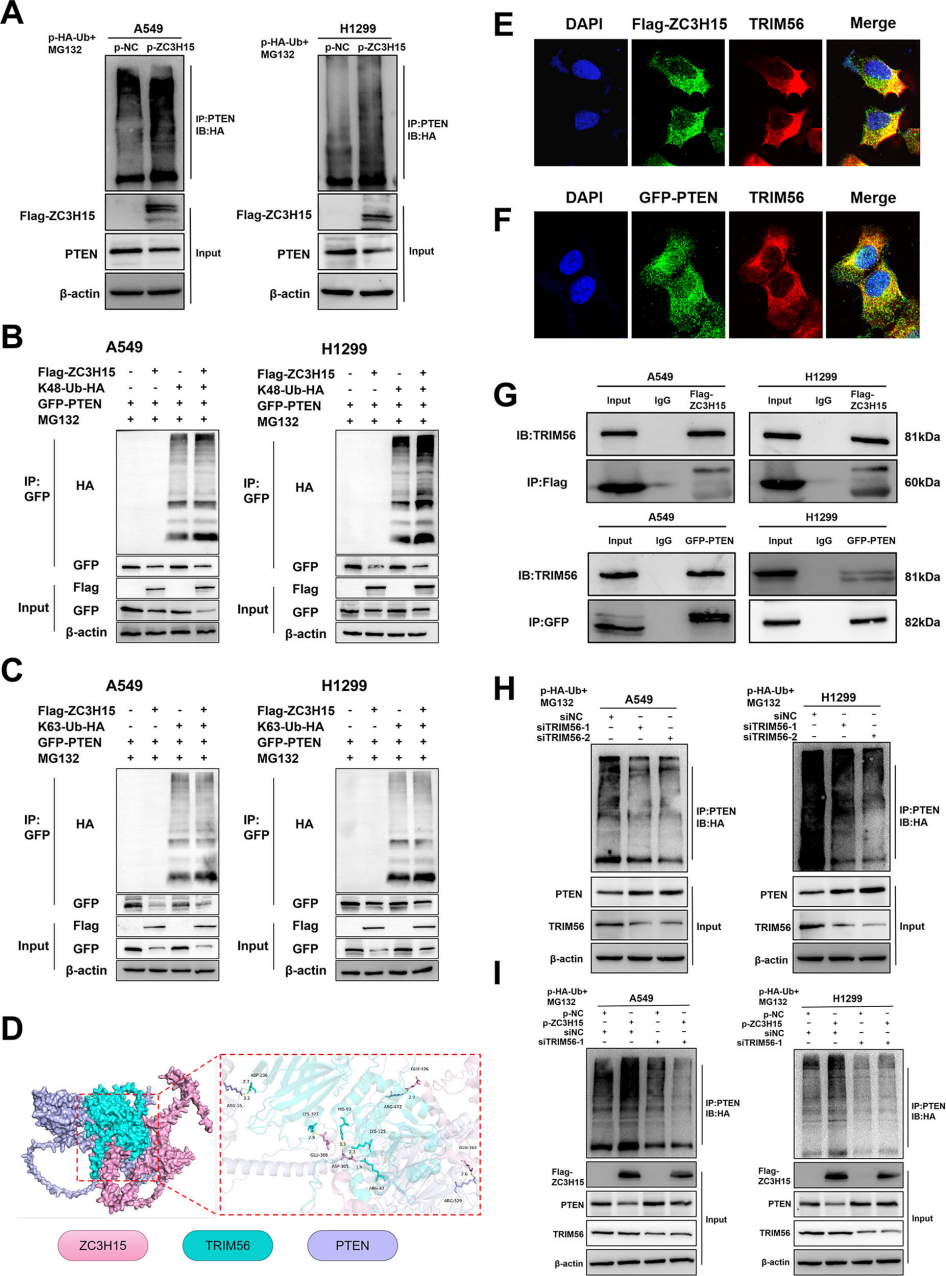
ZC3H15 recruits TRIM56 to promote PTEN ubiquitination. **A** Levels of PTEN ubiquitination were evaluated by immunoprecipitation using an anti-PTEN antibody, followed by anti-HA immunoblotting. **B**, **C** Levels of PTEN ubiquitination of A549 and H1299 transfected with K48/K63 mutant Ub were evaluated by immunoprecipitation using an anti-PTEN antibody, followed by anti-HA immunoblotting. **D** 3D Binding model analysis (ZC3H15 in pink, TRIM56 in blue and PTEN in cyan). The key residues are shown as sticks. H-bonds are shown as yellow dashed lines. **E** Confocal microscopy of triplestained Flag-ZC3H15(green), TRIM56(red) and DAPI(blue) in H1299 cells. **F** Confocal microscopy of triplestained GFP-PTEN(green), TRIM56(red) and DAPI(blue) in H1299 cells. **G** Interactions between TRIM56 with Flag-ZC3H15 and GFP-PTEN in A549 and H1299 cells measured by co-immunoprecipitation. **H** Levels of PTEN ubiquitination of A549 and H1299 with TRIM56 knockdown were evaluated by immunoprecipitation using an anti-PTEN antibody, followed by anti-HA immunoblotting. **I** Levels of PTEN ubiquitination were evaluated by immunoprecipitation using an anti-PTEN antibody, followed by anti-HA immunoblotting.

We found that PTEN mRNA expression levels were unaffected by overexpression or silencing of ZC3H15, while PTEN protein expression levels were altered (Supplementary Fig. 8A–D). Based on the results of the previous experiments, we hypothesized that ZC3H15 is involved in regulating the post-translational modifications of PTEN. Thus, we next sought to determine the specific mechanism by which ZC3H15 regulates PTEN. The two most common post-translational modifications are phosphorylation and ubiquitination. The Western blot results showed that ZC3H15 regulates both PTEN and p-PTEN protein levels. Based on the experimental findings, we speculate that the change in p-PTEN expression is caused by the alteration in total PTEN protein levels. (Supplementary Fig. 8C, D). We used cycloheximide (CHX) to block protein synthesis and found that knockdown of ZC3H15 prolonged the half-life of endogenous PTEN (Supplementary Fig. 8E). Then, we performed IP experiments using anti-PTEN antibodies and assessed PTEN ubiquitination levels by immunoblotting in A549 and H1299 (Fig. 6A). We found that upregulation of ZC3H15 promoted PTEN ubiquitination and degradation. So far, K48- and K63-linked polyubiquitin chains remain the most extensively studied and functionally characterized ubiquitin modifications [13]. We demonstrated that K48-linked ubiquitin chains, but not K63-linked chains, mediate PTEN polyubiquitination (Fig. 6B, C).

To date, no studies have shown that ZC3H15 can function as an E3 ubiquitin ligase. Furthermore, ZC3H15 does not have an E3 ubiquitin ligase-related active center structure. We analyzed the E3 ubiquitin ligase in ZC3H15 mass spectrometry data and identified TRIM56, which ranked first (Supplementary Table 1 and Supplementary Fig. 8F). We hypothesized that ZC3H15 promotes the ubiquitination of PTEN through TRIM56. Molecular docking technology, IP and IF experiments revealed that ZC3H15, TRIM56 and PTEN form a complex and co-localize in the cytoplasm (Fig. 6D–G). In addition, knockdown of TRIM56 has been shown to inhibit the ubiquitination and degradation of PTEN (Fig. 6H). Furthermore, knockdown of TRIM56 reduced PTEN ubiquitination induced by ZC3H15 overexpression (Fig. 6I). In summary, our findings suggest that ZC3H15 promotes PTEN K48-linked polyubiquitination and is dependent on TRIM56.

### Overexpression of ZC3H15 improves cisplatin resistance in vitro and in vivo

**Fig. 7:**
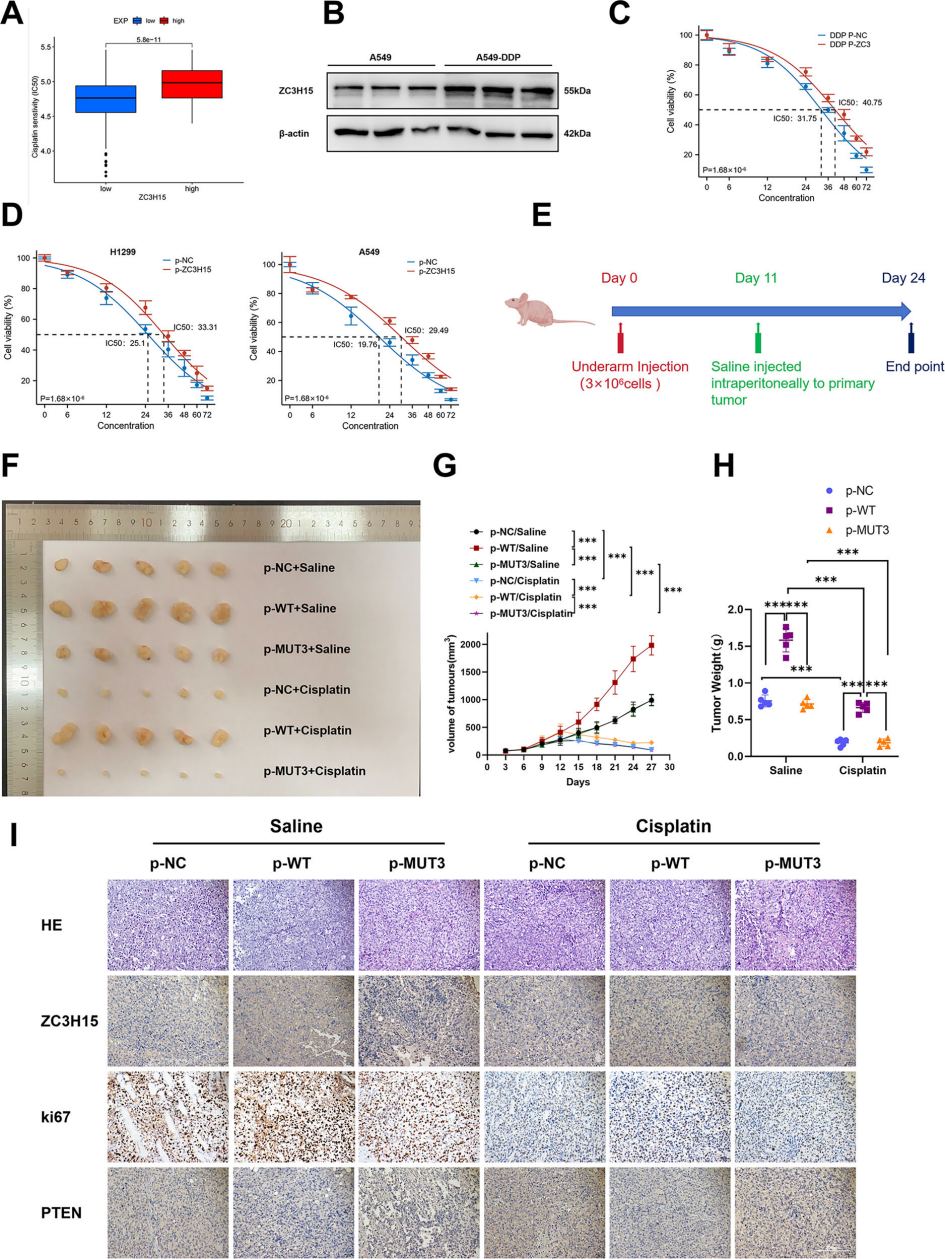
Overexpression of ZC3H15 improves cisplatin resistance in vitro and in vivo. **A** IC50 analysis of NSCLC patients with high and low ZC3H15 expression based on TCGA data. **B** Western blotting analyzing the expression of PTEN and p-PTEN in A549 and A549-DDP. **C** Viability of A549 and A549-DDP cells was analyzed by CCK8 24 h after treatment with different concentrations of cisplatin. **D** Viability of A549 and H1299 cells with ZC3H15 overexpressed was analyzed by CCK8 24 h after treatment with different concentrations of cisplatin. **E** Representative explanted tumor growth curve of mice treated as indicated (*n* = 5 per group). **F** Xenograft tumors from H1299 cells. **G** Tumor growth curves were shown. Mean ± SD, *n* = 5. ***P < 0.001. **H** Quantification of xenograft tumor weights. Mean ± SD, *n* = 5. ***P < 0.001. **I** Representative pictures of H&E and IHC staining of ZC3H15, Ki-67, and PTEN in the indicated xenograft tumors. Scale bar, 200 μm.

Drug therapy resistance is still one of the main factors affecting the survival and prognosis of lung cancer patients. Thus, we next asked whether ZC3H15 is involved in drug resistance. Drug screening was performed on the TCGA database, and revealed that high ZC3H15 expression levels were associated with cisplatin resistance (Fig. 7A). Furthermore, ZC3H15 was found to be highly expressed in A549/DDP cells, a human lung adenocarcinoma cisplatin-resistant cell strain (Fig. 7B). Importantly, ZC3H15 overexpression increased the resistance of NSCLC cells to cisplatin (Fig. 7C, D). Due to the fact that cisplatin treatment for tumors may cause cellular DNA damage, we investigated the role of ZC3H15 in DNA damage repair (DDR). γ-H2AX is a DNA double strand break marker [14]. After adding cisplatin, there was less staining of γ-H2AX in the ZC3H15 overexpressed cells compared to the control cells, indicating that ZC3H15 promoted DDR. Meanwhile, the addition of LY294002 inhibited the promoting effect of ZC3H15 on cell DDR (Supplementary Fig. 9A).

Finally, we constructed a xenograft tumor model in mice (Fig. 7E). We found that overexpression of ZC3H15 promoted tumor growth and cisplatin resistance in vivo, which was dependent on the DFRP structural domain of ZC3H15 (Fig. 7F–H). In vivo, ZC3H15 promoted proliferation and inhibited PTEN expression as detected by IHC (Fig. 7I). In addition, ZC3H15 promoted activation of the AKT signaling pathway (Supplementary Fig. 8B). Taken together, these findings suggest that high ZC3H15 expression promotes cisplatin resistance in NSCLC cells, emphasizing that ZC3H15 is a novel therapeutic target for cisplatin-treated patients.

## Discussion

Lung cancer is still the main cause of cancer deaths worldwide, accounting for about 23.8% of all cancer deaths [3]. Furthermore, ~80% of patients have already developed distant metastases at the time of diagnosis [15]. Current treatment for lung cancer patients involves surgery, radiotherapy and targeted therapy. Thus, development of novel more effective treatments is required to improve the prognosis of lung cancer patients [16].

Previous studies have reported that ZC3H15 participates in cellular homeostasis, disease occurrence and stress response through various molecular mechanisms. In the bone cancer pain model, ZC3H15 exacerbates neuronal oxidative stress through the KEAP1/NRF2 pathway and activates microglial inflammatory response through IκB α/NF - κ B activation [17]. ZC3H15 maintains telomere length homeostasis by binding to TERC RNA and interacting with telomerase. Its absence leads to abnormal retention of telomerase in Cajal bodies, hindering telomere recruitment and ultimately causing cellular aging [18]. Current research on ZC3H15 in cancer has primarily explored its role in transcriptional regulation. In gliomas, ZC3H15 suppresses CBL expression, thereby enhancing EGFR protein stability [11]. Similarly, in gastric cancer, ZC3H15 downregulates FBXW7 transcription, leading to increased c-Myc stability [12].

In this study, we found that ZC3H15 is highly expressed in NSCLC tissues, and that ZC3H15 expression levels are correlated with various clinicopathological parameters of NSCLC, including tumor size, lymph node metastasis, and TNM stage. Bioinformatics analysis and ZC3H15 staining of NSCLC patient specimens revealed that high ZC3H15 expression levels correlate with poor patient prognosis. Furthermore, we found that ZC3H15 significantly promotes NSCLC proliferation and migration by activating the AKT/mTOR signaling pathway. We identified PTEN as a binding partner of ZC3H15 and demonstrated that ZC3H15 recruits TRIM56 to promote PTEN ubiquitination and degradation. In addition, we showed that ZC3H15 interacts with PTEN via its DFRP structural domain, which is essential for regulating its stability. Our study reveals a previously uncharacterized role of ZC3H15 in regulating K48-linked polyubiquitination of PTEN. Mechanistically, the ZC3H15/PTEN/TRIM56 protein complex enhances AKT/mTOR signaling activation, thereby promoting malignant progression in NSCLC. These findings not only expand the functional diversity of ZC3H15 but also provide a molecular basis for targeting ZC3H15 in NSCLC therapy.

The AKT/mTOR signaling pathway plays a key role in the development of multiple cancers by regulating cell growth, proliferation, metabolism and apoptosis [19–22]. In NSCLC, aberrant activation of the PI3K-AKT-mTOR signaling pathway is closely associated with tumor aggressiveness, chemotherapy resistance, and poor prognosis [23–26]. Studies have shown that activation of this pathway can promote the proliferation and survival of lung cancer cells through multiple mechanisms, including the regulation of cell cycle proteins and apoptotic proteins [27, 28]. Here, we showed using data obtained from the GSEA database that ZC3H15 was associated with the AKT-mTOR signaling pathway and further verified these findings experimentally by showing that ZC3H15 promotes the expression of p-AKT and p-mTOR proteins, which leads to increased proliferation, invasion and migration of NSCLC cells. In addition, by using the AKT pathway inhibitor LY294002, we further demonstrated that the effects of ZC3H15 overexpression could be reversed by inhibiting the AKT-mTOR signaling pathway. Although the AKT-mTOR signaling pathway is the main pathway mediating the effects of ZC3H15, other pathways may be involved. Thus, exploring the cross-talk between ZC3H15 and other signaling pathways, such as the Kras and TGFβ pathways (Supplementary Fig. 9C, D), in future studies will help to fully understand its complex mechanisms in tumor development.

Although we have demonstrated the role of ZC3H15 in promoting tumor proliferation and migration by regulating the AKT signaling pathway in lung cancer. It is worth noting that TCGA database analysis shows that ZC3H15 mRNA is significantly overexpressed in various malignant tumors, including lung adenocarcinoma. This discovery suggests that the regulation of the AKT-mTOR signaling pathway by ZC3H15 may have broader implications in tumor biology. Based on this, we plan to systematically explore the regulatory role of ZC3H15 on the AKT-mTOR pathway in different tumor types, as well as the tissue-specific differences and molecular basis of this regulatory mechanism in subsequent studies. These studies will help clarify whether ZC3H15 acts as a pan cancer molecule driving tumor progression.

In the present study, we utilized A549 cells harboring the KRASG12S mutation [29]. Our findings demonstrate that ZC3H15 promotes lung cancer progression through activation of the AKT-mTOR pathway in these cells, suggesting a potential functional interaction with oncogenic KRAS signaling. Given that KRAS acts upstream of AKT and is frequently mutated in lung cancer [30], combined targeting of KRAS and ZC3H15 may represent a promising therapeutic approach. BI-2865, as a pan-KRAS inhibitor [31, 32], will be employed in our subsequent studies to systematically investigate potential synergistic effects with ZC3H15 knockdown. This line of investigation may provide novel strategies for precision therapy in KRAS-driven cancers.

Numerous studies have shown that RNA-based drugs, including mRNA, shRNA, siRNA, and ASO, have been approved to enter the clinical trial stage or have been applied in clinical therapeutic practice [33–35]. In this study, our experiments revealed that the proliferation and migration abilities of NSCLC cells were significantly inhibited after knocking down ZC3H15 with shRNA. This would be a feasible approach for the treatment of NSCLC. However, the drug delivery mode, stability and specificity are still issues that need to be urgently addressed. Therefore, ZC3H15 may be a potential target for the development of small molecule drugs to overcome metastasis and cisplatin resistance, and may provide clues for the treatment of future patients.

Here, we demonstrated for the first time that ZC3H15 is an interacting protein of PTEN. In addition, we show that ZC3H15 forms a complex with TRIM56 and PTEN, and that ZC3H15 promotes the ubiquitination and degradation of PTEN by recruiting TRIM56. AutoDock Vina is an open-source molecular docking program that predicts binding modes and affinities by simulating the flexible docking between ligands and receptors [36, 37]. We investigated the molecular-level binding between ZC3H15 and PTEN, as well as between ZC3H15 and TRIM56. It is worth noting that GLU-308 and ASP-305 on ZC3H15 can form four hydrogen bonds with LYS-327, HIS-93, LYS-125 and ARG-47 on PTEN. Meanwhile, GLU-426 and GLU-364 on ZC3H15 form two hydrogen bonds with ARG-472 and ARG-529 on TRIM56. Furthermore, ARG-55 on TRIM56 can form a hydrogen bond with ASP-236 on PTEN. By simulating the binding of proteins through this program, we can explore the interactions between proteins. Virtual screening of small molecule drugs targeting the docking sites will be conducted, and small molecule drugs that can target the binding sites of proteins will be identified, which will help in the development of new therapeutic strategies, such as using small molecule inhibitors to block the interactions between ZC3H15, PTEN and TRIM56.

Although this study confirmed that ZC3H15 promotes K48 ubiquitination modification of PTEN, the specific ubiquitination site of PTEN still needs further analysis. In addition, future functional recovery experiments using the PTEN K48R mutant will help clarify the role of PTEN K48 ubiquitination in ZC3H15 induced lung cancer progression. Nevertheless, the findings of this study further deepen our understanding of the biological function of ZC3H15 in lung cancer and suggest that it promotes malignant progression of non-small cell lung cancer by regulating PTEN protein stability.

Although this study focused on NSCLC, ZC3H15 is a multifunctional protein that may play an important role in other types of tumors. Future studies could be extended to other tumor types to explore the role of ZC3H15 in tumor heterogeneity and metastasis, as well as its potential as a pan-cancer marker. Although the present study provides strong evidence for the role of ZC3H15 in NSCLC, some limitations remain. This study mainly relied on bioinformatics analysis, in vitro experiments and animal experiments. However, cell line models cannot fully simulate the complexity of the tumor microenvironment. Although we partially compensated for this deficiency through xenograft tumor models, there is still a lack of large-scale clinical sample validation, and further verification of the function of ZC3H15 is needed in primary tumor samples.

In summary, this study describes for the first time the biological function and clinical significance of ZC3H15 in NSCLC. ZC3H15 affects the ubiquitination of PTEN by recruiting TRIM56, which promotes the malignant progression of NSCLC. Therefore, ZC3H15 may be a potentially valuable diagnostic biomarker for NSCLC and may serve as a new target for NSCLC treatment in the future.

## Conclusion

**Fig. 8:**
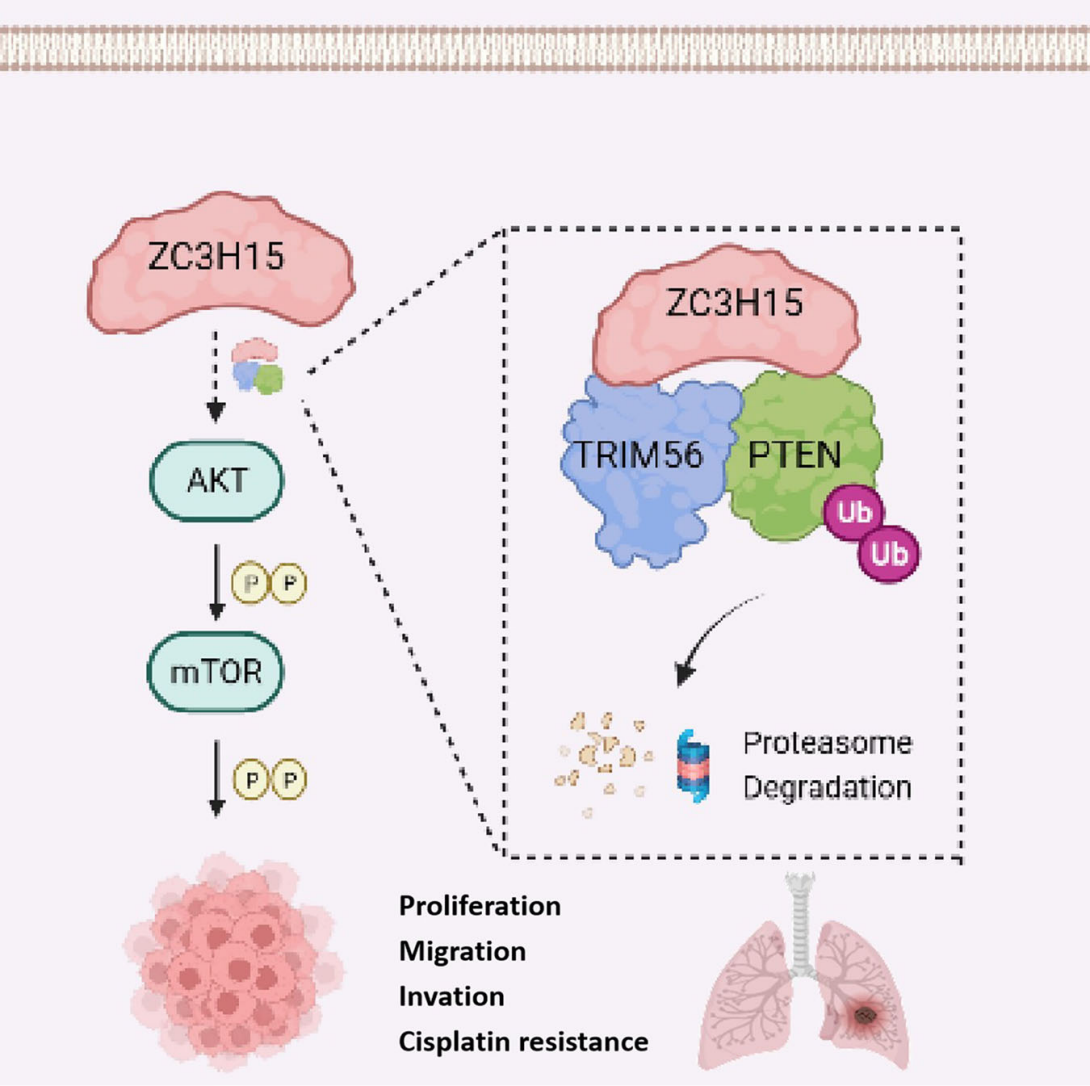
A schematic diagram illustrating the proposed mechanism of ZC3H15 in NSCLC. ZC3H15 promotes the proliferation, migration, invasion and chemotherapy resistance of NSCLC by mediating PTEN ubiquitination degradation and activating the AKT-mTOR signaling pathway.

This study reveals the important role of ZC3H15 in NSCLC, and shows that high ZC3H15 expression levels are closely associated with malignant tumor progression and poor prognosis. ZC3H15 enhances the proliferation, migration and invasion of NSCLC cells by interacting with PTEN and promoting its ubiquitination and degradation, which in turn activates the AKT-mTOR signaling pathway. In addition, we show that ZC3H15 is involved in the resistance of NSCLC to the chemotherapeutic drug cisplatin. These findings provide a scientific basis for the development of targeted therapeutic strategies against ZC3H15, which may improve the prognosis of NSCLC patients (Fig. 8).

## Materials and methods

### Patients and specimens

This retrospective analysis focused on 164 patients diagnosed with NSCLC at the First Affiliated Hospital of China Medical University (Shenyang City, Liaoning Province, China) between 2017 and 2020. These patients did not display any driver gene mutations and were treatment-naïve, meaning they had not undergone radiotherapy or chemotherapy. Following surgical intervention, eight pairs of NSCLC tumors along with adjacent lung tissues were randomly chosen for further examination through immunoblot analysis. The research protocol was granted approval by the Medical Research Ethics Committee associated with China Medical University. All participants provided their informed consent for the study.

### Bioinformatics analysis

TIMER (https://cistrome.shinyapps.io/timer) was used to identify differentially expressed genes between tumors and normal tissues, and box plots along with the Wilcoxon test method were employed to illustrate gene expression levels. The Xiantao Academic TCGA database website (https://www.xiantao.love) was utilized to statistically analyze the correlation between ZC3H15 expression and the T and N staging of lung cancer, employing the Kruskal–Wallis test, Dunn’s multiple hypothesis test, and the Mann–Whitney U test. The Cox regression method of R software (version 4.2.1) was used to analyze the impact of high ZC3H15 expression on lung cancer prognosis. Potential protein interaction partners of ZC3H15 were identified by searching the BioGRID, HALLMARK, WP, and KEGG databases, to uncover associations between ZC3H15 and specific signaling pathways.

### Cell culture and treatment

The following cell lines were used in this study: HBE, SK-MES-1, A549, A549/DDP, NCI-H1299, NCI-H460, and NCI-H1975. The A549, A549/DDP and NCI-H1299 cell lines were purchased from Wuhan Pricella Biotechnology Co., Ltd. (Wuhan,China), while the remaining cell lines were obtained from the cell bank of Shanghai Institutes of Biological Sciences (Shanghai, China). HBE cells were cultured in high-glucose DMEM medium, SK-MES-1 in MEM medium, A549, A549/DDP in Ham’s F-12K medium, and NCI-H1299, NCI-H460, and NCI-H1975 in RPMI-1640 medium. All media were supplemented with 10% fetal bovine serum (Clark Biosciences, Richmond, VA, USA). Cells were maintained in a humidified incubator with 5% CO_2_ at a temperature of 37 °C. All human cell lines were authenticated using Short Tandem Repeat (STR) profiling.

For inhibitor assay, cells were treated with the AKT-mTOR pathway inhibitor LY294002 (40 μM; MedChemExpress, Monmouth Junction, NJ, USA) [38] or the PTEN inhibitor VO-Ohpic trihydrate (50 nM; MedChemExpress) and incubated for 24 h. All inhibitor concentrations were selected based on established efficacy from previous studies and our preliminary dose-response experiments.

### Immunohistochemistry

Immunohistochemical assays were performed as described previously. Slides were incubated overnight with polyclonal rabbit-derived ZC3H15 antibody (26241-1-AP, 1:200, Proteintech, China) at 4 °C.

### Plasmid transfection and viral infection

The pLV3-CMV-ZC3H15-Flag-Puro, pCDH-CMV-MCS-EF1-Puro and pLV3-U6-ZC3H15-shRNA1-3-EGFP-Puro vectors were purchased from Miaoling Biosciences (Wuhan, China). Cells stably overexpressing and silencing ZC3H15 expression were screened using puromycin (SigmaAldrich). The target sequences of ZC3H15 shRNA were as follows: shZC3H15-1: 5’-CCTTTAGTACACTGTCCTTGC-3’; shZC3H15-2: 5’-CCTTTCTTATTCTTCAAACCG-3’; and shZC3H15-3: 5’-GCATCAATGTAAACACTTCGC-3’. The target sequences of TRIM56 siRNA were: siTRIM56-1: 5’-CCAGAAGGAUGGUGGGAAATT-3’; and siTRIM56-2: 5’-UGACCCUUCGAGAAGUCAATT-3’. Lipofectamine 3000 (Invitrogen, Carlsbad, CA, USA) was used for transient transfections. Stable transfections were screened using puromycin.

### Western blotting

Western blot analysis was carried out as described previously [39]. The primary antibody dilution ratios are shown in Table 2. Glyceraldehyde-3-phosphate dehydrogenase (GAPDH) was used as the relative loading control.

**Table 2:** Antibodies for western blot.

Target	Catalog number	Source	Dilution
ZC3H15 (IHC)	#NBP1-81312	Novus Biologicals	1:500
ZC3H15 (WB)	#26241-1-AP	Proteintech	1:1000
ZC3H15 (IF)	#26241-1-AP	Proteintech	1:1000
β-actin	#GB11001	Servicebio	1:1000
CyclinD1	#60186-1-lg	Proteintech	1:5000
CDK4	#11026-1-AP	Proteintech	1:1000
CDK6	#14052-1-AP	Proteintech	1:1000
E-cadherin	#M025415	Abmart	1:2000
N-cadherin	#T55015	Abmart	1:1000
MMP9	#10375-2-AP	Proteintech	1:1000
DYKDDDDK	#66008-4-lg	Proteintech	1:1000
IgG	#ZB2305	ZSGB-Bio	1:2000
IgG	#ZB-2301	ZSGB-Bio	1:2000
PI3K-p85ɑ	#T40115	Abmart	1:1000
PI3K-p110ɑ	#27921-1-AP	Proteintech	1:1000
p-AKT (S473)	#T40067	Abmart	1:1000
mTOR	#T55306	Abmart	1:1000
p-mTOR	#T56571	Abmart	1:1000
p-EGFR (Y1068)	#3777T	Cell Signaling Technology (CST)	1:1000
p-NF-κB	#3033T	Cell Signaling Technology (CST)	1:1000
p-p38	#28796-1-AP	Proteintech	1:2000
β-catenin	#51067-2-AP	Proteintech	1:5000
PTEN	#22034-1-AP	Proteintech	1:3000
p-PTEN	#9551T	Cell Signaling Technology (CST)	1:1000
TRIM56	#R25985	ZEN-BIOSCIENCE	1:500
GFP	#66002-1-lg	Proteintech	1:20000
HA	#66006-2-lg	Proteintech	1:10000

### Real-time PCR

Total RNA was isolated from cells using an RNA Extraction Kit (RC112-01, Vazyme Biotech Co., Nanjing, China) according to the manufacturer’s instructions. Reverse transcription was carried out using a cDNA Synthesis Kit (Vazyme Biotech Co.) and 1 μg RNA as the input material. Quantitative RT-PCR assays were carried out using SYBR Green PCR Master Mix, in a reaction volume of 20 μl, on the 7900HT Fast Real-Time PCR System (Applied Biosystems, Foster City, CA, USA). The thermal cycling conditions were as follows: an initial hold at 95 °C for 30 s; for the PCR stage, the first step was at 95 °C for 10 s, and the second step was at 60 °C for 30 s, which was repeated for a total of 40 cycles; the melt stage included a hold at 95 °C for 15 s followed by a hold at 60 °C for 60 s. Relative gene expression levels were determined with respect to β-actin using the 2^−ΔΔCt^ calculation method. The primers for real-time PCR are listed in Table 3.

**Table 3:** Primers for real-time PCR.

Genes	Primers (5’–3’)
ZC3H15-Forward	TCCCATGACTTGACTCTGGAG
ZC3H15-Reverse	ACCGTGCTTCTTGTTCACTAC
PTEN-Forward	CCCAGTTTGTGGTCTGCCAGC
PTEN-Reverse	ATGAGCTTGTCCTCCCGCCGC
β-actin-Forward	GGGAAATCGTGCGTGACATT
β-actin-Reverse	AGGTAGTTTCGTGGATGCCA

### CCK-8 assay

A549 and NCI-H1299 cells lines were placed in 96-well plates (2500 cells/100 μl specific media per well; five replicates) and incubated at 37 °C. At various time points, CCK-8 reagent was added to a plate, which was then wrapped in foil and returned to the incubator. After 2 h incubation, OD values were measured at 450 nm using a microplate reader. The experiment was repeated three times to ensure accuracy.

### Colony formation assay

Complete medium (4 ml) containing 500 cells was added to each well of a six-well plate and incubated at 37 °C. Cell growth was observed every 3–5 days until visible colonies formed after ~12 days for A549 cells and 10 days for NCI-H1299 cells. Cells were washed three times with PBS, fixed in cold methanol, stained with crystal violet for 20 min, rinsed with ddH_2_O, air dried, and photographed.

### EdU staining

Stable cell lines were cultured on coverslips in 24-well plates with complete medium. Cells were incubated with 2 × EdU working solution for 1.5 h. Then, samples were fixed in 4% paraformaldehyde, washed with PBS and permeabilized with 0.3% Triton X-100. Cells were then incubated with the Click reaction mixture containing specific reagents and washed. Cell nuclei were stained with Hoechst 33342 and washed again. Images were captured using a confocal microscope.

### Wound-healing assay

ZC3H15-transfected A549 and NCI-H1299 cells were cultured in six-well plates. The medium was replaced with serum-free mitomycin-containing medium, and cells were incubated for 2 h at 37 °C. After removing the serum-free mitomycin-containing medium and washing with PBS, fresh serum-free medium was added. A 200 μl pipette tip was used to scratch a “+“-shaped wound through the cells. The plate was marked with observation points, imaged, and disinfected before incubation. Images were captured at 6, 12, and 24 h post-incubation, with PBS washes before each, and the experiment was repeated three times.

### Transwell migration and invasion assays

For the migration assay, lung cancer cells (4*10^5^ cells/200 μl) were cultured in Transwell chambers (8-μm pore size, Corning, Inc., Corning, NY, USA) for 24 h. For the invasion assay, the membranes in the upper chamber of each Transwell insert were coated with Matrigel basement membrane matrix (BD Biosciences), then the steps described above for the migration assay were carried out. Cells that crossed the Transwell membrane were stained with crystal violet, counted manually, and photographed.

### Immunofluorescence

Assays were performed as described previously [39].

### Co-immunoprecipitation assays

Cells were cultured in 10-cm cell dishes until they reached confluence, then lysed using lysis buffer. The lysate was centrifuged at 13,400 × *g* for 20 min at 4 °C to separate the components. Protein A/G agarose (Beyotime Biotechnology, P2012) was added to the resulting supernatant in a volume of 40 μL and incubated for 4 h. Then, the supernatant was centrifuged at 1000 rpm for 5 min at 4 °C. The supernatant was subsequently split into two equal portions. The specific target antibody was added to one portion at a concentration of 6 μg. Anti-rabbit IgG (Beyotime Biotechnology; 1:5000) was added to the second portion. The samples were incubated with continuous shaking overnight at 4 °C. The next day, samples were incubated with 25 μL agarose A/G magnetic beads for 6 h at 4 °C. After incubation, the magnetic beads were washed three times using a lysis buffer. Finally, the beads were subjected to continuous heating in boiling water for 10 min prior to immunoblotting analysis.

### Molecular docking

The sequences of the target proteins were obtained from Uniport and subsequently modeled and docked using AlphaFold3, an advanced deep-learning-based protein structure prediction tool. AlphaFold3 employs an end-to-end transformer-based architecture, leveraging both evolutionary multiple sequence alignments (MSAs) and physical constraints to predict highly accurate three-dimensional protein structures. The docking results were analyzed and visualized using PyMOL 2.6.1, a widely used molecular visualization tool. The final complex structures were rendered in high-resolution three-dimensional graphics, highlighting key interactions.

### Ubiquitination assays and immunoprecipitation

Stably transfected cell lines were transfected with the Ub-HA or K48/K63 mutant Ub plasmid generously gifted by Professor Li Qingchang affiliated with China Medical University. Subsequently, cells were treated with the 26 S proteasome inhibitor MG132 (MedChemExpress, Monmouth Junction, NJ, USA) at a final concentration of 20 μM. Co-immunoprecipitation was performed. The obtained immune complexes were washed with cell lysis buffer, then subjected to immunoblotting analysis.

### Cell-derived xenograft model

Four-week-old nude mice were obtained from Beijing Vital River Laboratory Animal Co., Ltd and acclimated for one week prior to the experiment. Mice were randomly divided into two groups: a control LV-NC group and an LV-ZC3H15 overexpression group, containing five mice per group. Mice were injected with ~1 × 10^7^ A549 stably-transfected cells in serum-free medium into the mid-posterior region of the axilla. After 6 weeks, when the tumors in the right axillary regions were significantly enlarged but still within ethical guidelines, all mice were humanely killed by cervical dislocation, and tumor samples were collected and measured.

### Statistical analysis

Statistical analyses were carried out utilizing the SPSS 19.0 software package (IBM, Armonk, NY, USA) or GraphPad Prism 6.0 software (GraphPad Software, Inc., San Diego, CA, USA). The relationship between ZC3H15 expression levels and various clinical and pathological features was assessed using the cardinality test. For group comparisons, statistical significance was determined using either two-tailed unpaired *t*-tests or one-way ANOVA (analysis of variance) tests. Each experiment was conducted independently and repeated a minimum of three times under identical conditions. Statistical significance was defined as *P* values less than 0.05.

## Supplementary information

Supplementary Figure1

Supplementary Figure2

Supplementary Figure3

Supplementary Figure4

Supplementary Figure5

Supplementary Figure6

Supplementary Figure7

Supplementary Figure8

Supplementary Figure9

clean Additional files

Supplementary Table 1

full length blots

## Data Availability

The datasets used and analyzed during the current study are available from the corresponding author on reasonable request.
