# Supplementary figures and images for: ZC3H15 regulates the ubiquitination of PTEN via recruitment of TRIM56 and promotes malignant progression of non-small cell lung cancer
Source: Cell Death Dis. 2026 Jan 9;17(1):17. doi: 10.1038/s41419-025-08138-2 (PMC12789496; doi:10.1038/s41419-025-08138-2)

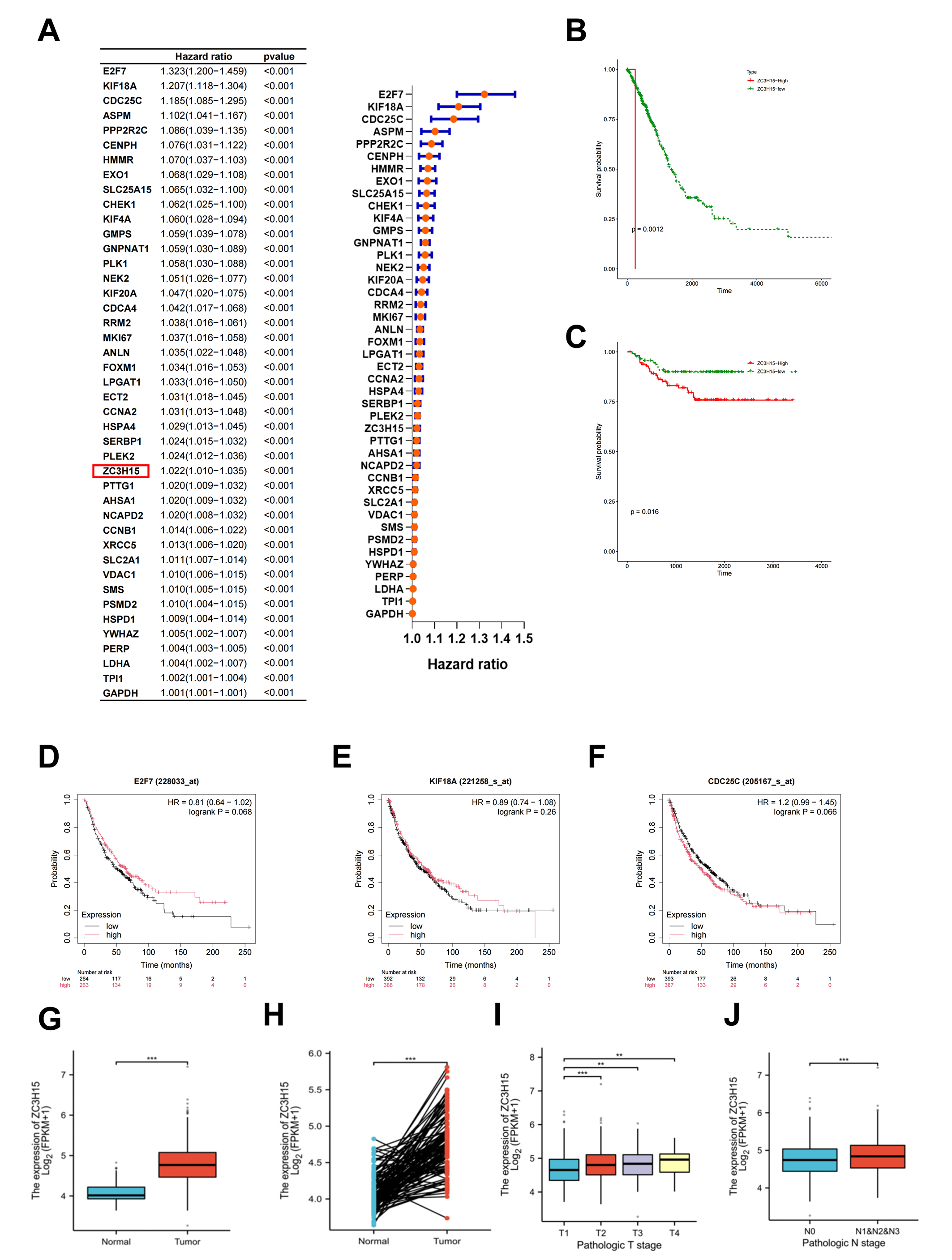

Supplement: Supplementary file 1 — Supplementary Figure1 [file 41419_2025_8138_MOESM1_ESM.png]

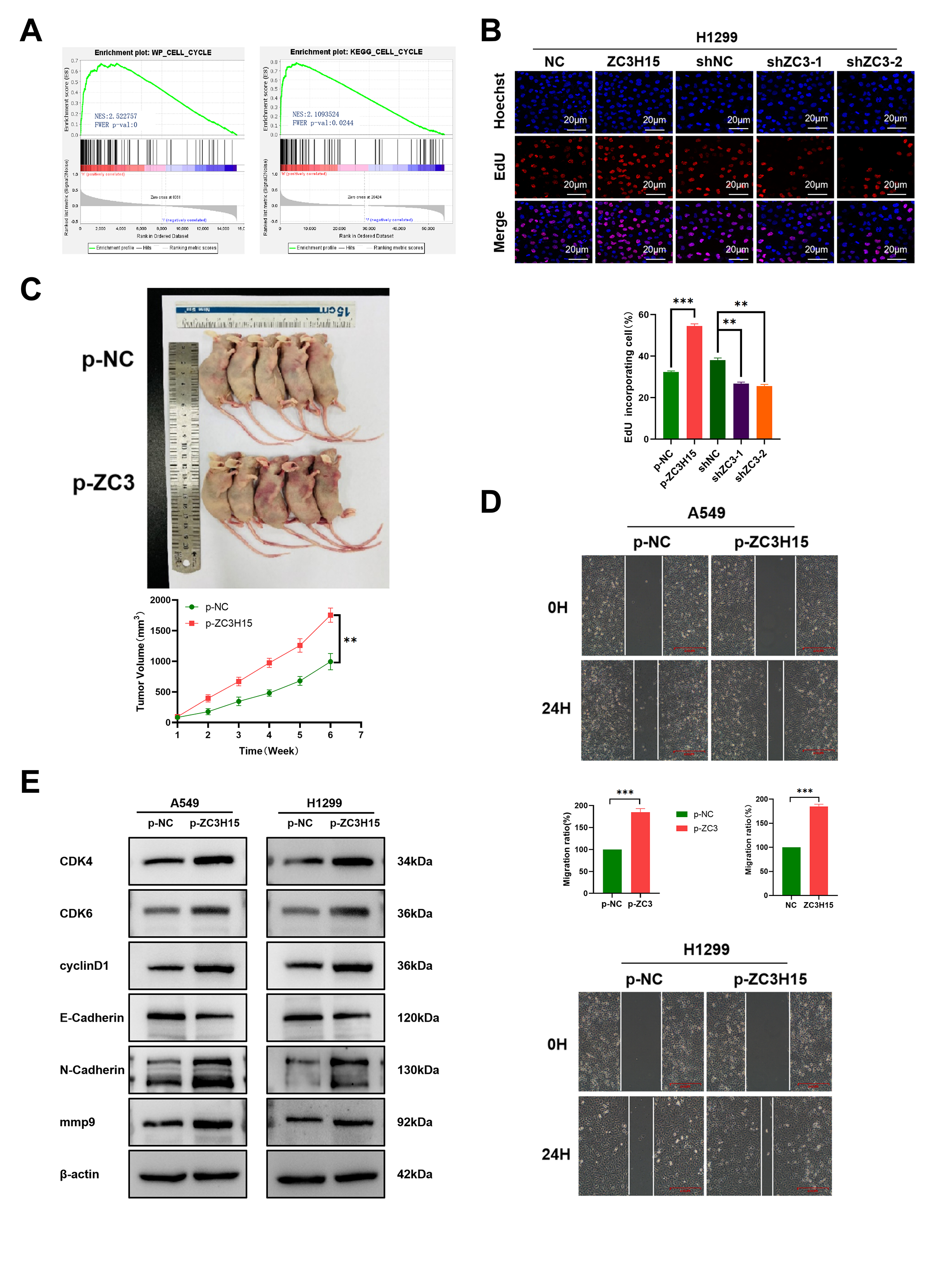

Supplement: Supplementary file 2 — Supplementary Figure2 [file 41419_2025_8138_MOESM2_ESM.png]

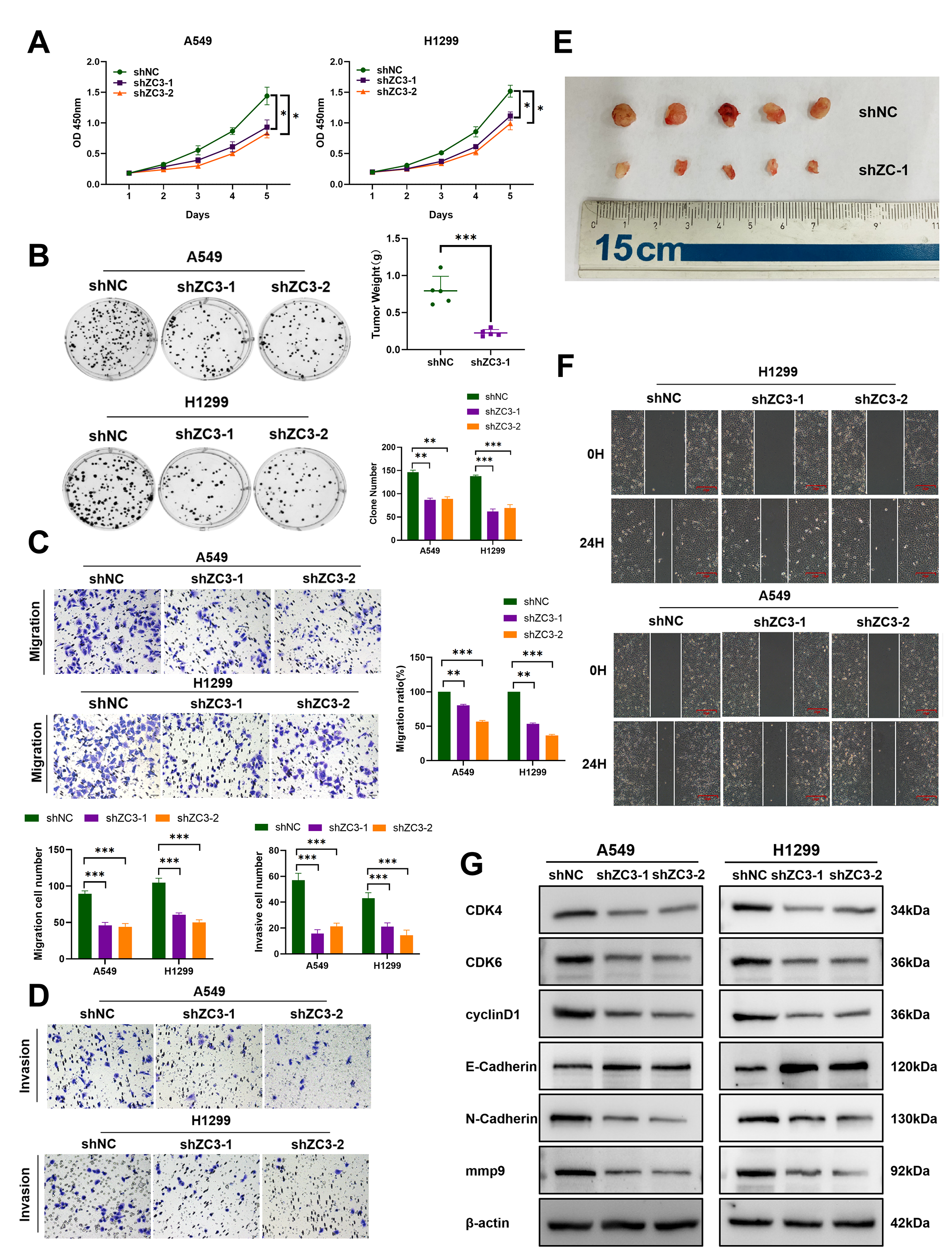

Supplement: Supplementary file 3 — Supplementary Figure3 [file 41419_2025_8138_MOESM3_ESM.png]

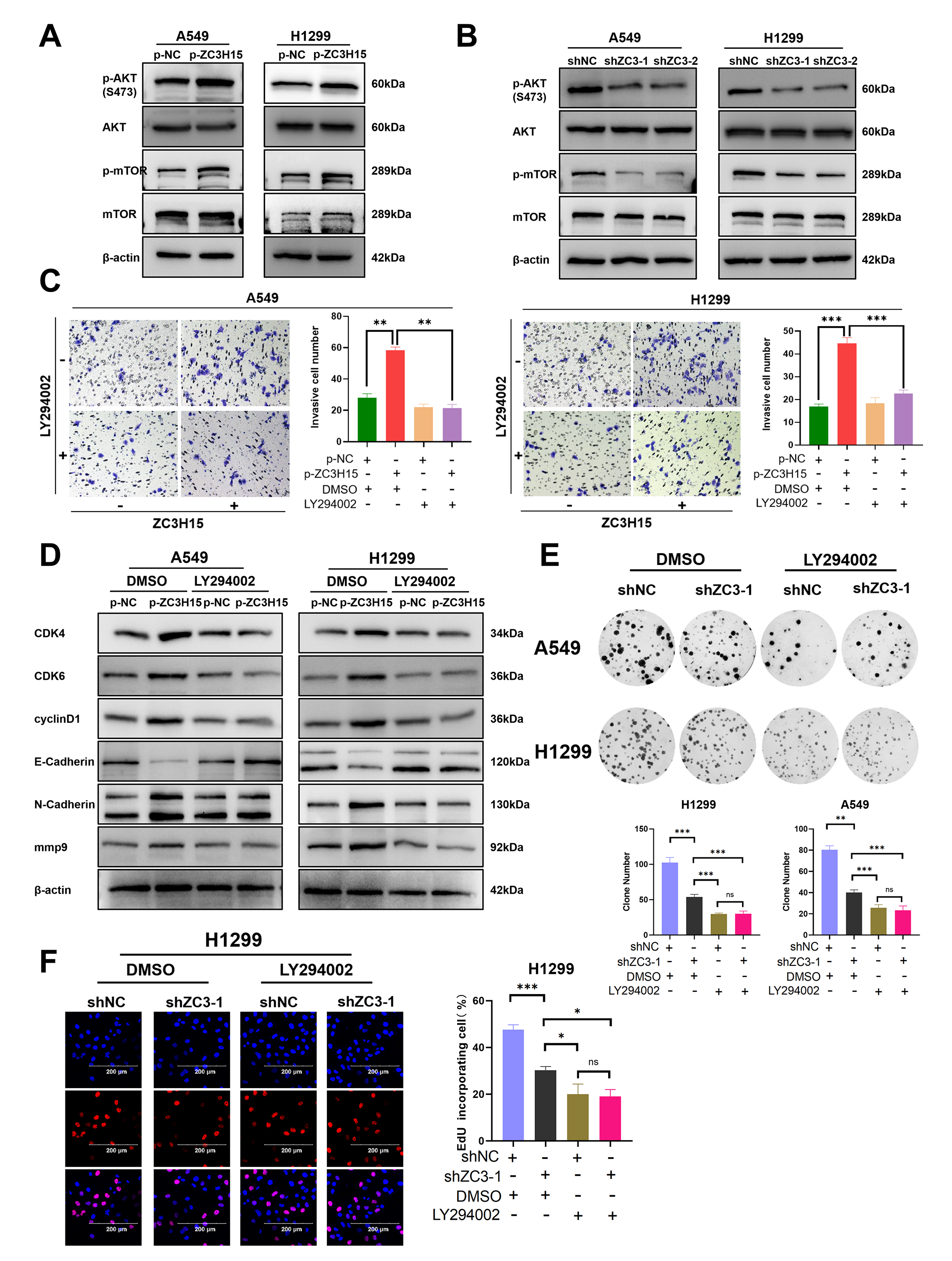

Supplement: Supplementary file 4 — Supplementary Figure4 [file 41419_2025_8138_MOESM4_ESM.png]

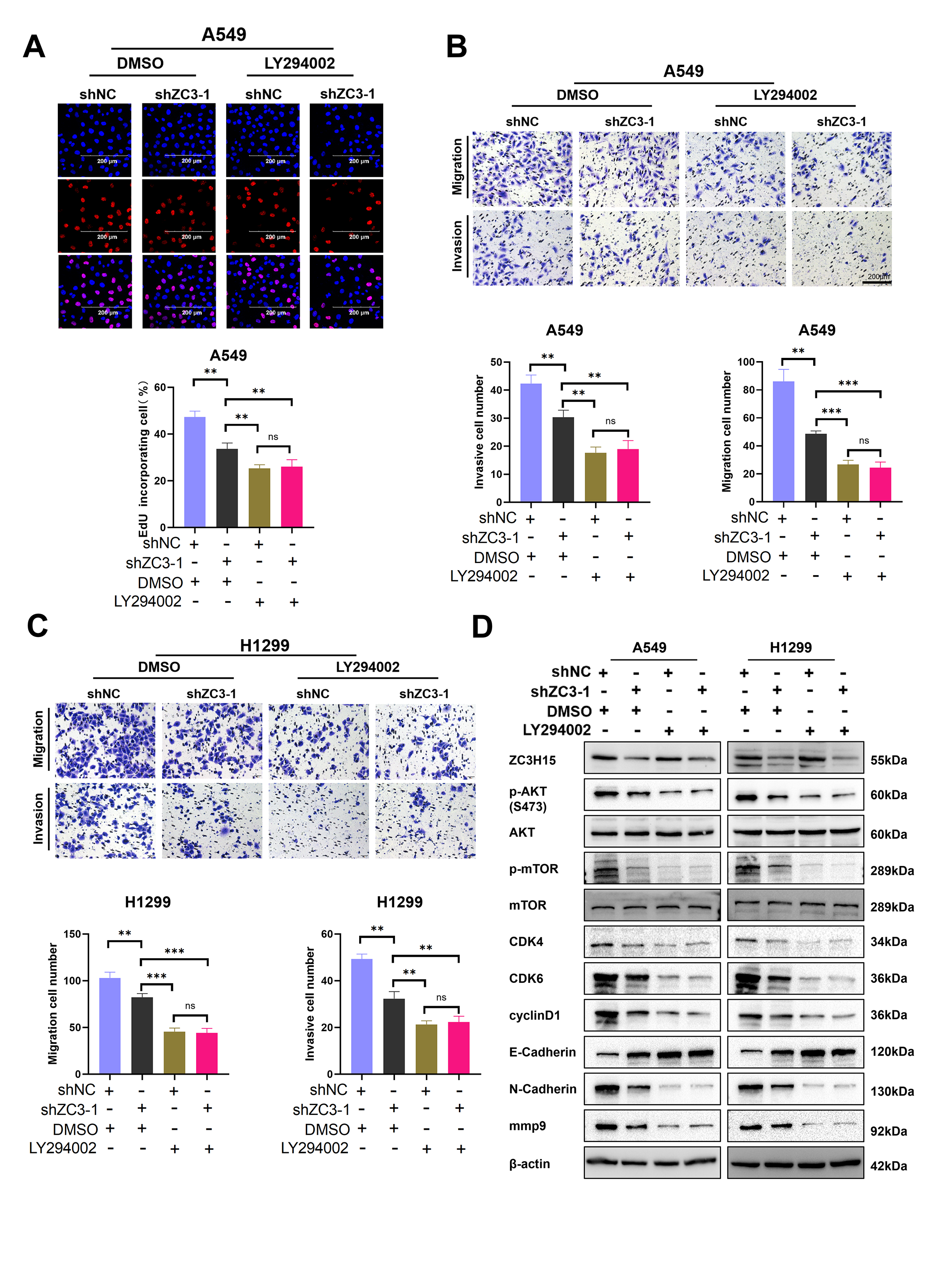

Supplement: Supplementary file 5 — Supplementary Figure5 [file 41419_2025_8138_MOESM5_ESM.png]

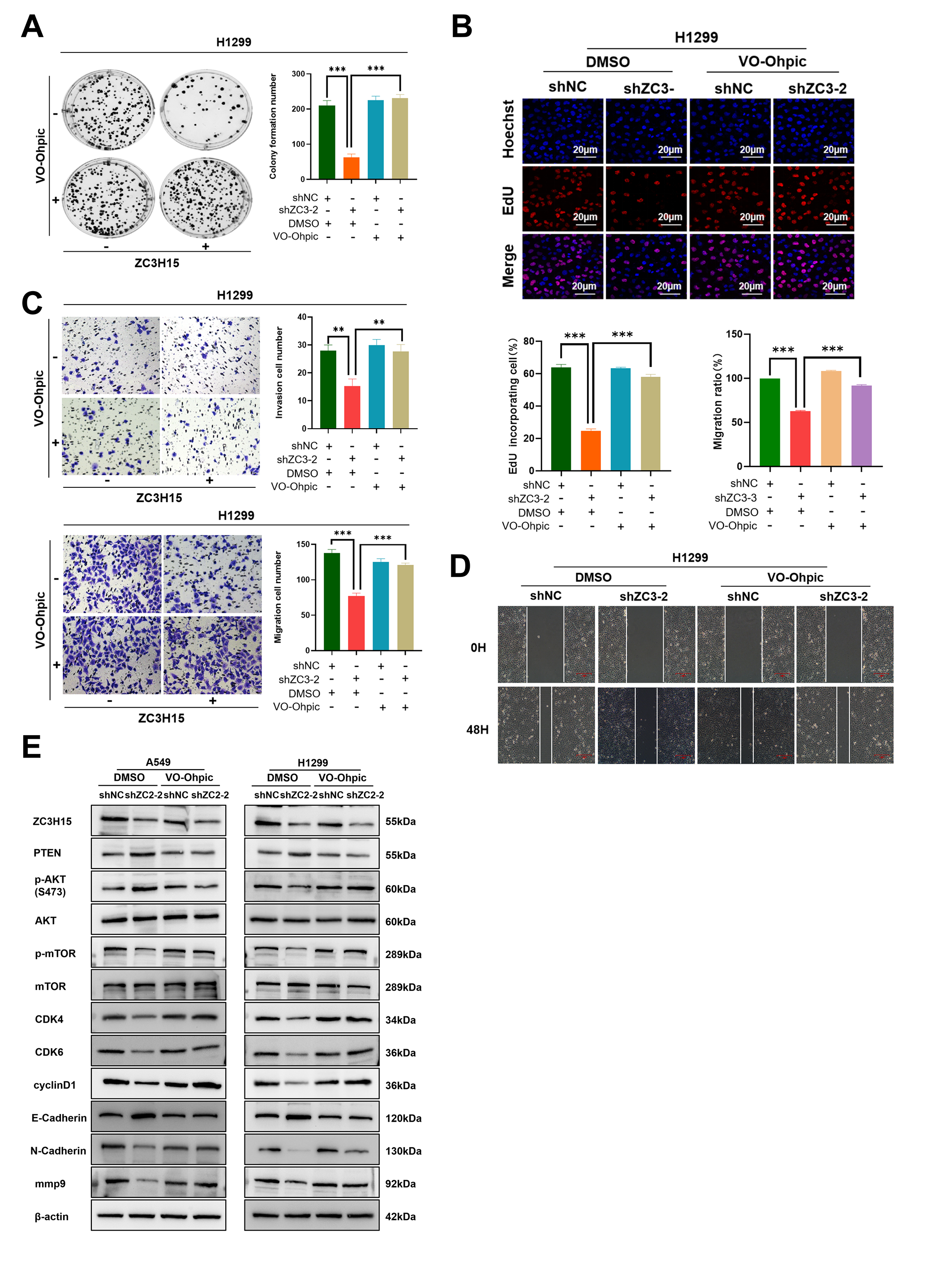

Supplement: Supplementary file 6 — Supplementary Figure6 [file 41419_2025_8138_MOESM6_ESM.png]

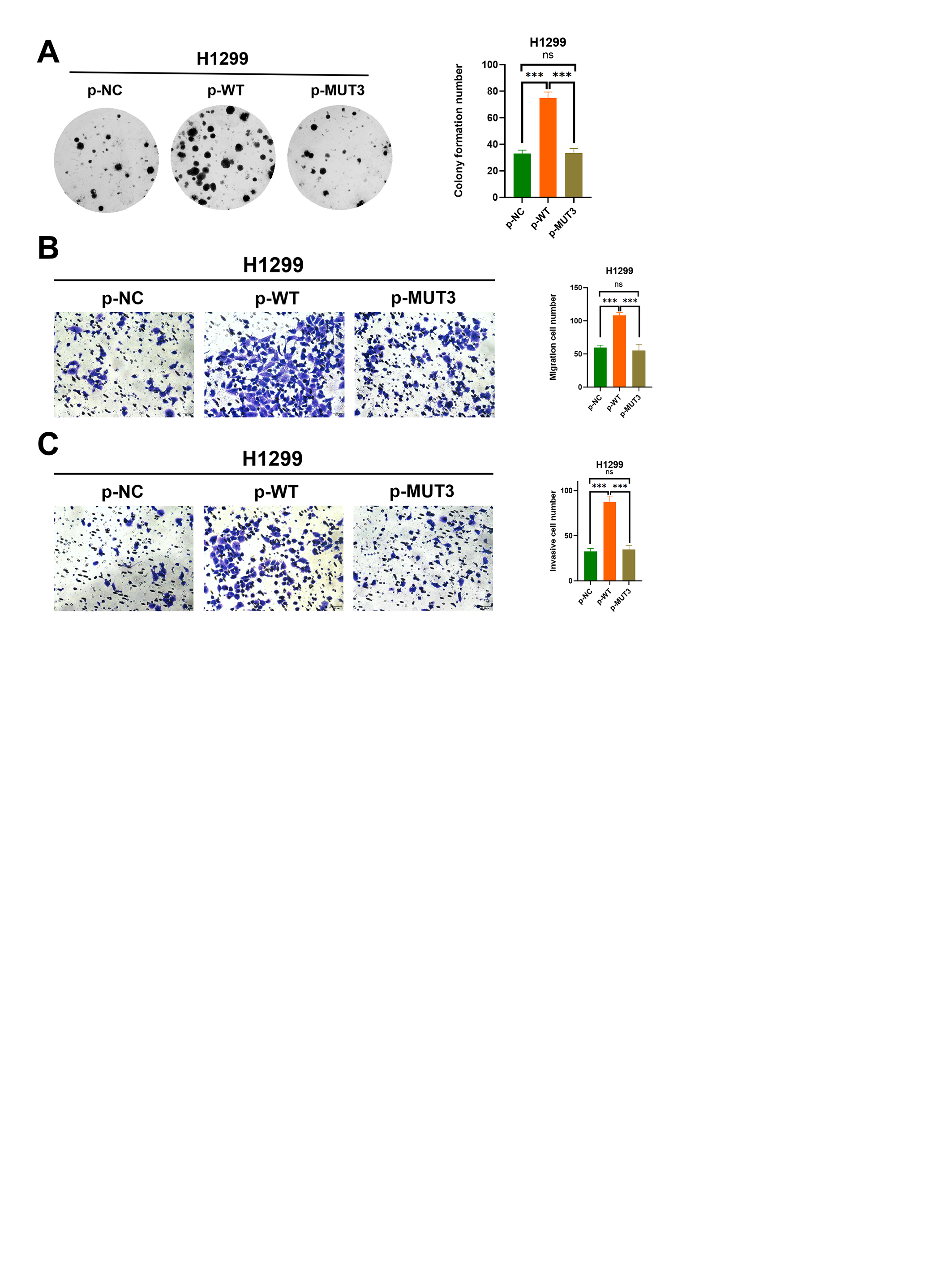

Supplement: Supplementary file 7 — Supplementary Figure7 [file 41419_2025_8138_MOESM7_ESM.png]

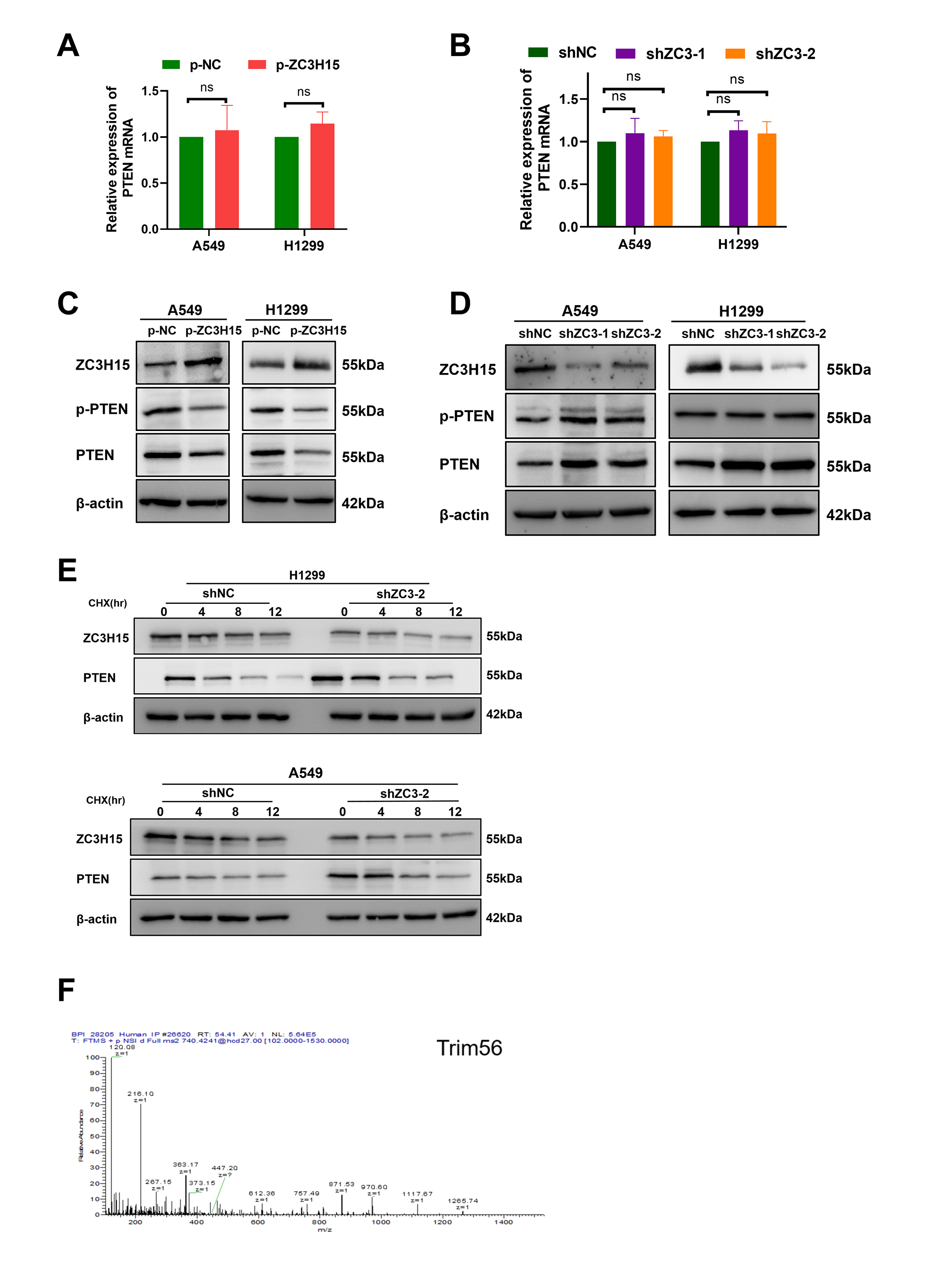

Supplement: Supplementary file 8 — Supplementary Figure8 [file 41419_2025_8138_MOESM8_ESM.png]

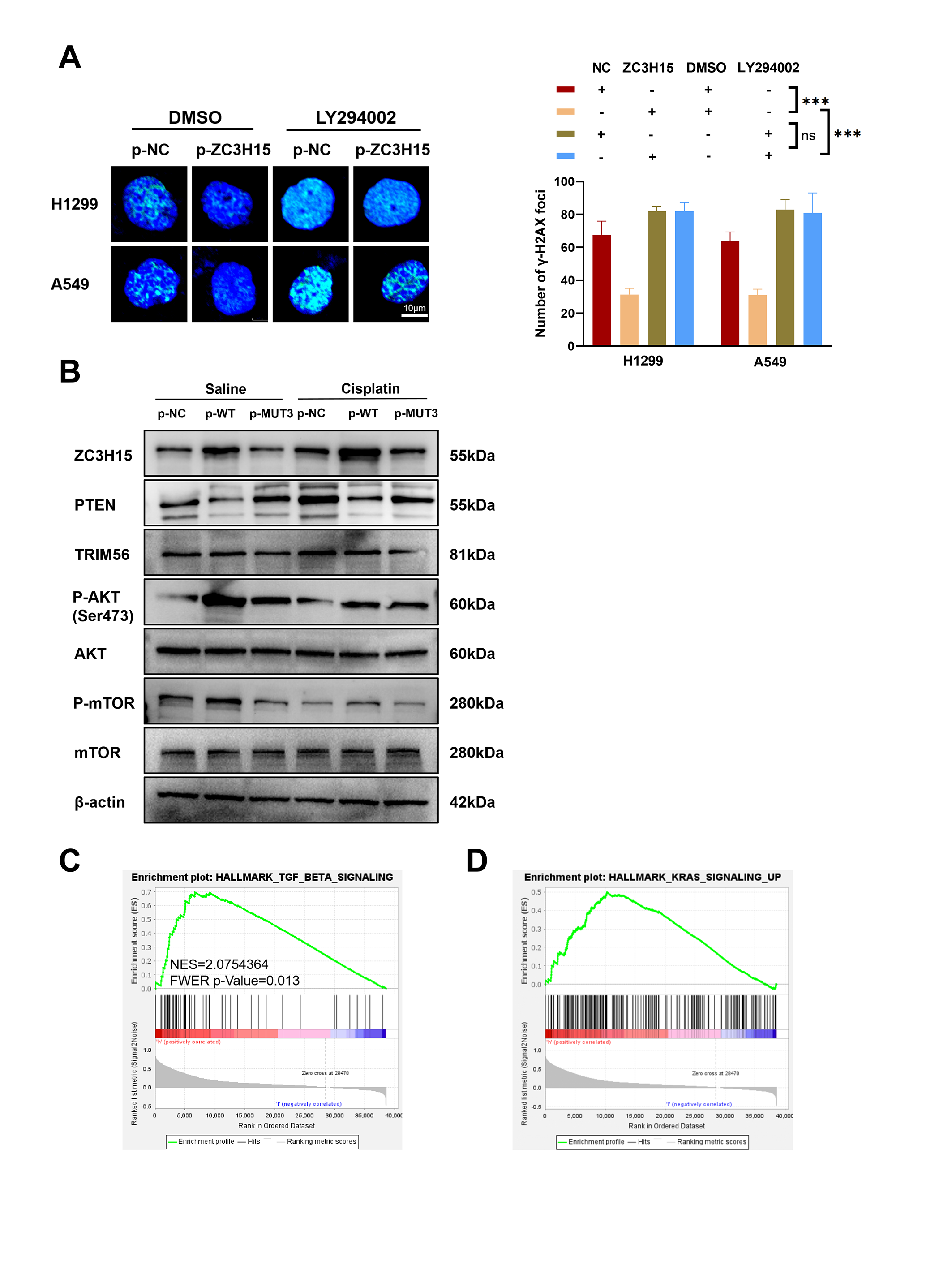

Supplement: Supplementary file 9 — Supplementary Figure9 [file 41419_2025_8138_MOESM9_ESM.png]
