# Supplementary material for: ZC3H15 regulates the ubiquitination of PTEN via recruitment of TRIM56 and promotes malignant progression of non-small cell lung cancer
Source: Cell Death Dis. 2026 Jan 9;17(1):17. doi: 10.1038/s41419-025-08138-2 (PMC12789496; doi:10.1038/s41419-025-08138-2)
Supplement: Supplementary file 10 — clean Additional files [file 41419_2025_8138_MOESM10_ESM.docx]

Supplementary Fig.1

A 43 independent independent prognostic factors were obtained by uniCOX analysis .

B-C Survival analysis of ZC3H15 in TCGA database.

D-F Survival analysis of E2F7,KIF18A and CDC25C from the Kaplan-Meier Plotter.

G-H Analysis of the expression of ZC3H15 mRNA in lung samples from the TCGA databases.

I-J Correlation analysis between ZC3H15 and tumor size and lymph node metastasis.

Supplementary Fig.2

A GSEA in the TCGA database was performed.ZC3H15-related enrichment plots were shown.

B DNA replication of H1299 cells was determined by EDU staining.Scale bar:20μm.Mean±SD,n=3.****P* < 0.001.

C Analysis of cell proliferation ability by the xenograft tumor model in nude mice.

D Cell migration was evaluated by the wound-healing assay.Mean±SD,n=3.****P* < 0.001.

E Expression of cell proliferation- and migration-related proteins in A549 and H1299.

Supplementary Fig.3

A Cell viability was analyzed by CCK8.Mean±SD,n=3.****P* < 0.001.

B Cell growth was determined by colony formation.Mean±SD,n=3.****P* < 0.001.

1. D Cell migration(C) and invasion(D) was evaluated by the Transwell migration assay.Mean±SD,n=3.****P* < 0.001.

E The xenograft tumor model in nude mice established by A549 cells with ZC3H15 knockdown. ****P* < 0.001.

F Cell migration was evaluated by the wound-healing assay.Mean±SD,n=3.****P* < 0.001.

G Expression of cell proliferation- and migration-related proteins in A549 and H1299.

Supplementary Fig.4

A-B Western blotting analyzing the expression of proteins involved in the AKT-mTOR pathway in A549 and H1299 cells with ZC3H15 overexpression or knockdown.

C Cell invasion was evaluated by the Transwell migration assay.Mean±SD,n=3.***P*<0.01,****P* < 0.001.

D Western blotting analyzing the expression of proliferation- and migration-related proteins in A549 and H1299 cells treated with DMSO or the AKT pathway inhibitor LY294002.

E Cell growth was determined by colony formation.Mean±SD,n=3. ***P*<0.01,****P* < 0.001.

F DNA replication of H1299 cells treated with DMSO or the AKT pathway inhibitor LY294002 was determined by EDU staining.Scale bar:200μm.Mean±SD,n=3. **P*<0.05, ***P*<0.01, ****P* < 0.001.

Supplementary Fig.5

A DNA replication of A549 cells treated with DMSO or the AKT pathway inhibitor LY294002 was determined by EDU staining.Scale bar:200μm.Mean±SD,n=3. **P*<0.05, ***P*<0.01, ****P* < 0.001.

1. C Cell migration and invasion was evaluated by the Transwell migration assay.Mean±SD,n=3. ***P*<0.01, ***P < 0.001.

D Expression of cell proliferation- and migration-related proteins in A549 and H1299.

Supplementary Fig.6

A Cell growth of H1299 treated with DMSO or VO-Ohpic was determined by colony formation.Mean±SD,n=3.****P* < 0.001.

B DNA replication of A549 cells treated with DMSO or VO-Ohpic was determined by EDU staining.Scale bar:20μm.Mean±SD,n=3.****P* < 0.001.

C Cell migration and invasion was evaluated by the Transwell migration assay.Mean±SD,n=3.****P* < 0.001.

D The migration of H1299 treated with DMSO or VO-Ohpic was evaluated by the wound-healing assay.Mean±SD,n=3.****P* < 0.001.

E Western blotting analyzing the expression of proteins involved in the AKT-mTOR pathway and proliferation- and migration-related proteins in A549 and H1299 cells treated with DMSO or the PTEN inhibitor VO-Ohpic.

Supplementary Fig.7

A Cell growth of H1299 transfected with ZC3H15 cDNA or ZC3H15 MUT3 cDNA was determined by colony formation.Mean±SD,n=3.****P* < 0.001.

1. C The migration(B) and invasion(C) of H1299 transfected with ZC3H15 cDNA or ZC3H15 MUT3 cDNA was evaluated by the Transwell migration assay.Mean±SD,n=3.****P* < 0.001.

Supplementary Fig.8

1. B qRT-PCR analyses of PTEN expression levels in H1299 and A549 cells with ZC3H15 overexperssion.Mean±SD,n=3.ns, nonsignifcant.

C-D Western blotting analyzing the expression of pten and p-pten in A549 and H1299 cells with ZC3H15 overexpression and knockdown.

E The level of ZC3H15 and PTEN proteins in H1299 and A549 cells treated with increasing concentration of cycloheximide (CHX) by western blotting.

F Mass spectrometry detection showing the spectrogram of TRIM56 in H1299 cells pulled down by the ZC3H15 antibody.

Supplementary Fig.9

A γ-H2AX foci formation in H1299 cells with the AKT inhibitor LY294002 was detected by Immunofluorescence 24 h after treatment with cisplatin (15 μmol/L). Mean ± SD, n = 3. ****P* <0.001.

B Western blotting analyzing the protein expression of PTEN,TRIM56 and the proteins involved in the AKT-mTOR pathway of xenograft tumors.

C-D GSEA in the TCGA database was performed. ZC3H15-related enrichment plots were shown.
