# Supplementary material for: ZC3H15 regulates the ubiquitination of PTEN via recruitment of TRIM56 and promotes malignant progression of non-small cell lung cancer
Source: Cell Death Dis. 2026 Jan 9;17(1):17. doi: 10.1038/s41419-025-08138-2 (PMC12789496; doi:10.1038/s41419-025-08138-2)
Supplement: Supplementary file 11 — Supplementary Table 1 [file 41419_2025_8138_MOESM11_ESM.docx]

| **Accession** | **Gene Name** | **Mass** | **Score** | **Matches** | **Sequences** | **emPAI** | **Coverage** |
| --- | --- | --- | --- | --- | --- | --- | --- |
| [sp\|Q9BRZ2\|TRI56_HUMAN](protein/sp_Q9BRZ2_TRI56_HUMAN.html) | TRIM56 | 83147 | 106 | 8(2) | 7(2) | 0.11 | 16% |
| [tr\|A0A087WTW0\|A0A087WTW0_HUMAN](protein/tr_A0A087WTW0_A0A087WTW0_HUMAN.html) | UHRF1 | 98213 | 77 | 8(2) | 5(2) | 0.07 | 8% |
| [tr\|A0A024RCP3\|A0A024RCP3_HUMAN](protein/tr_A0A024RCP3_A0A024RCP3_HUMAN.html) | TRIM26 | 62925 | 45 | 5(2) | 5(2) | 0.08 | 5% |
| [tr\|A0A6Q8PGG9\|A0A6Q8PGG9_HUMAN](protein/tr_A0A6Q8PGG9_A0A6Q8PGG9_HUMAN.html) | TRIP12 | 230380 | 70 | 5(2) | 5(2) | 0.03 | 2% |
| [tr\|A0A669KBL1\|A0A669KBL1_HUMAN](protein/tr_A0A669KBL1_A0A669KBL1_HUMAN.html) | USP7 | 133441 | 42 | 4(1) | 3(1) | 0.02 | 3% |

**Supplementary Table 1 The E3 ubiquitin ligase in ZC3H15 mass spectrometry data**

Mass: Protein molecular mass

Matches: The total number of peptide segments matched, with parentheses indicating the number of matches above the significance threshold

Sequences: The total number of sequences matched, with parentheses indicating the number of sequences above the significance threshold

emPAI: 10^(Nobserved/Nobservable)-1^

Coverage: Protein identification coverage rate
