# Supplementary material for: ZC3H15 regulates the ubiquitination of PTEN via recruitment of TRIM56 and promotes malignant progression of non-small cell lung cancer
Source: Cell Death Dis. 2026 Jan 9;17(1):17. doi: 10.1038/s41419-025-08138-2 (PMC12789496; doi:10.1038/s41419-025-08138-2)

fig1

组织对ZC3H15-1

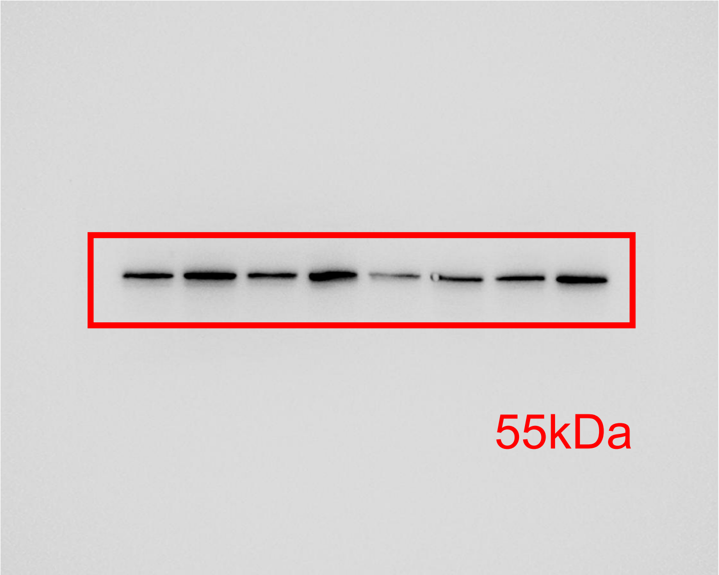

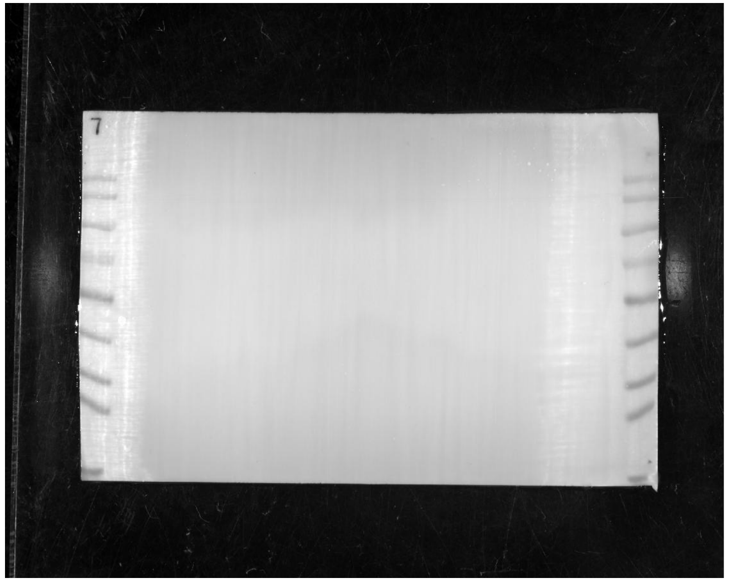

组织对ZC3H15-2

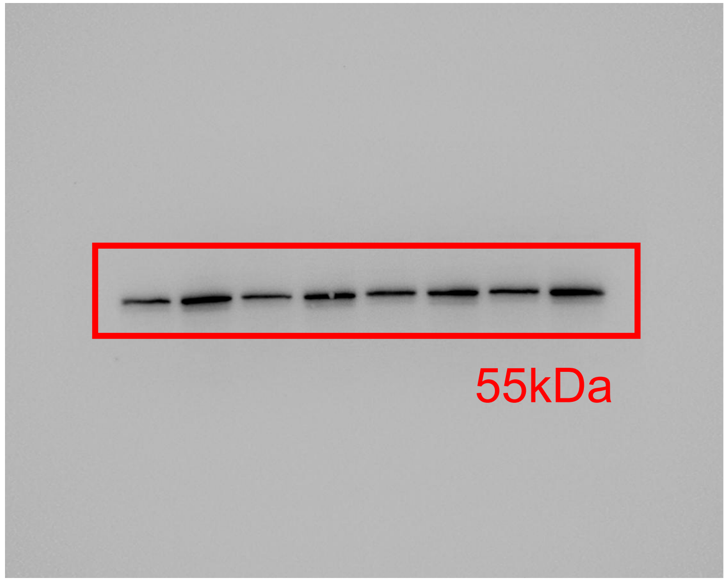

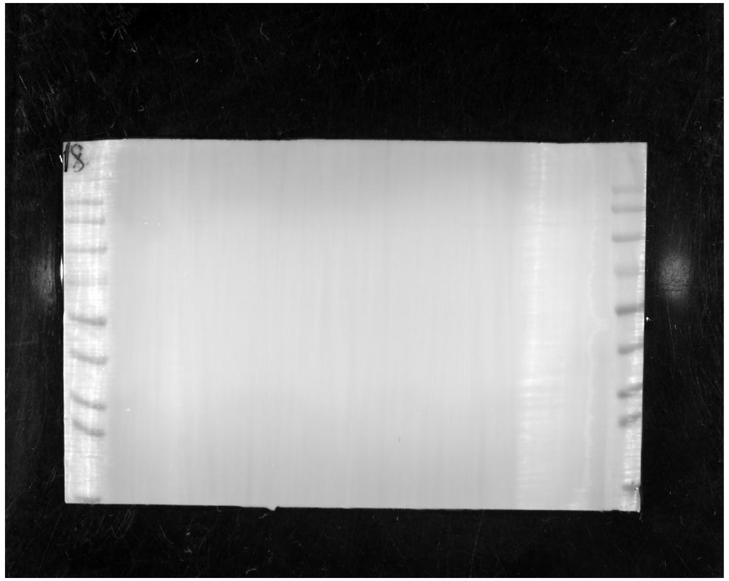

组织对gapdh-1

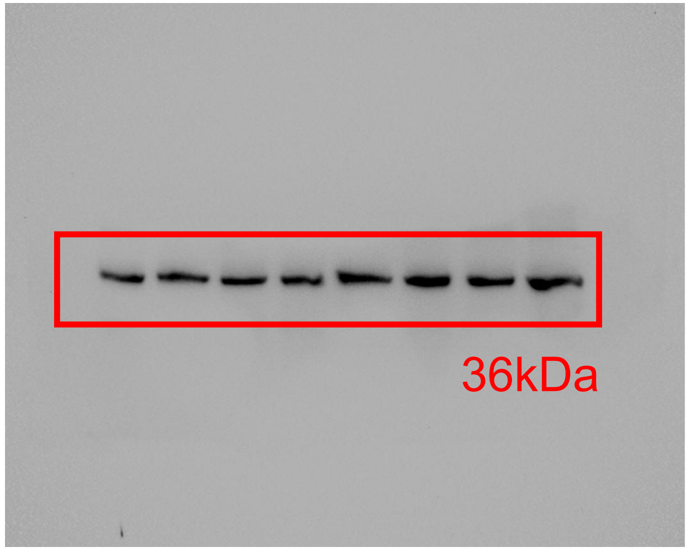

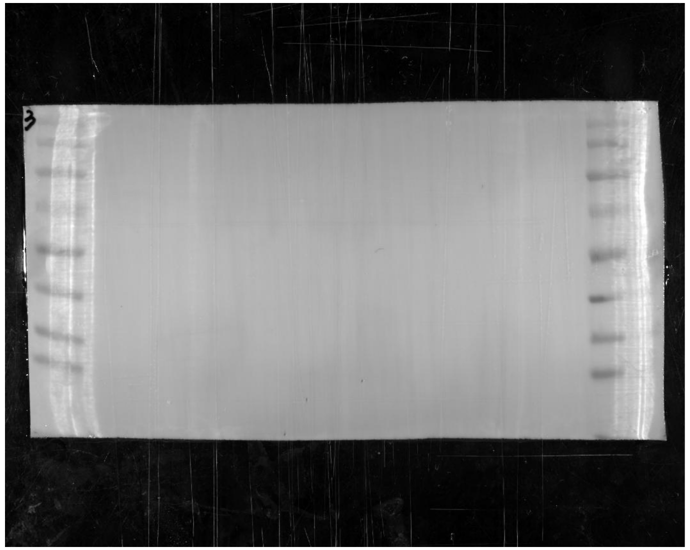

组织对gapdh-2

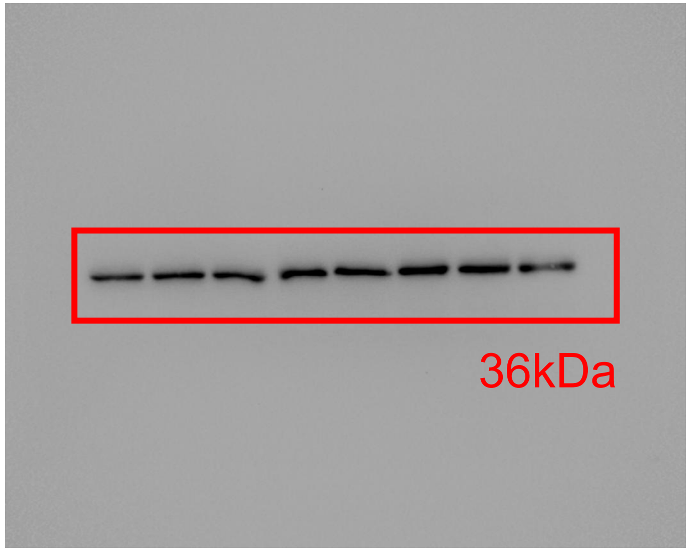

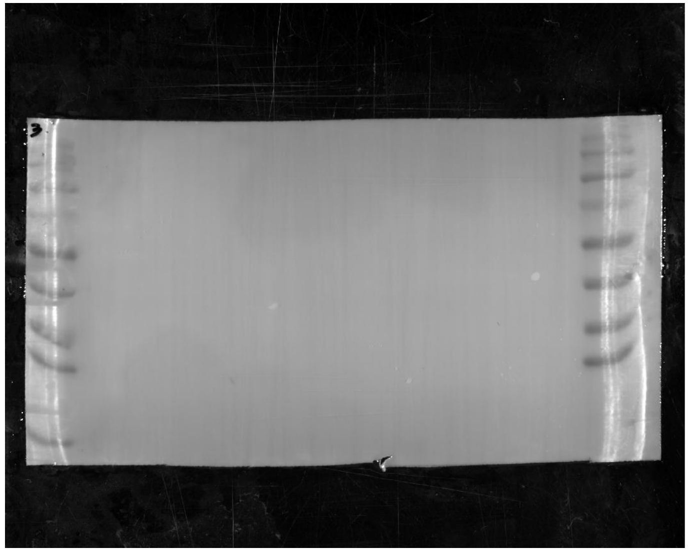

fig2

细胞系ZC3H15-1

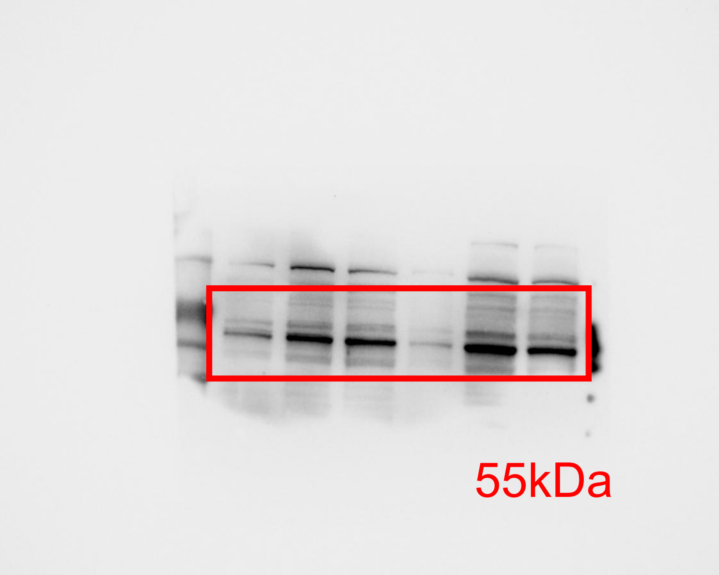

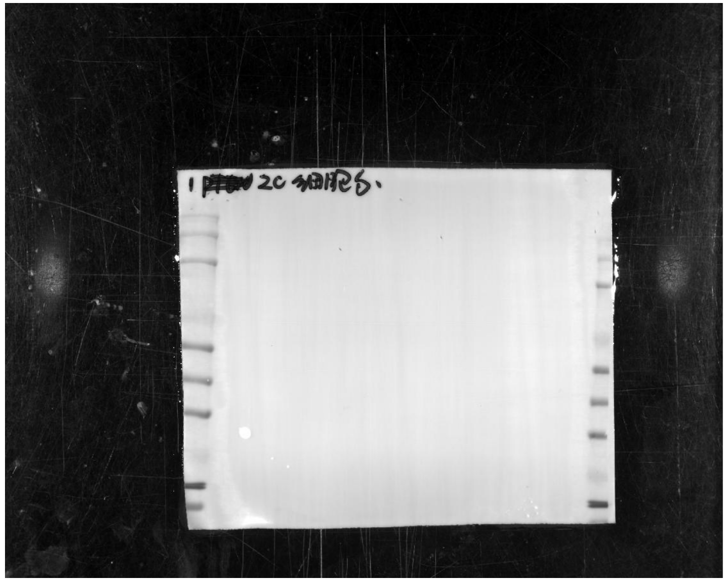

细胞系GAPDH-1

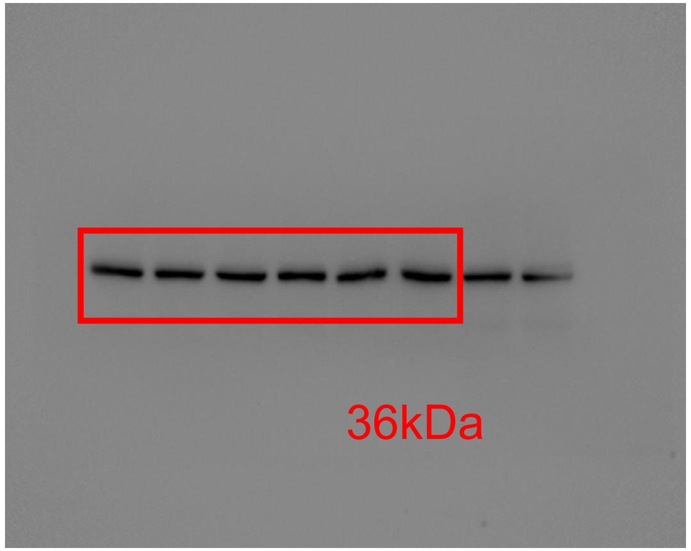

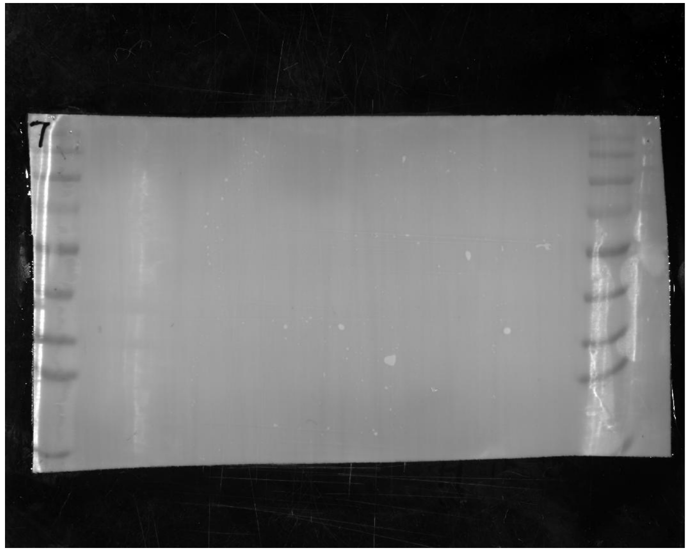

A549 ZC3H15

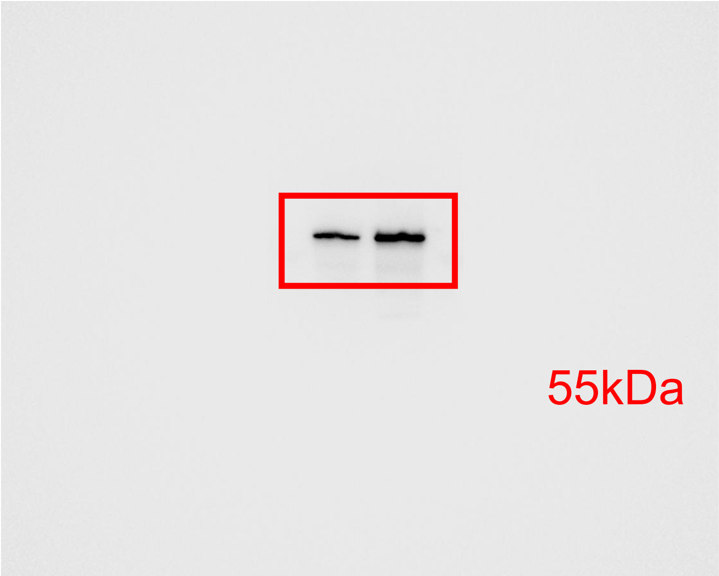

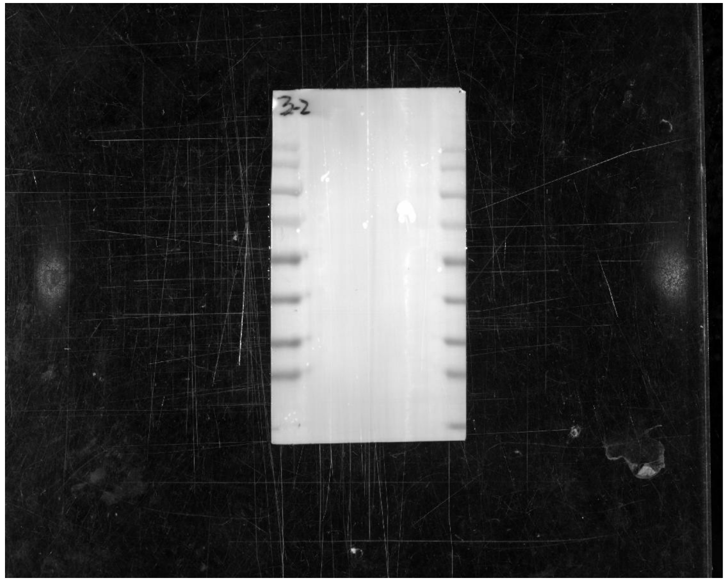

H1299 ZC3H15

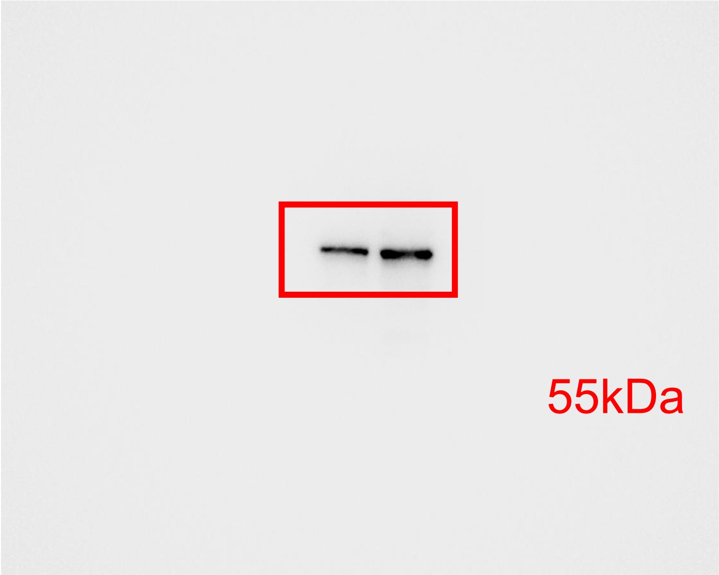

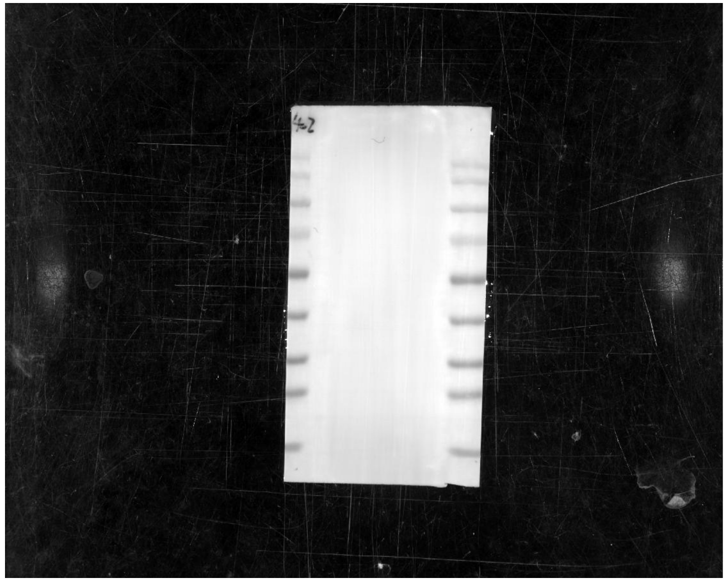

A549 β-actin

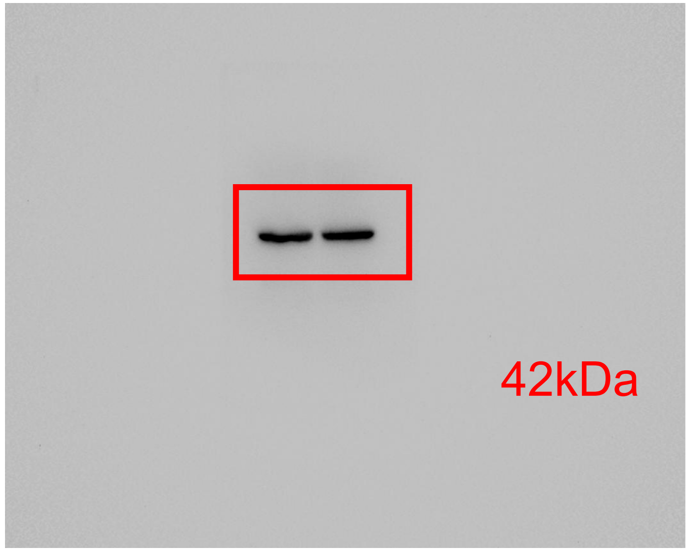

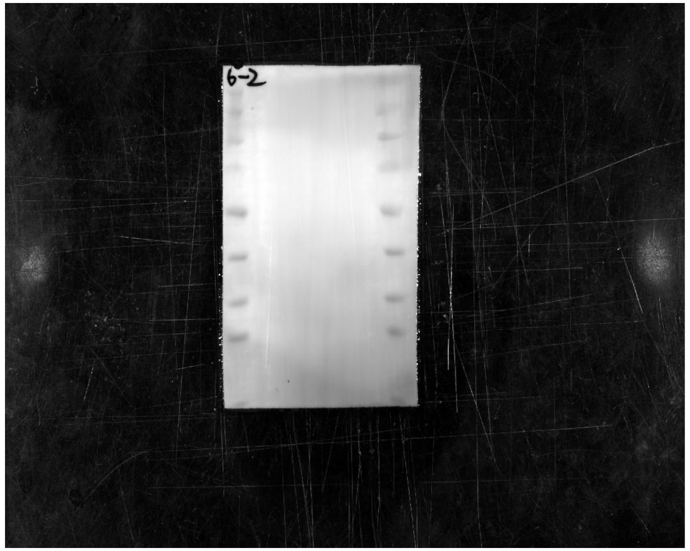

H1299 β-actin

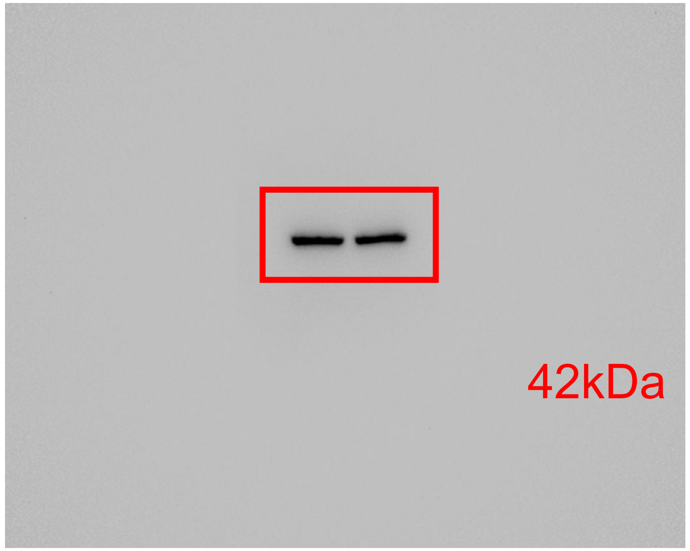

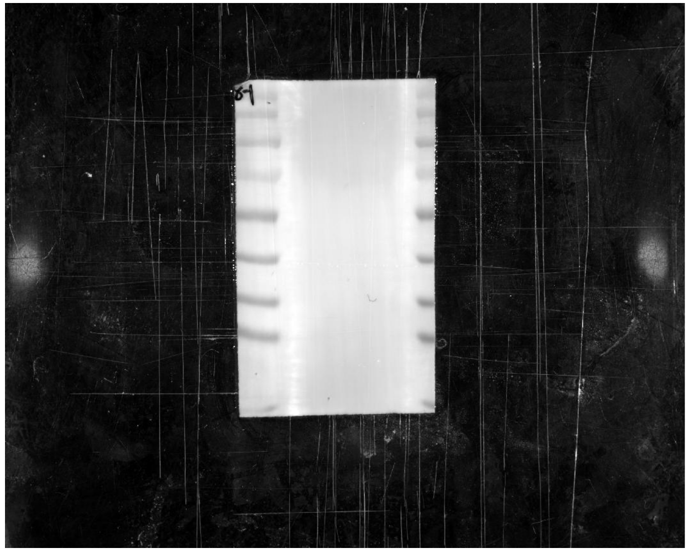

A549 ZC3H15

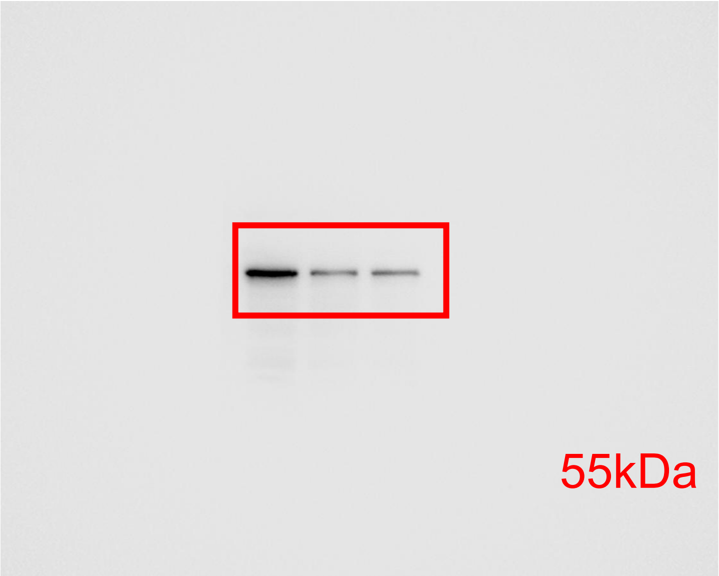

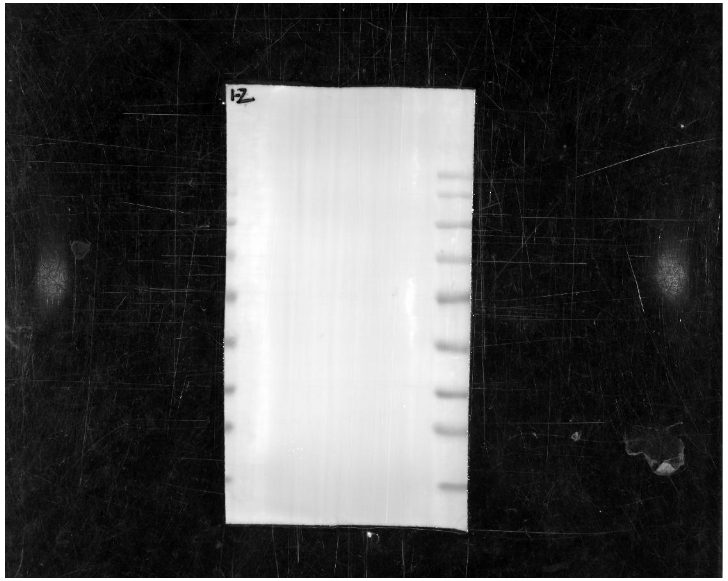

H1299 ZC3H15

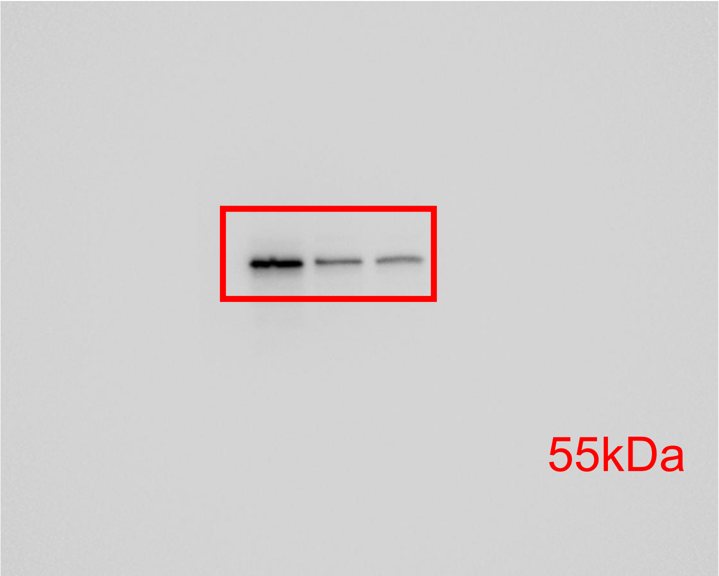

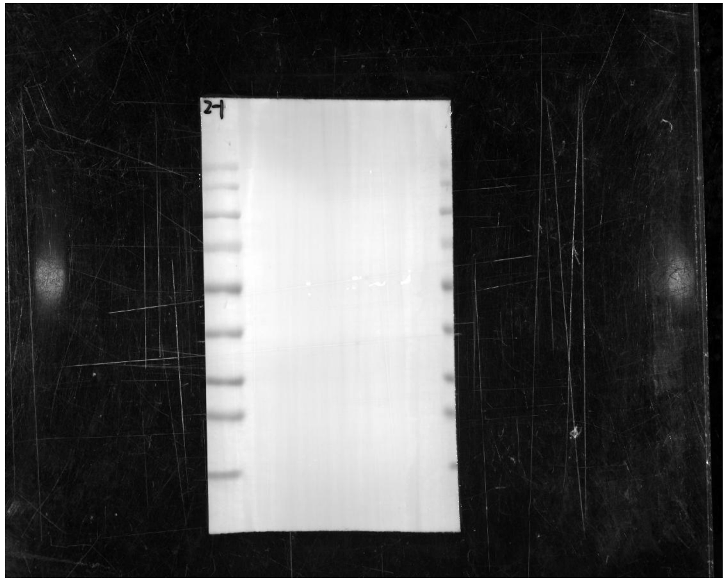

A549 β-actin

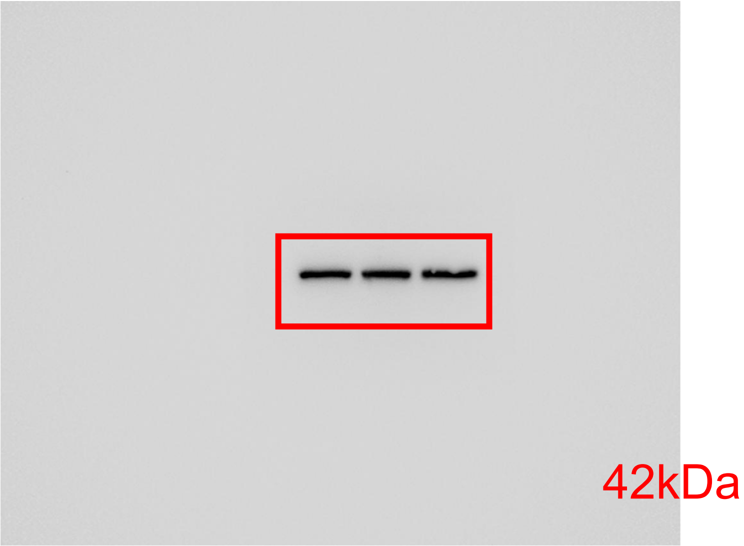

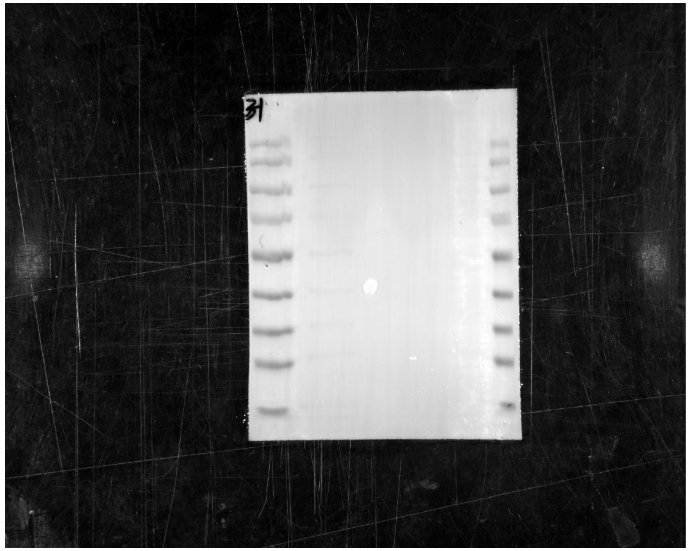

H1299 β-actin

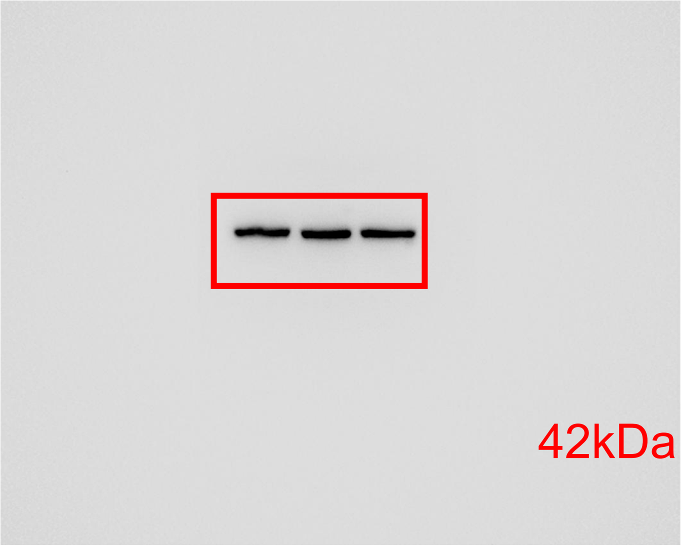

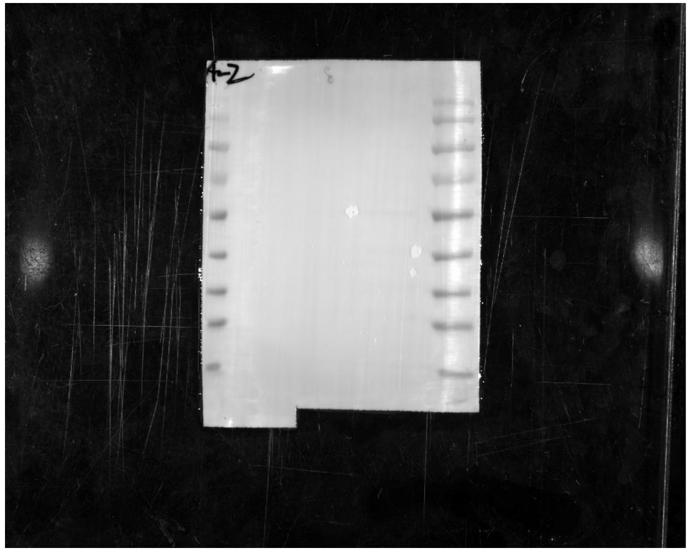

S-fig2-3

A549 OE CDK4

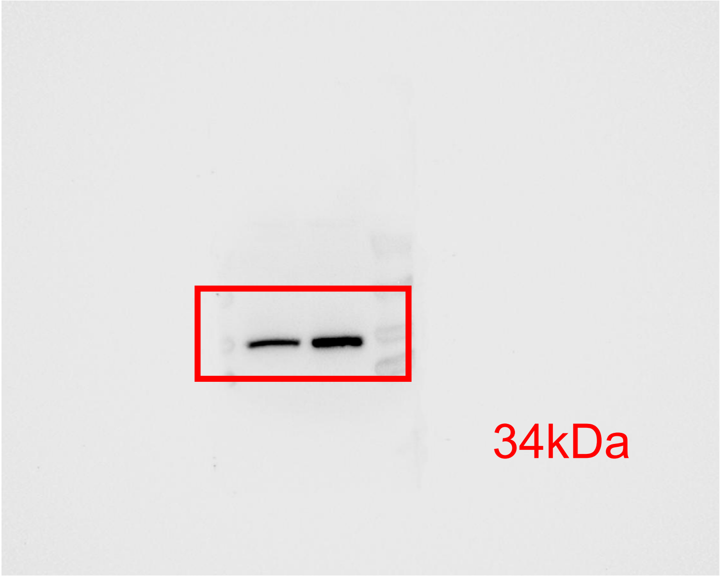

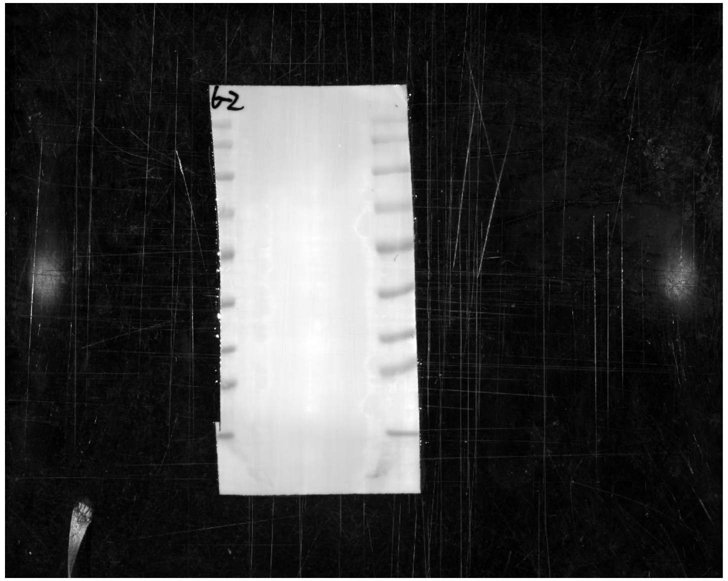

H1299 OE CDK4

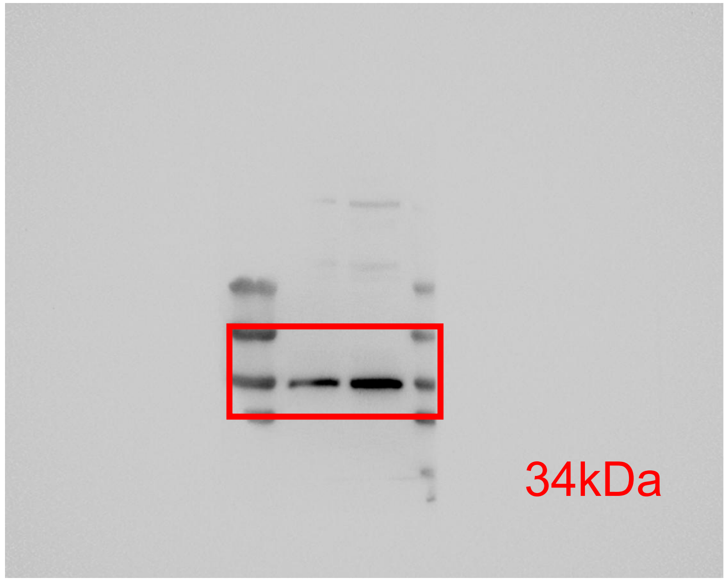

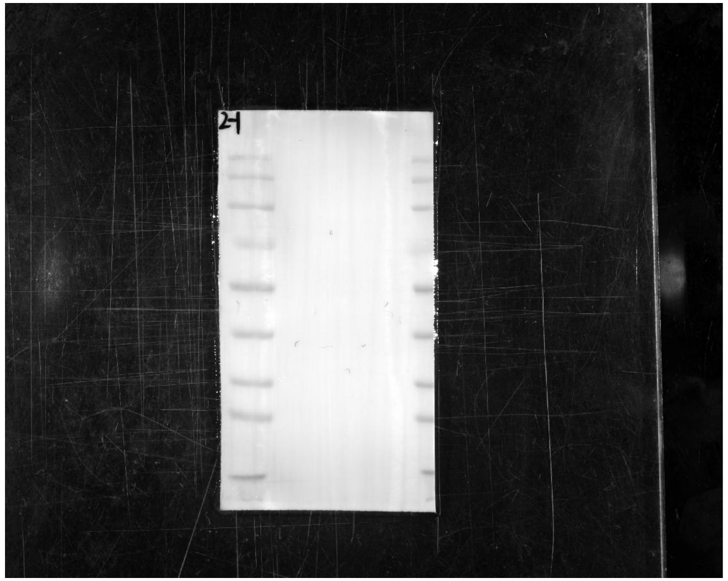

A549 SH CDK4

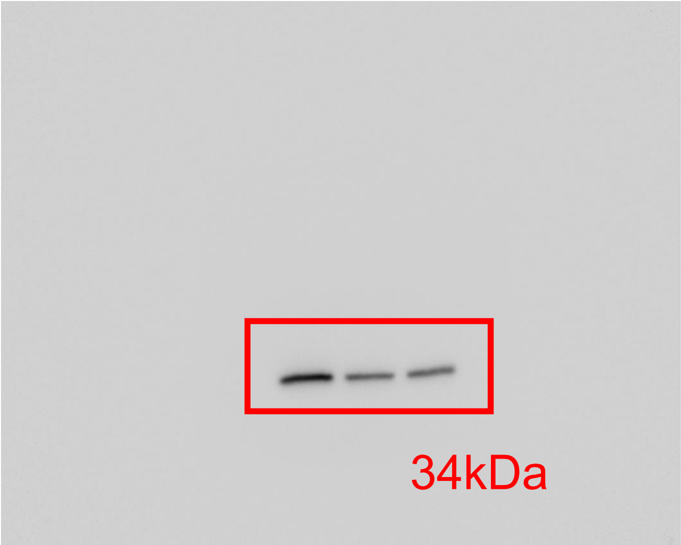

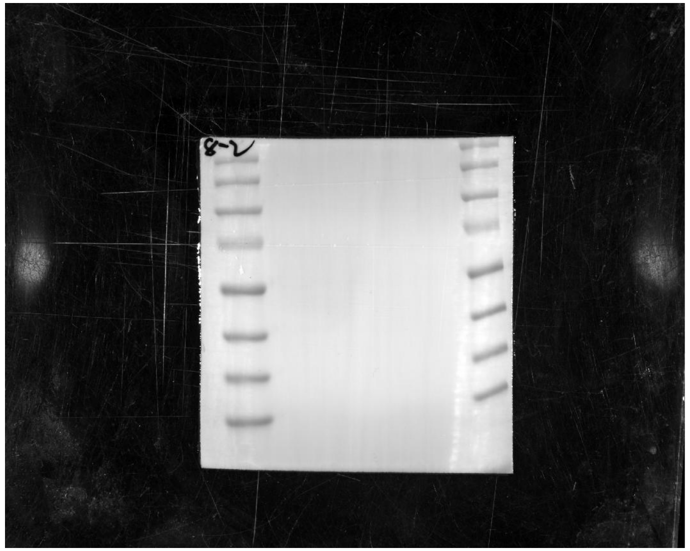

H1299 SH CDK4

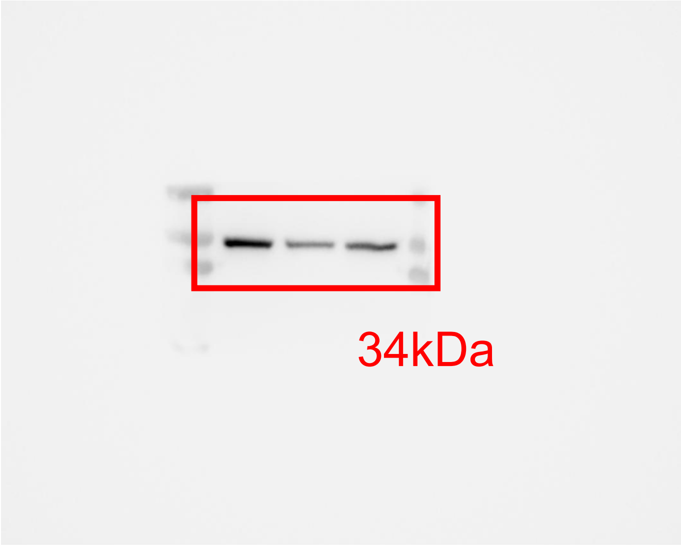

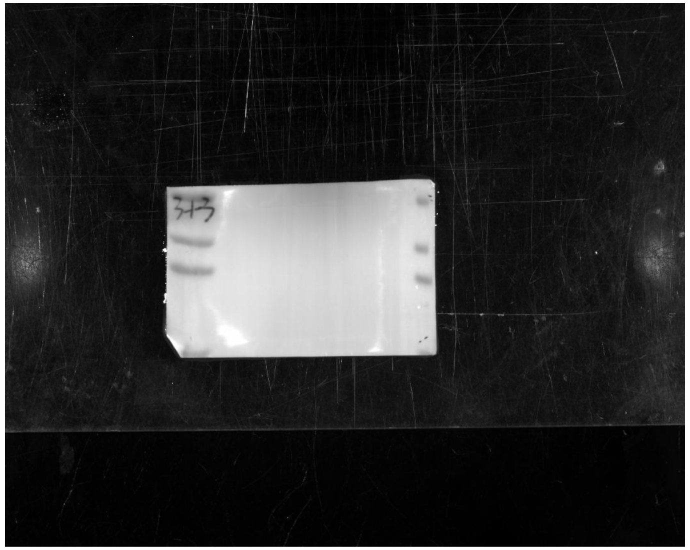

A549 OE CDK6

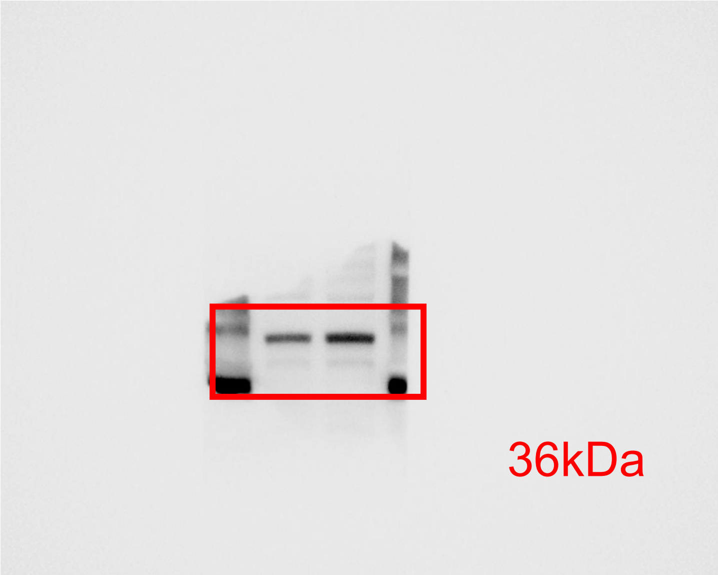

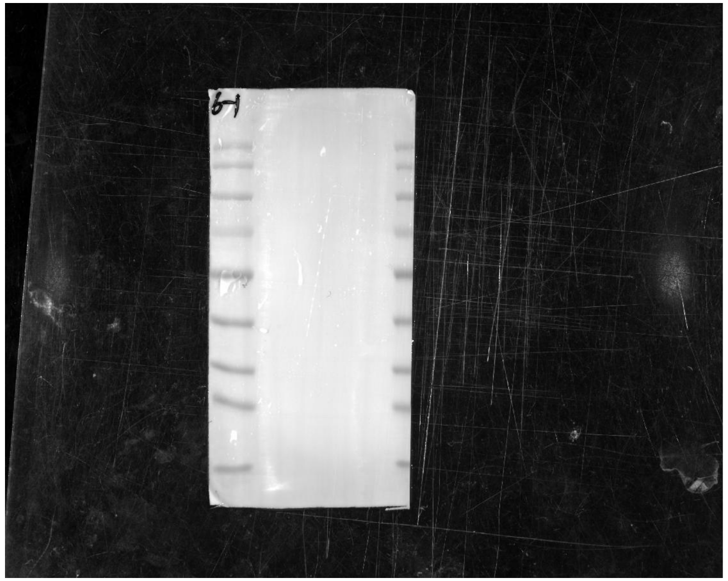

H1299 OE CDK6

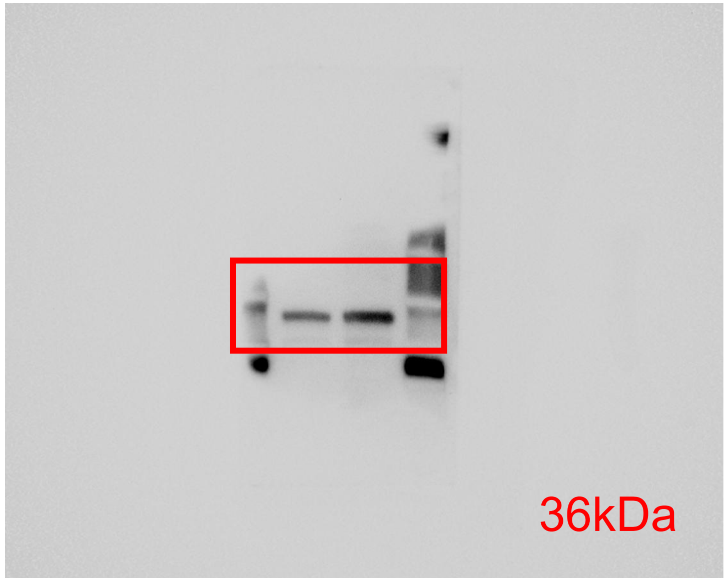

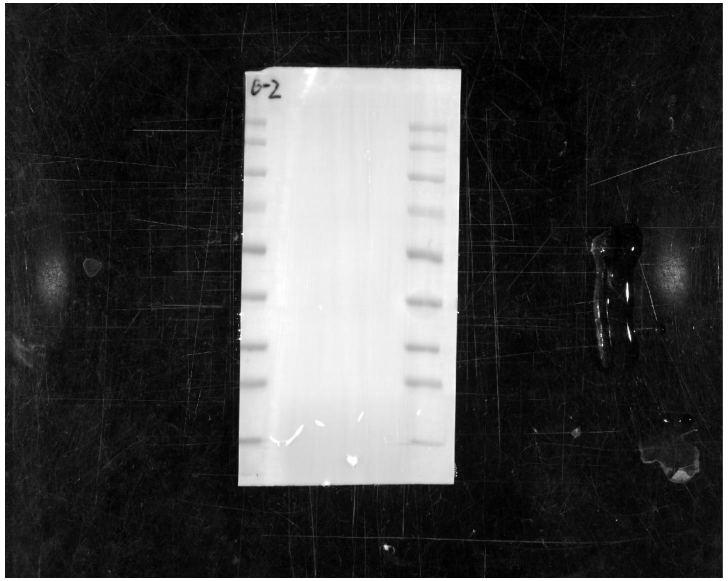

A549 SH CDK6

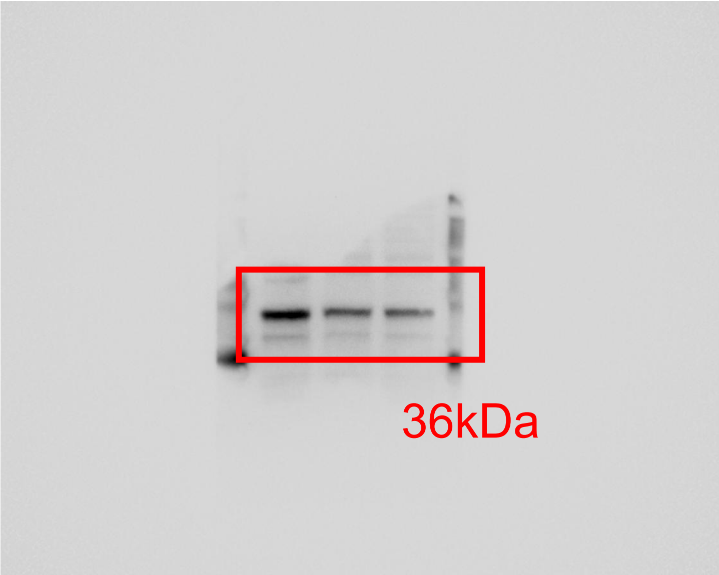

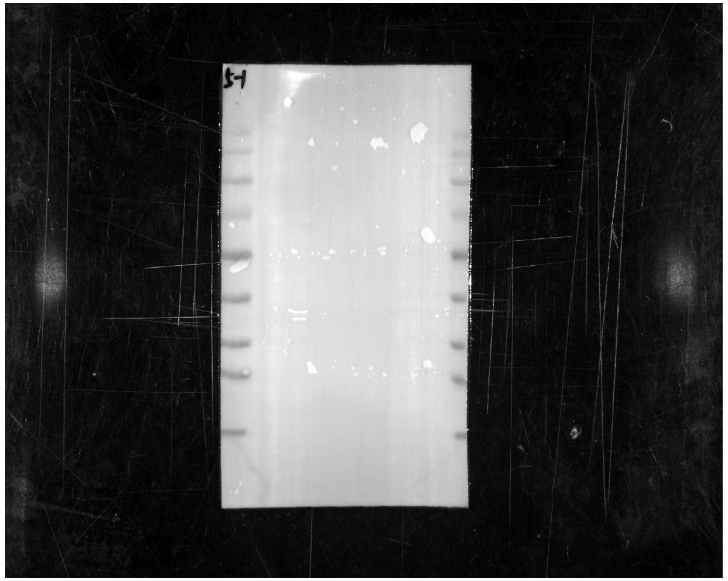

H1299 SH CDK6

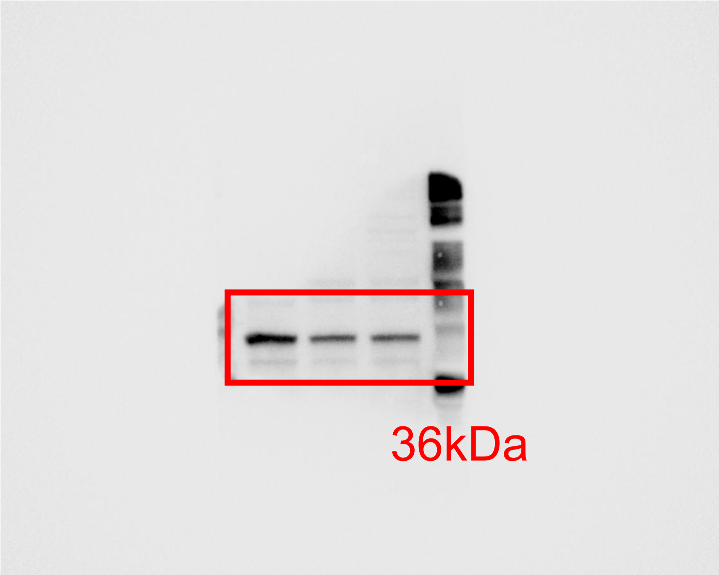

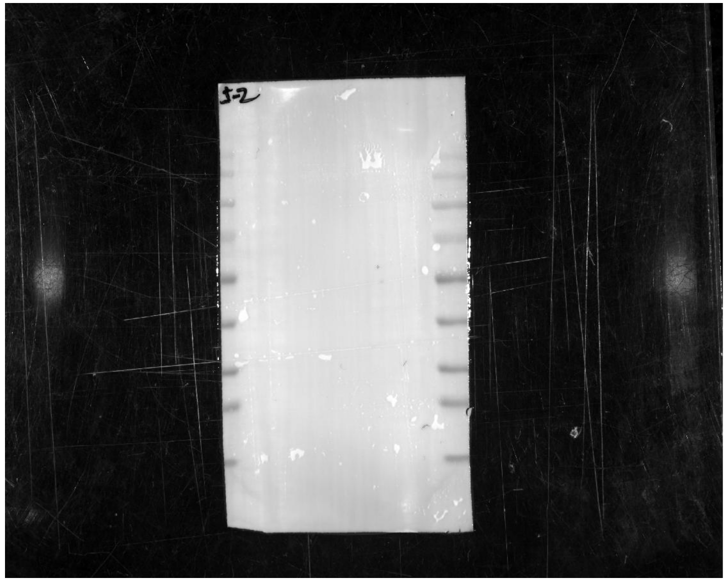

A549 OE D1

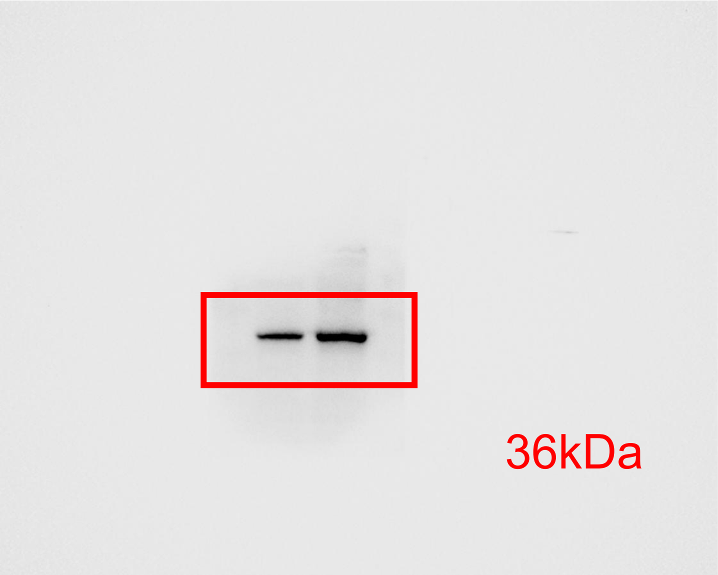

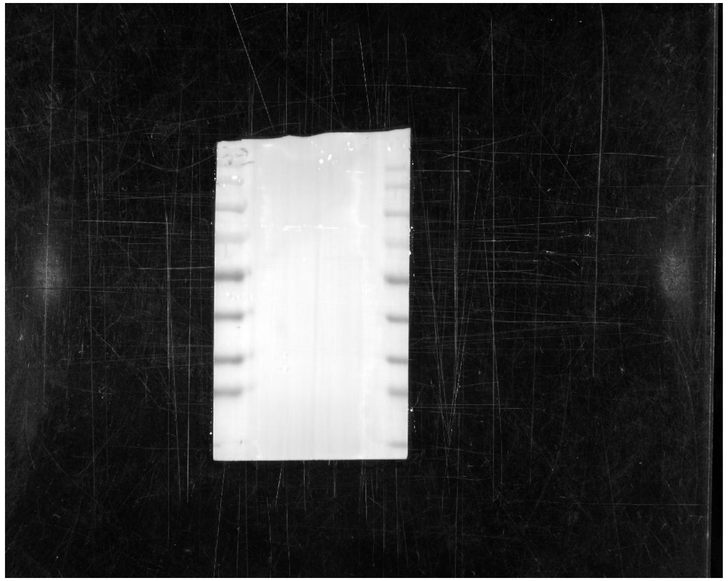

H1299 OE D1

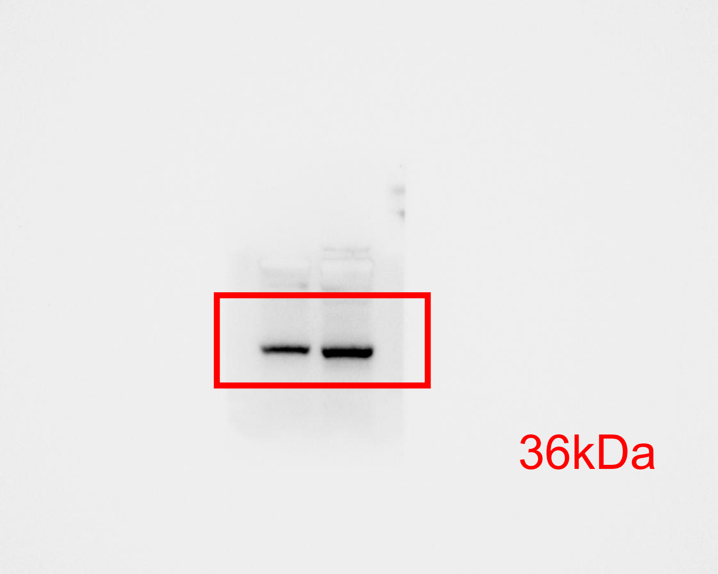

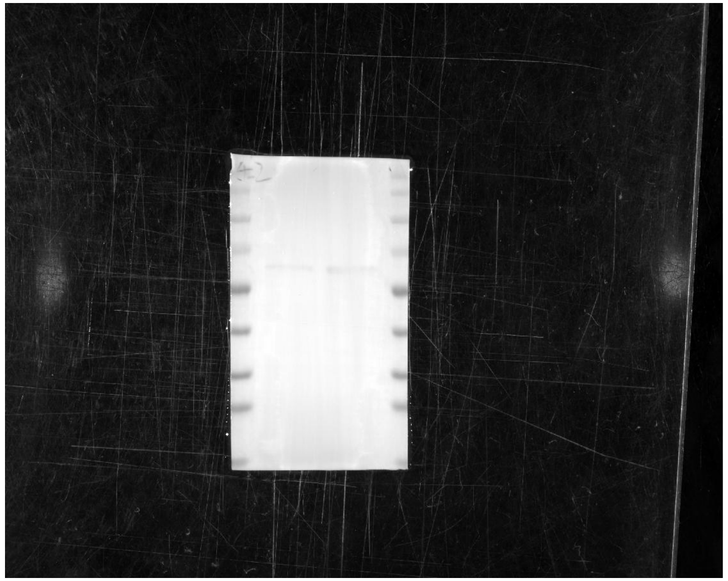

A549 SH D1

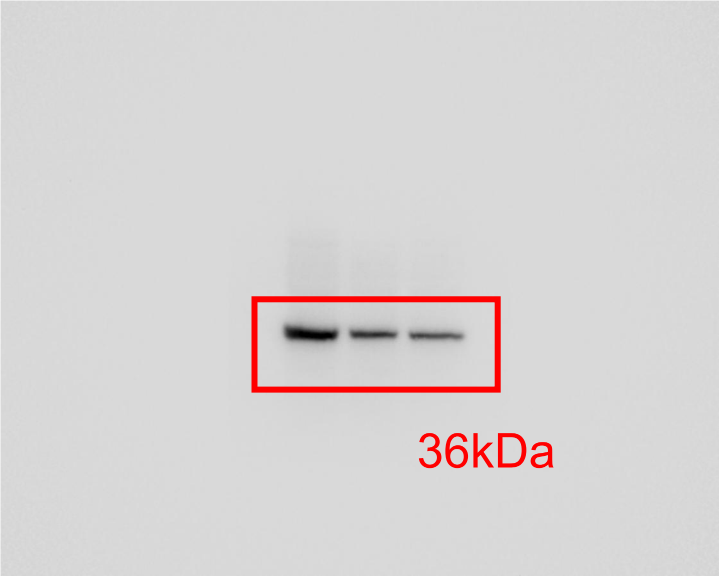

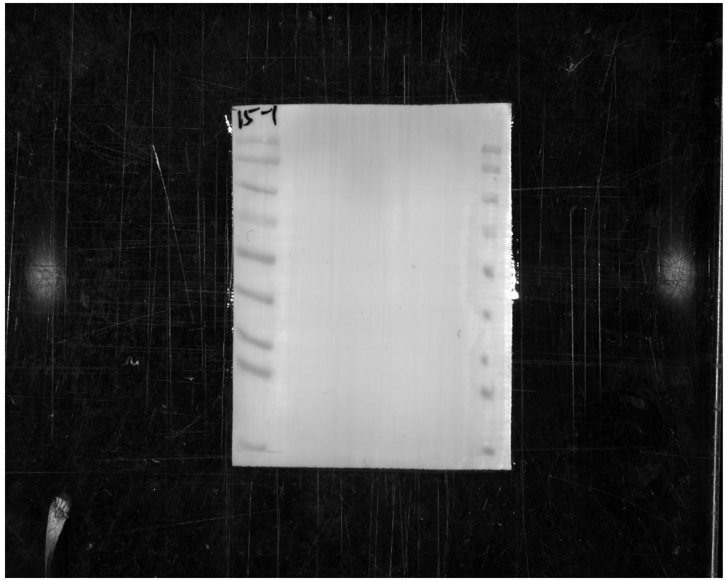

H1299 SH D1

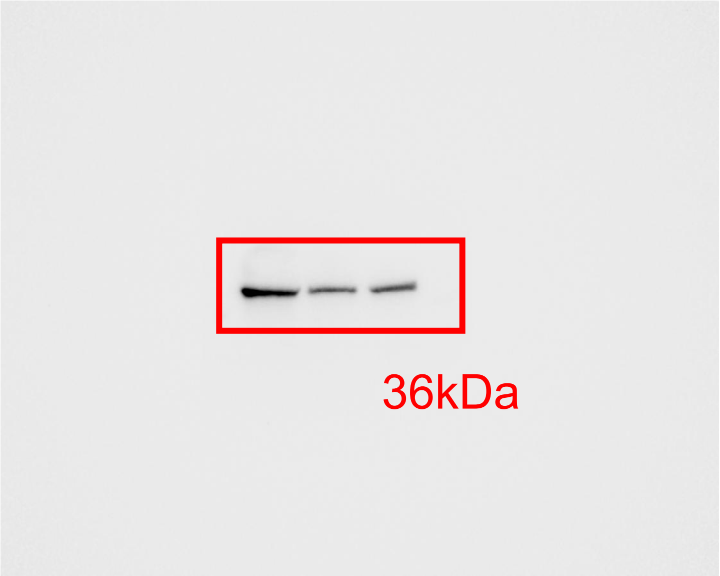

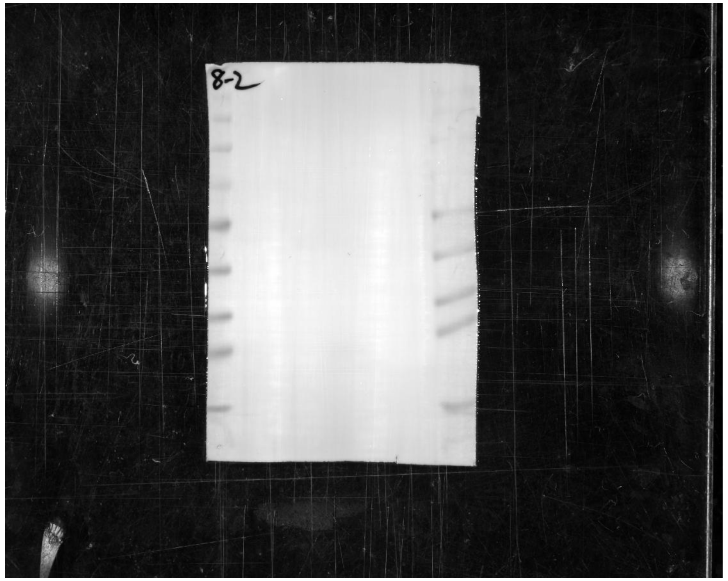

A549 OE E-ca

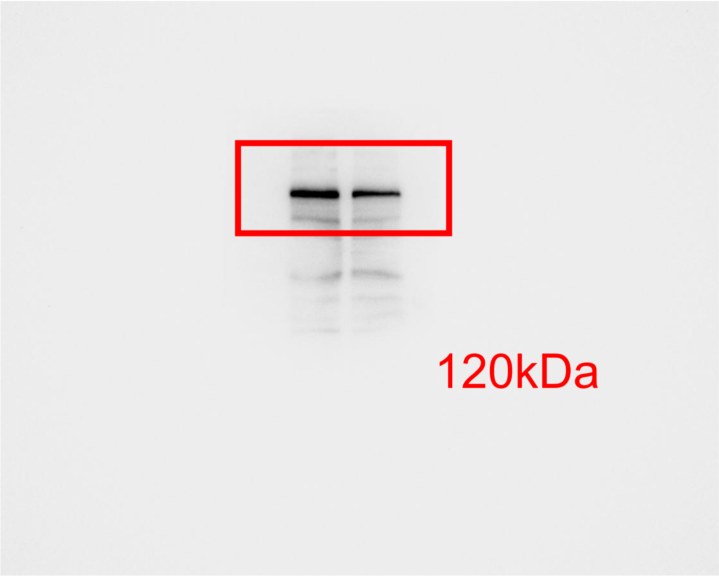

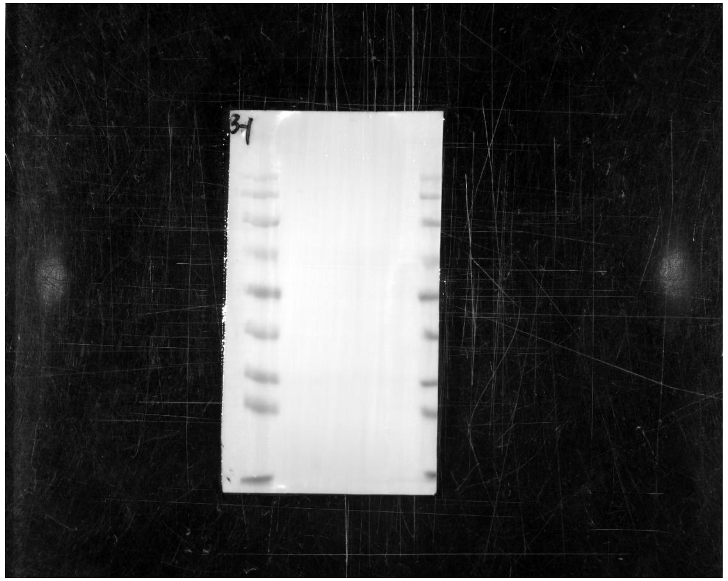

H1299 OE E-ca

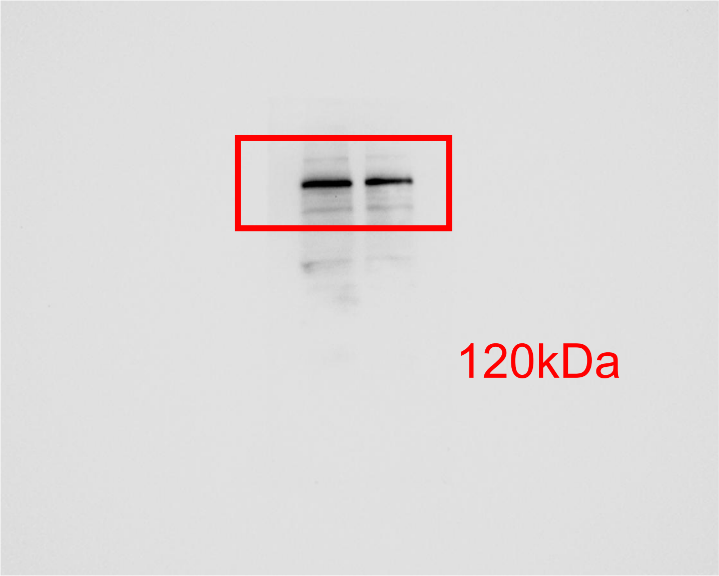

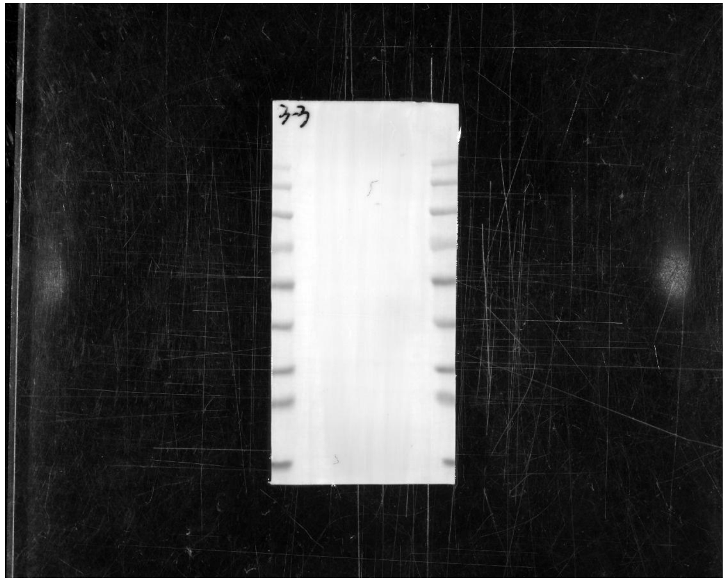

A549 SH E-ca

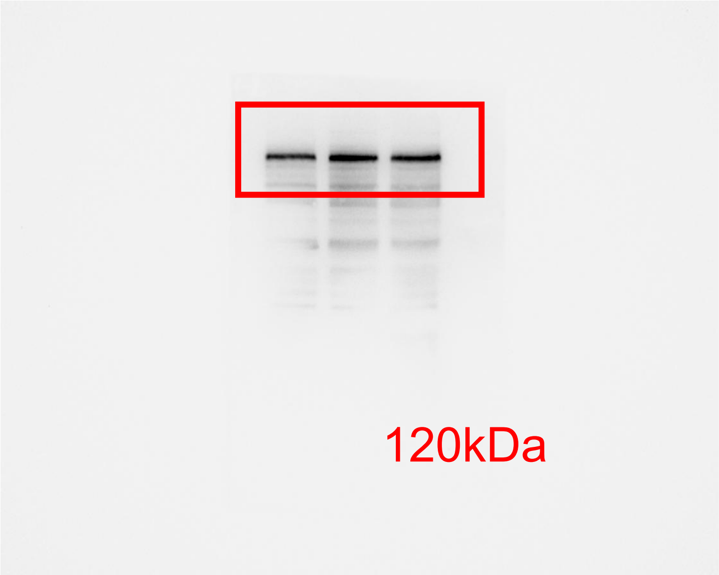

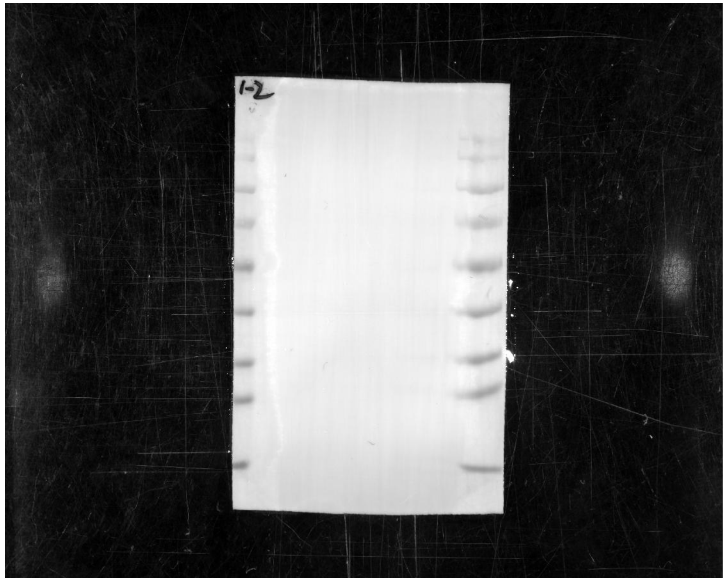

H1299 SH E-ca

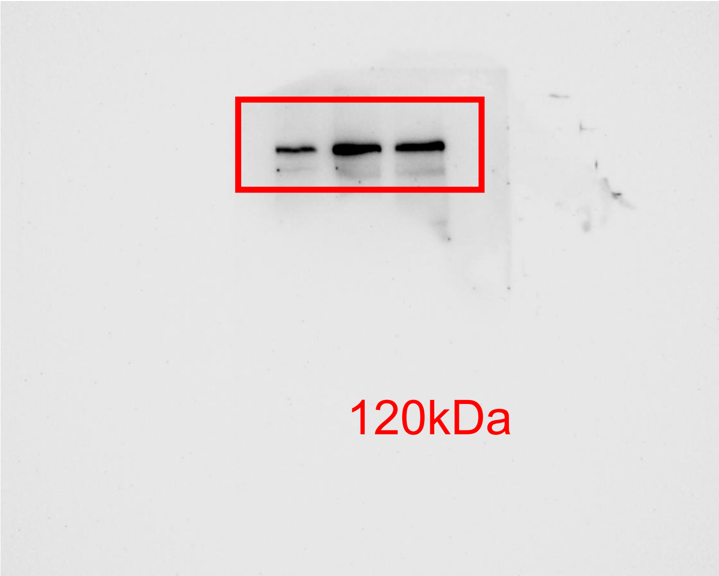

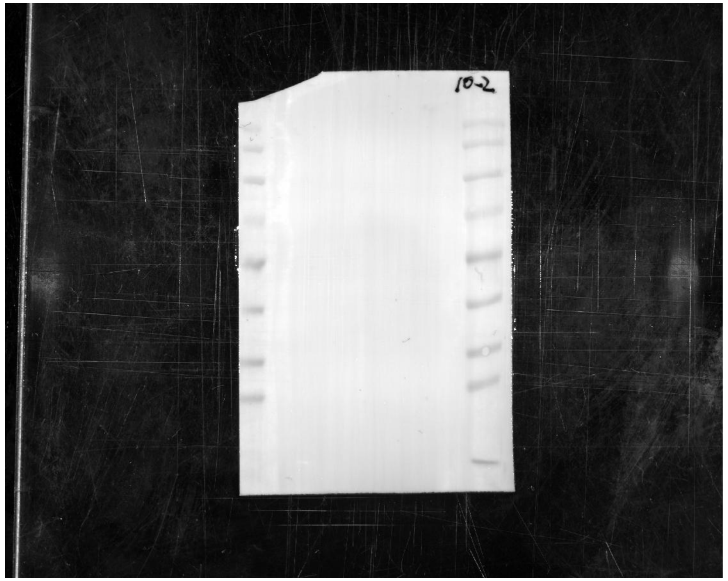

A549 OE N-ca

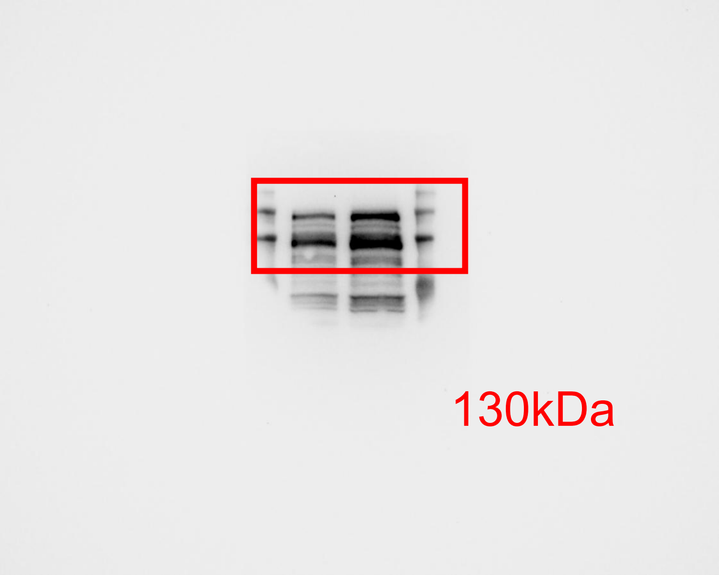

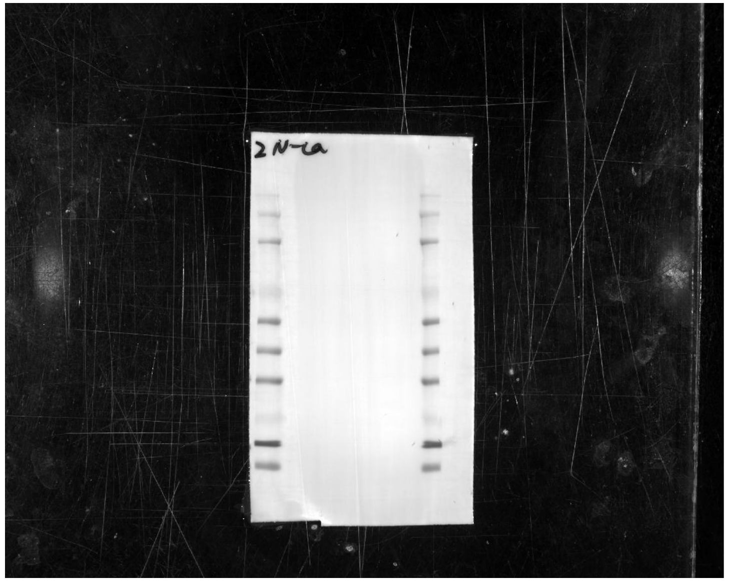

H1299 OE N-ca

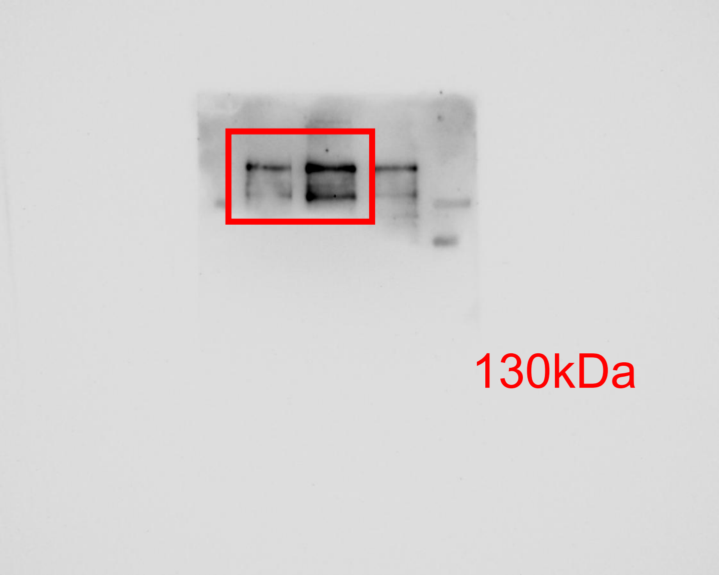

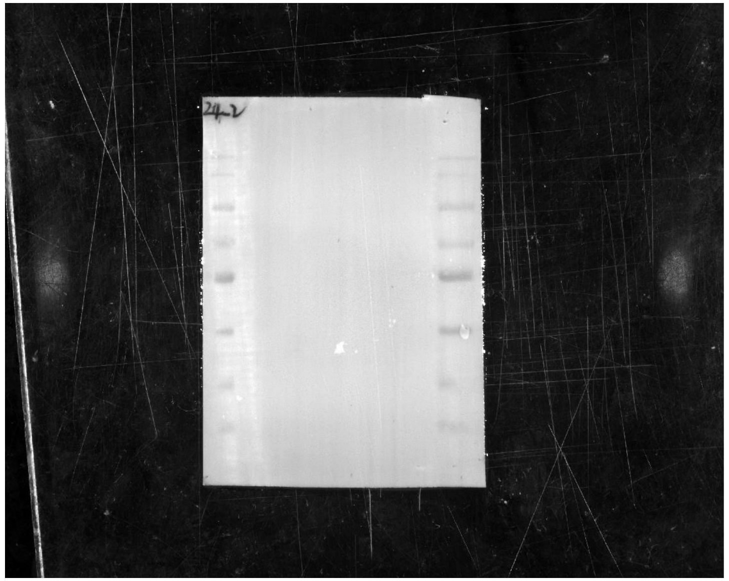

A549 SH N-ca

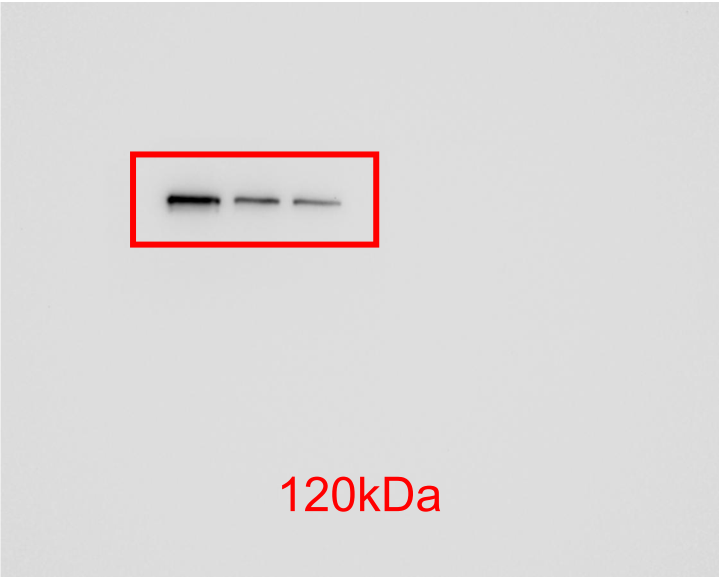

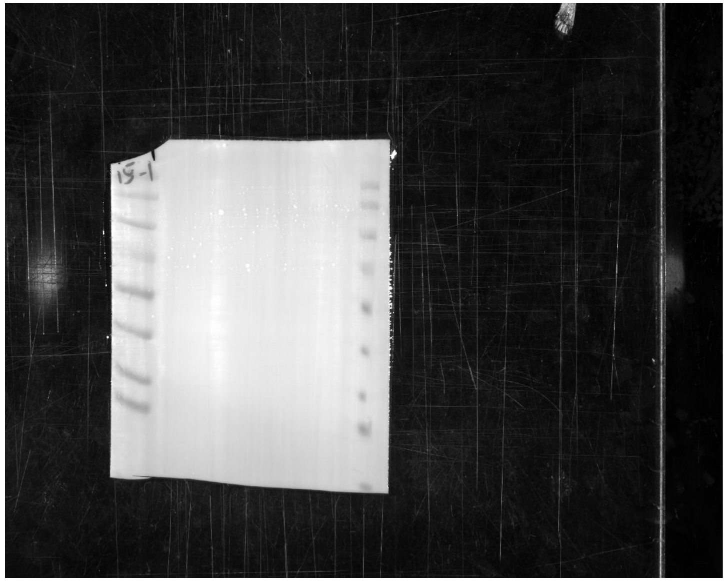

H1299 SH N-ca

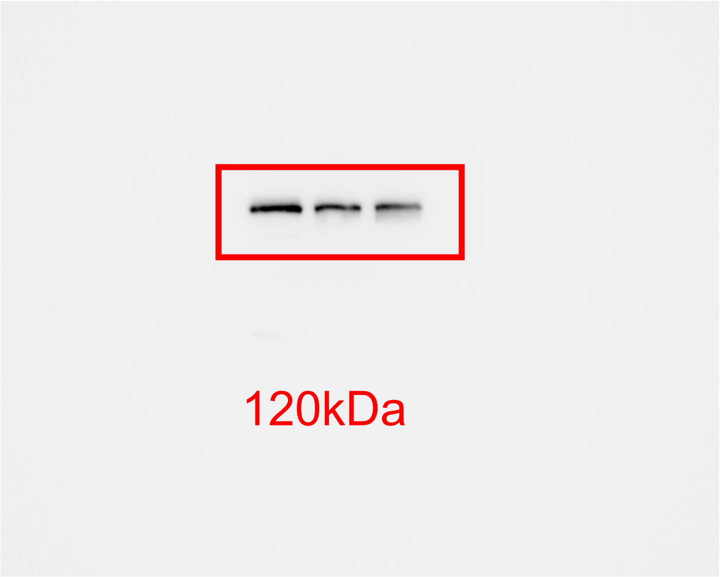

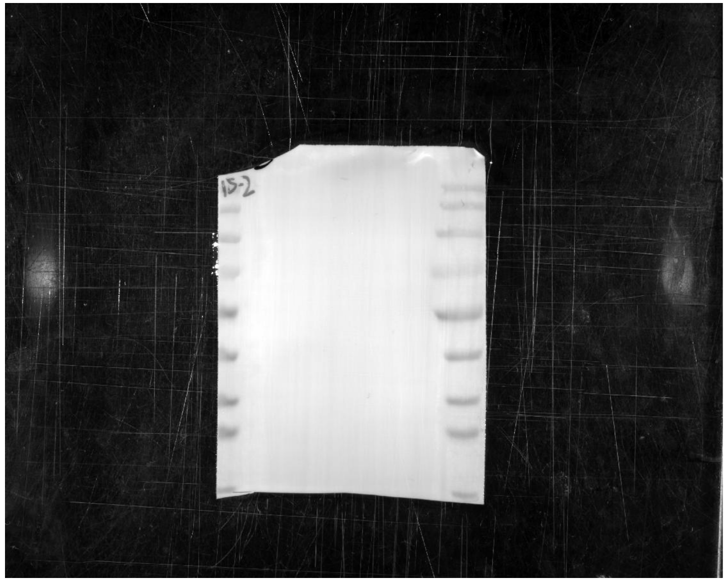

A549 OE mmp9

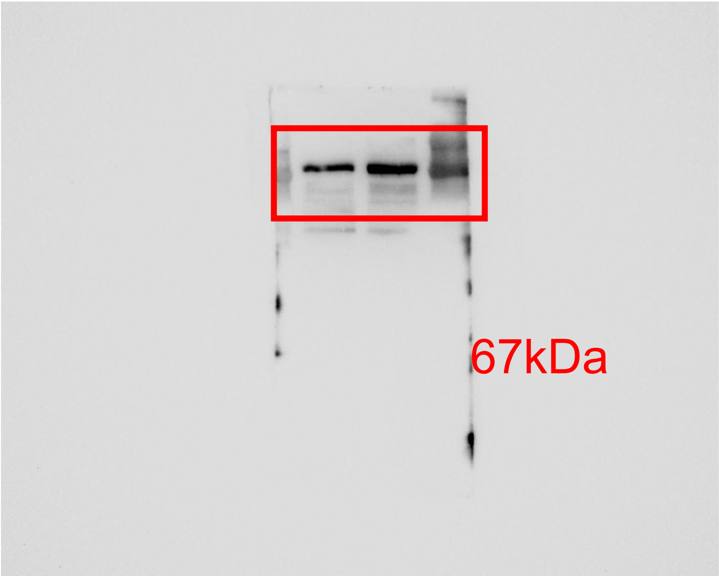

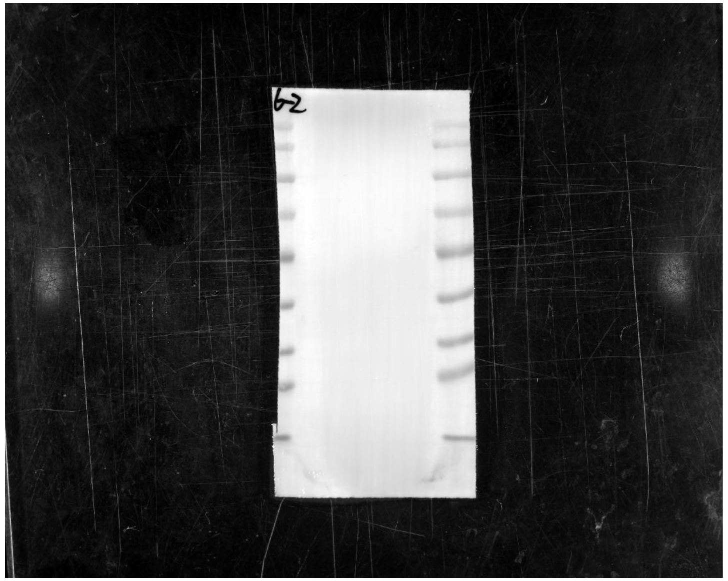

H1299 OE mmp9

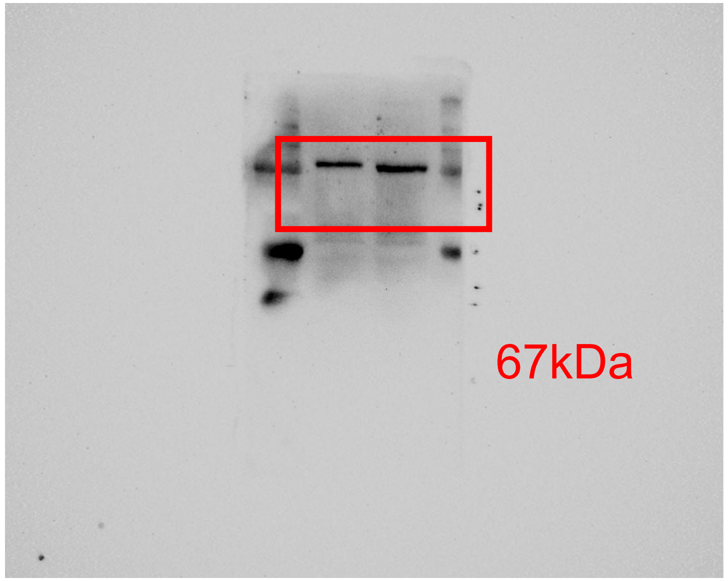

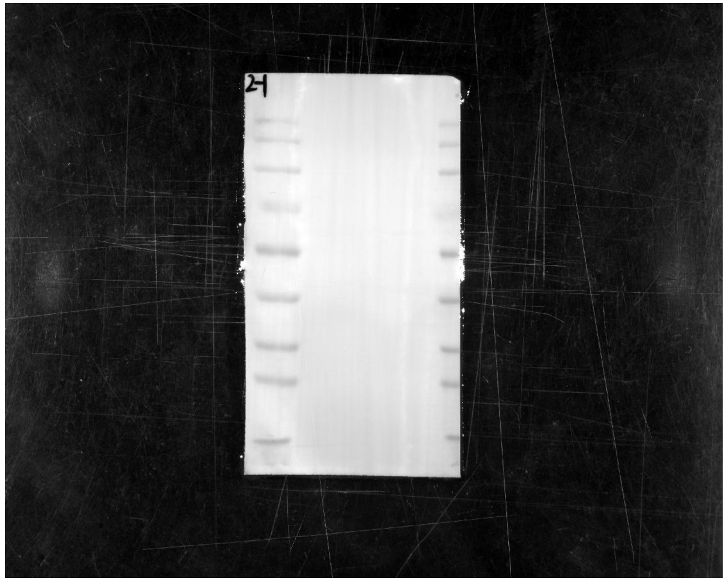

A549 SH mmp9

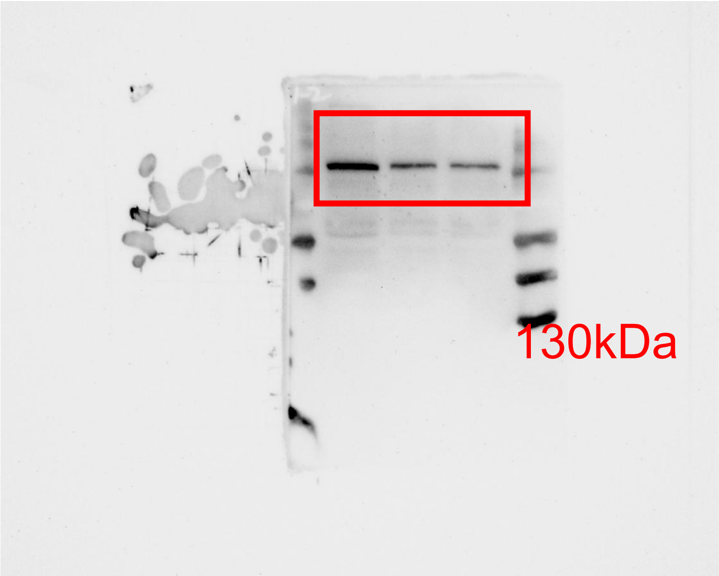

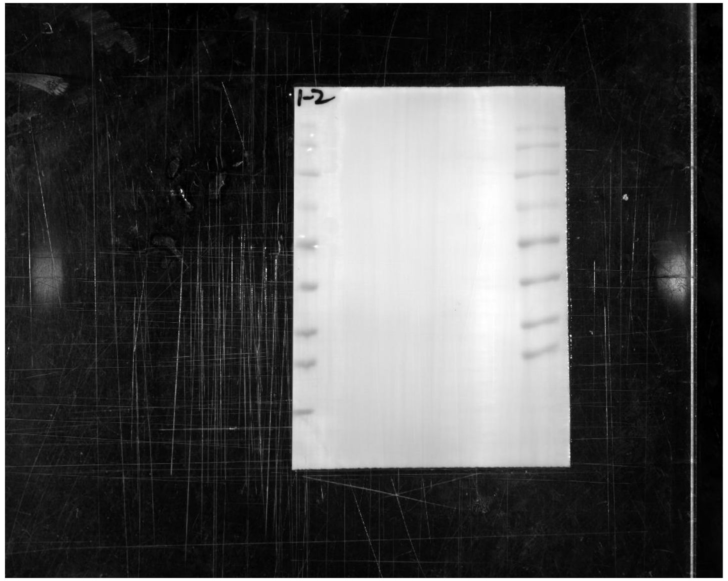

H1299 SH
mmp9

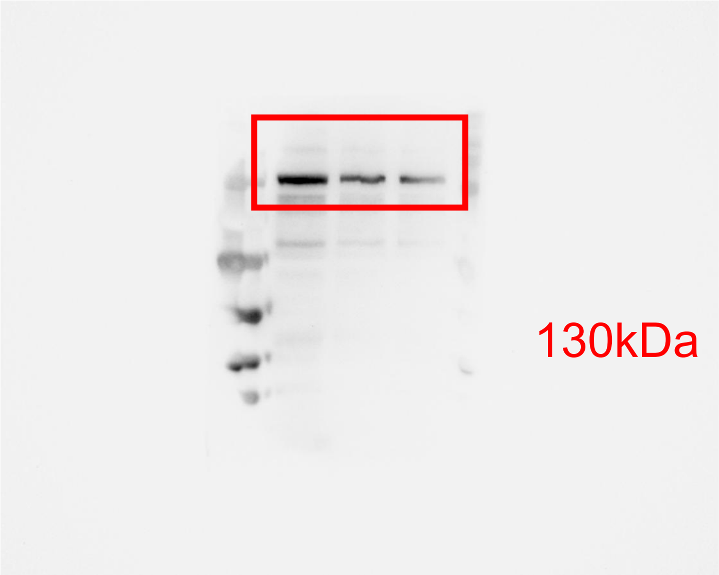

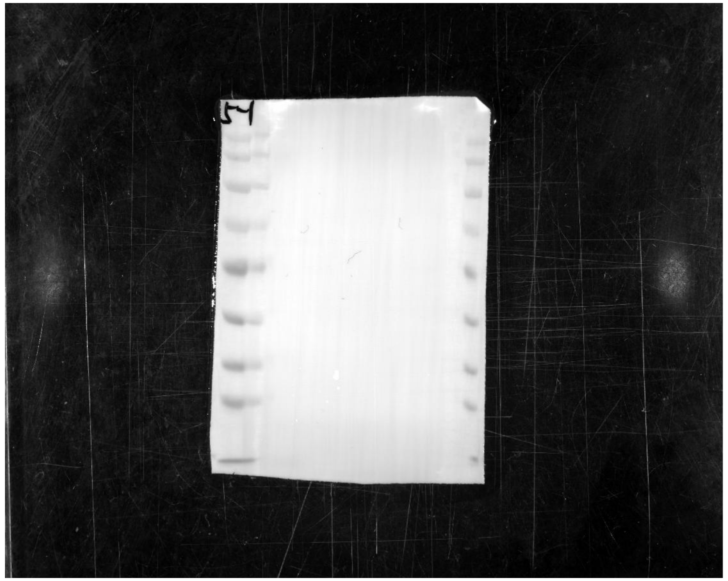

A549 OE β-actin

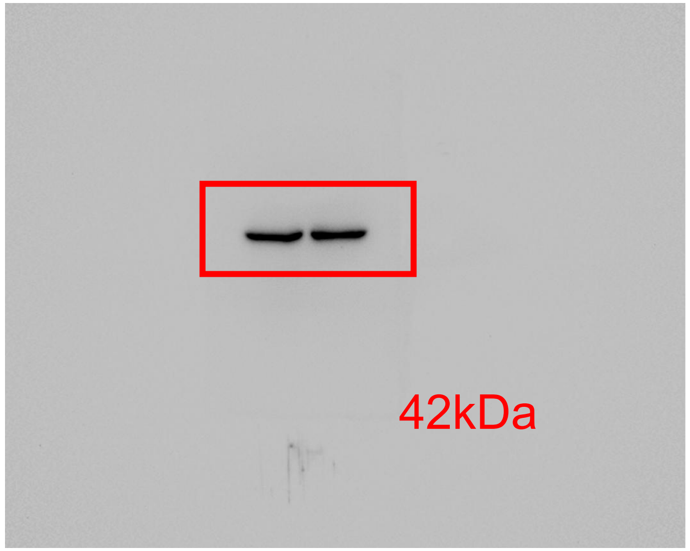

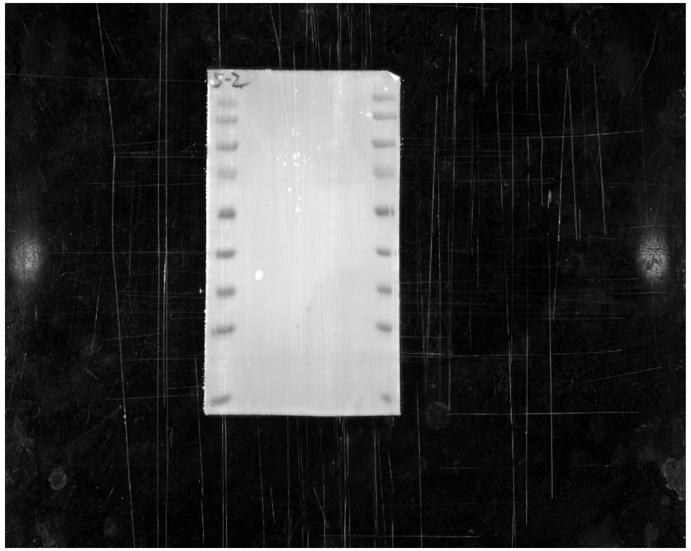

H1299 OE β-actin

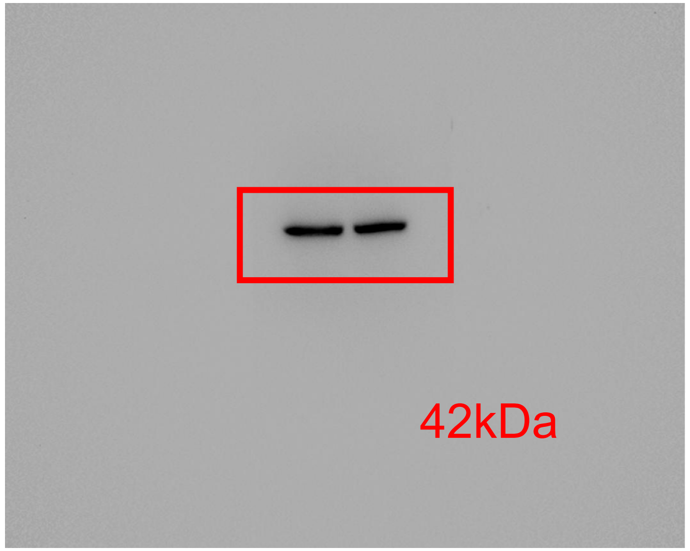

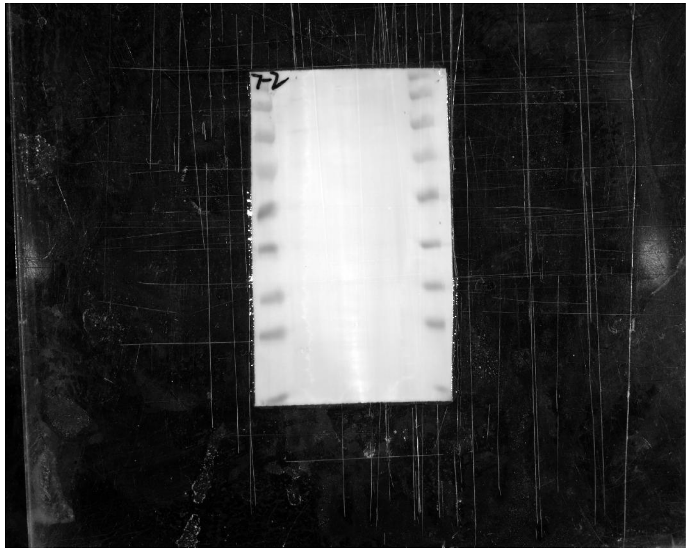

A549 sh β-actin

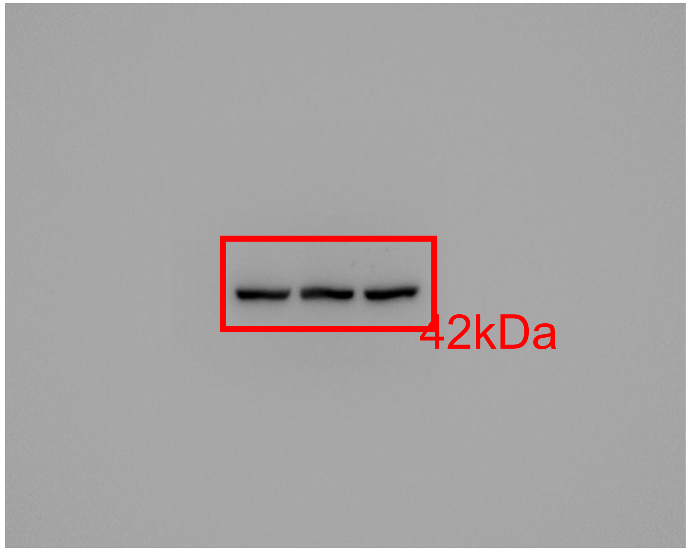

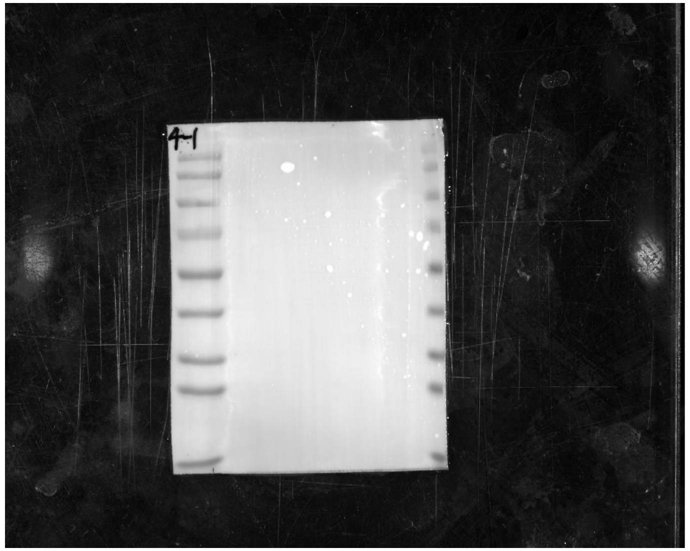

H1299 sh β-actin

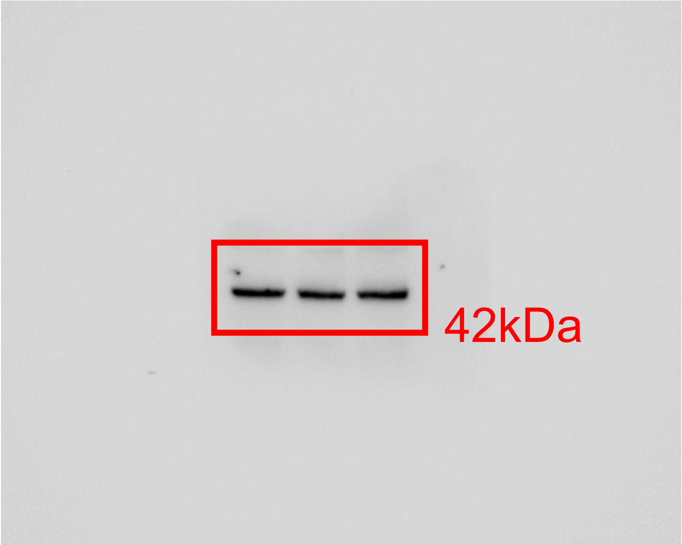

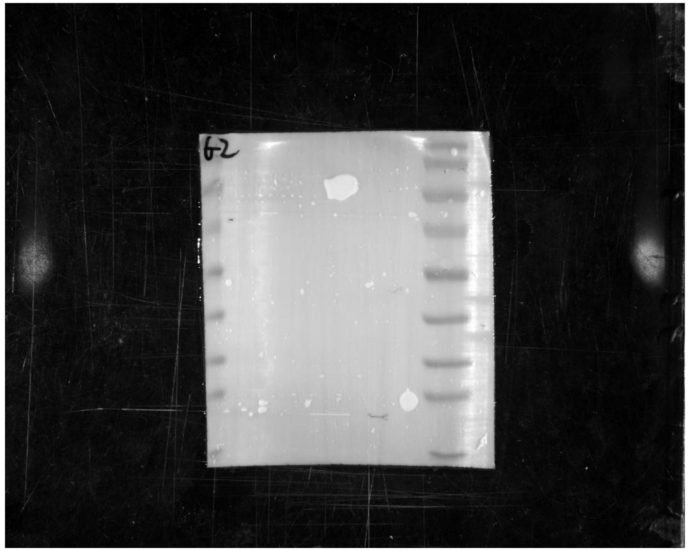

fig3

A549 flag-zc3h15

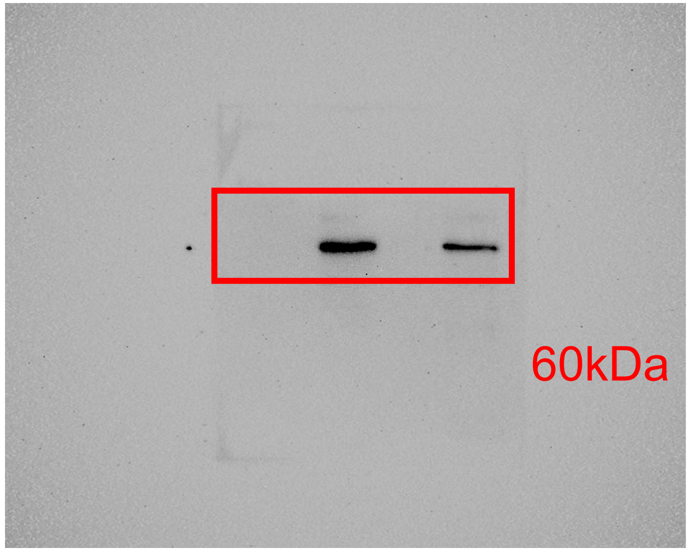

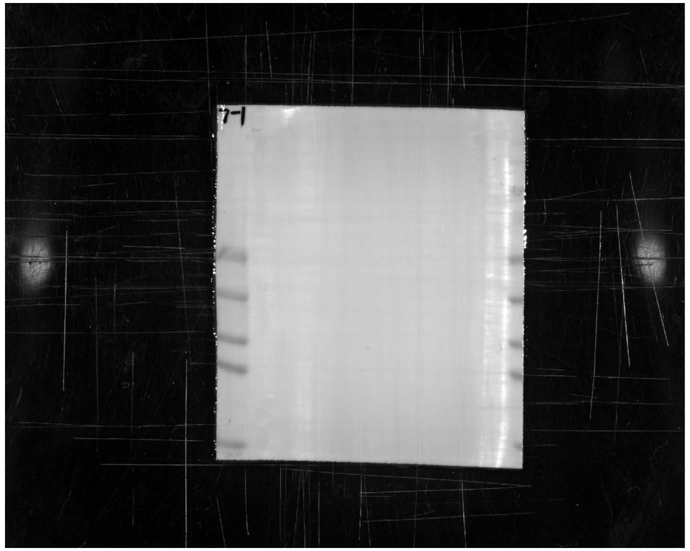

H1299 flag-zc3h15

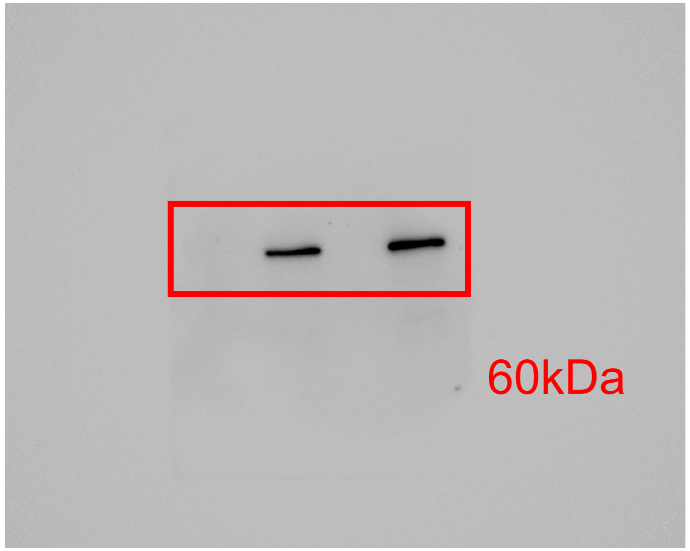

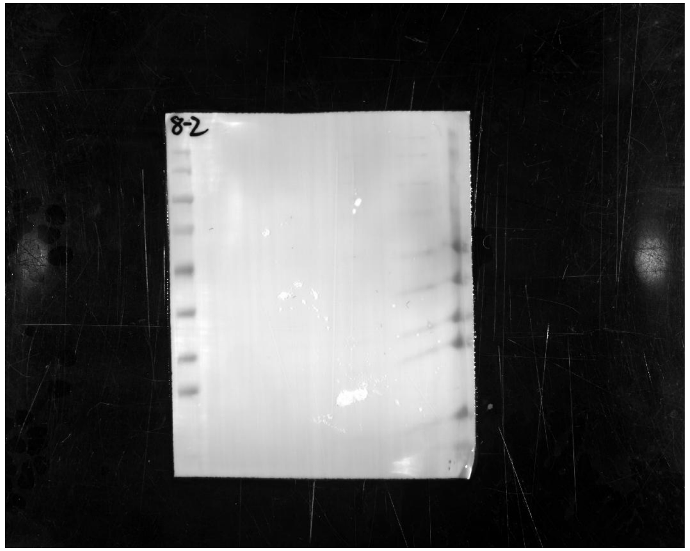

A549 P-AKT

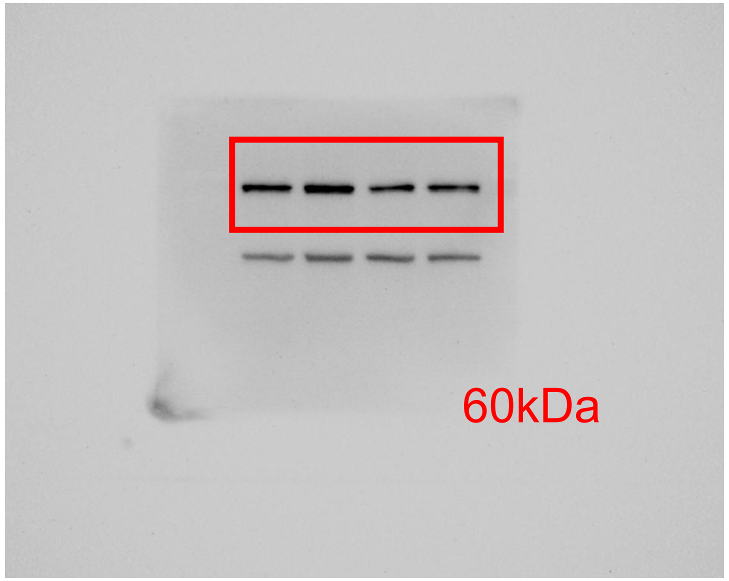

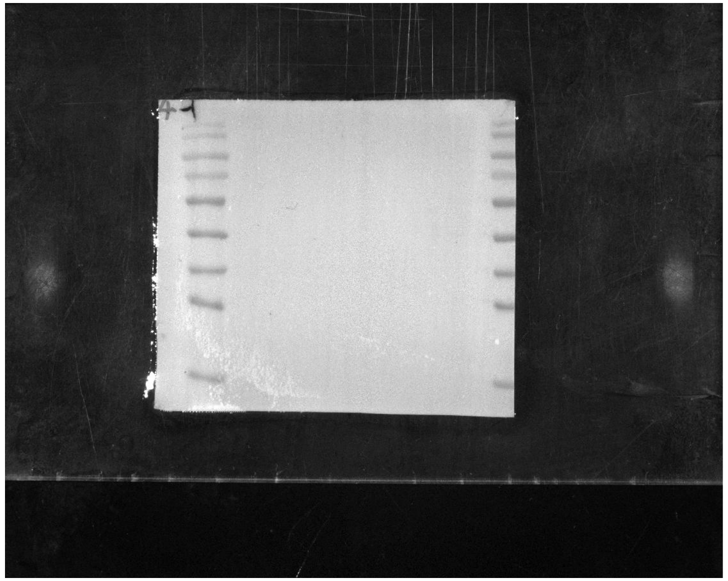

H1299 P-AKT

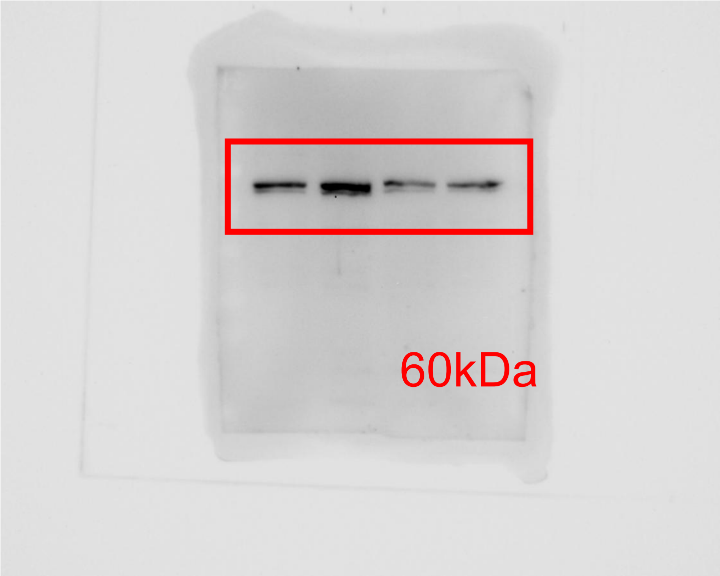

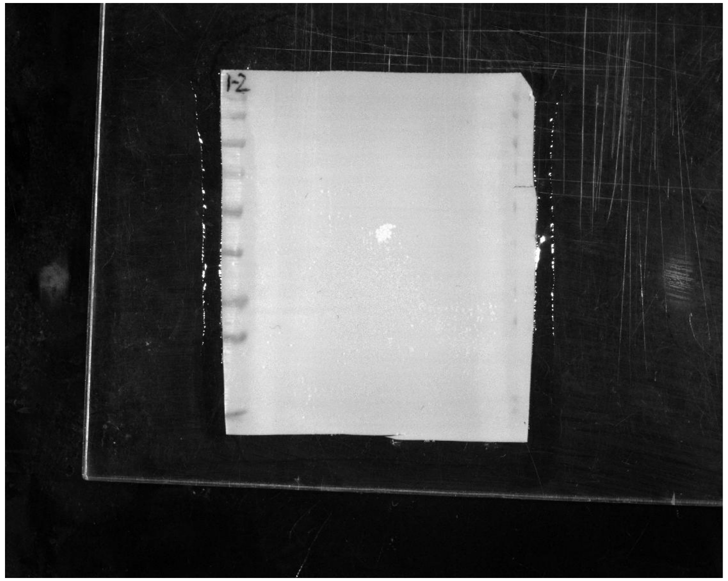

A549 AKT

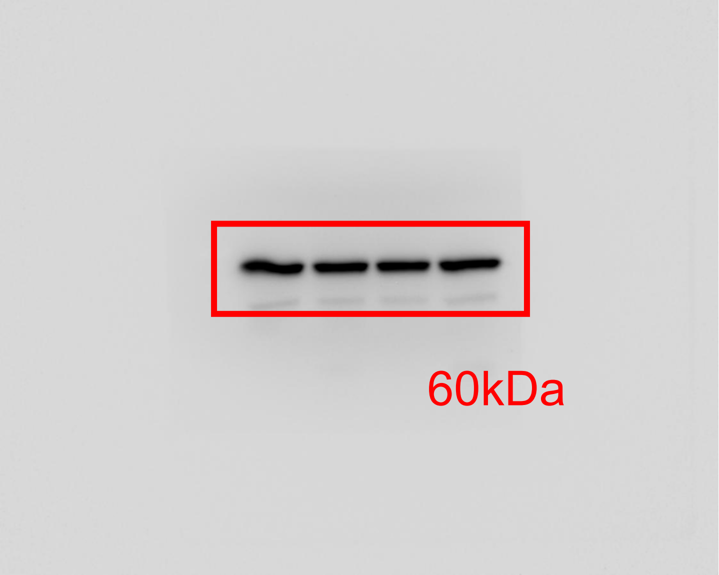

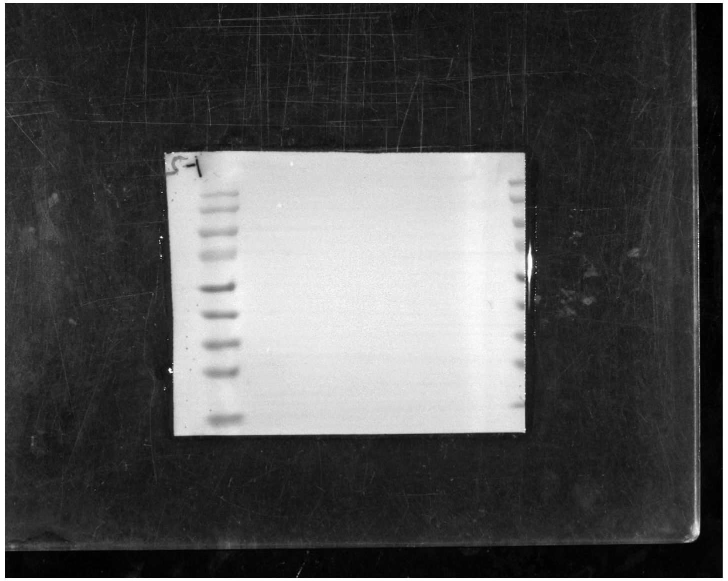

H1299 AKT

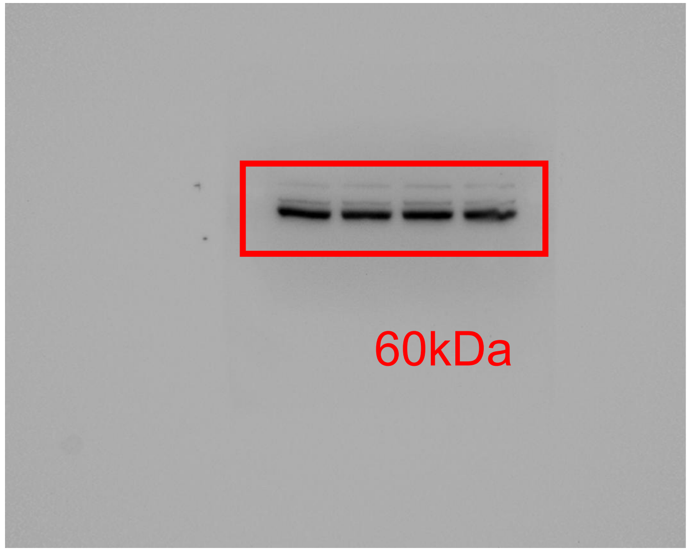

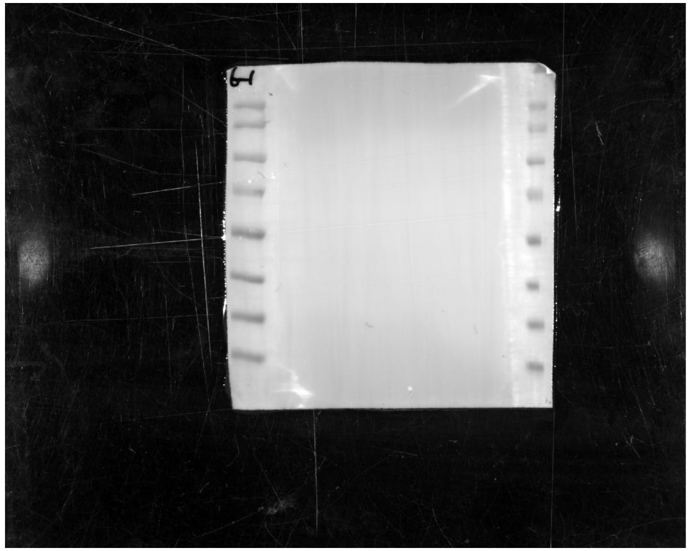

A549 p-mtor

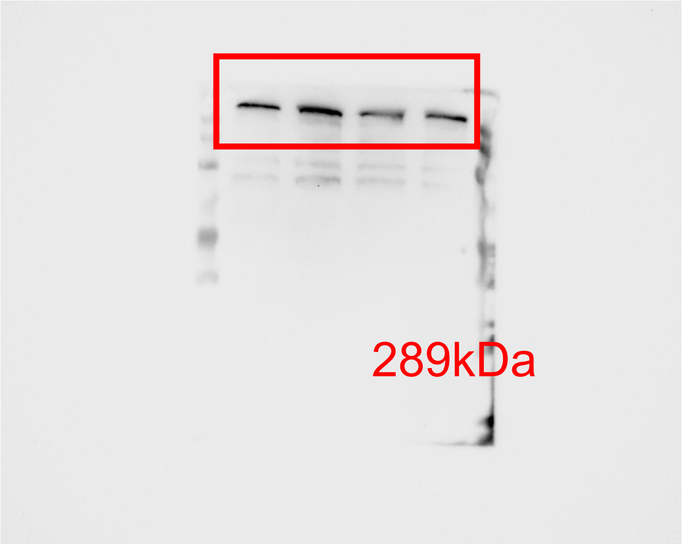

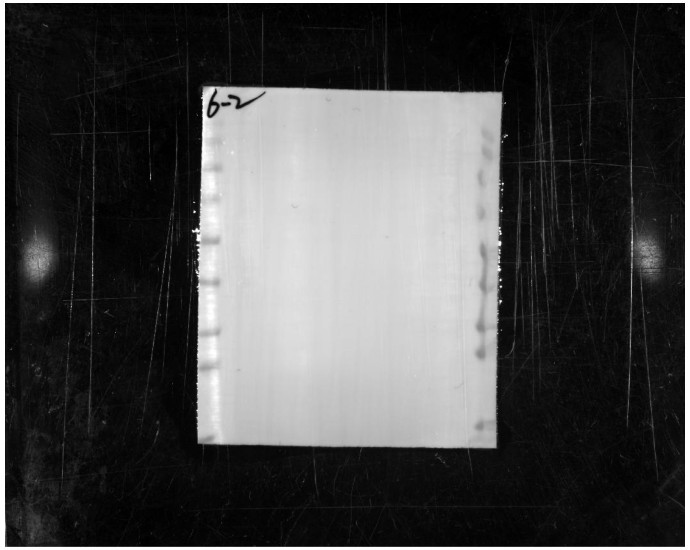

H1299 p-mtor

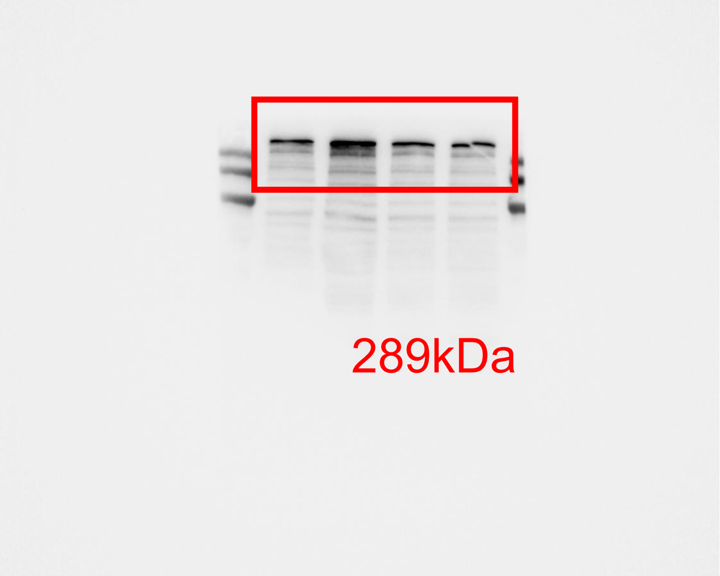

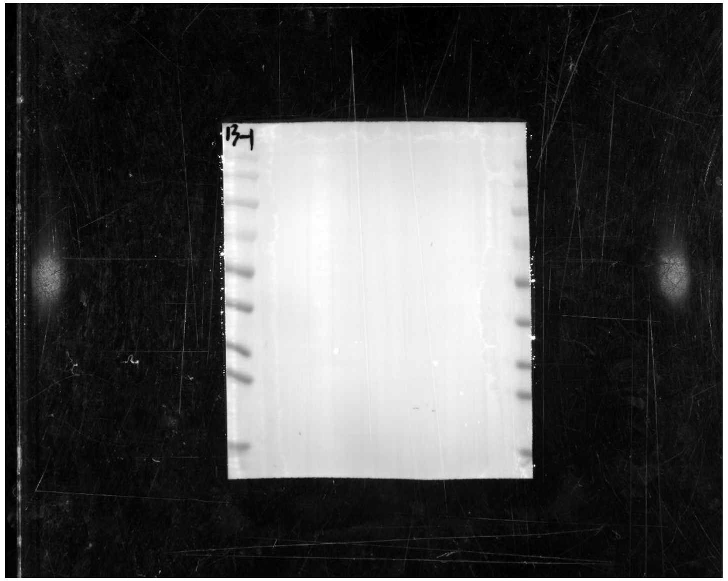

A549 mtor

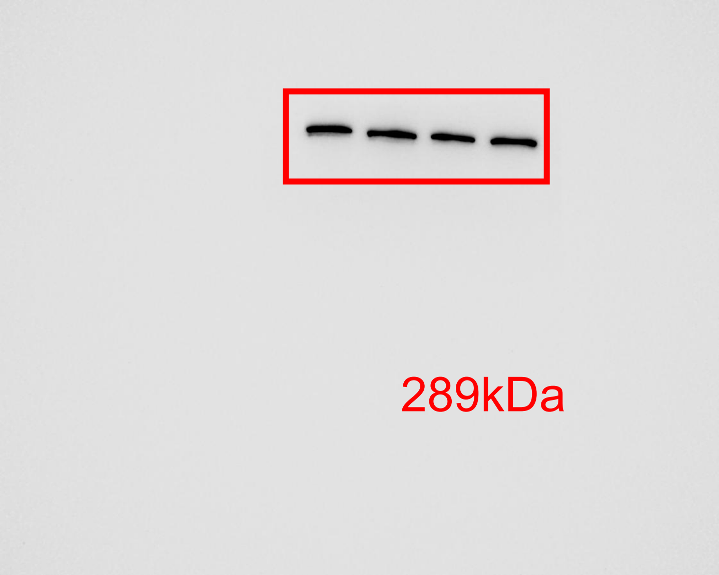

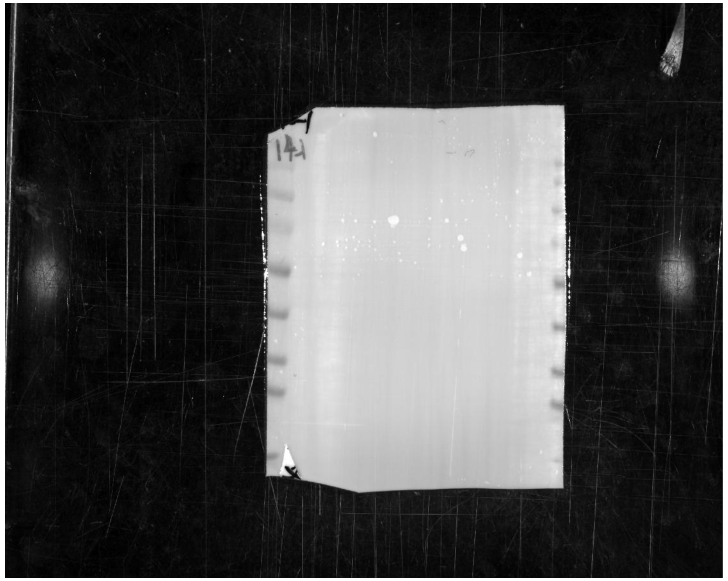

H1299 mtor

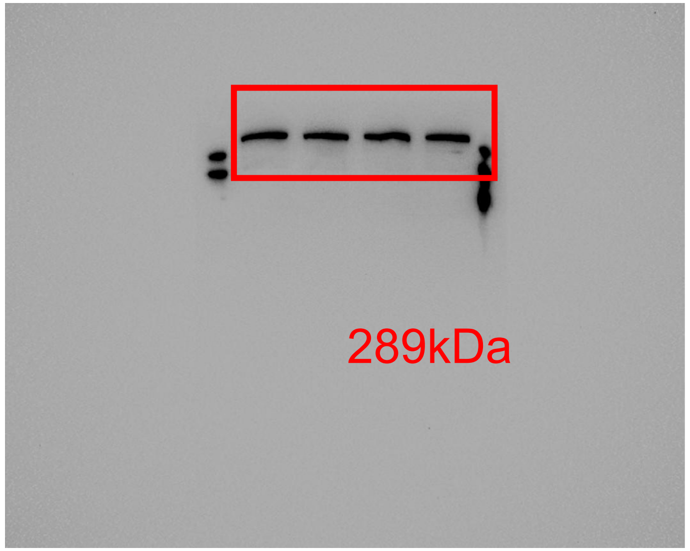

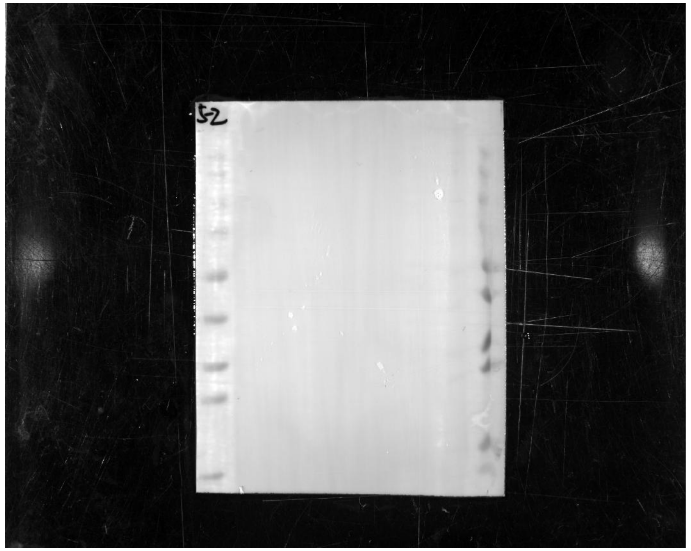

A549 β-actin

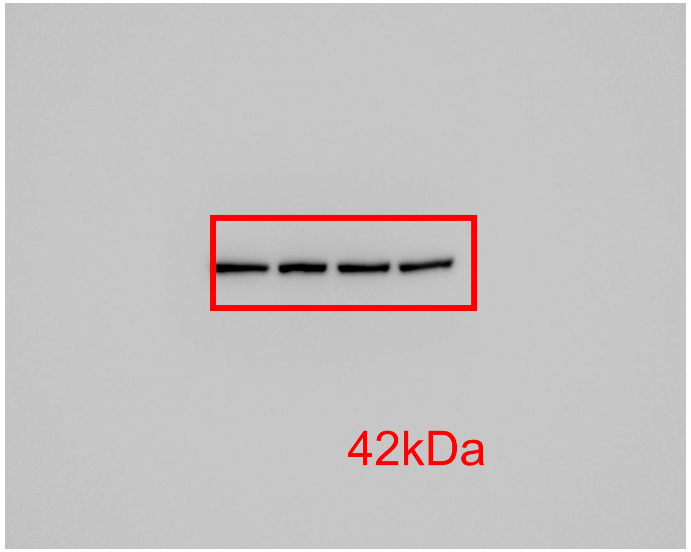

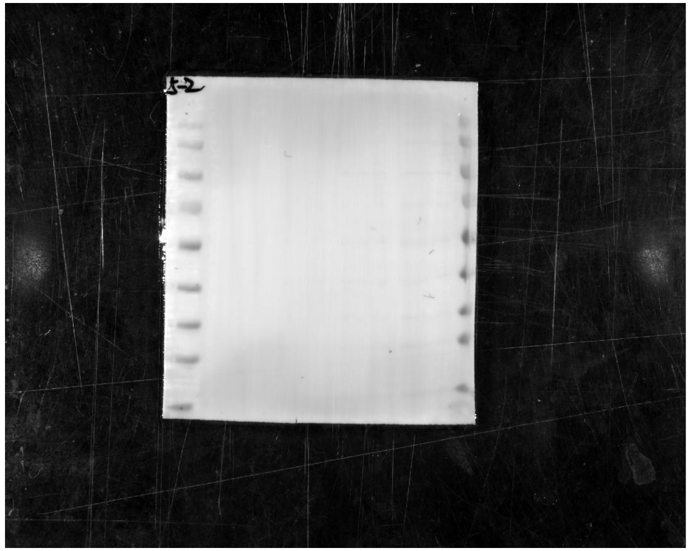

H1299 β-actin

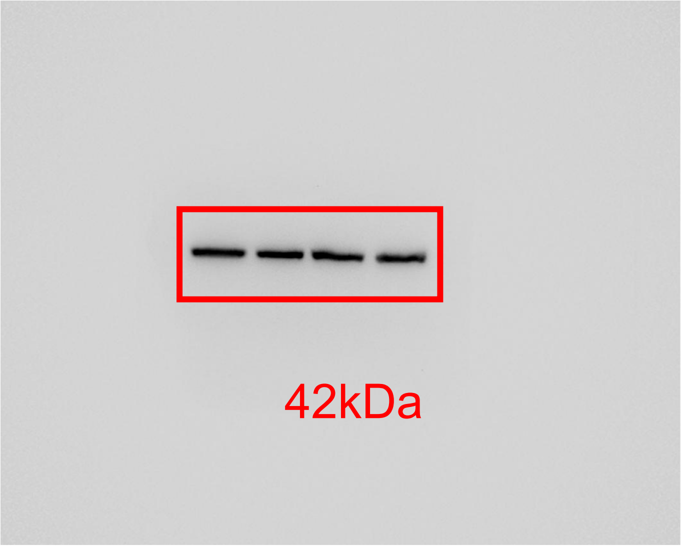

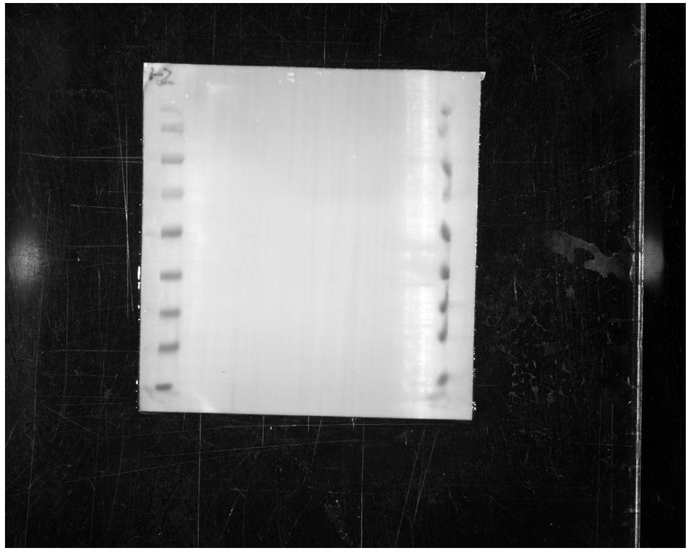

S-fig3

A549 OE P-AKT

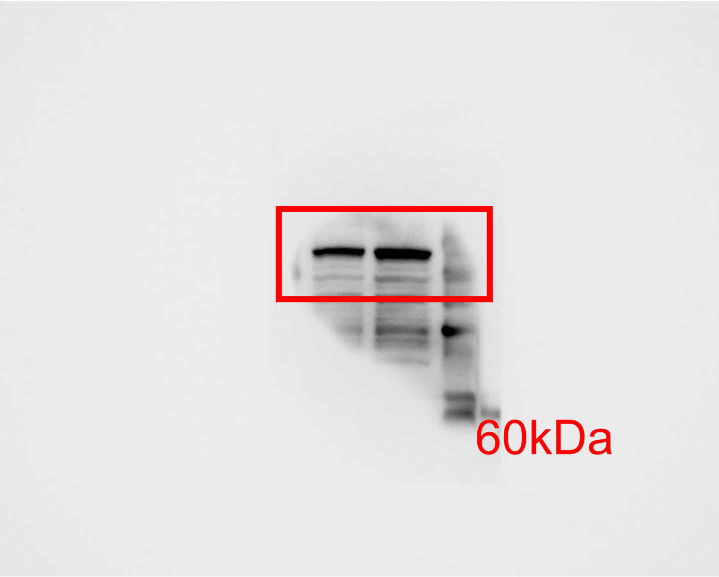

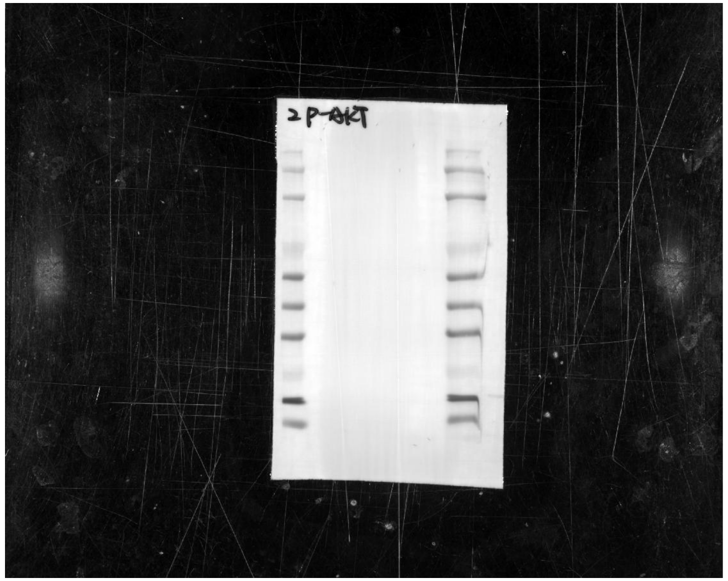

H1299 OE P-AKT

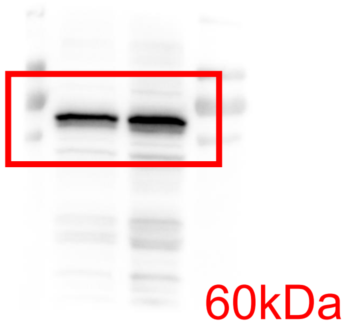

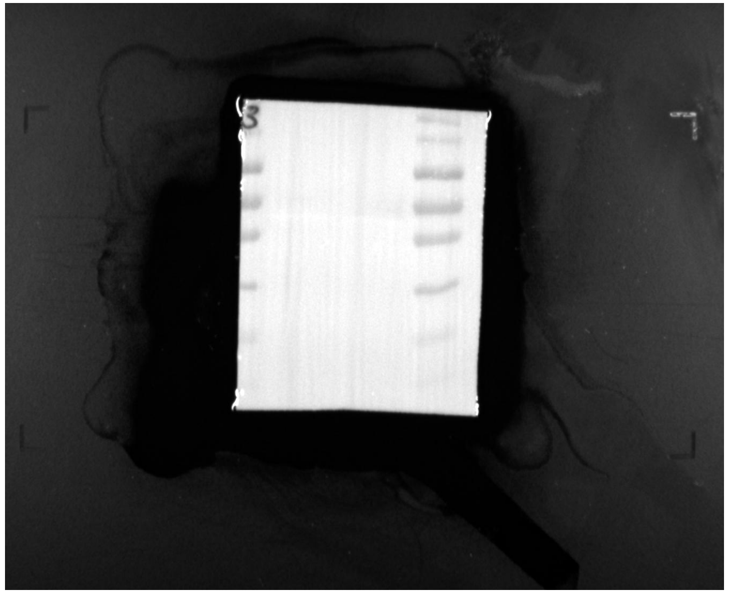

A549 OE AKT

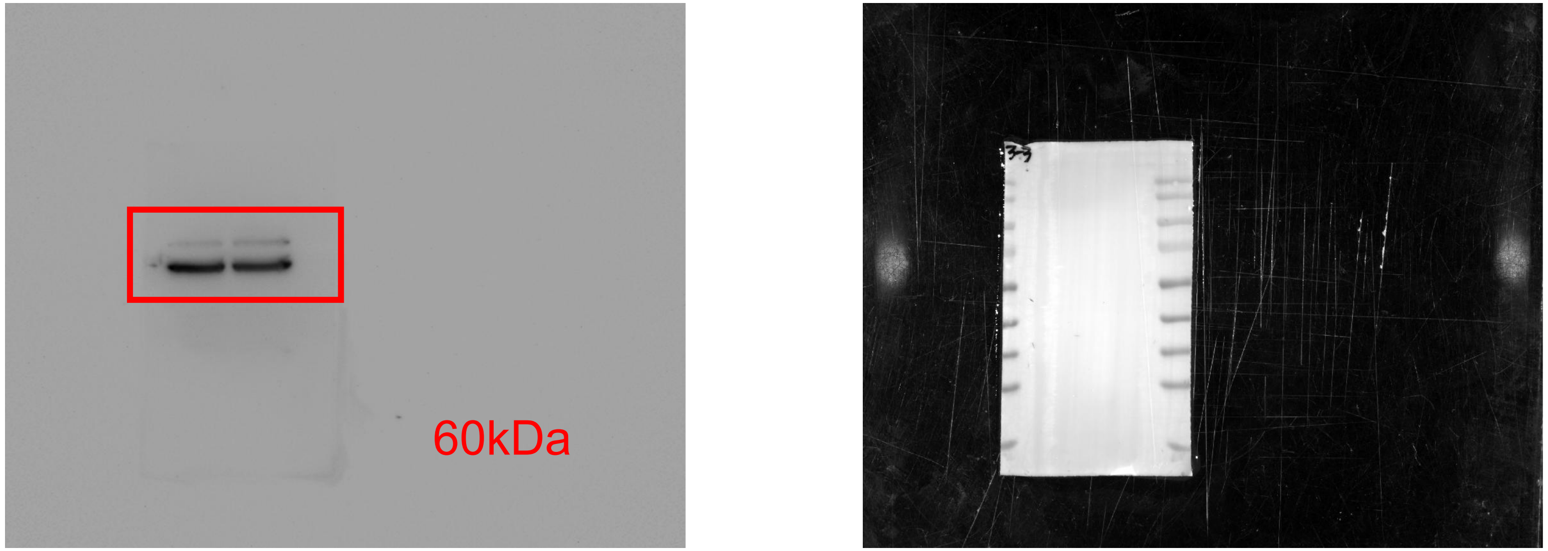

H1299 OE AKT

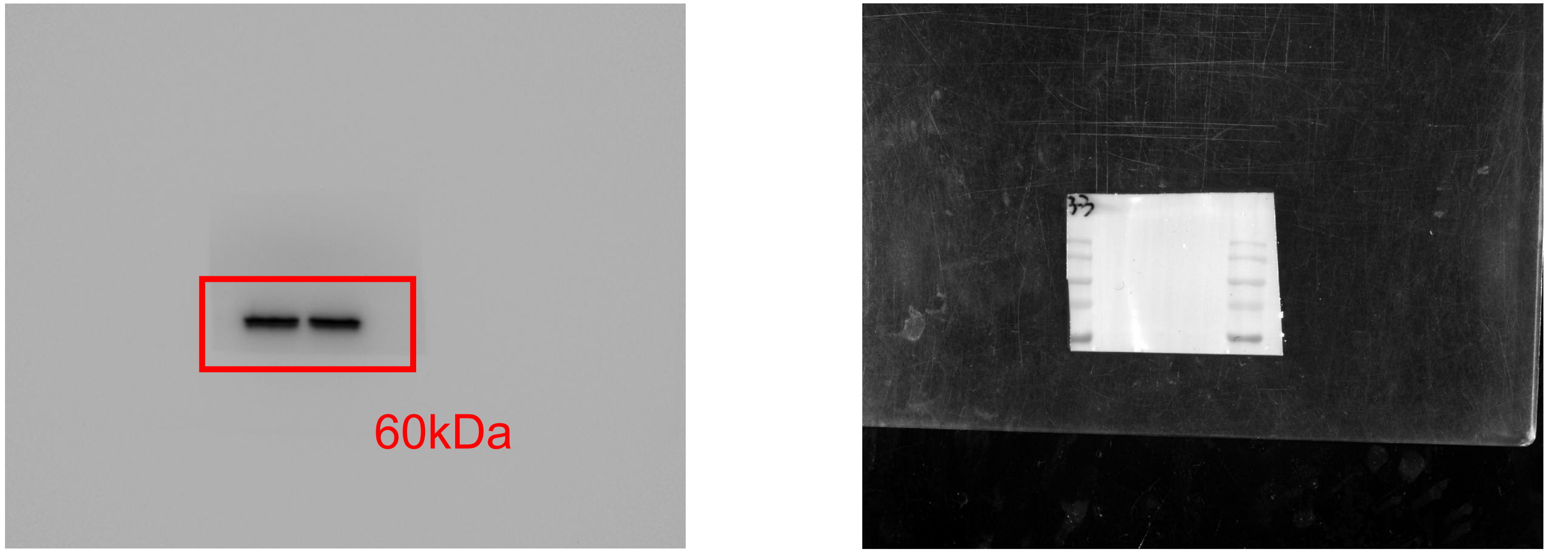

A549 OE p-mtor

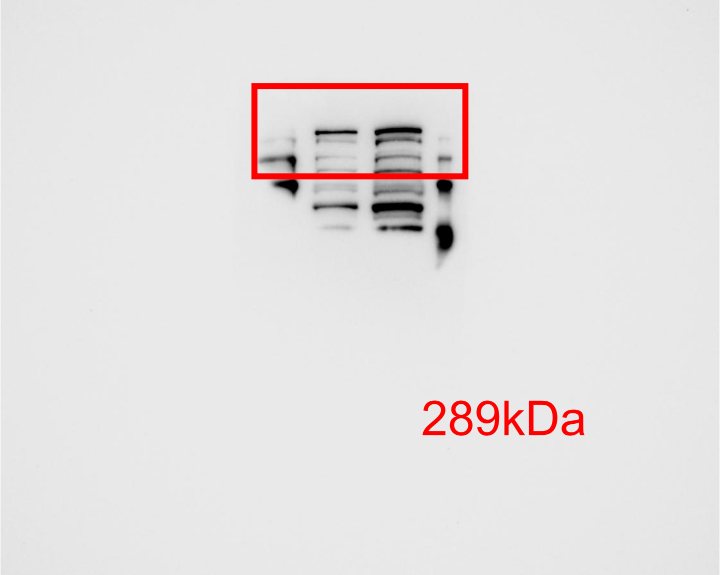

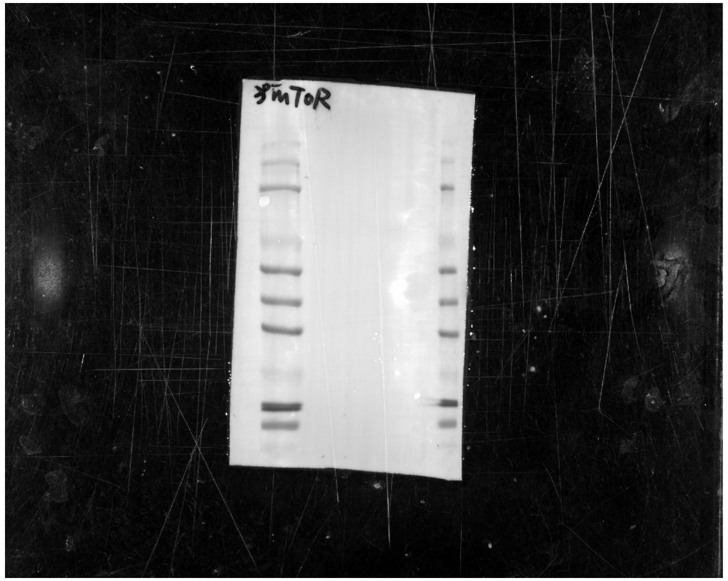

H1299 OE p-mtor

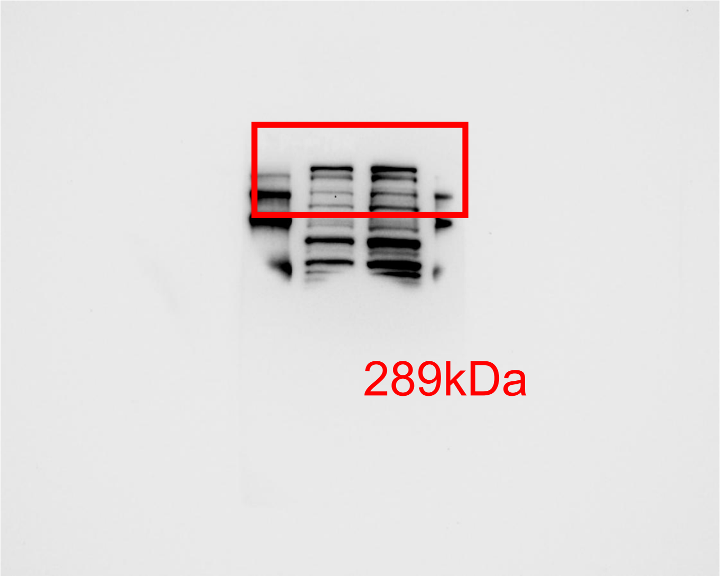

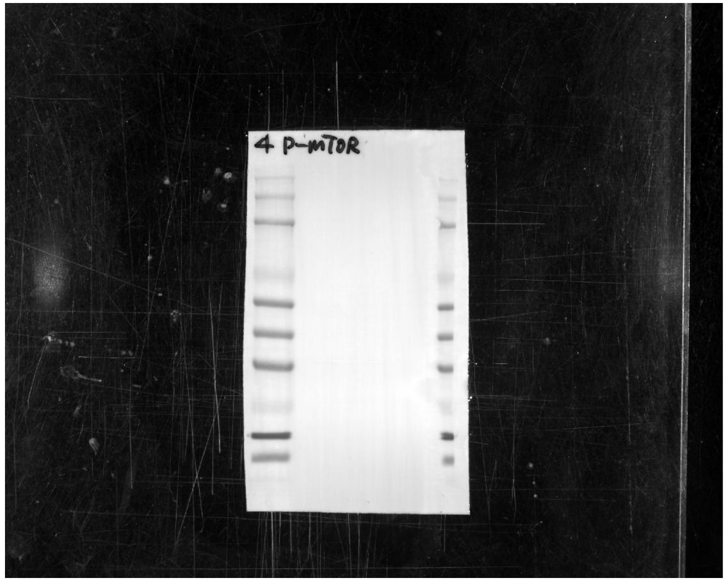

A549 OE mtor

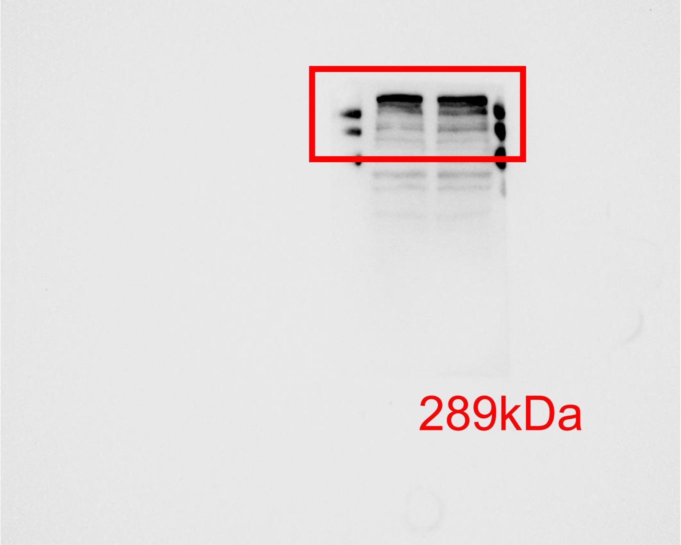

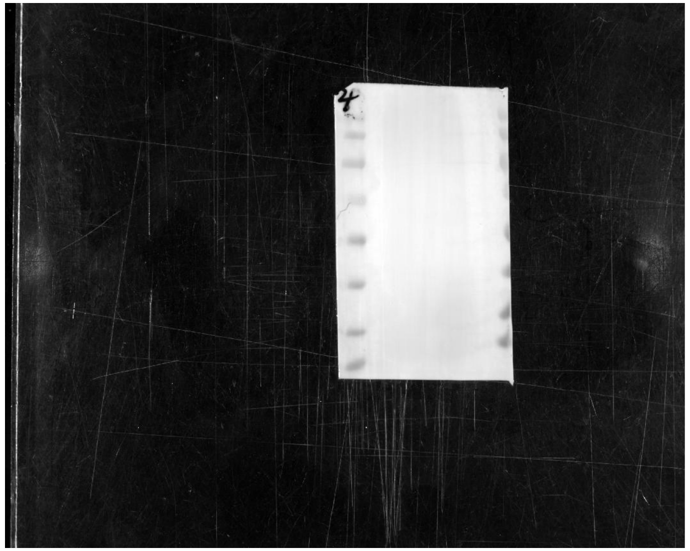

H1299 OE mtor

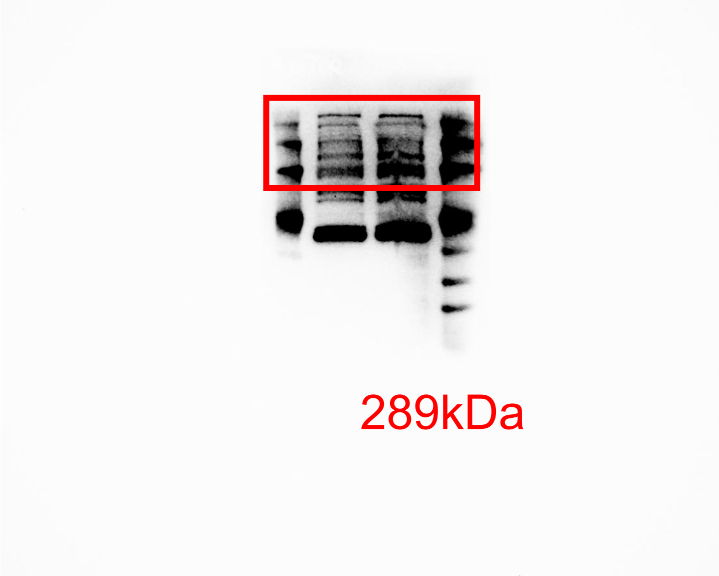

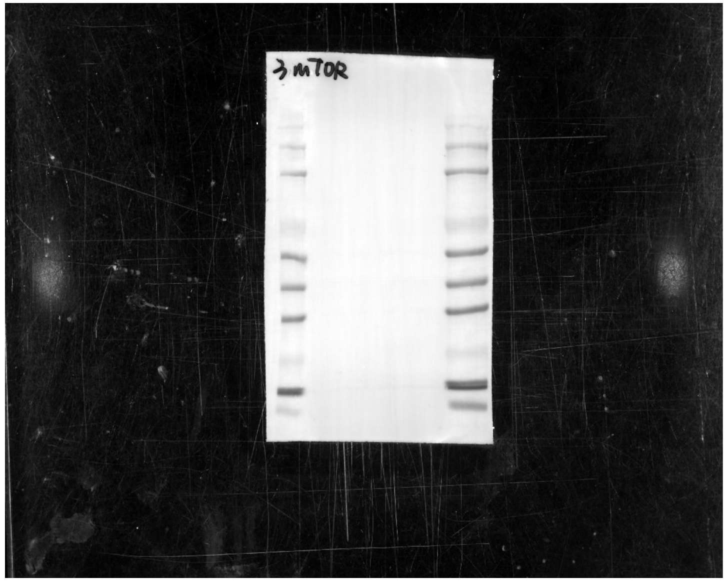

A549 OE β-actin

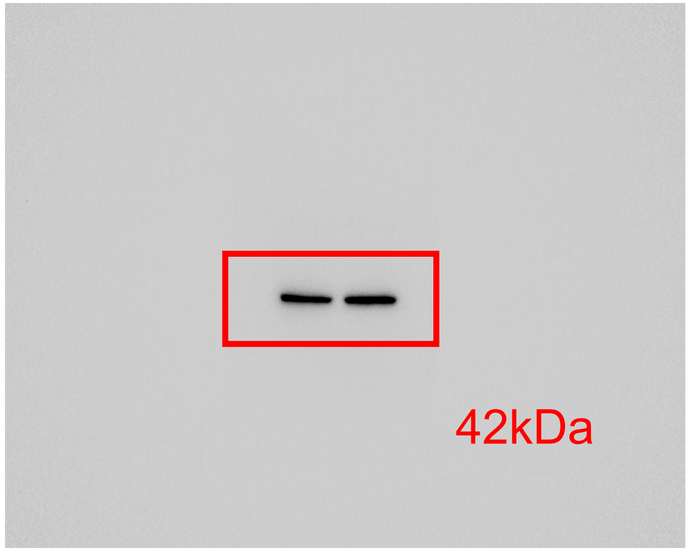

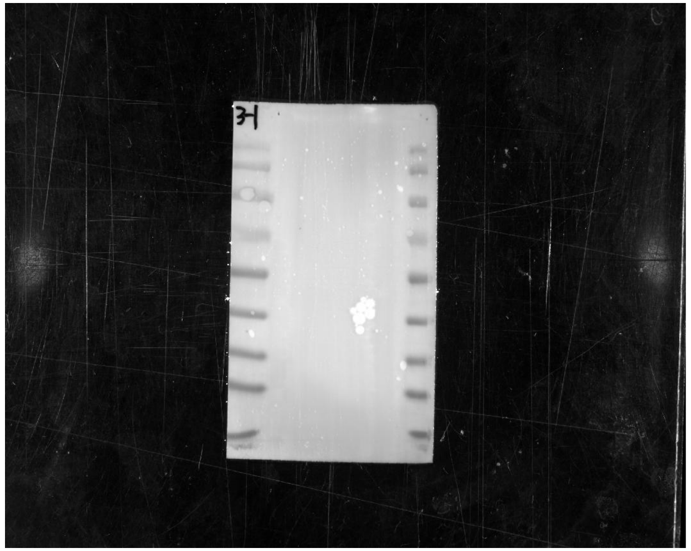

H1299 OE β-actin

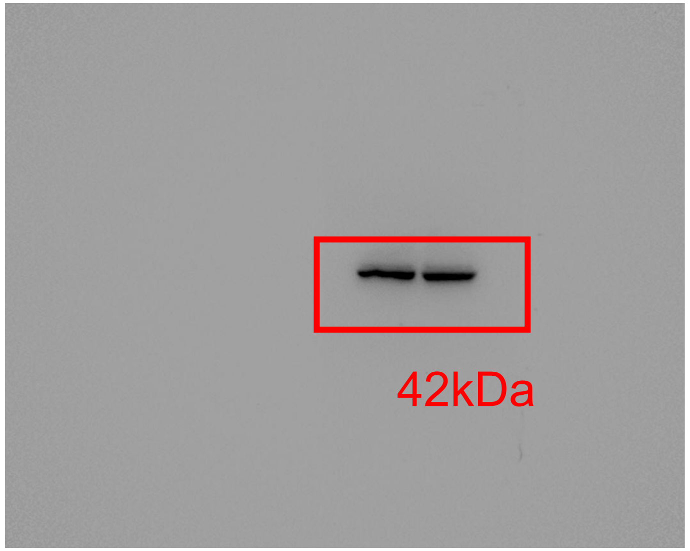

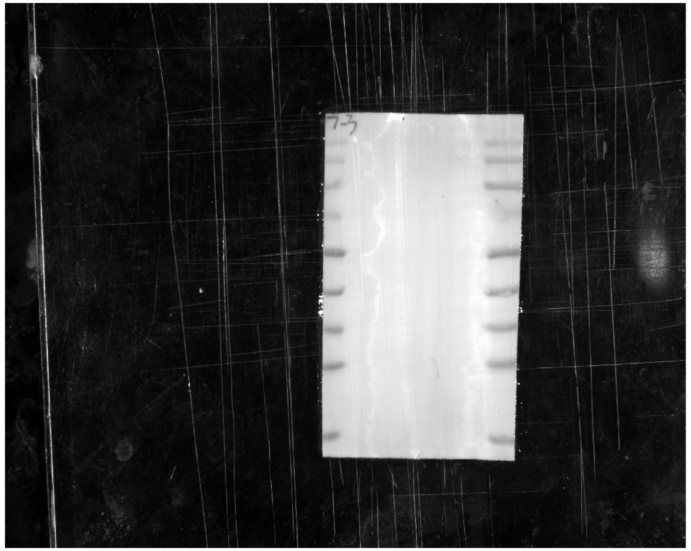

A549 sh P-AKT

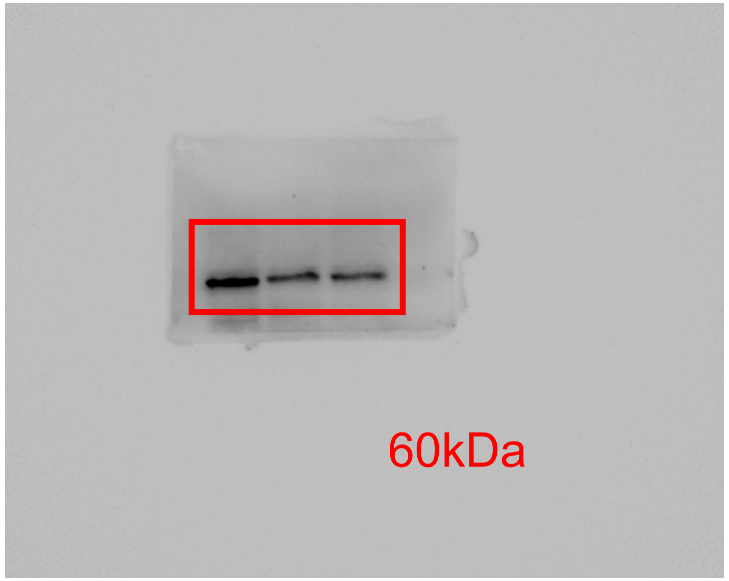

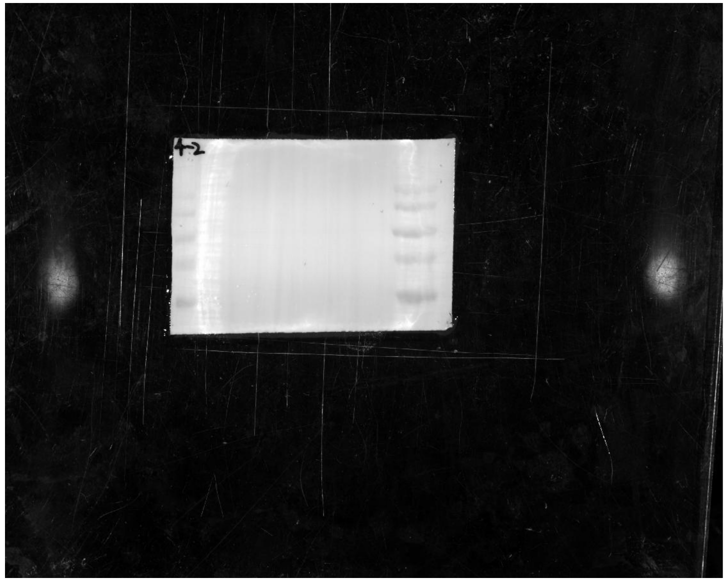

H1299 sh P-AKT

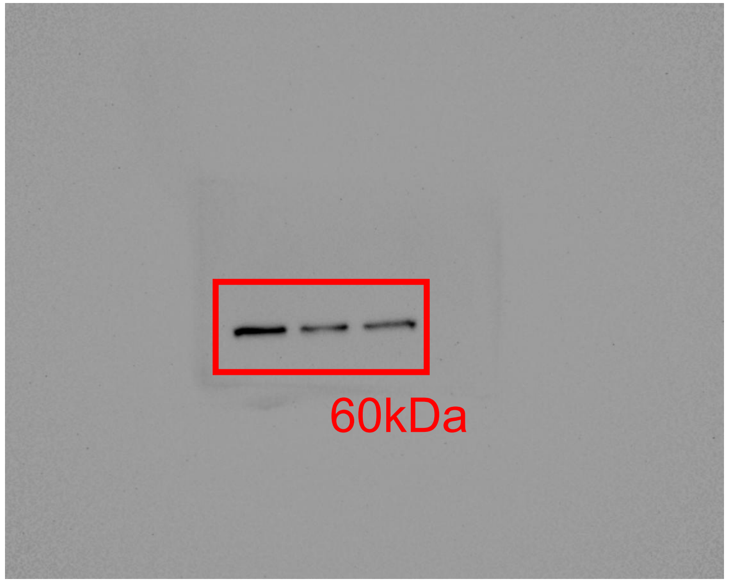

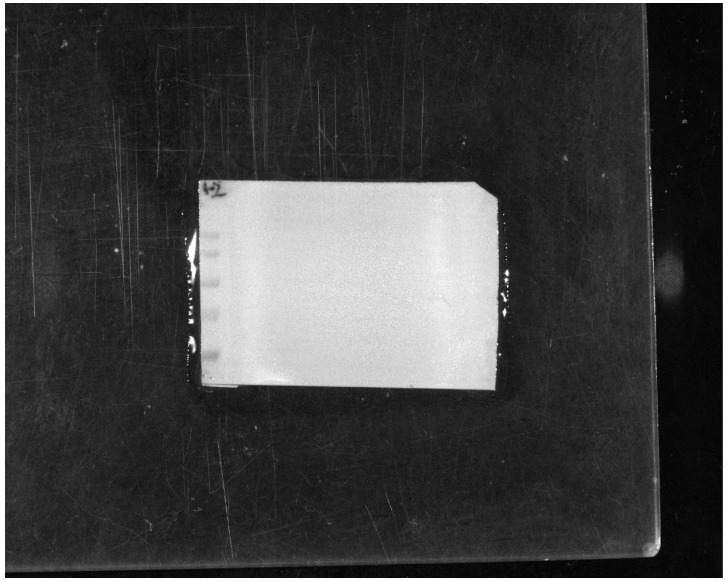

A549 sh AKT

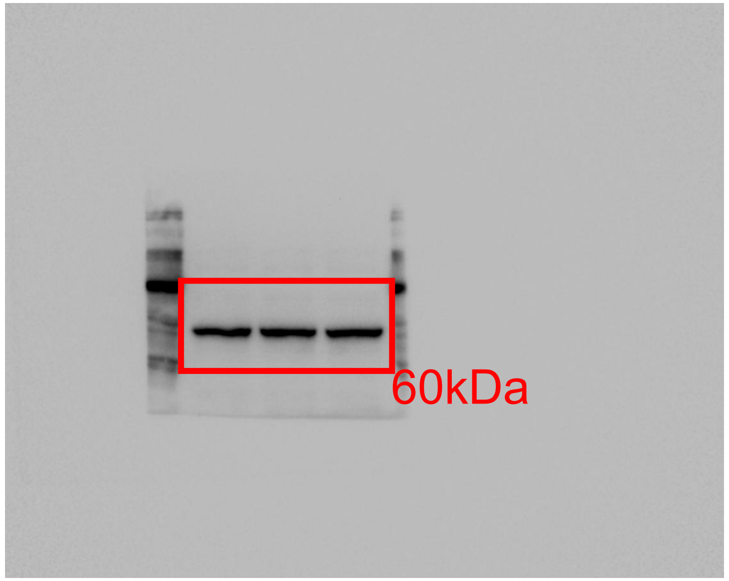

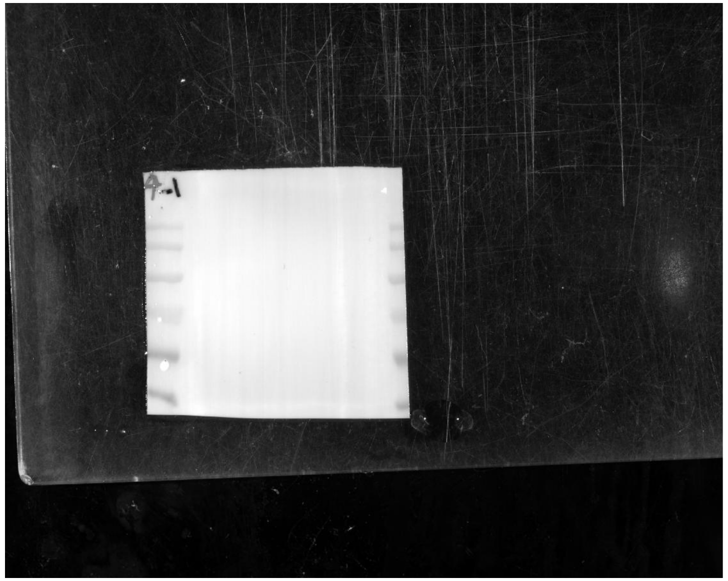

H1299 sh AKT

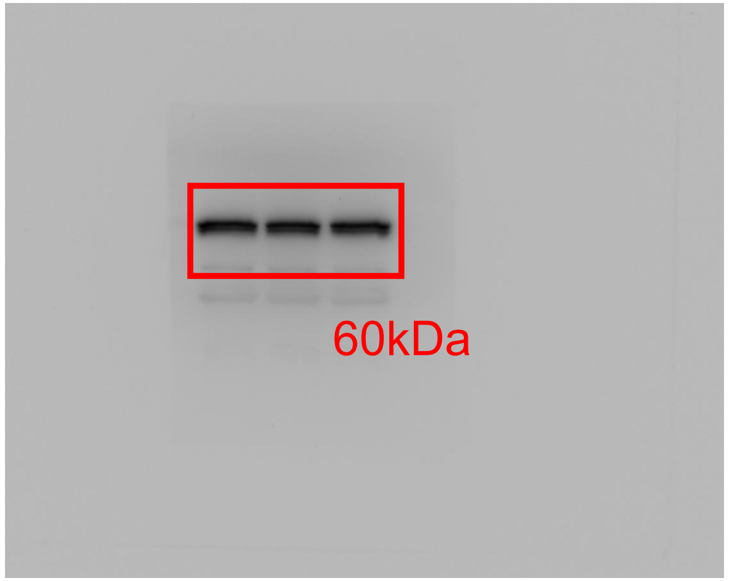

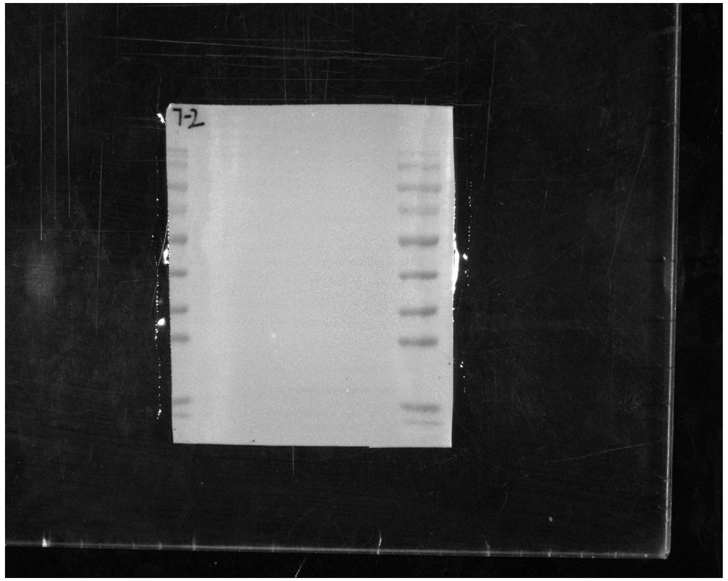

A549 sh p-mtor

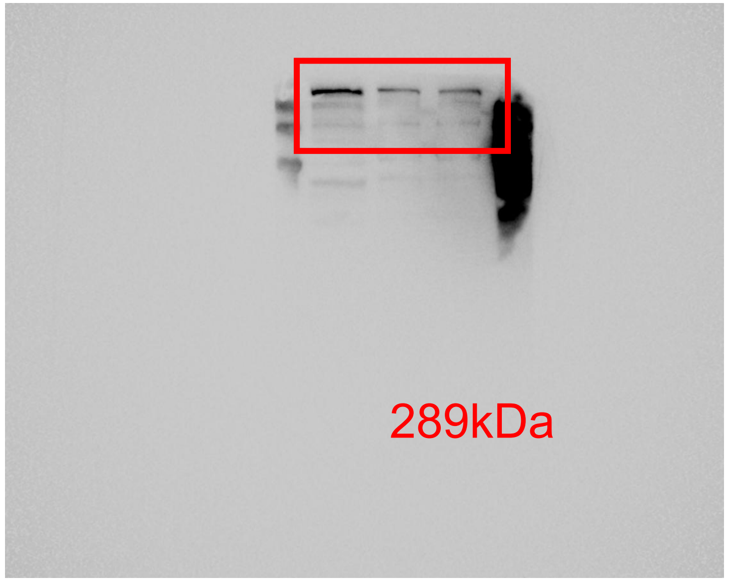

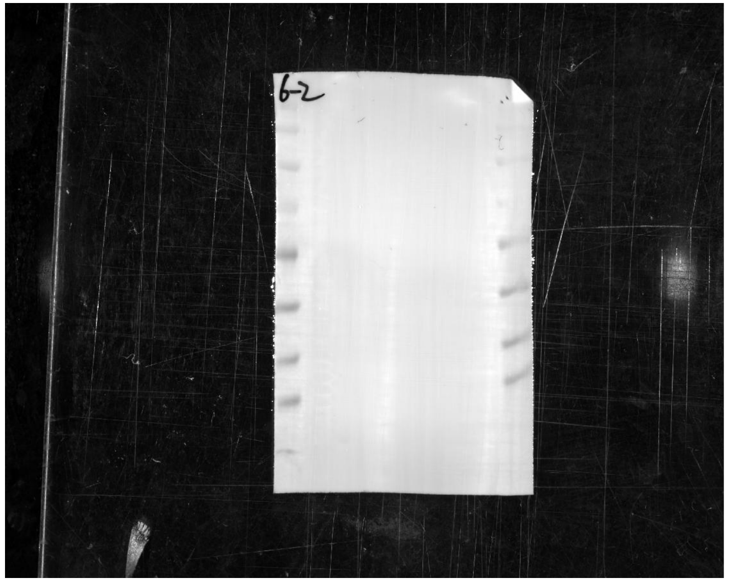

H1299 sh p-mtor

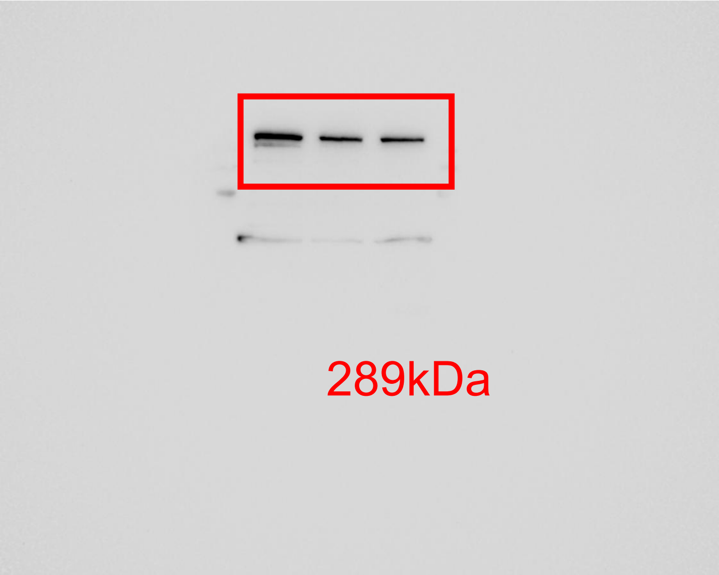

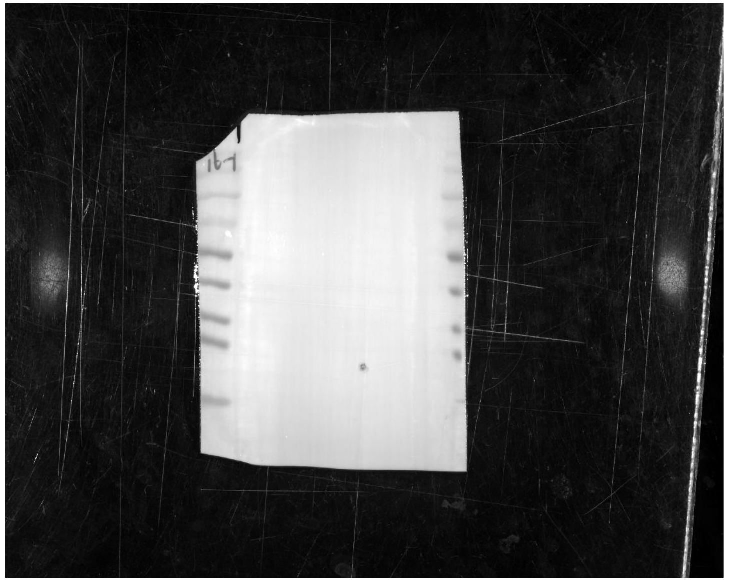

A549 sh mtor

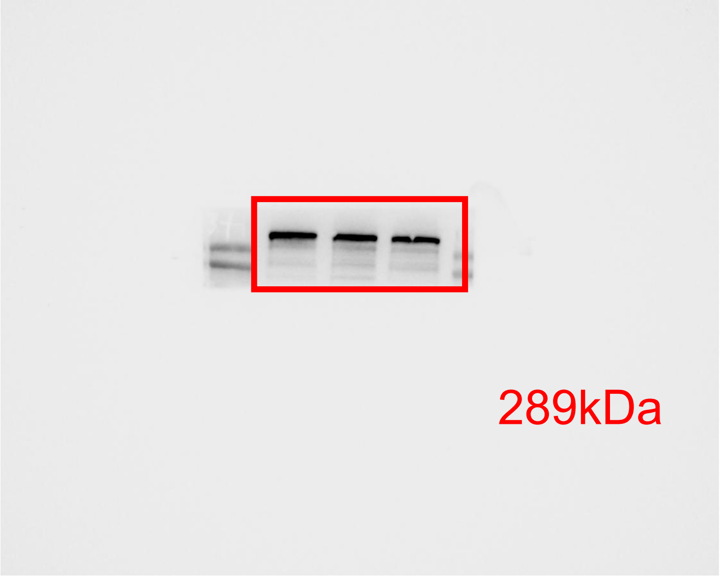

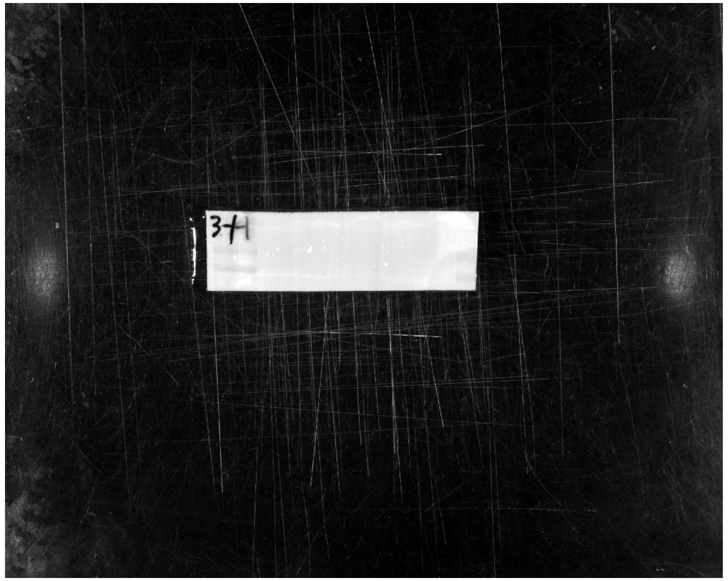

H1299 sh mtor

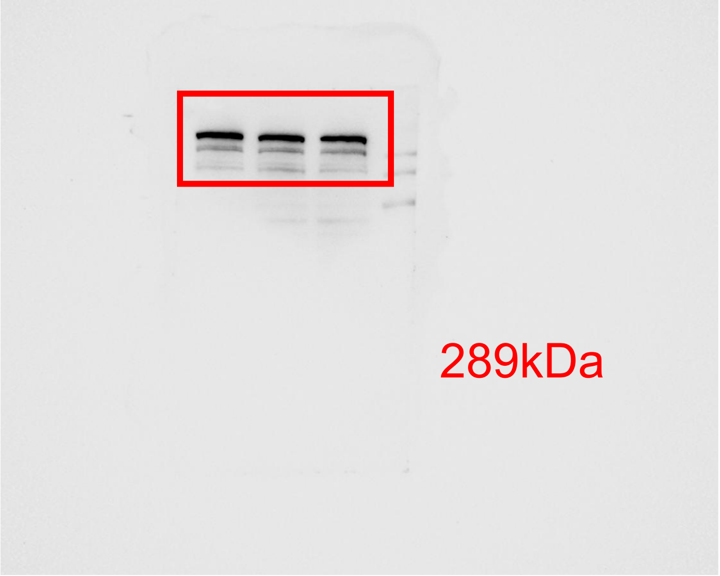

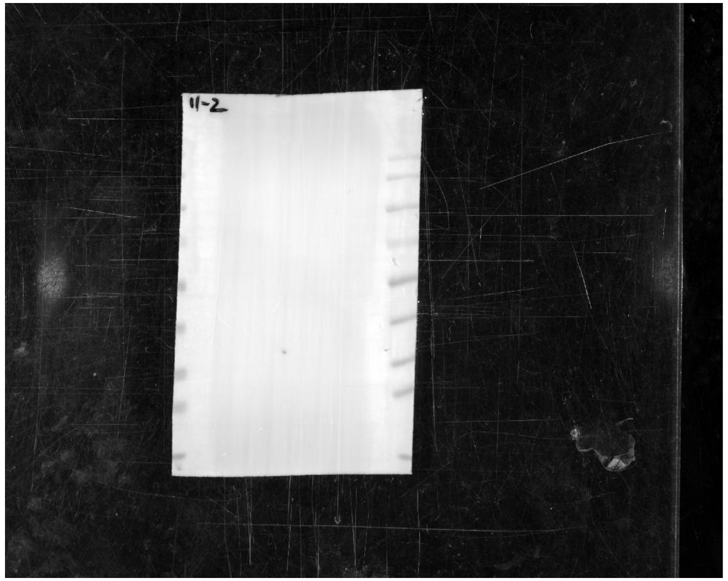

A549 sh β-actin

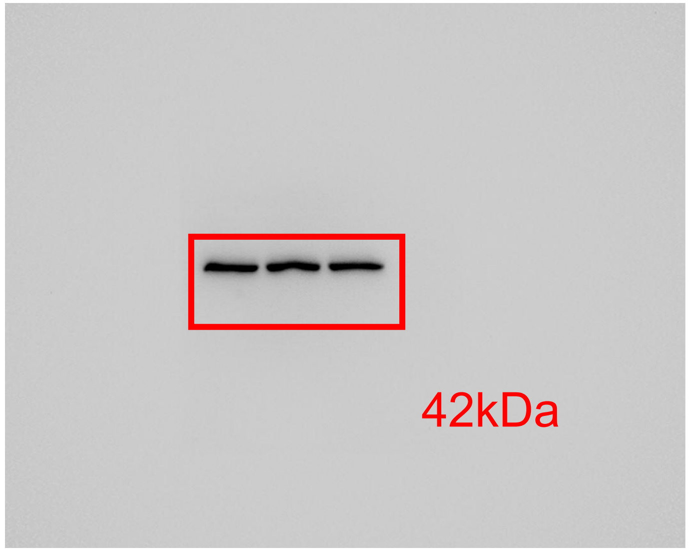

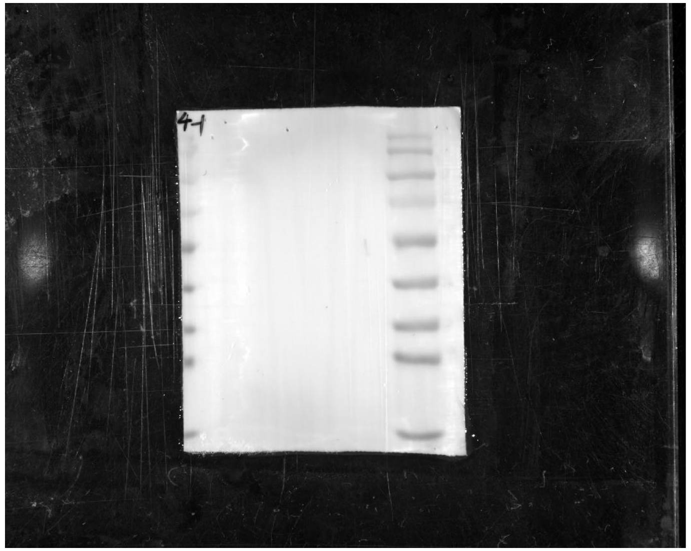

H1299 sh β-actin

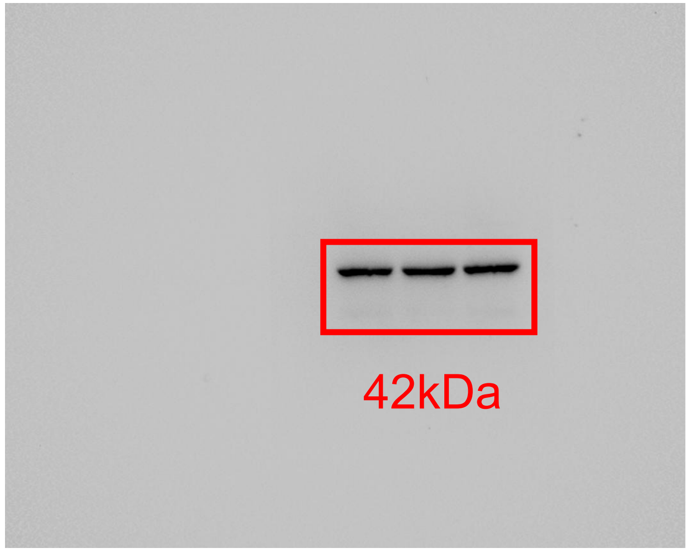

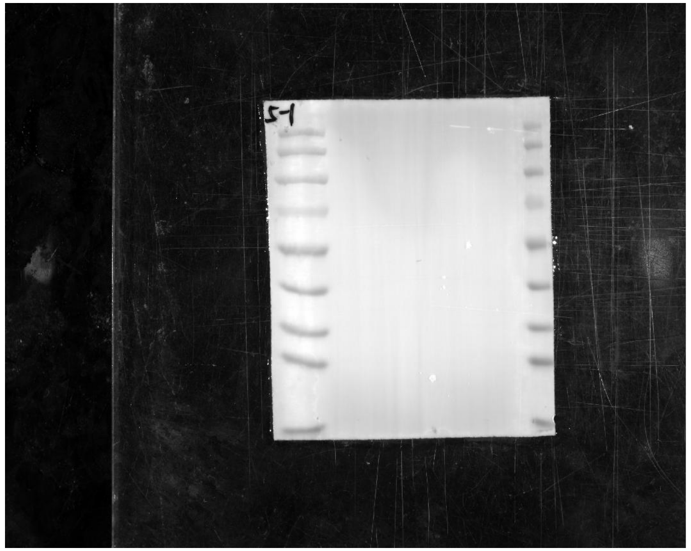

S-fig5

A549 CDK4

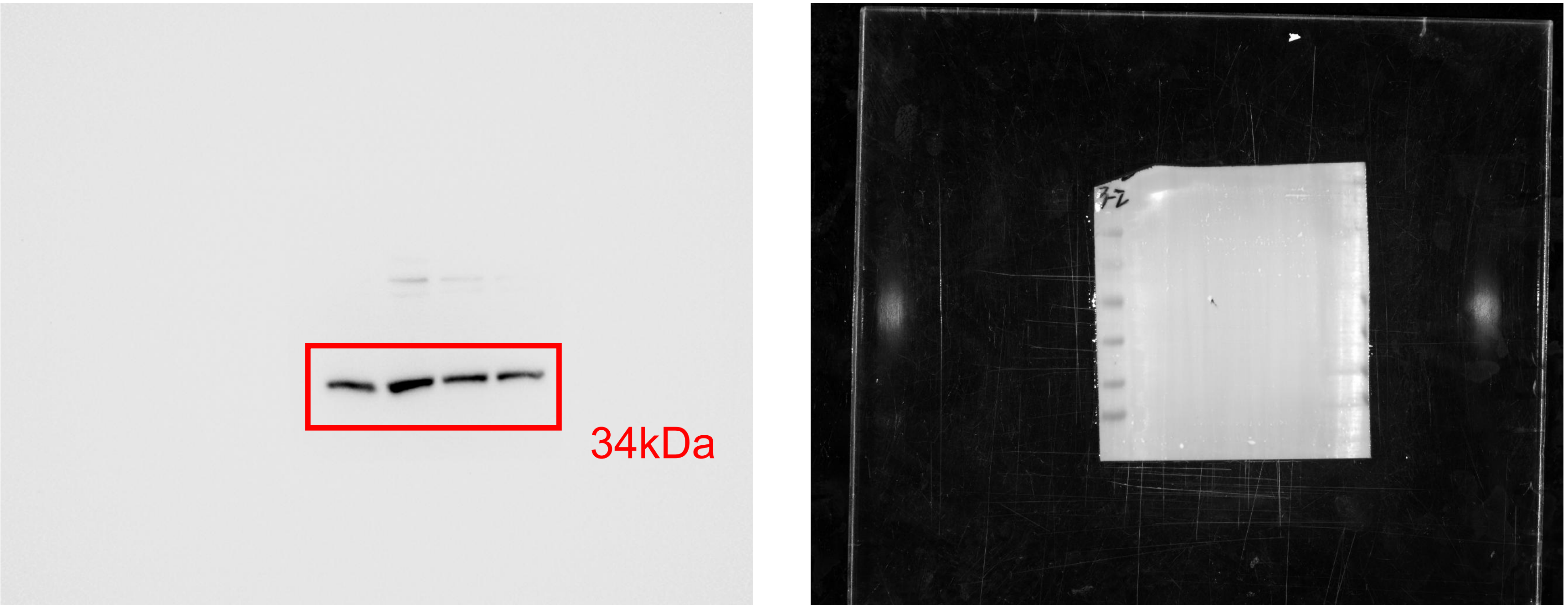

H1299 CDK4

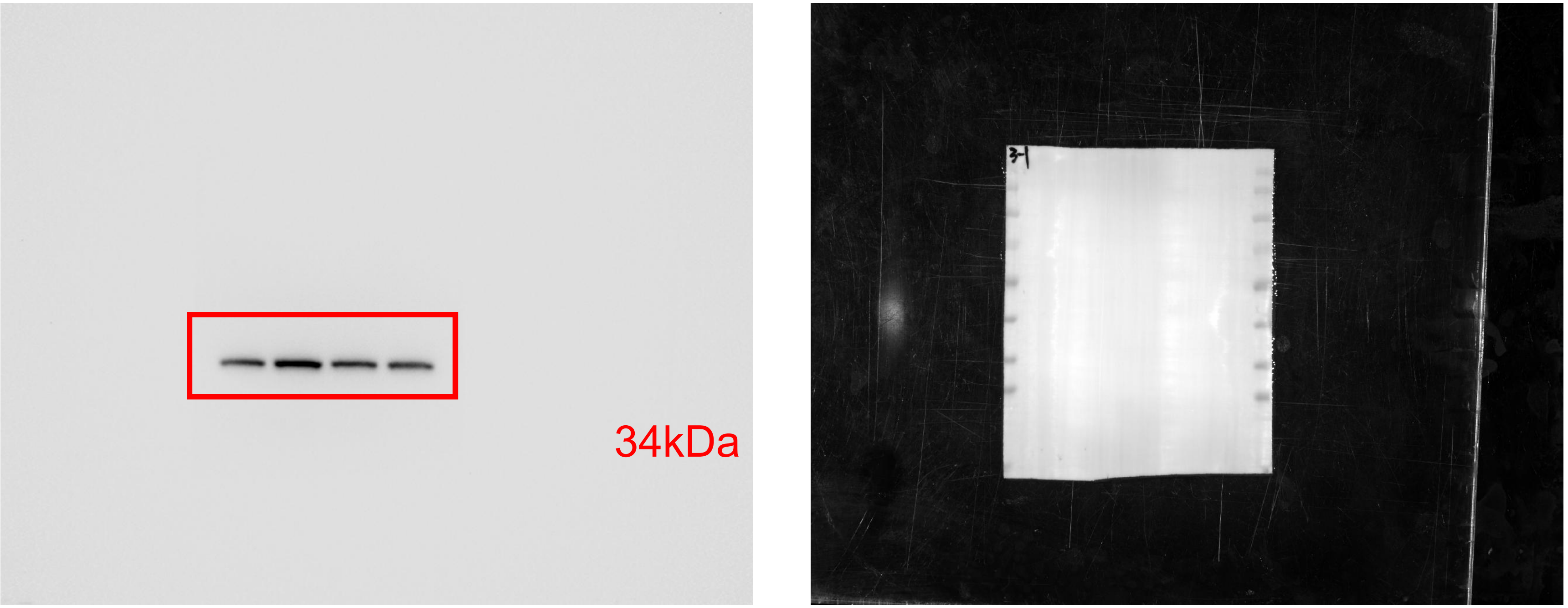

A549 CDK6

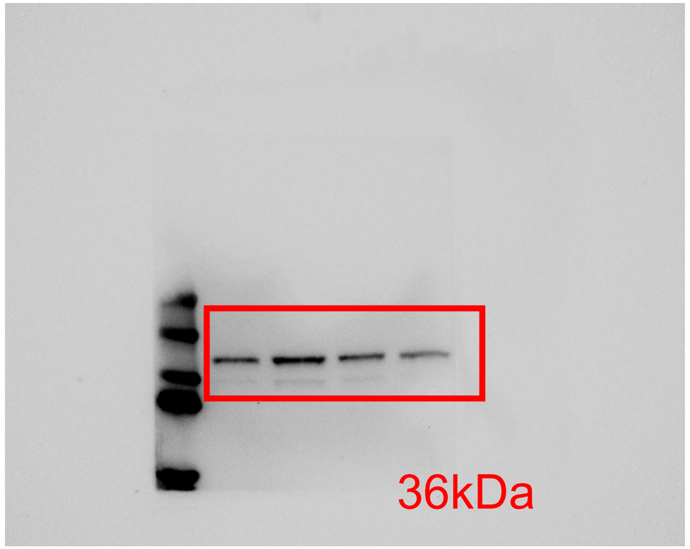

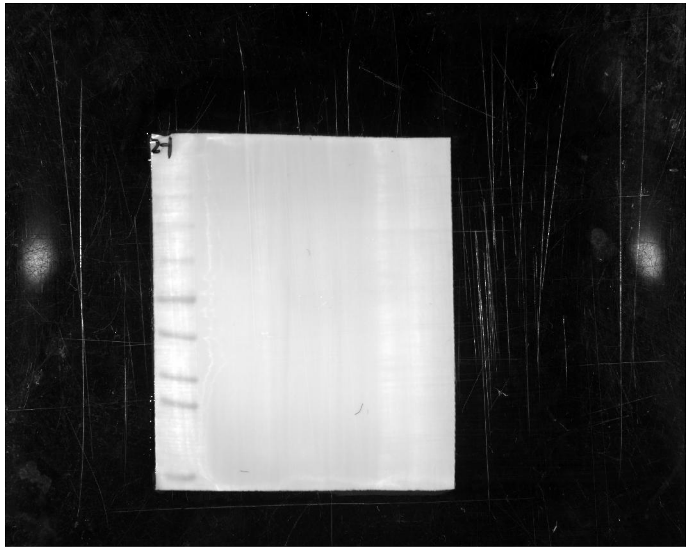

H1299 CDK6

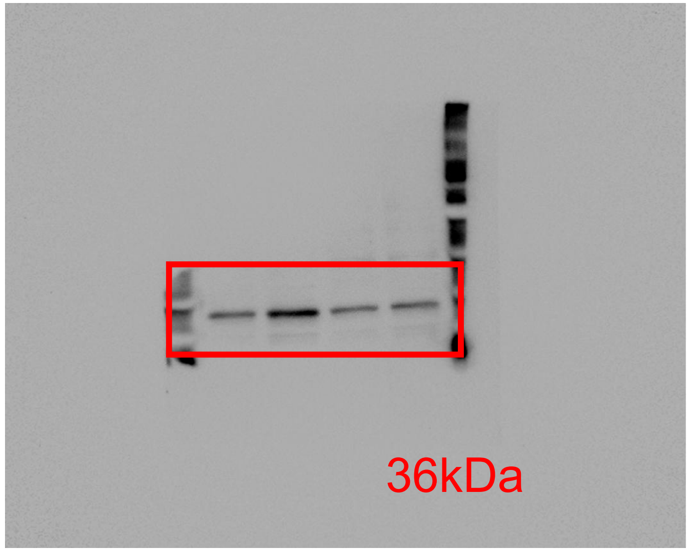

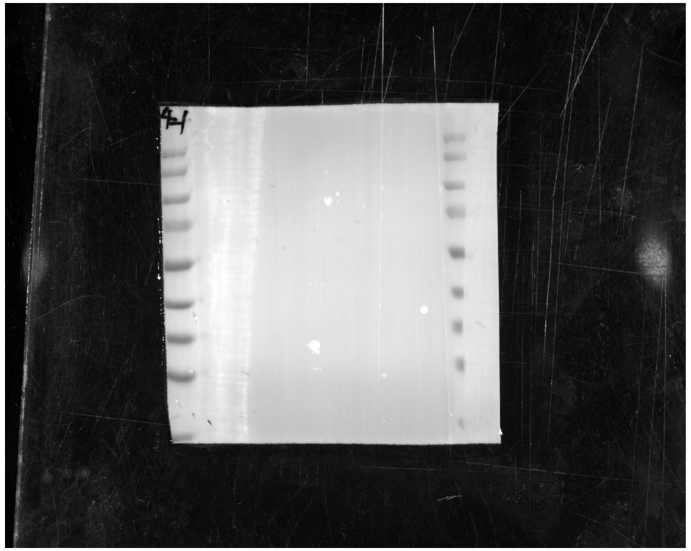

A549 d1

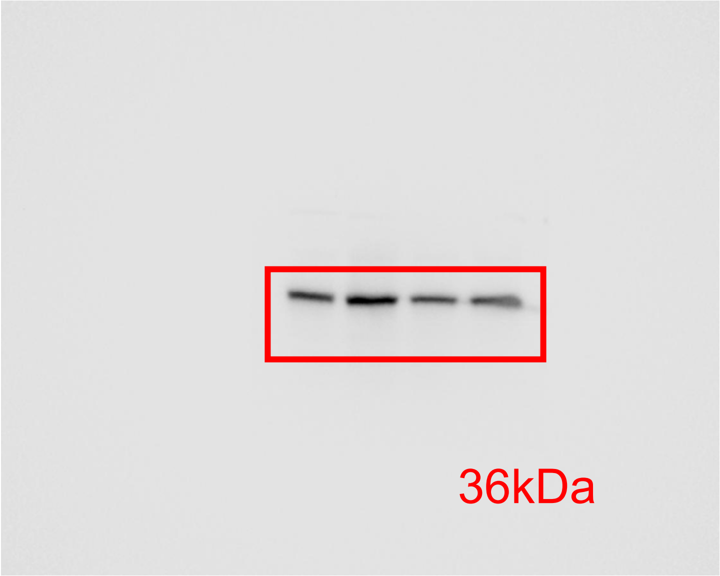

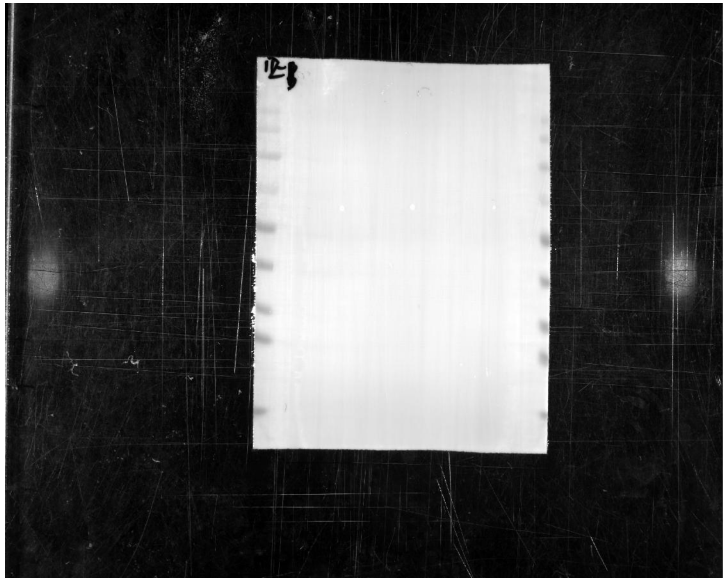

H1299 d1

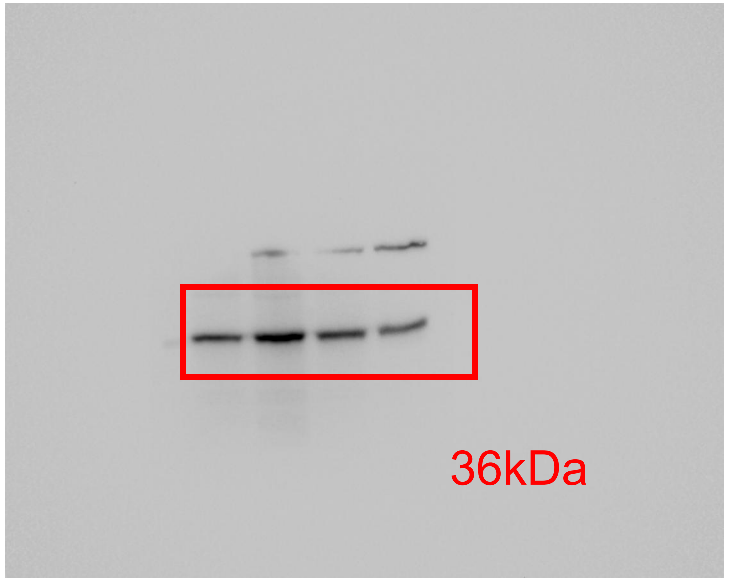

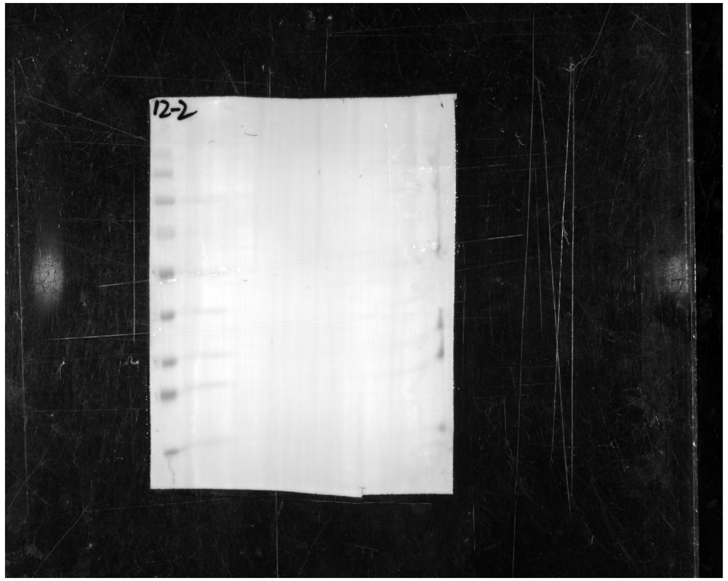

A549 E-ca

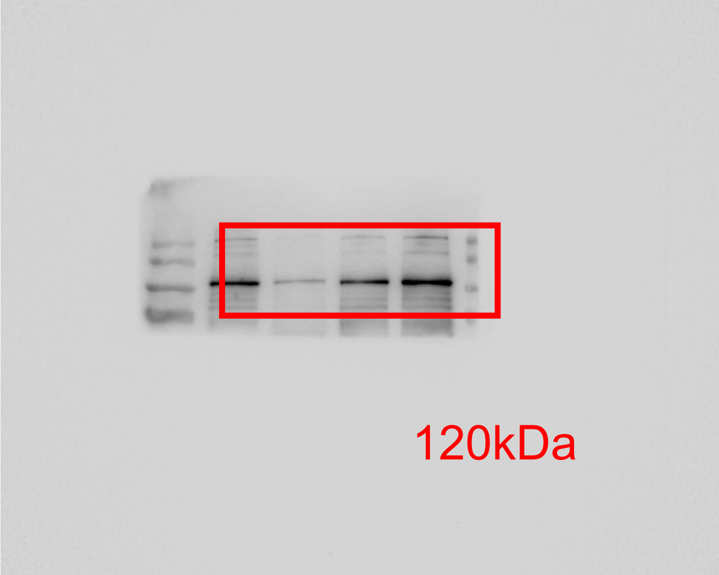

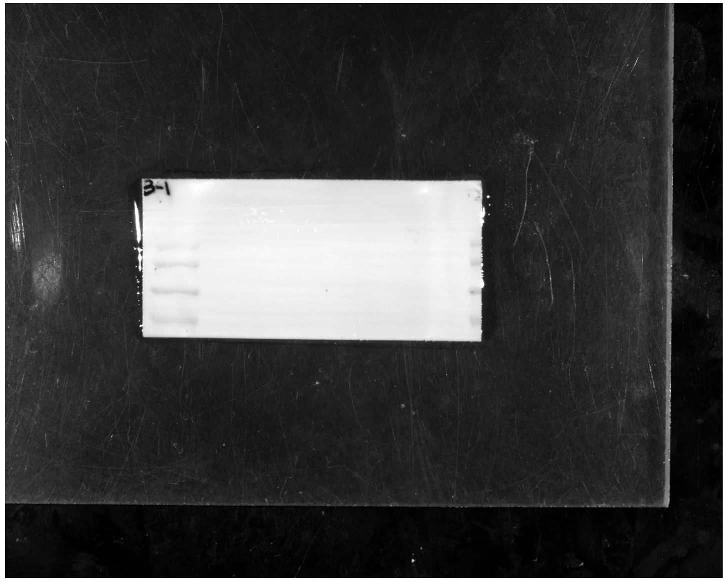

H1299 E-ca

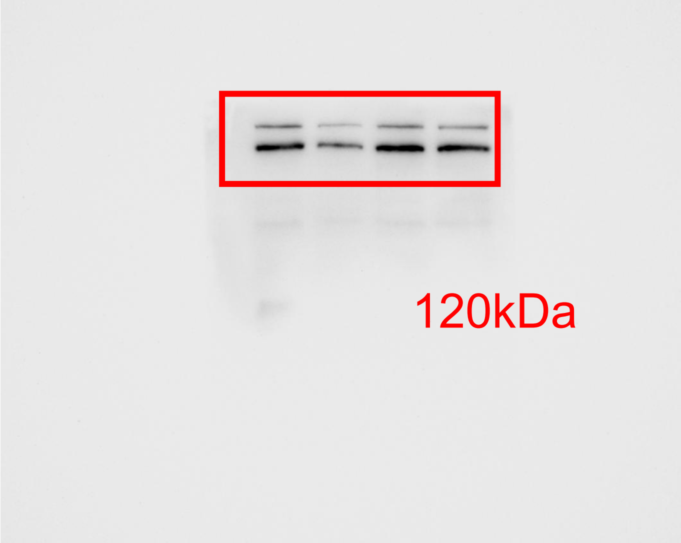

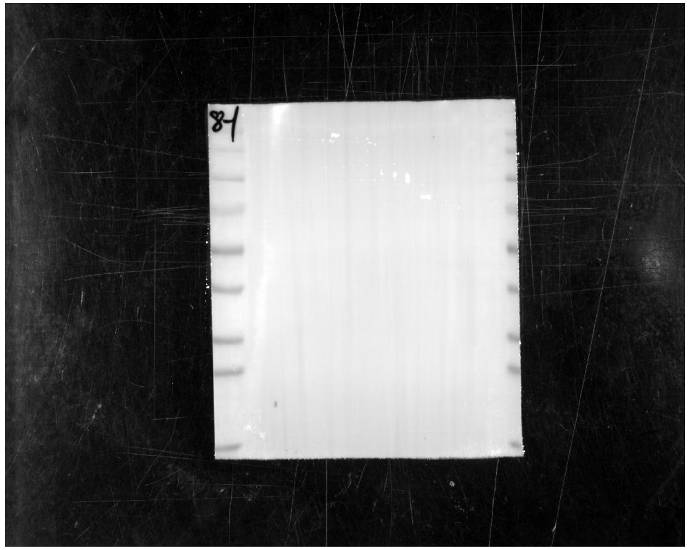

A549 N-ca

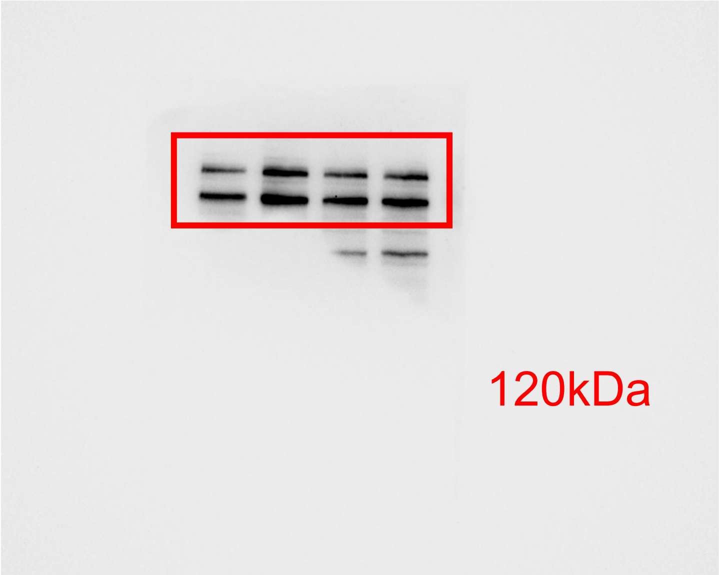

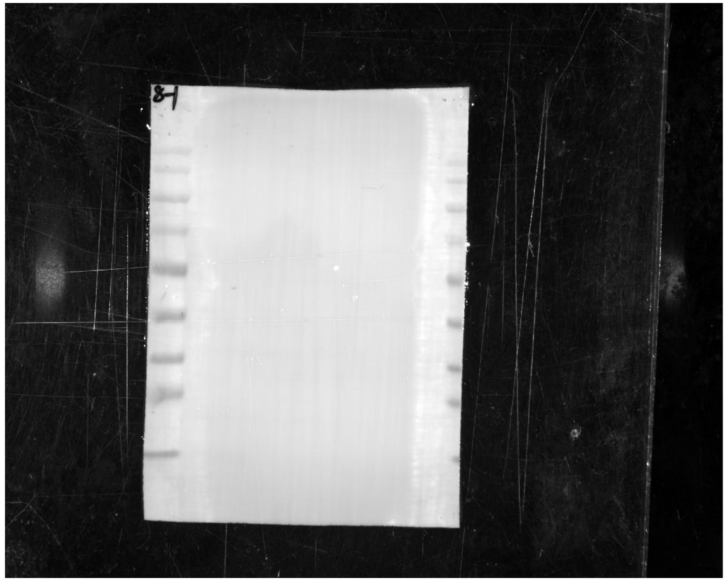

H1299 N-ca

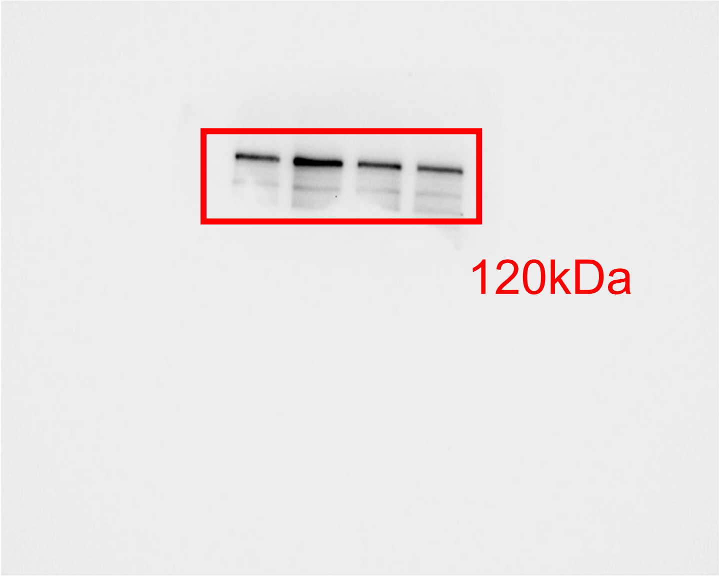

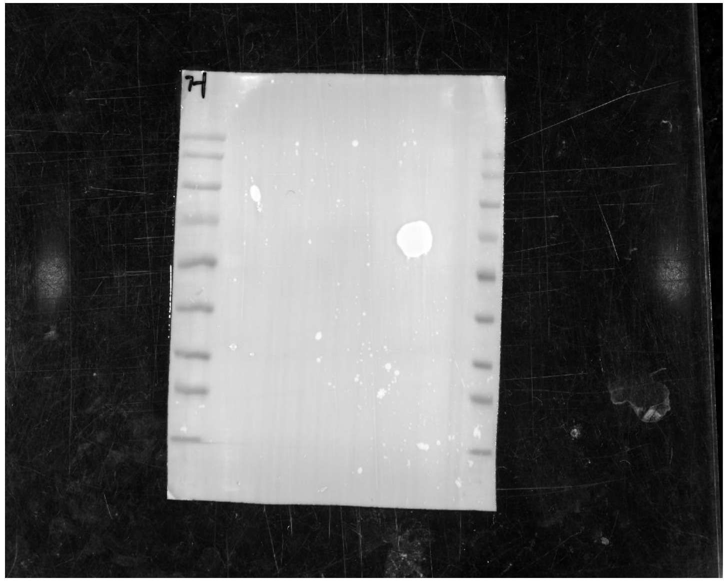

A549 mmp9

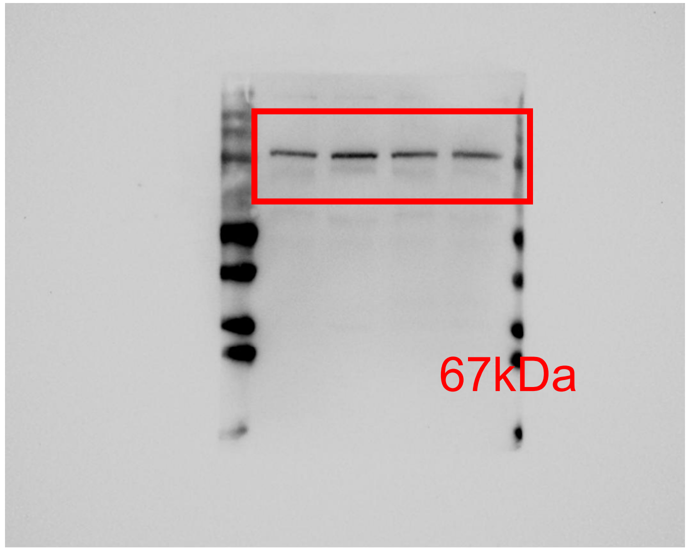

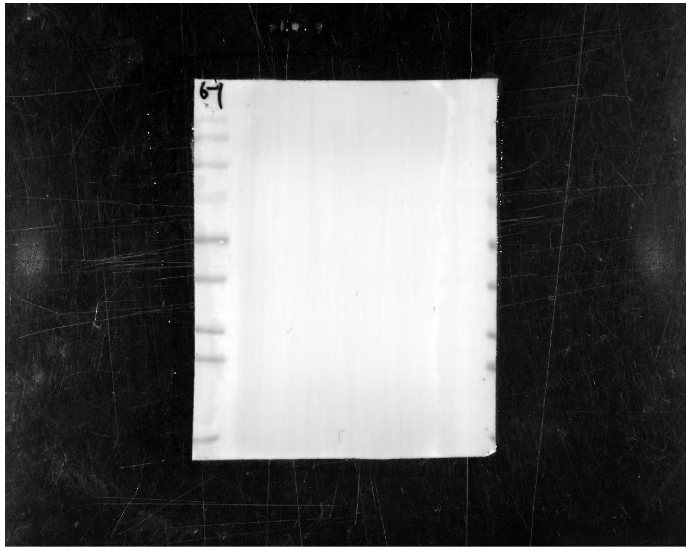

H1299 mmp9

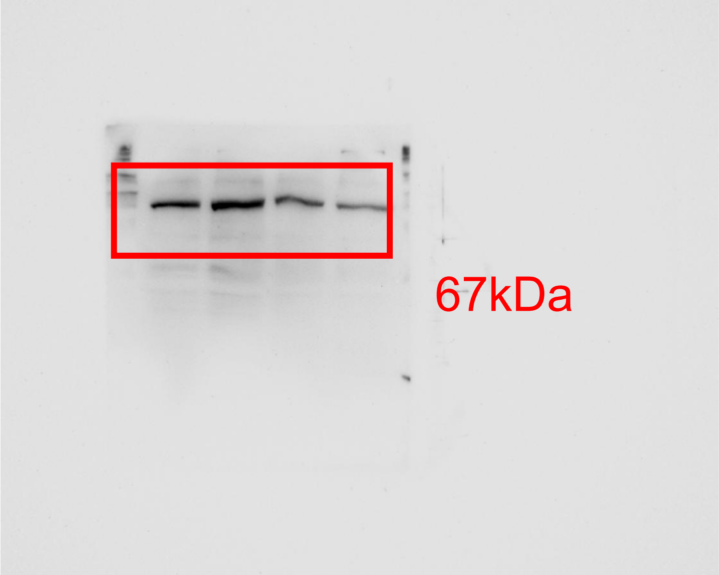

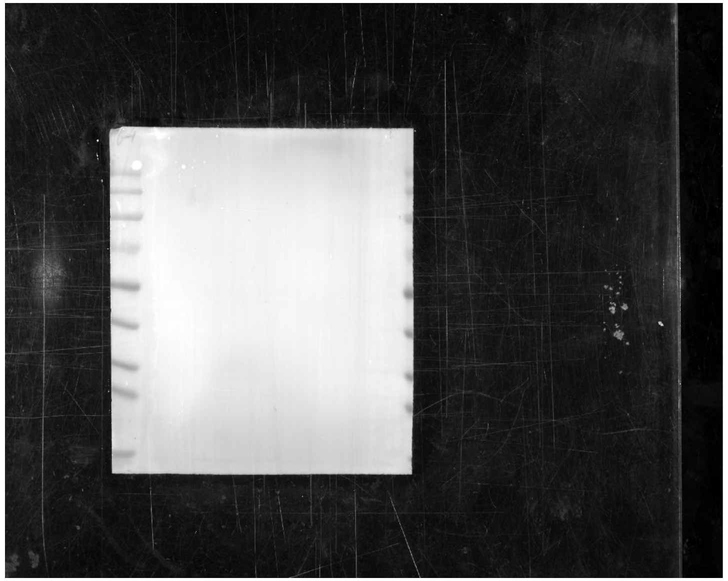

A549 β-actin

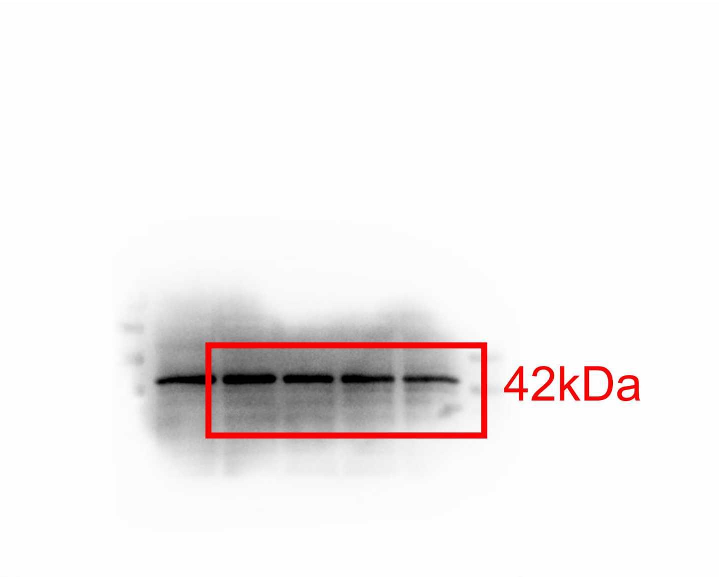

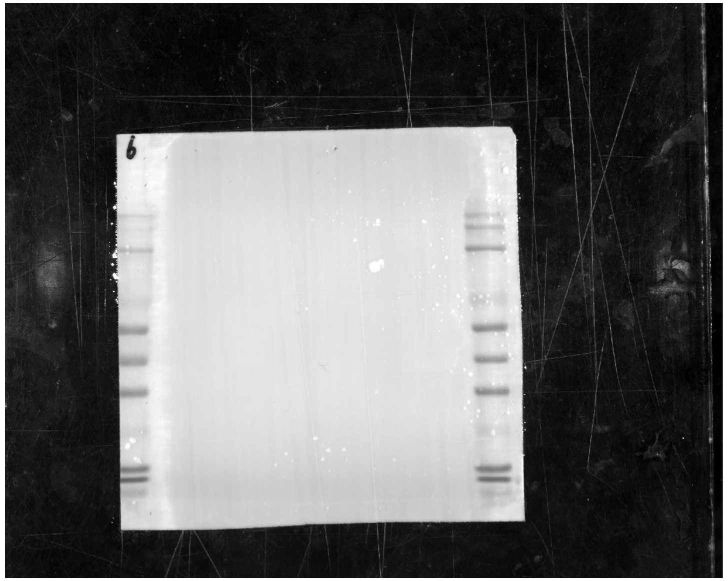

H1299 β-actin

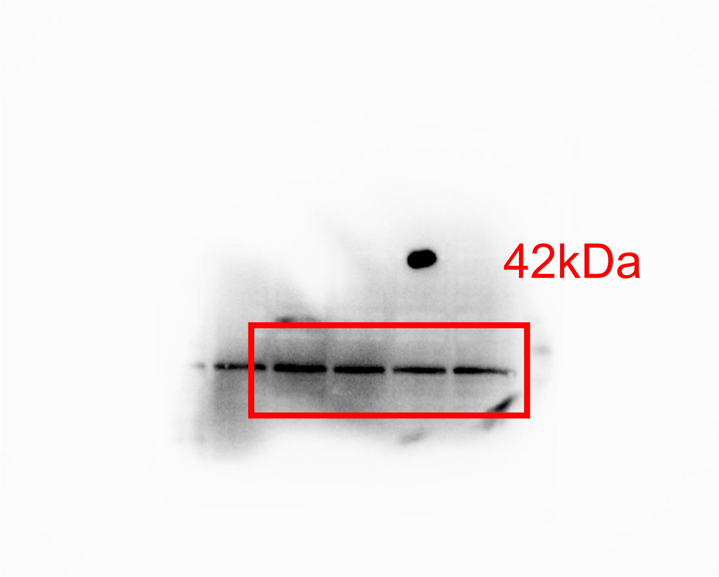

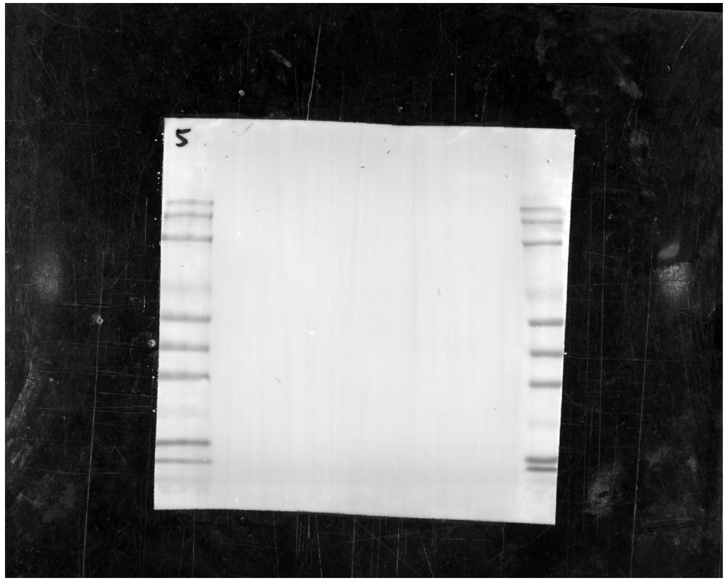

fig4

A549 PTEN

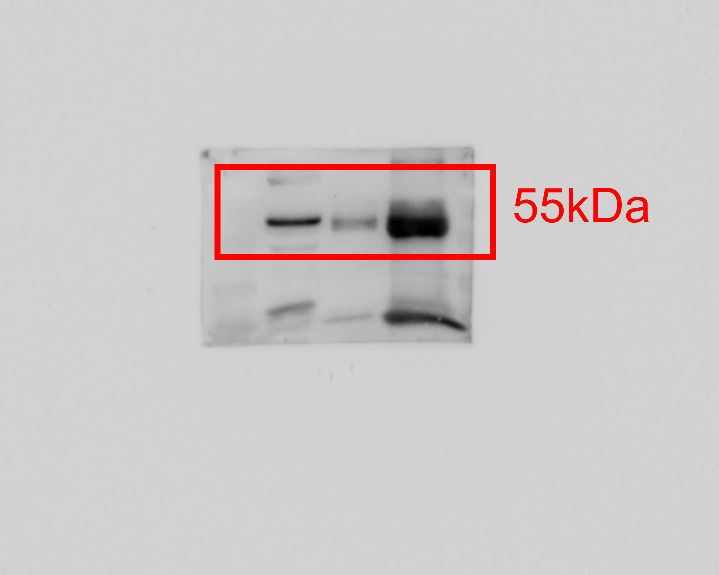

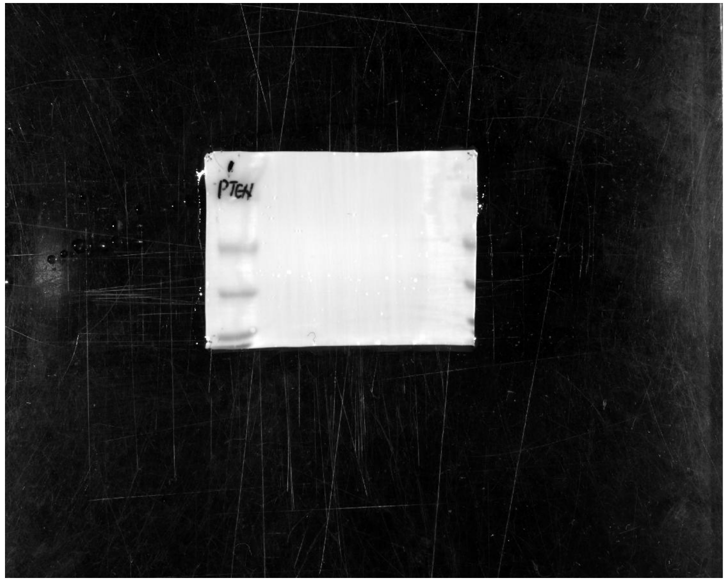

A549 ZC3H15

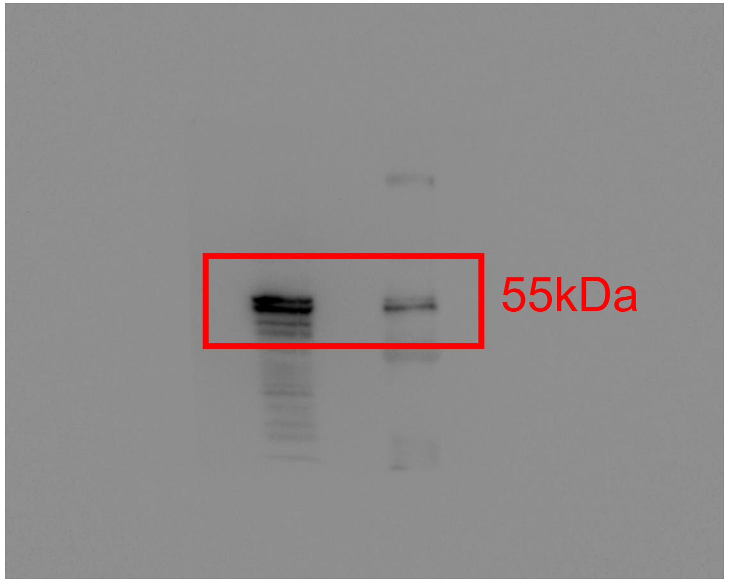

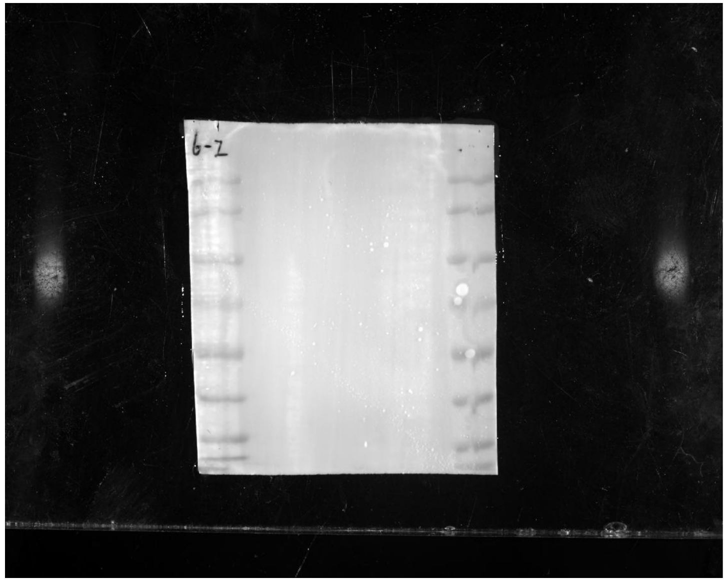

H1299 PTEN IP

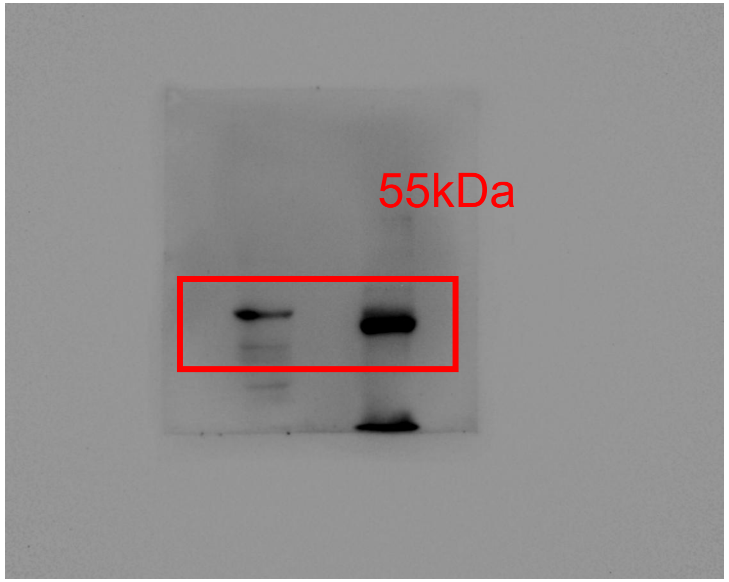

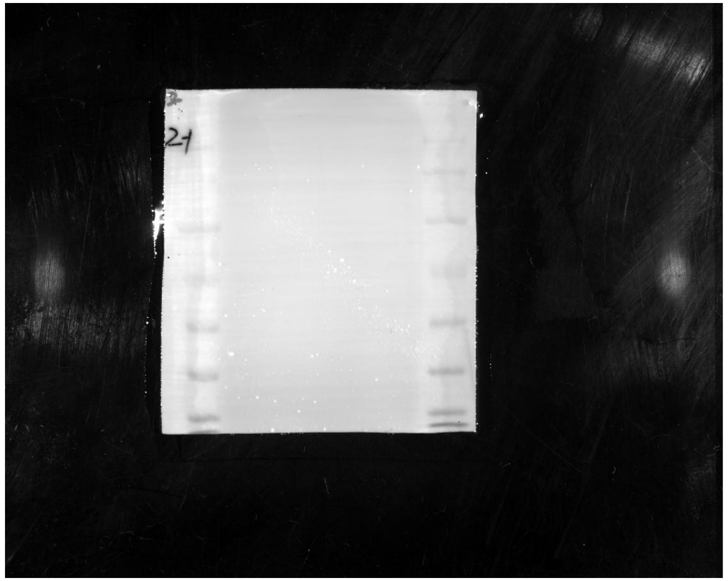

H1299 ZC3H15 IB

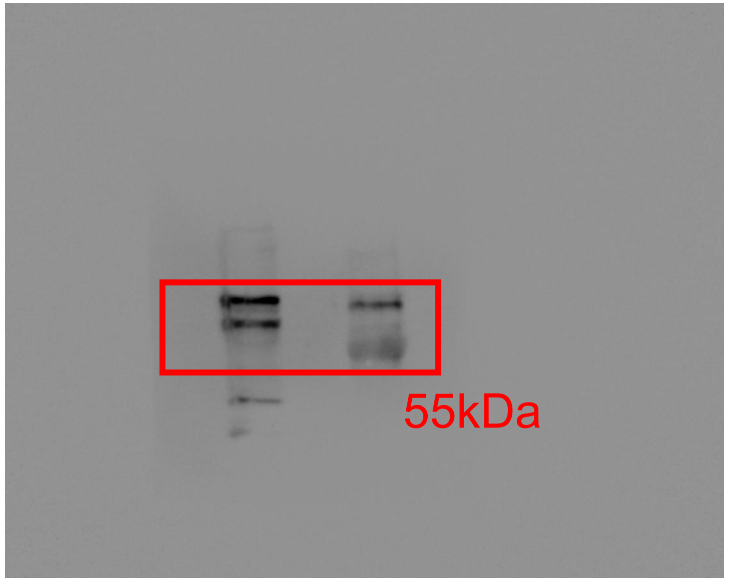

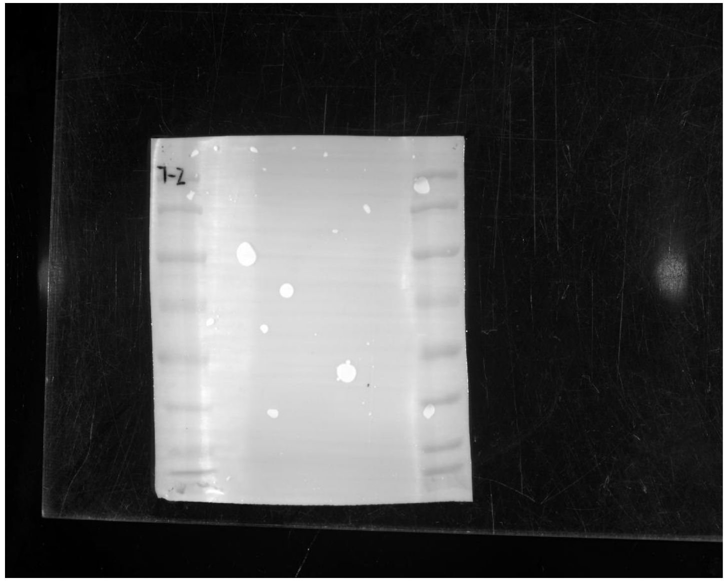

A549 PTEN IB

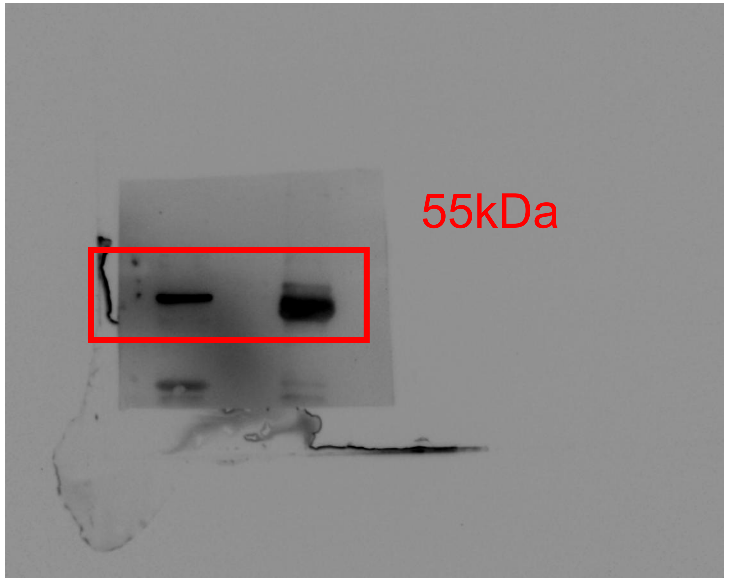

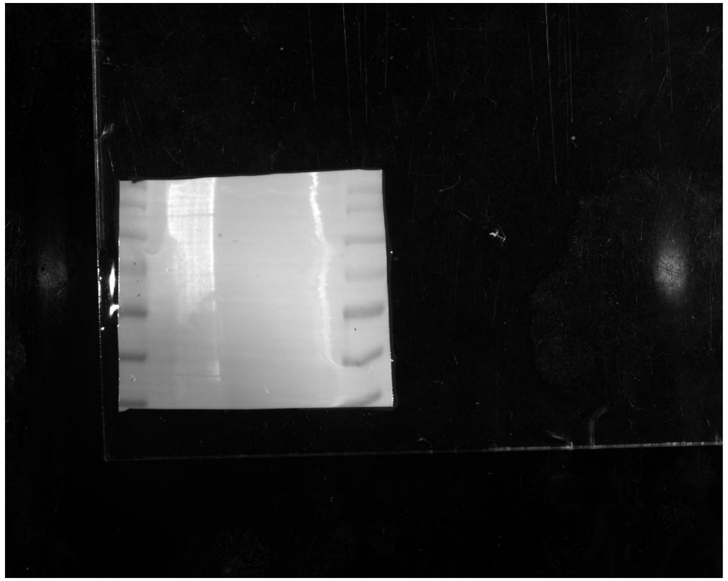

A549 ZC3H15
IP

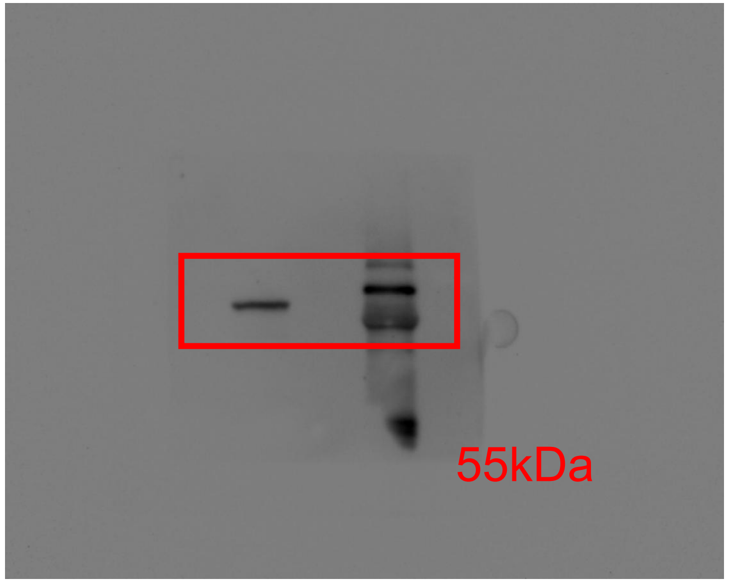

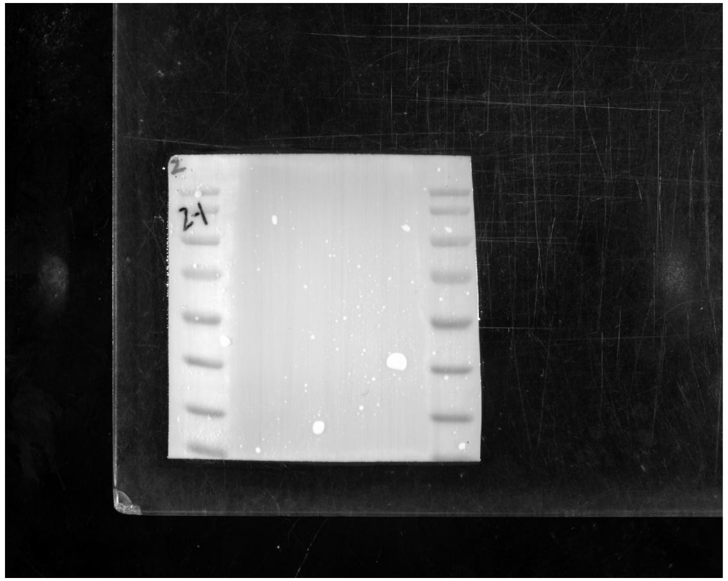

H1299 PTEN IB

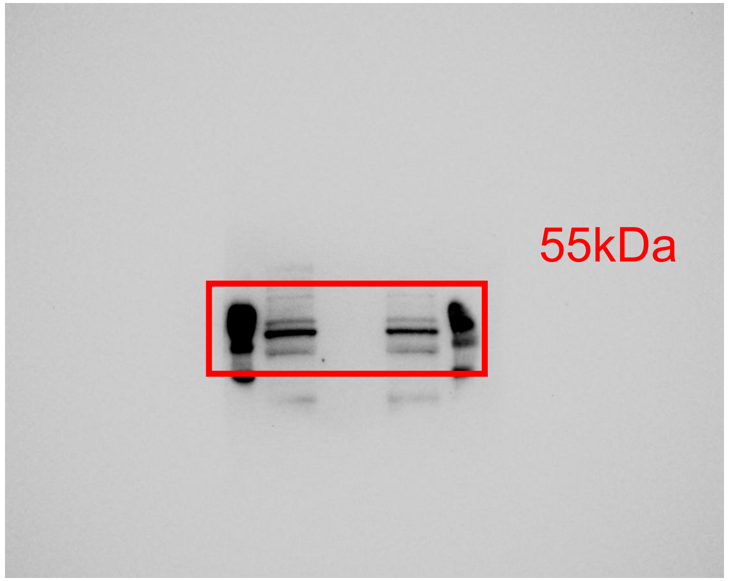

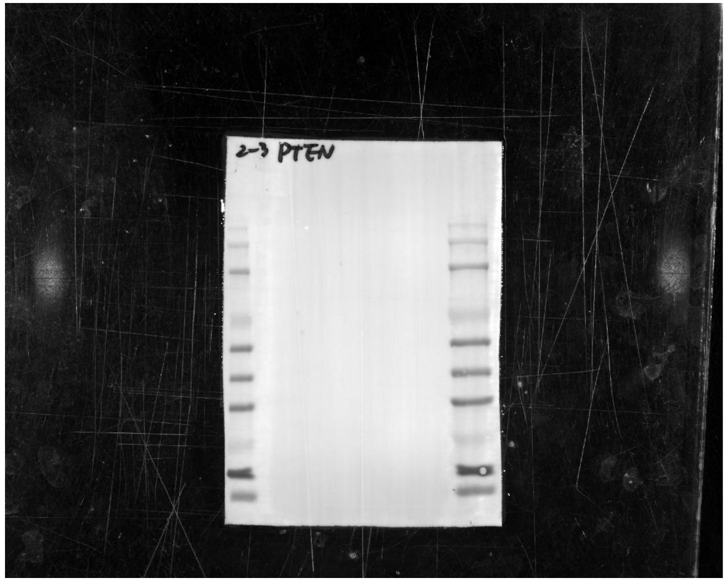

H1299 ZC3H15 IP

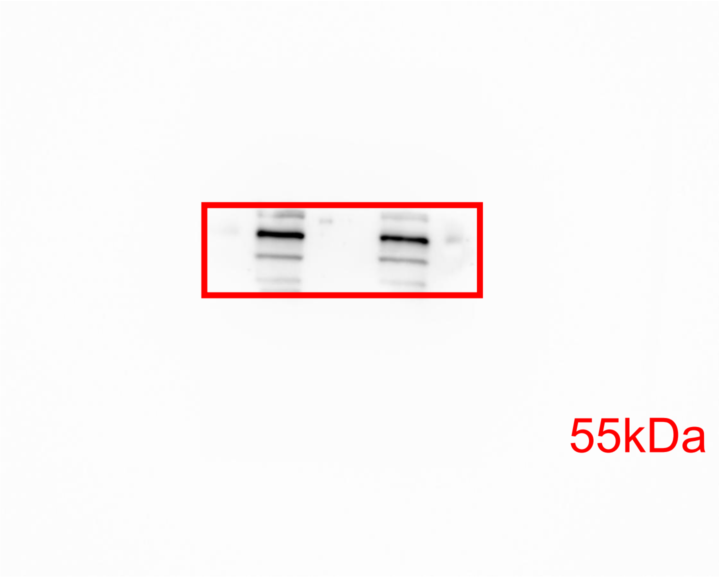

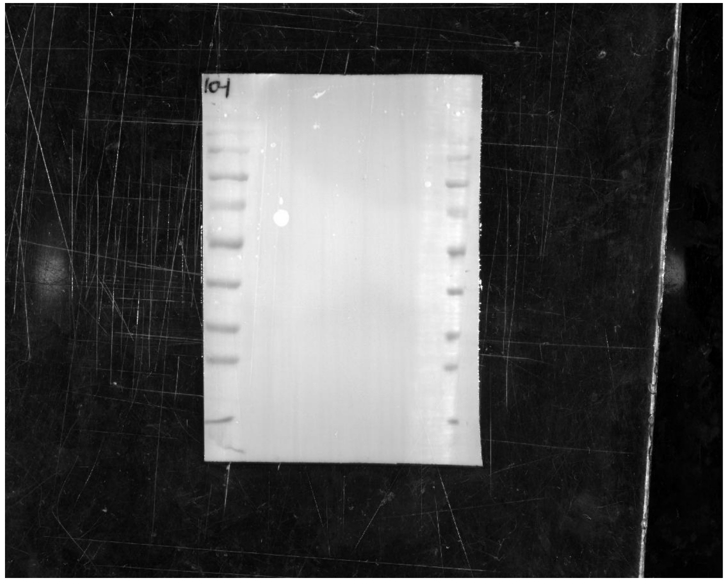

S-fig5

A549 ZC3H15

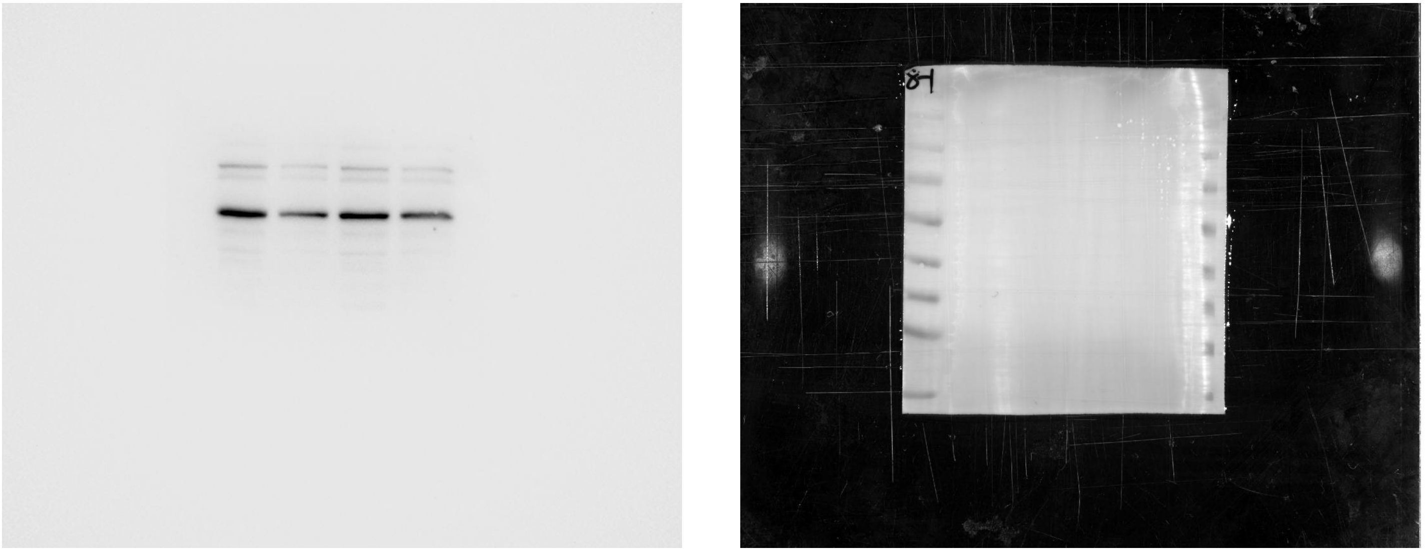

H1299 ZC3H15

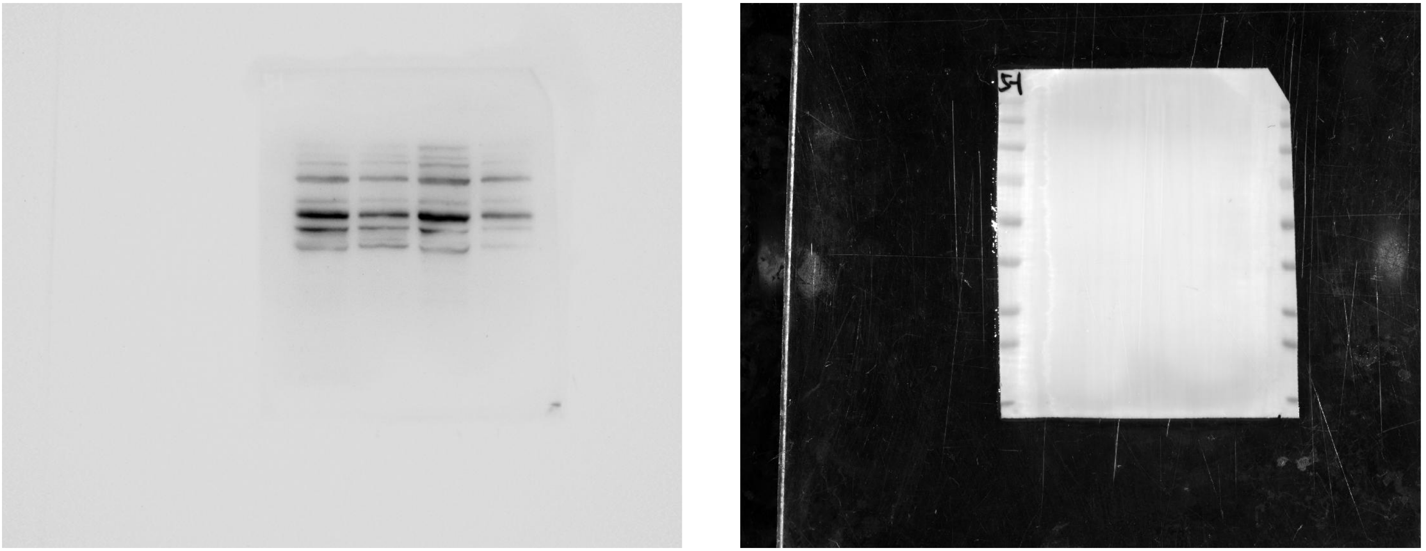

A549 p-akt

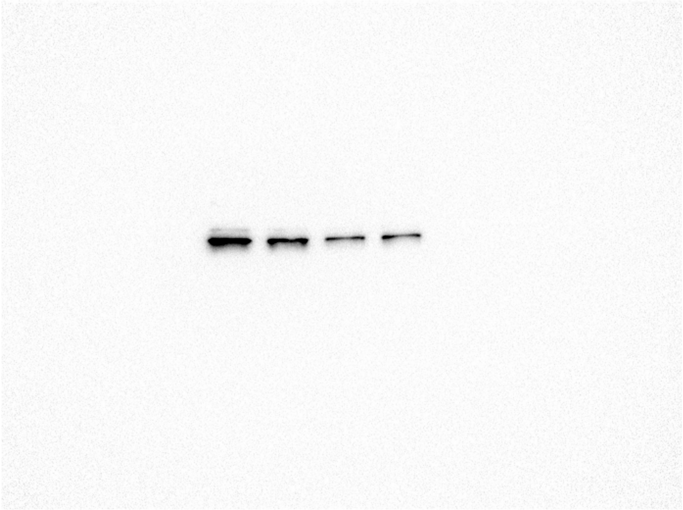

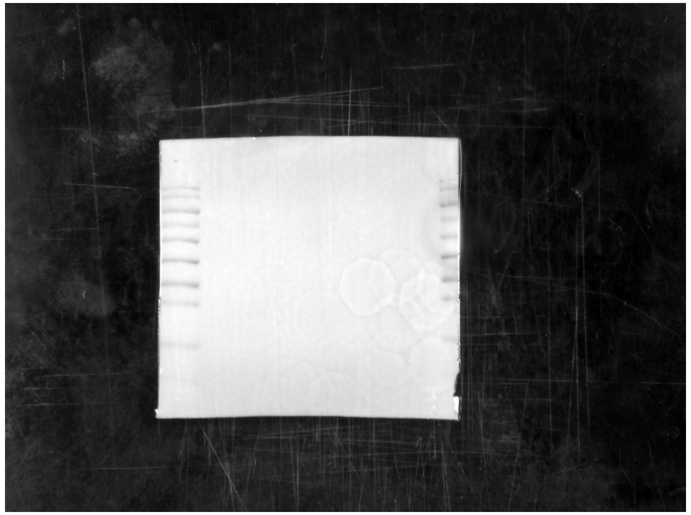

H1299 p-akt

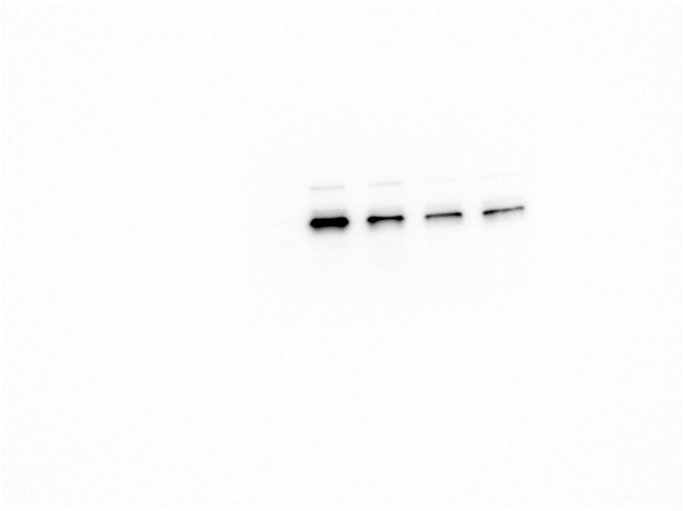

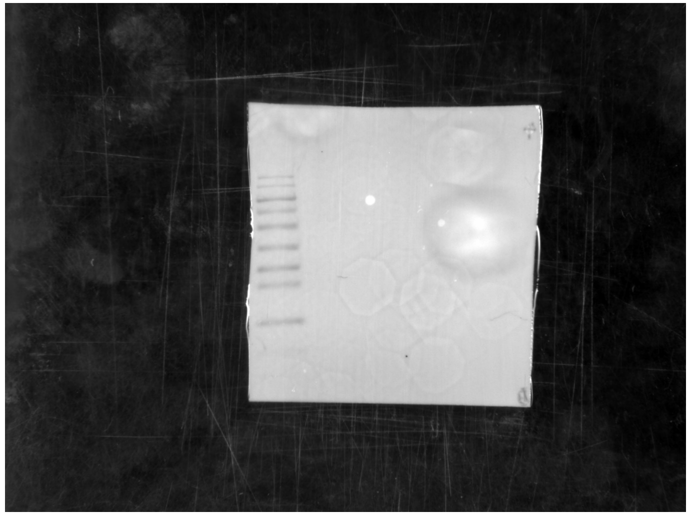

A549 akt

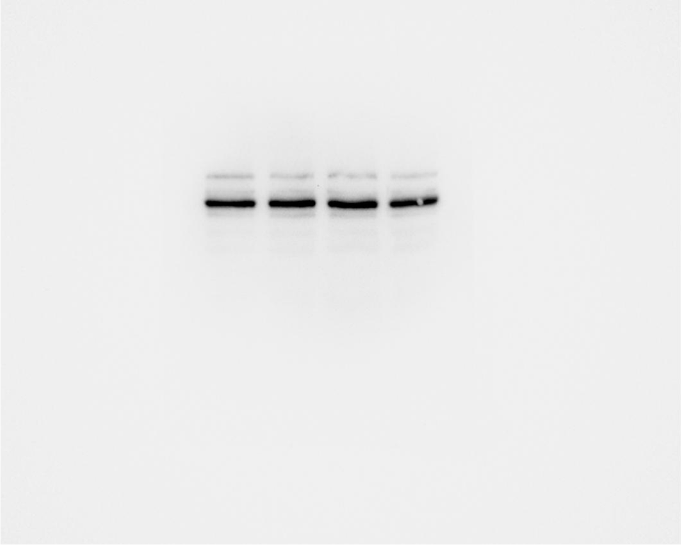

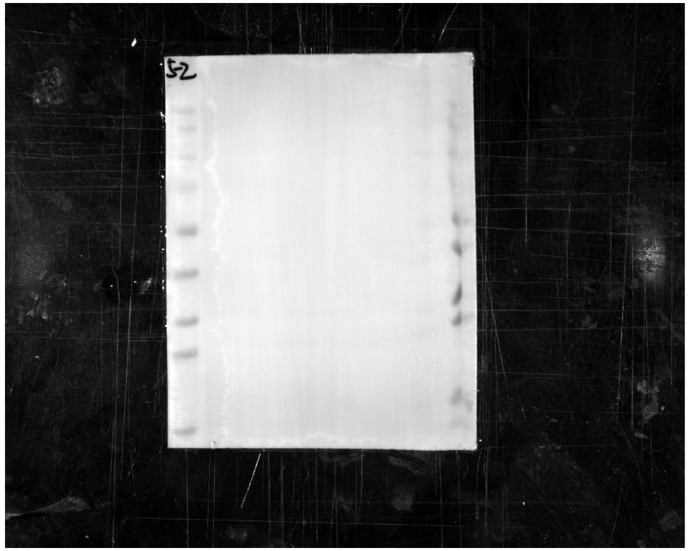

H1299 akt

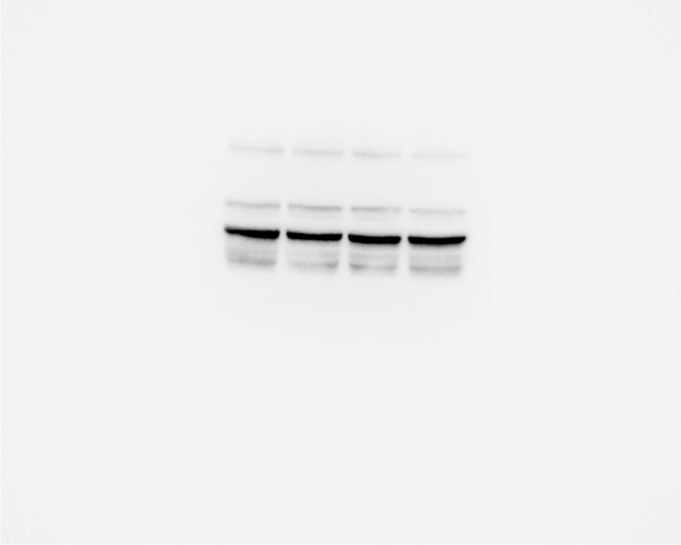

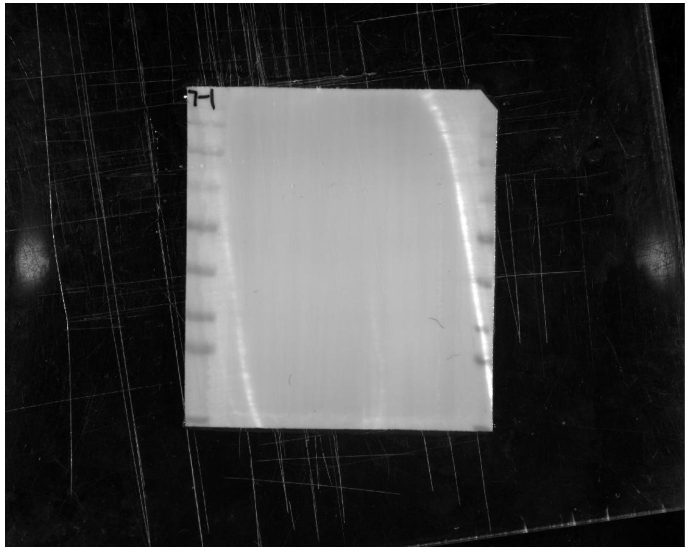

A549 p-mtor

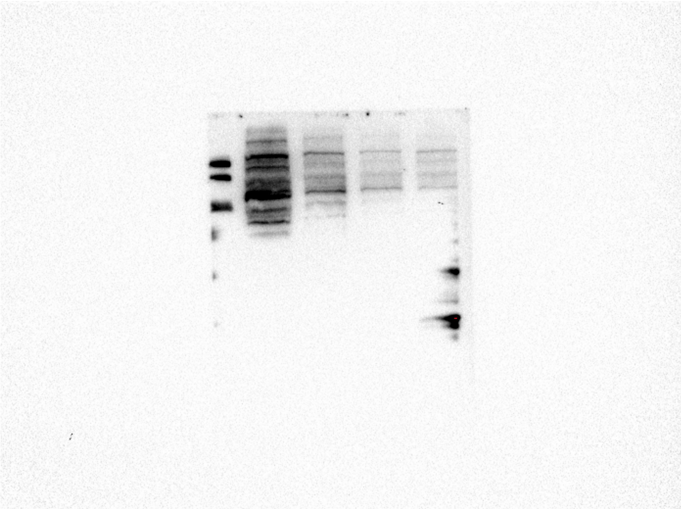

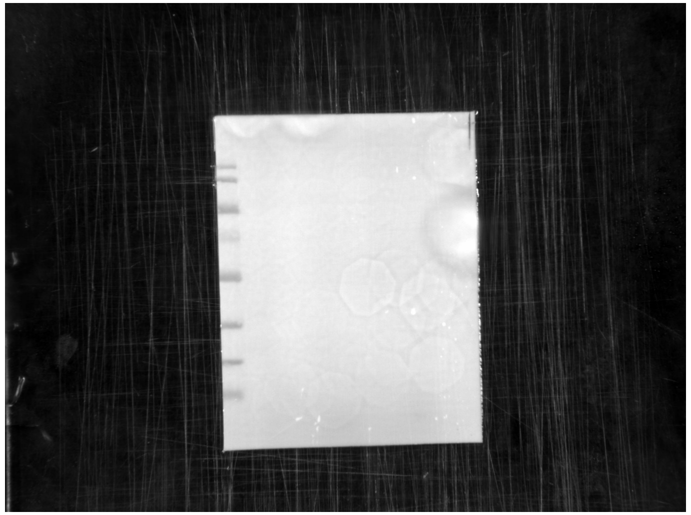

H1299 p-mtor

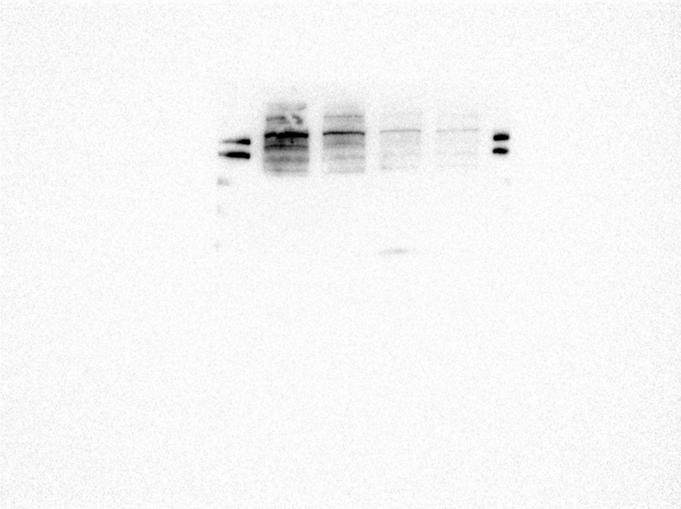

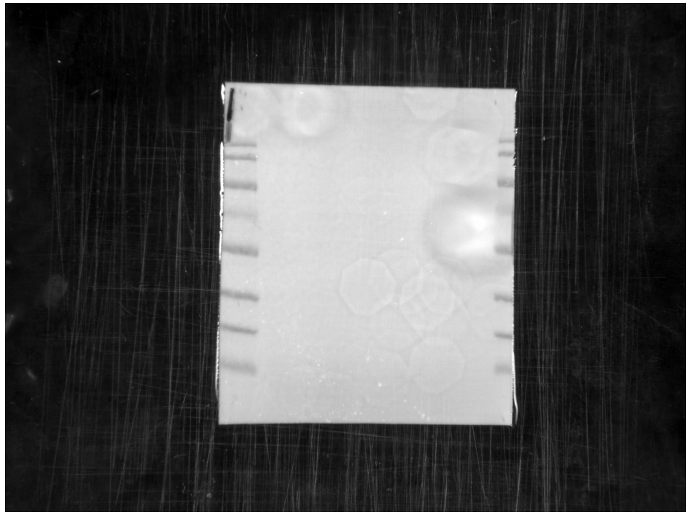

A549 mtor

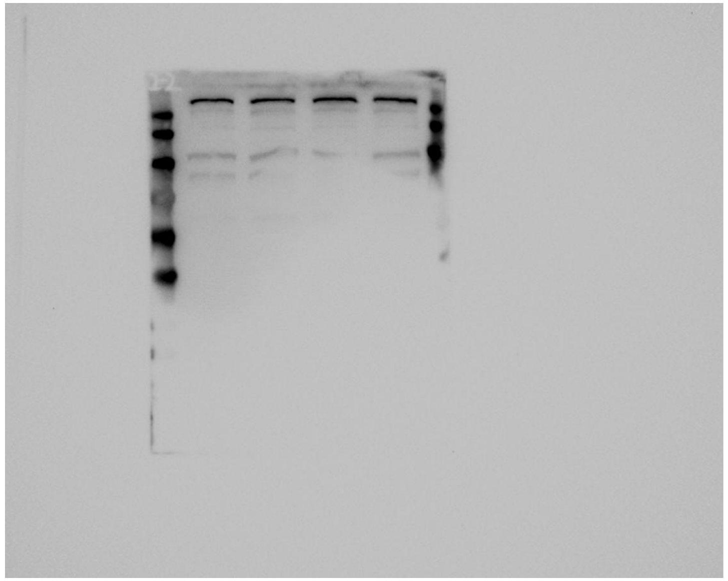

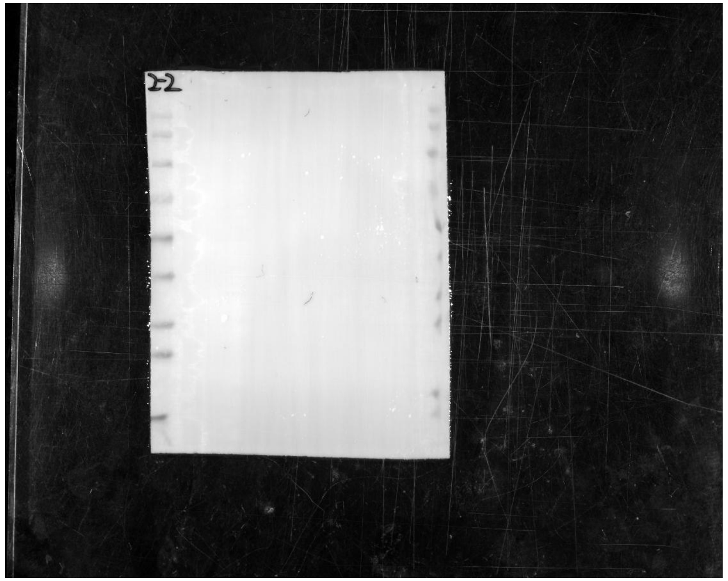

H1299 mtor

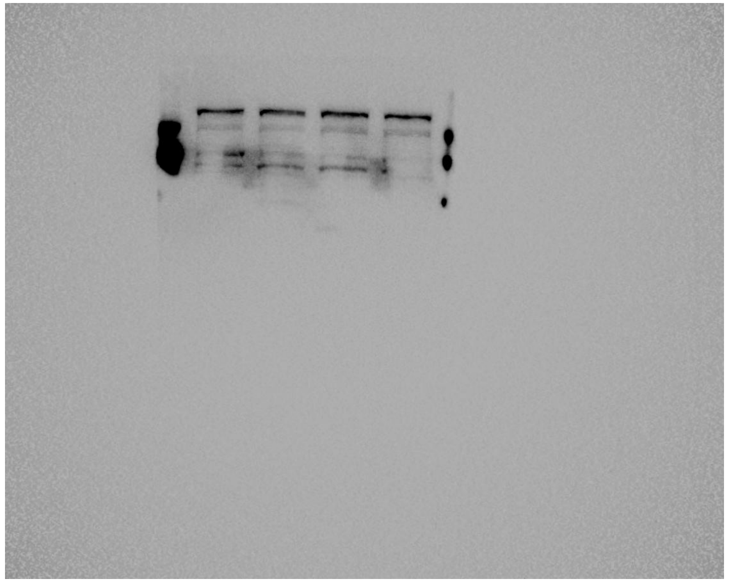

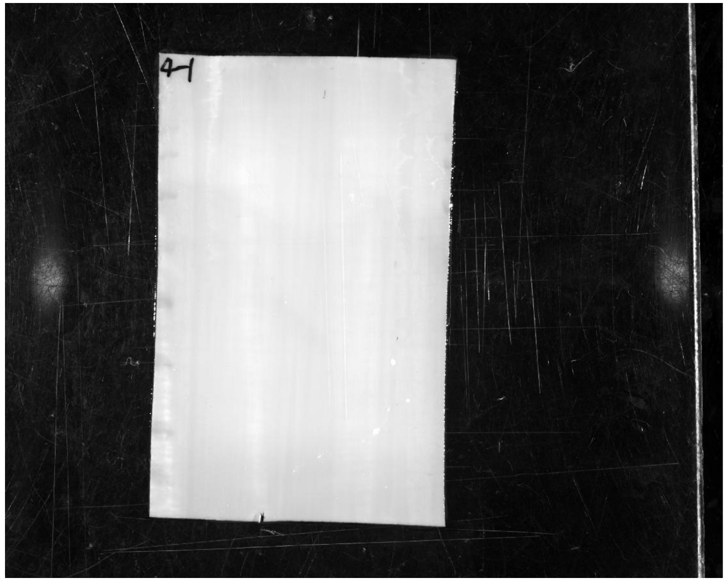

A549 β-actin

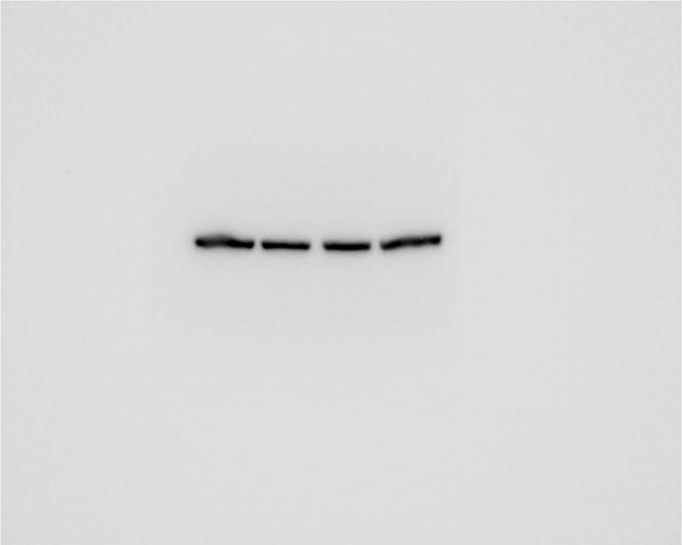

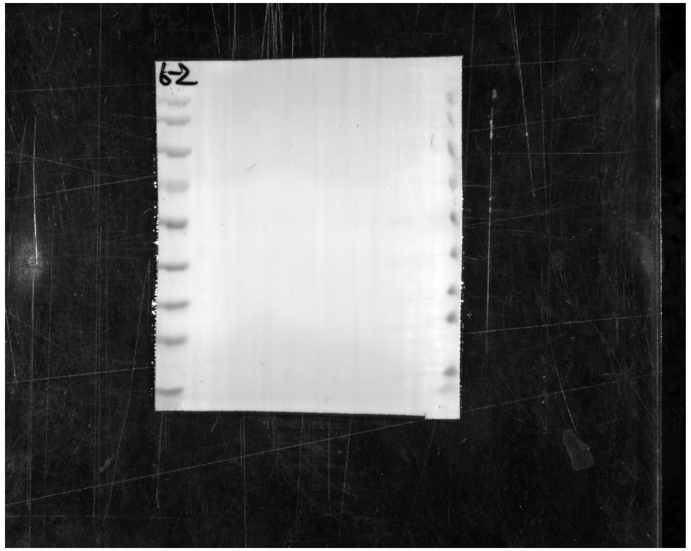

H1299 β-actin

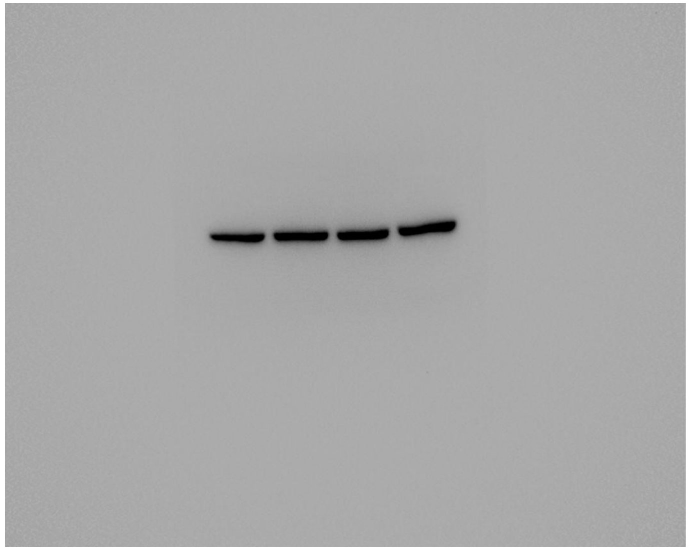

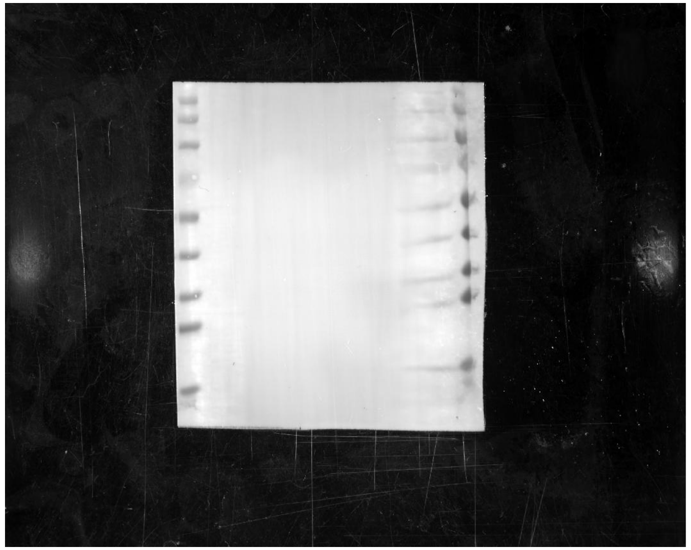

A549 CDK4

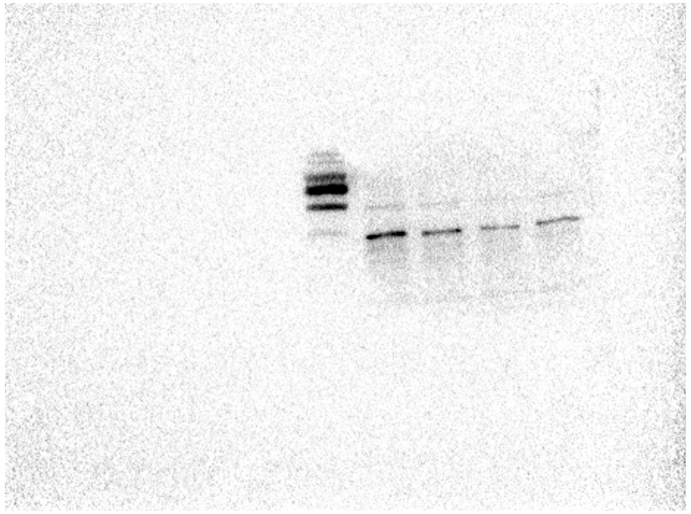

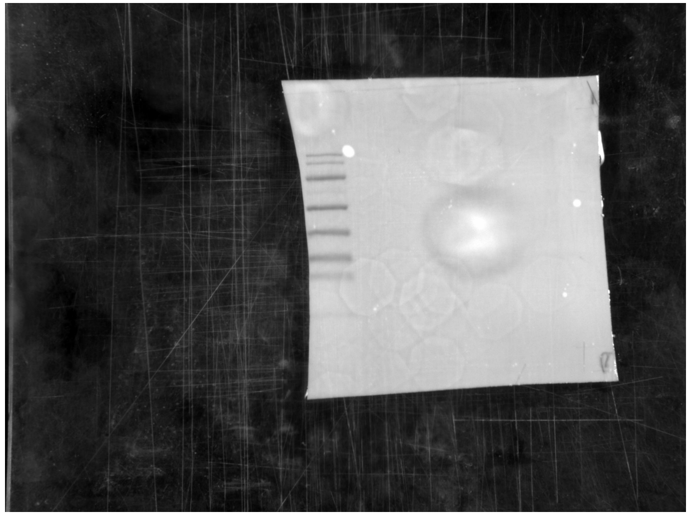

H1299 CDK4

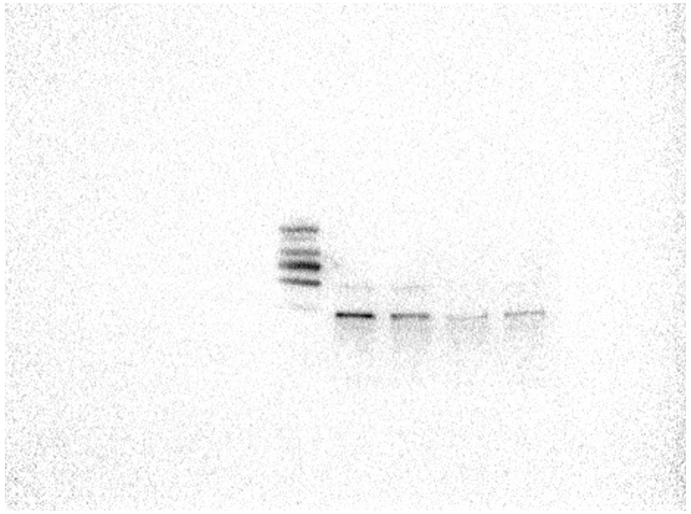

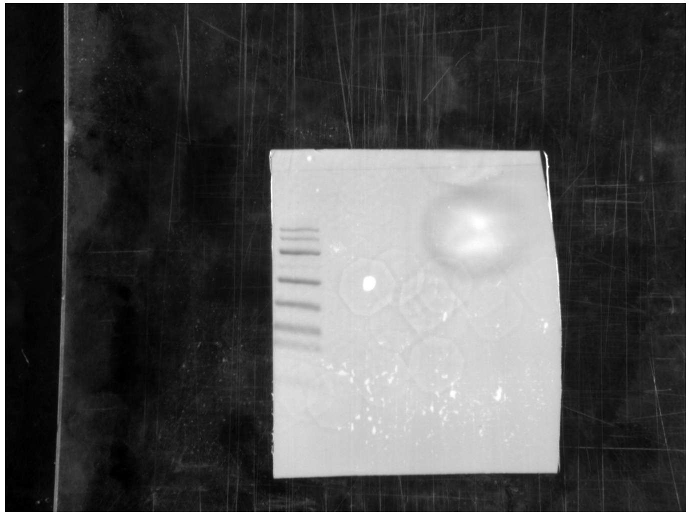

A549 CDK6

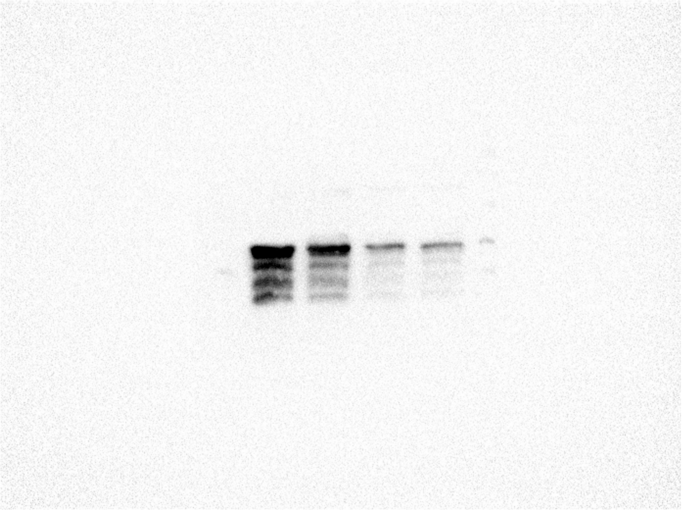

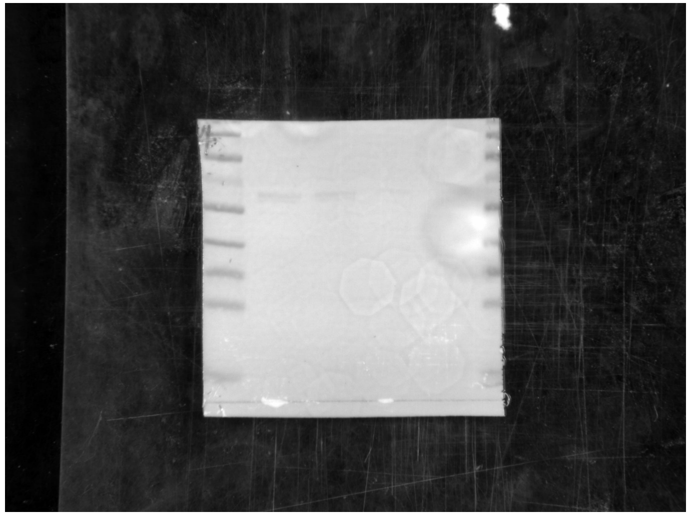

H1299 CDK6

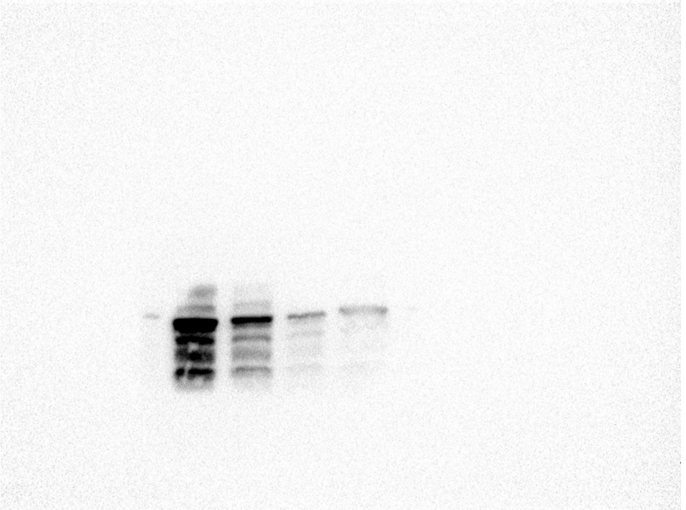

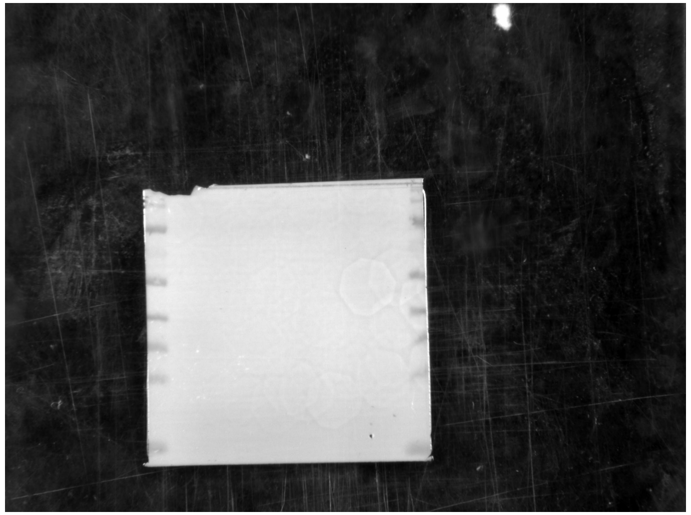

A549 D1

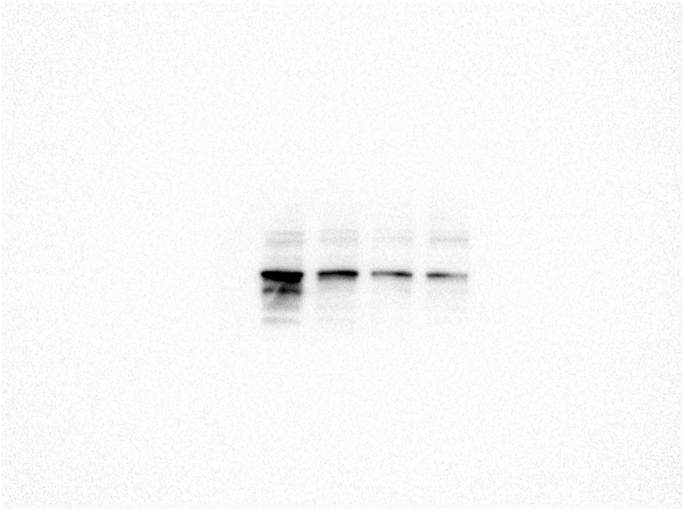

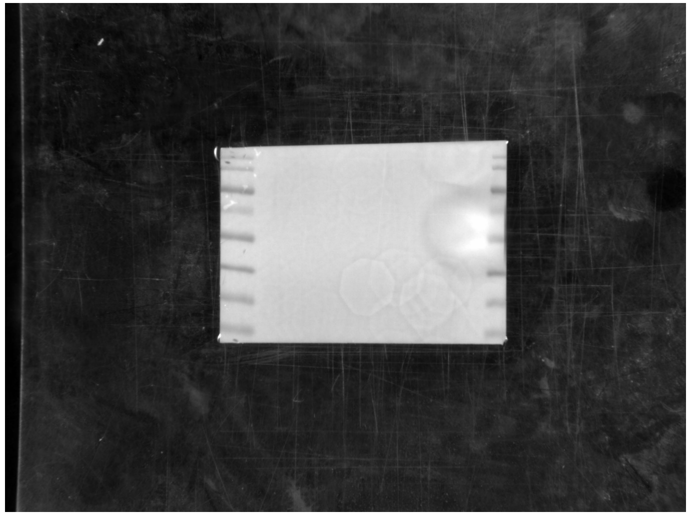

H1299 D1

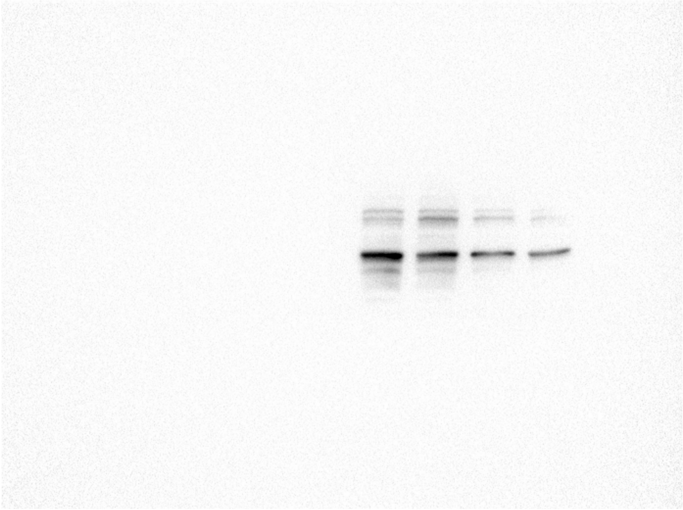

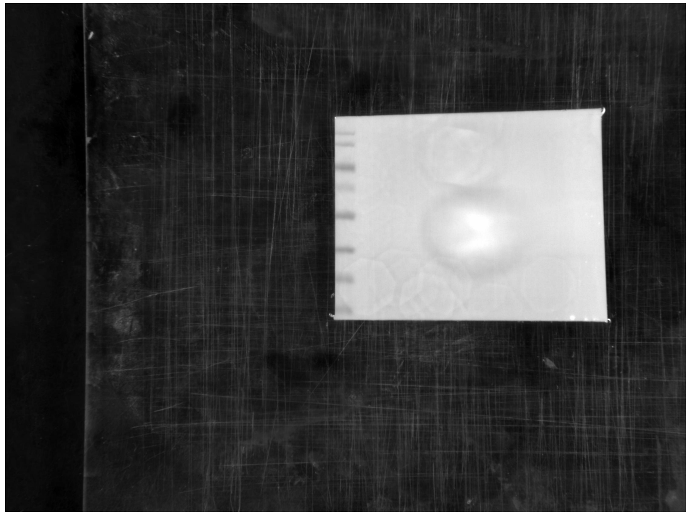

A549 E-CA

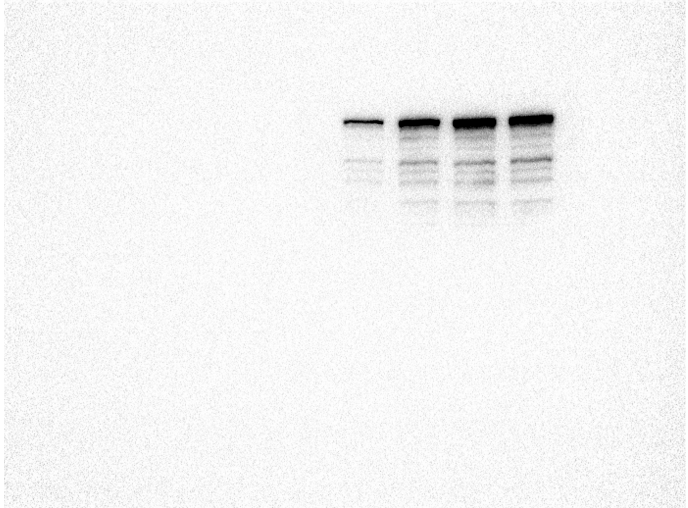

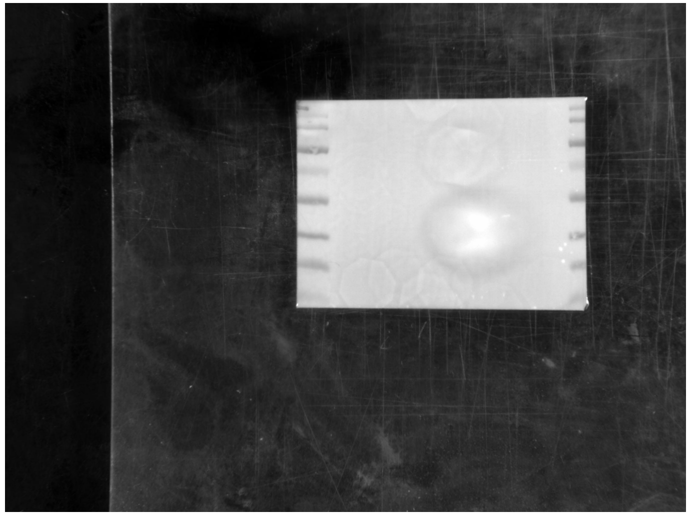

H1299 E-CA

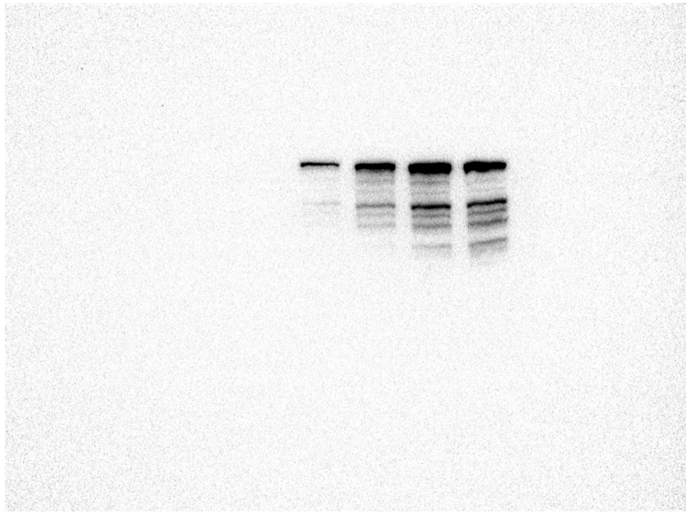

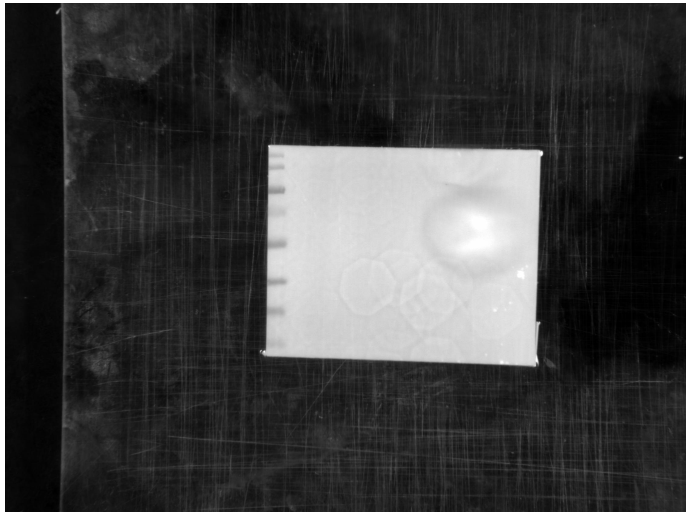

A549 N-CA

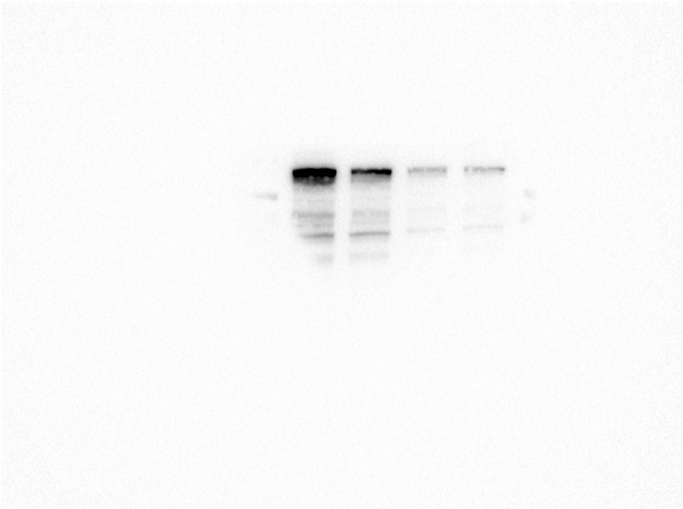

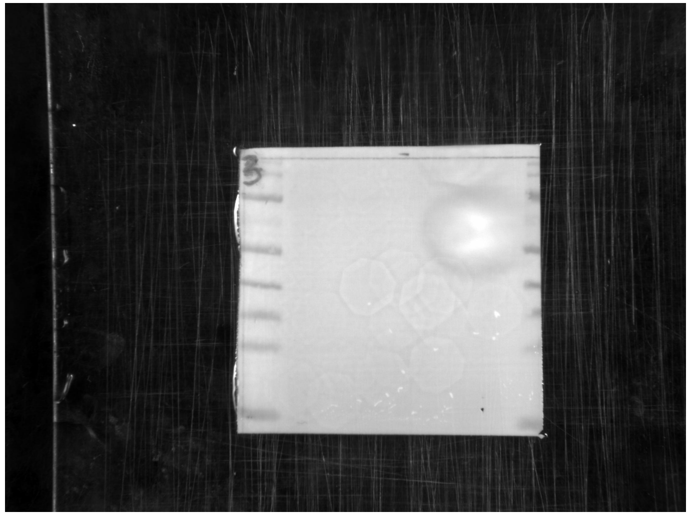

H1299 N-CA

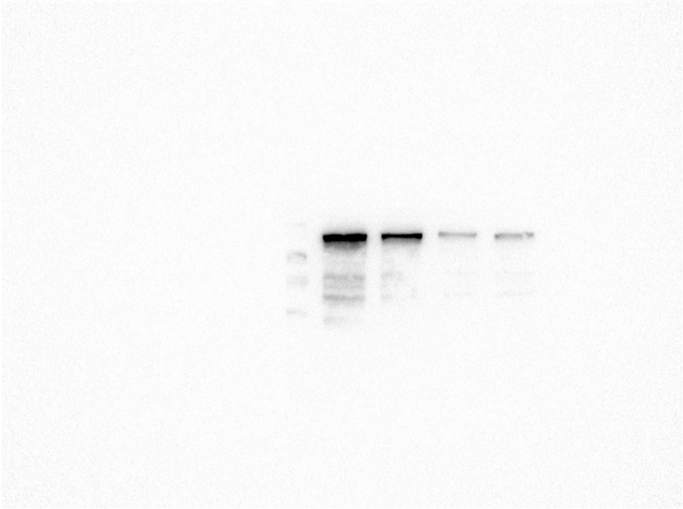

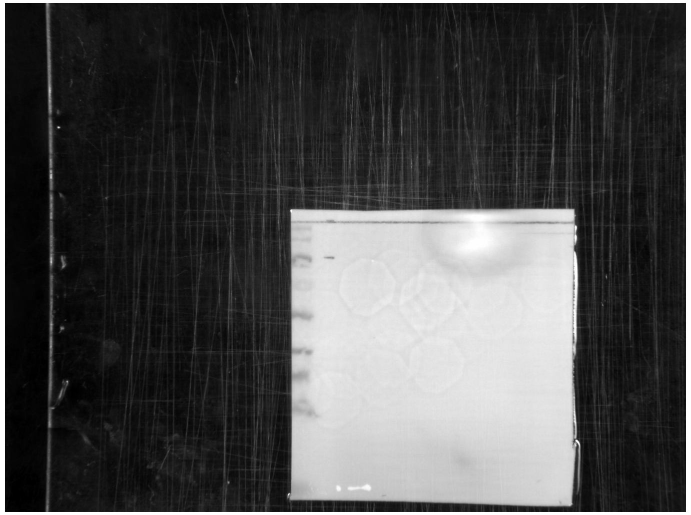

A549 MMP9

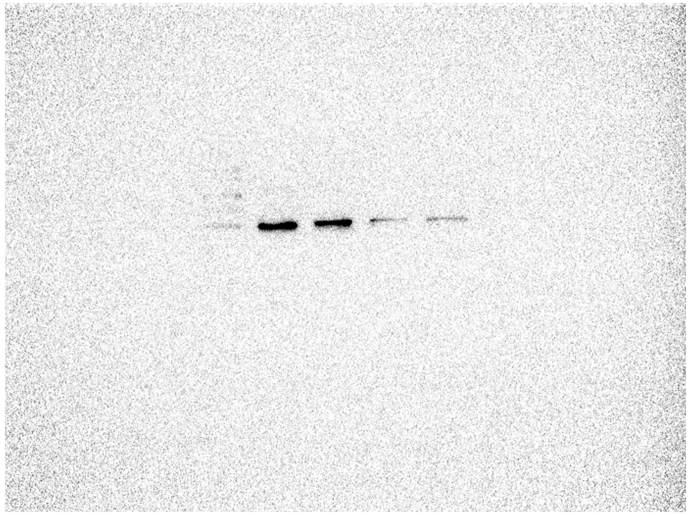

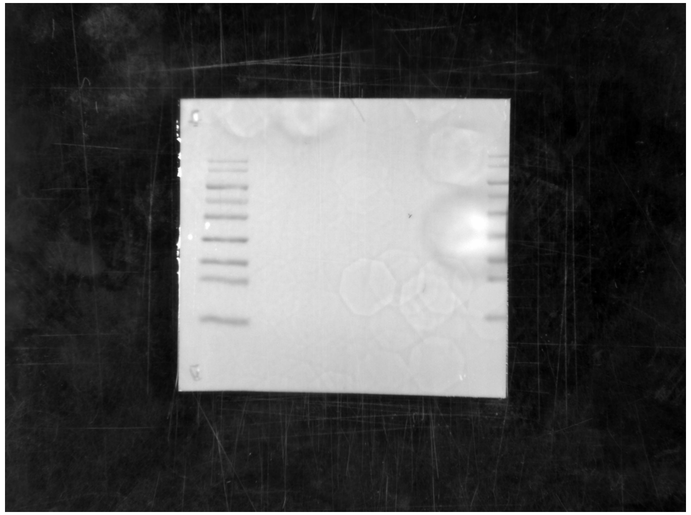

H1299 MMP9

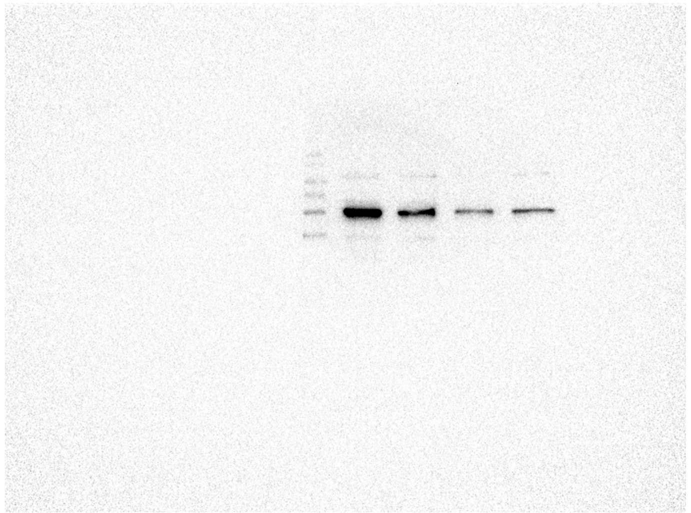

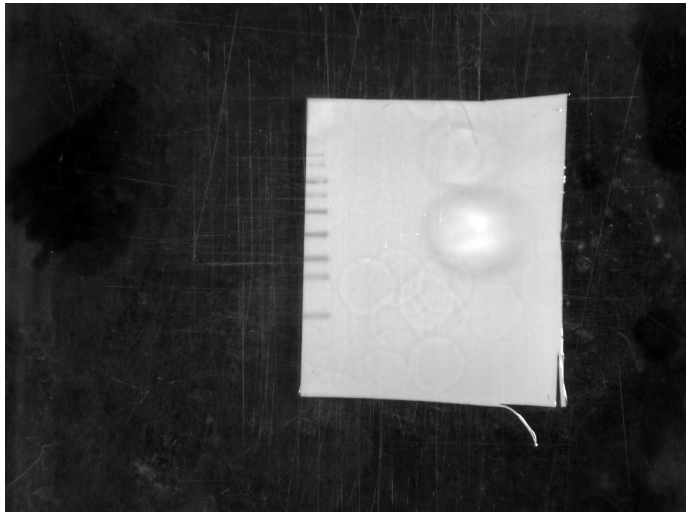

A549 β-actin

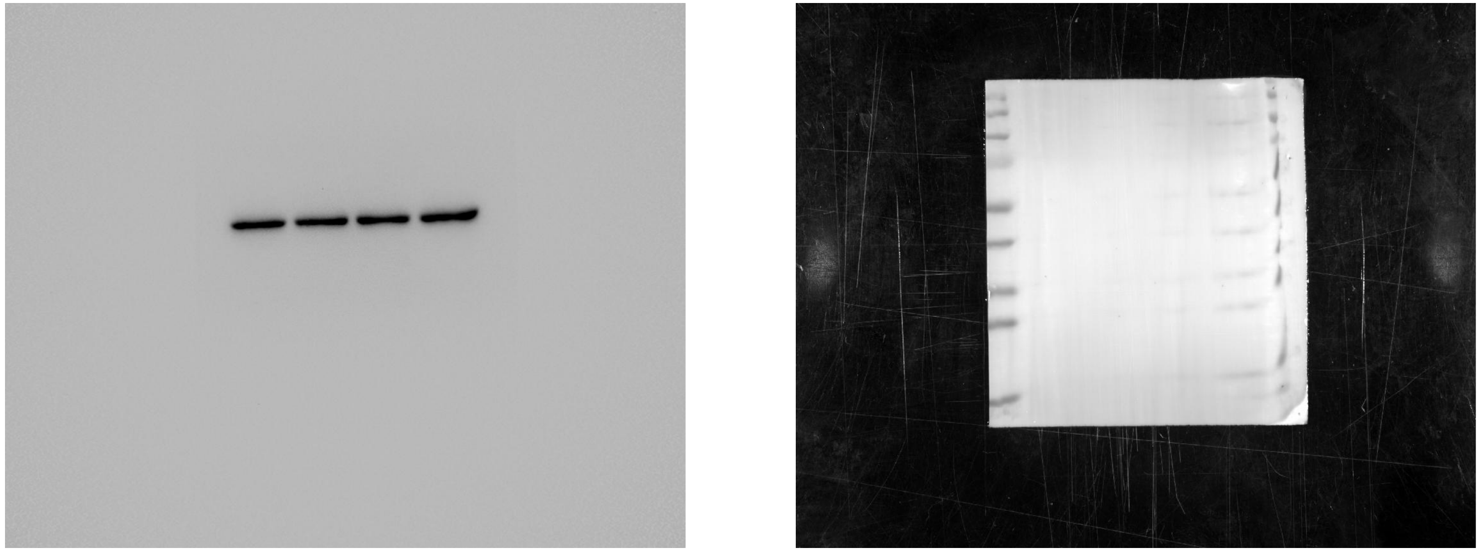

H1299 β-actin

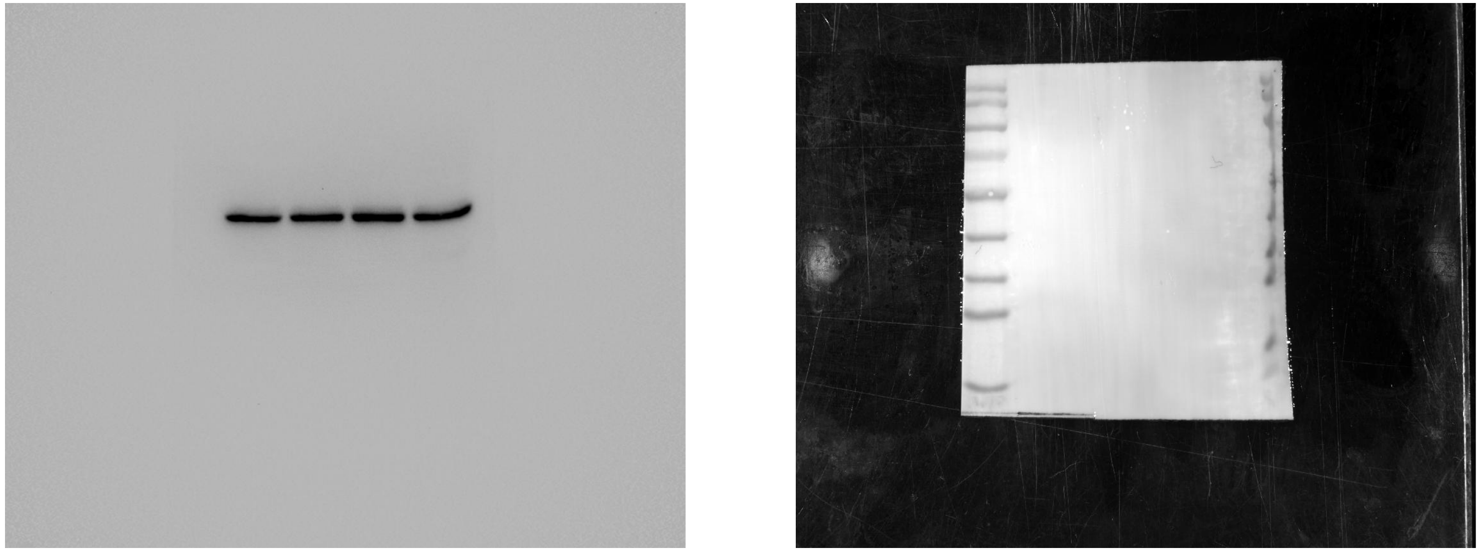

S-fig6

A549 ZC3H15

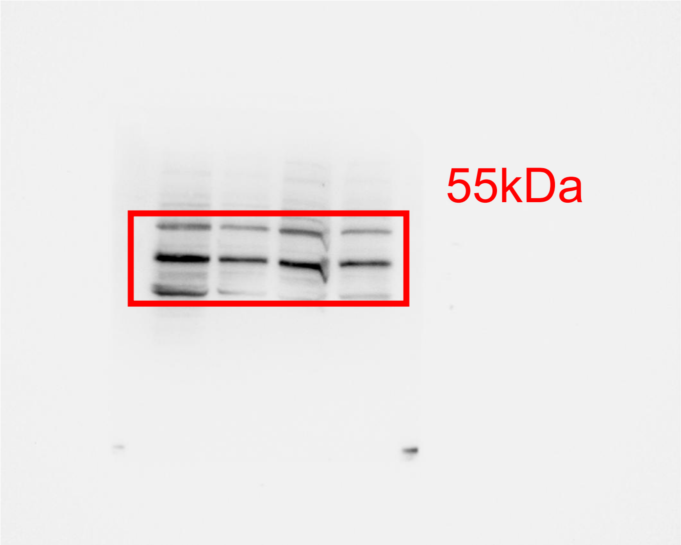

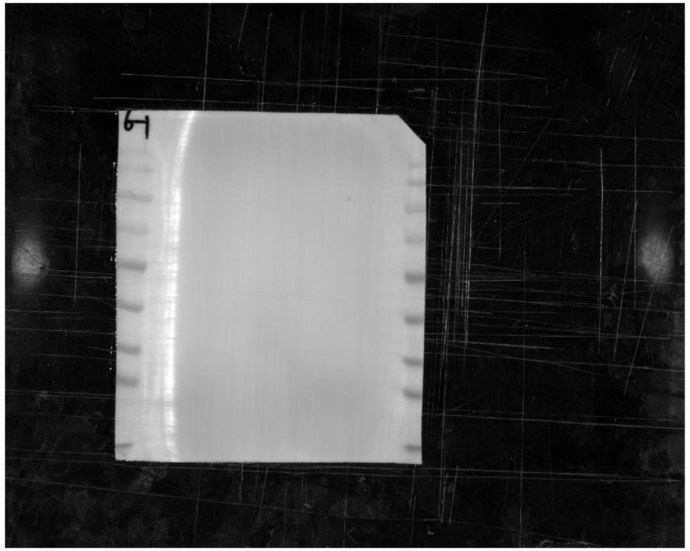

H1299 ZC3H15

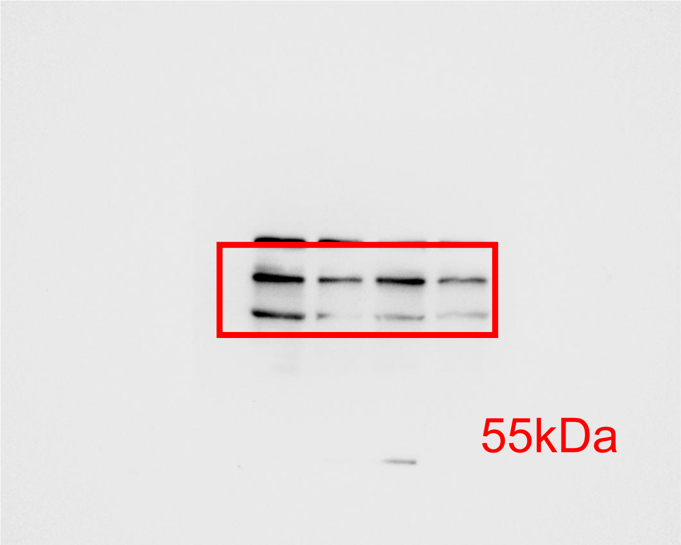

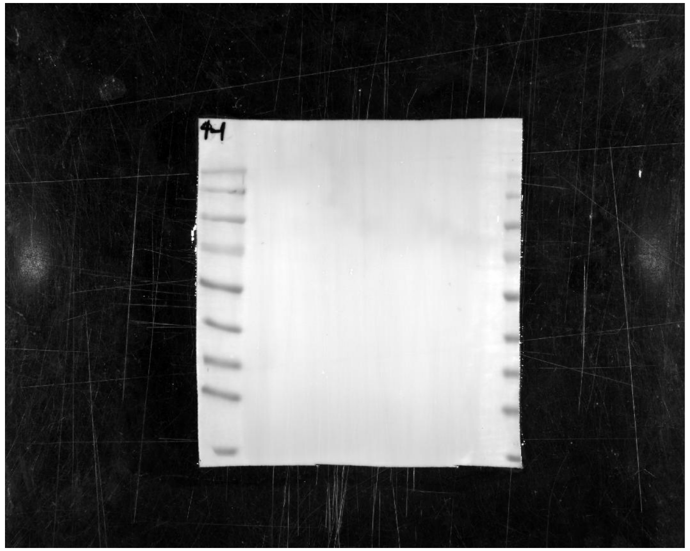

A549 pten

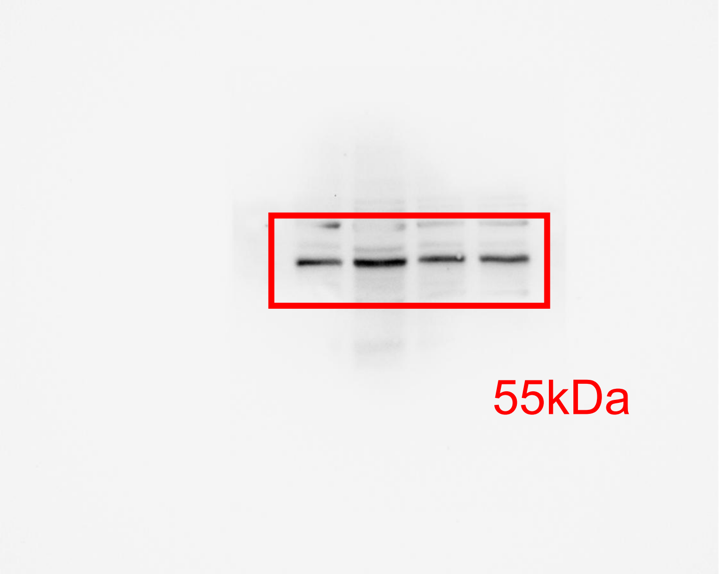

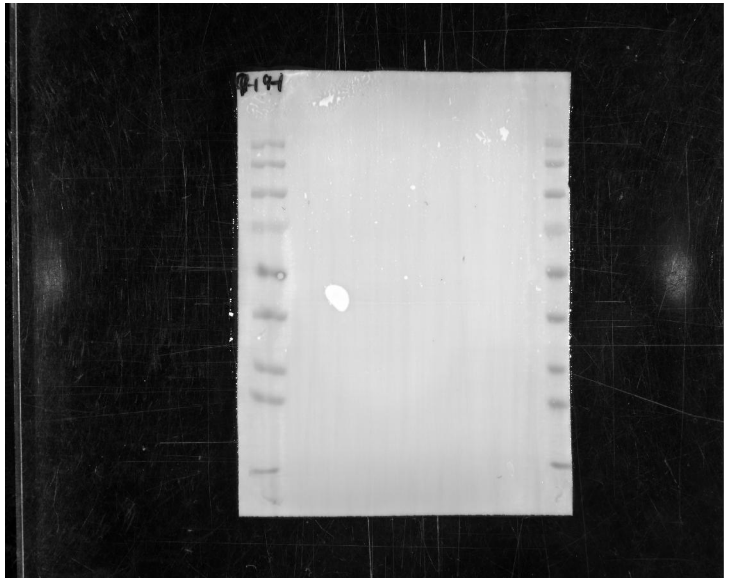

H1299 pten

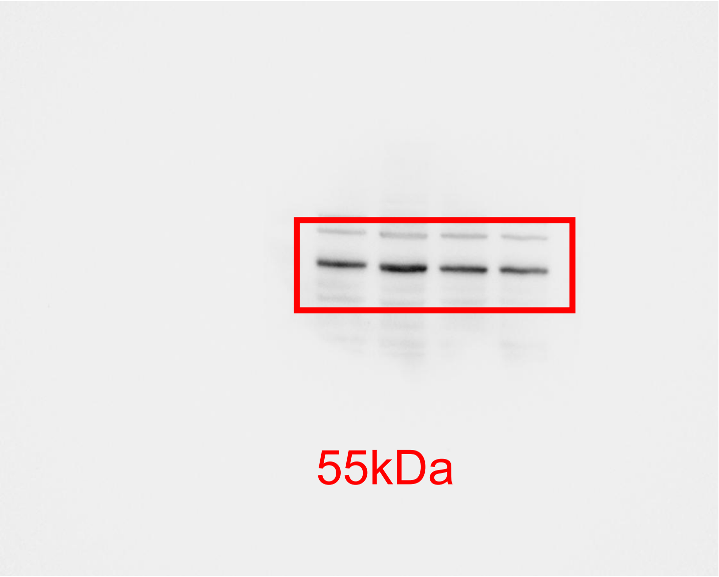

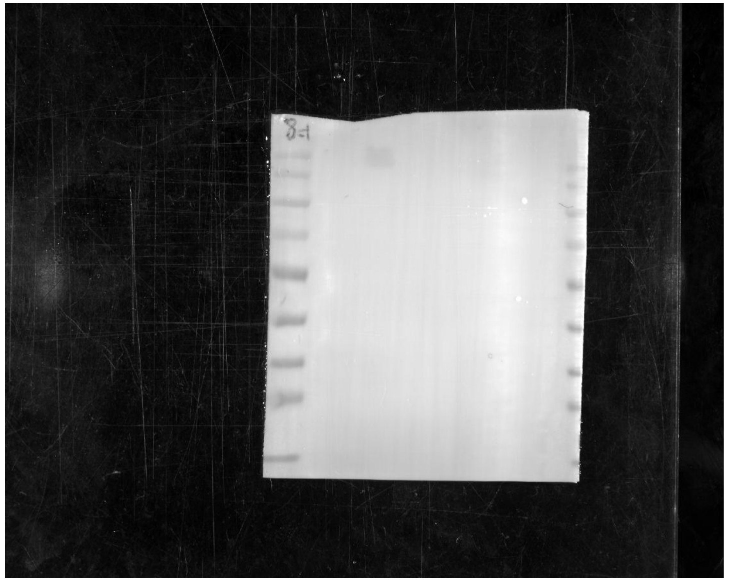

A549 P-AKT

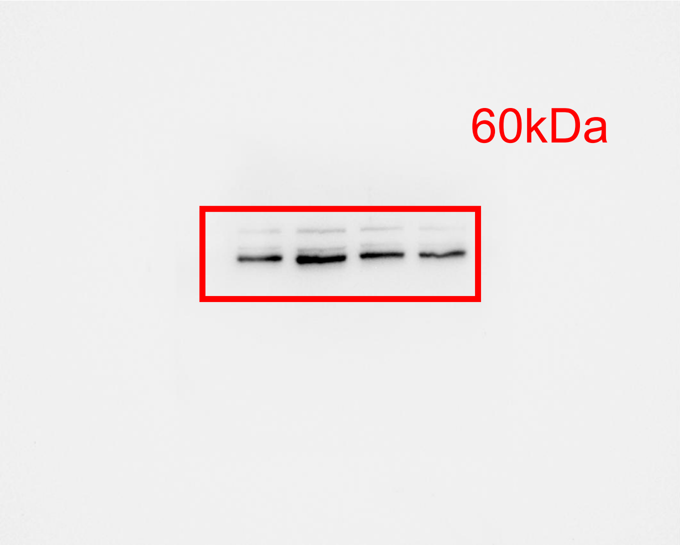

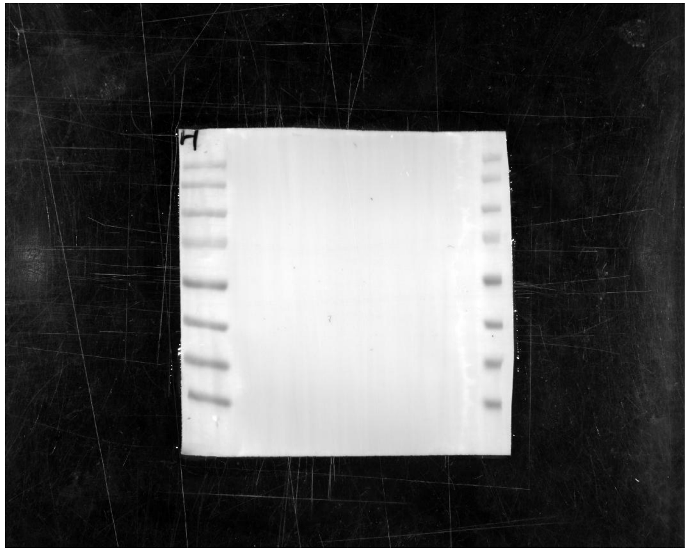

H1299 P-AKT

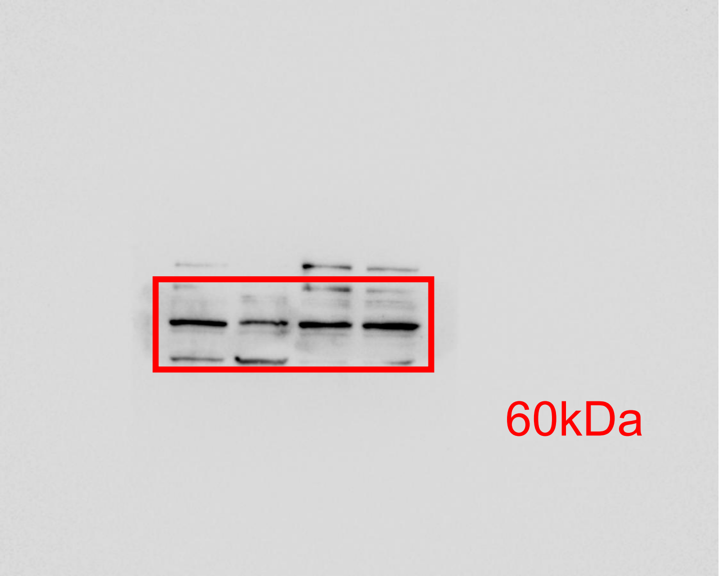

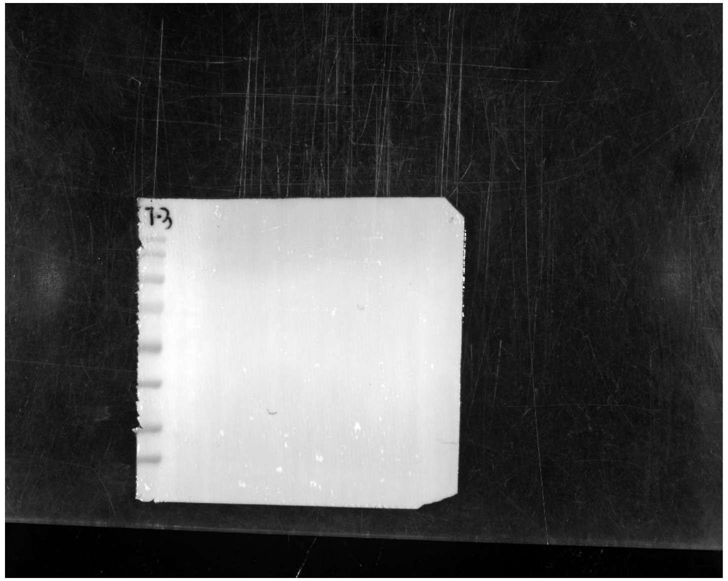

A549 AKT

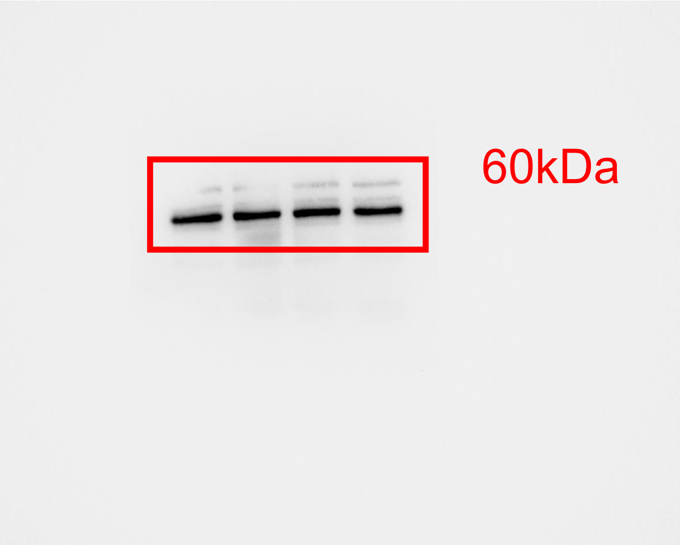

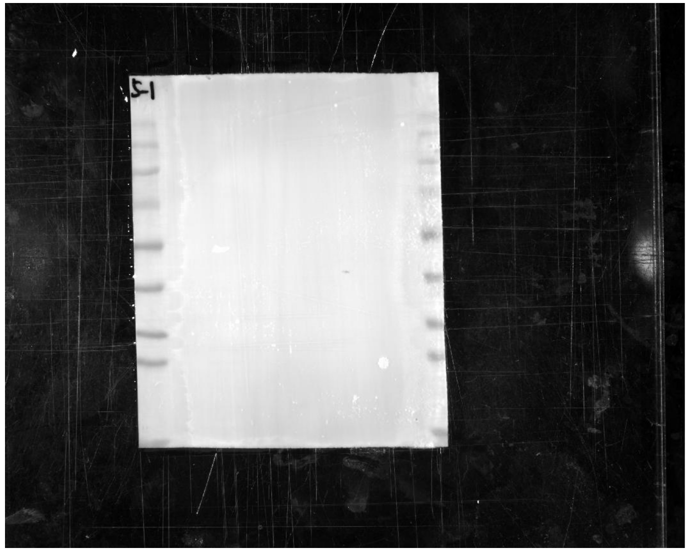

H1299 AKT

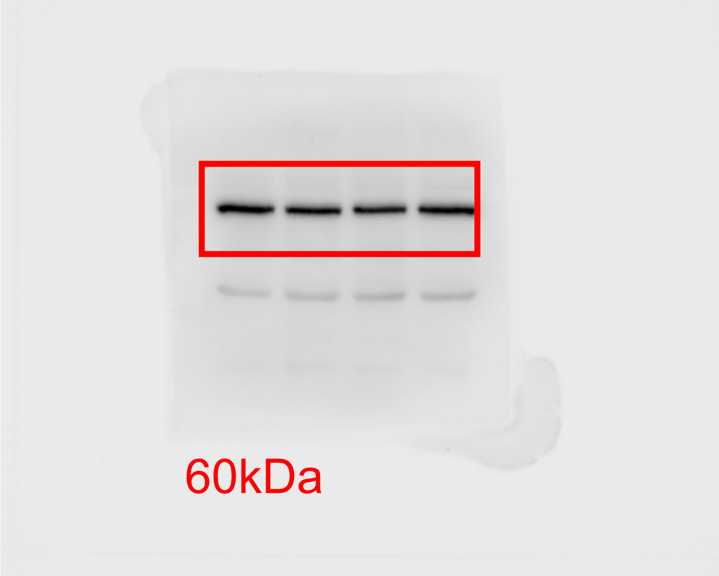

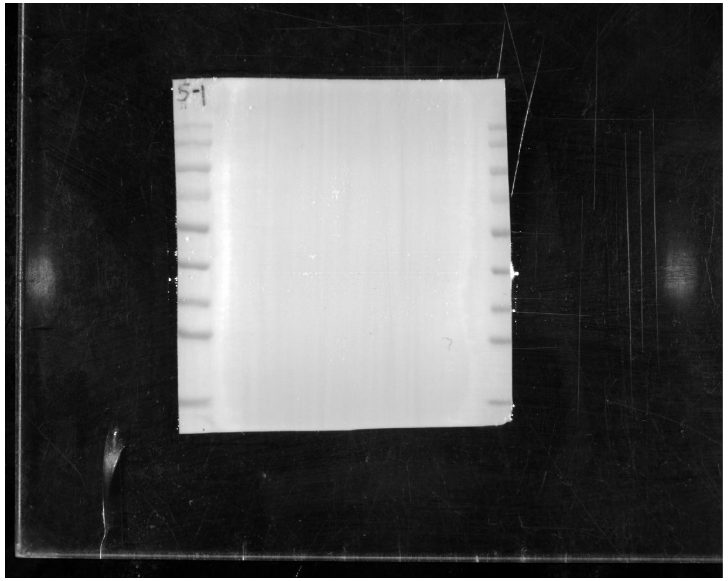

A549 P-MTOR

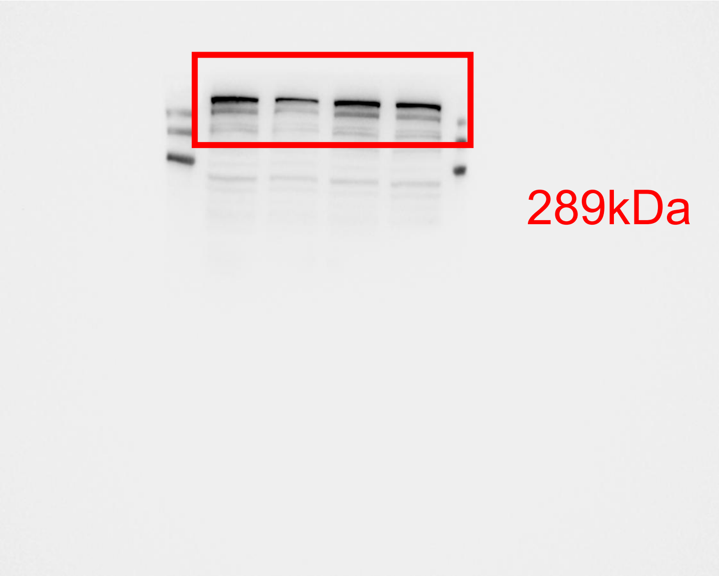

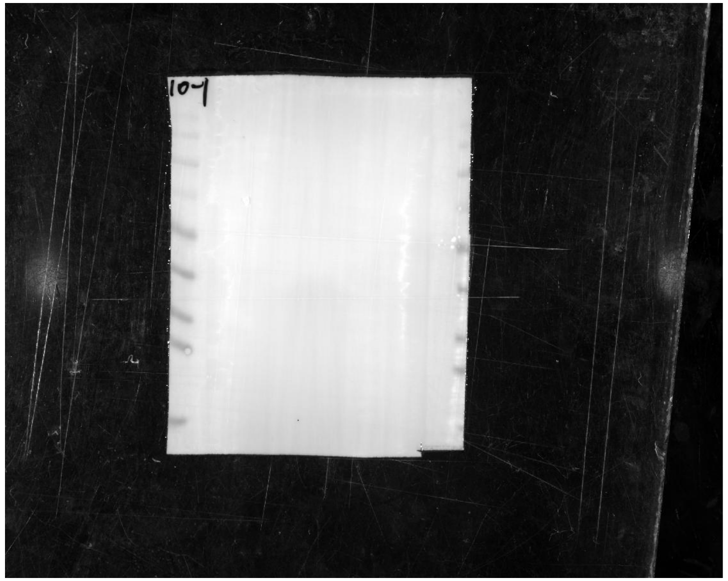

H1299 P-MTOR

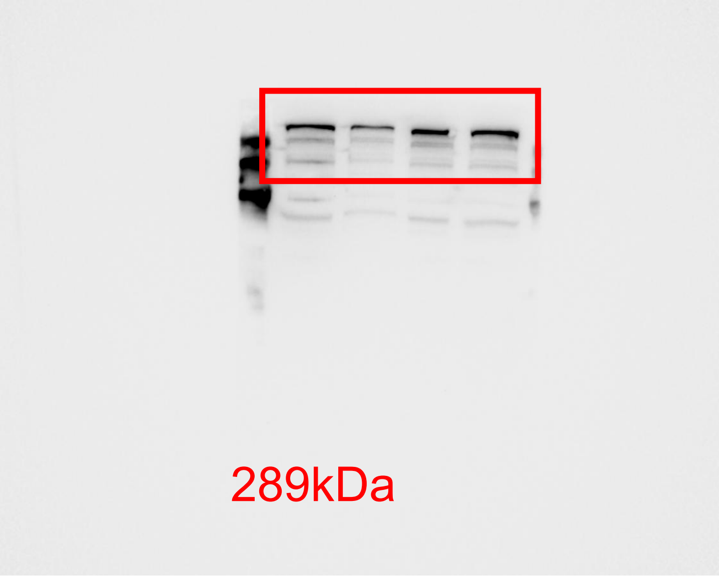

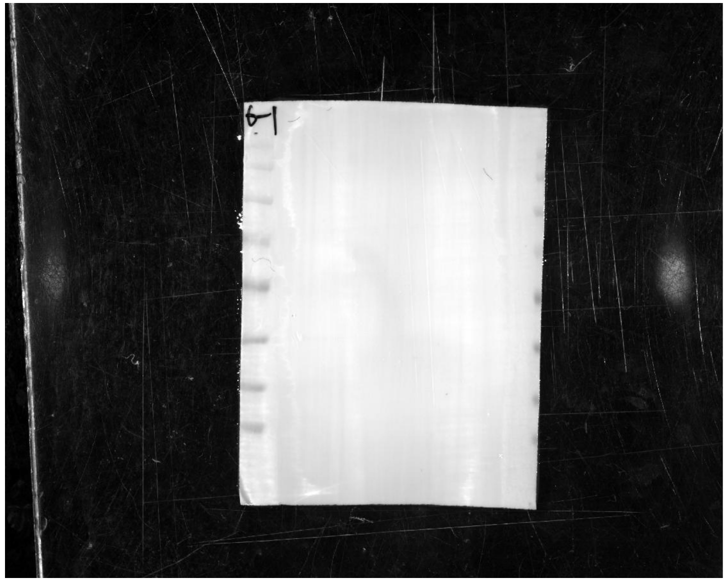

A549 MTOR

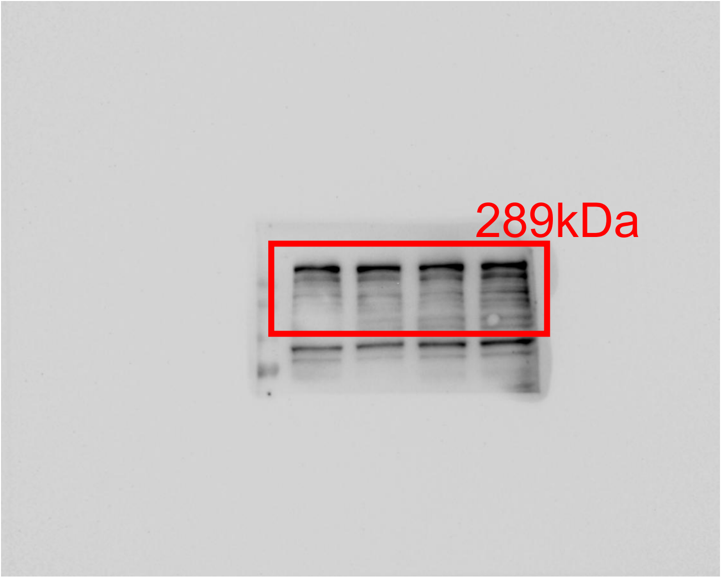

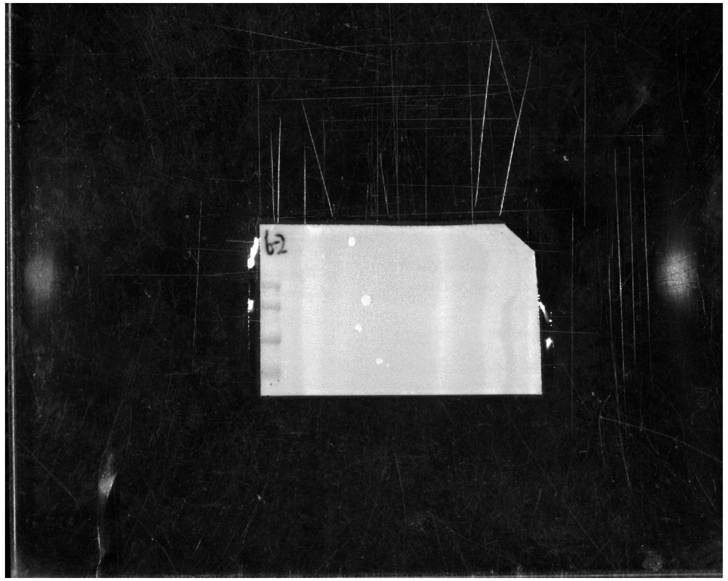

H1299 MTOR

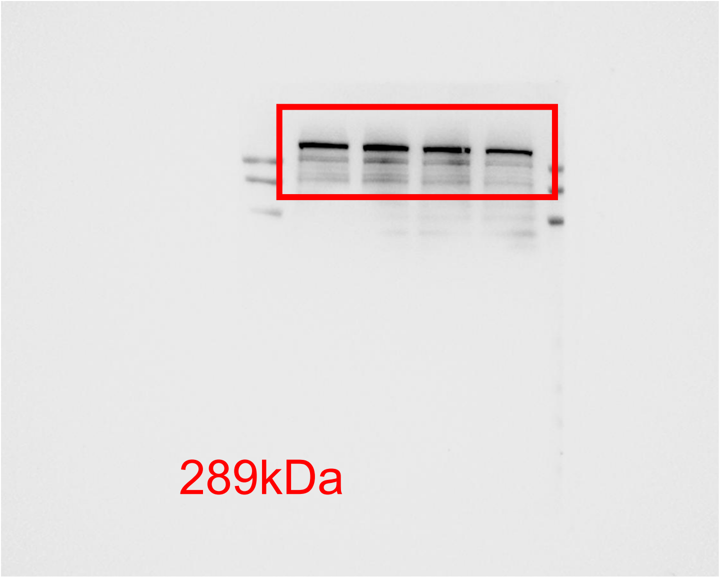

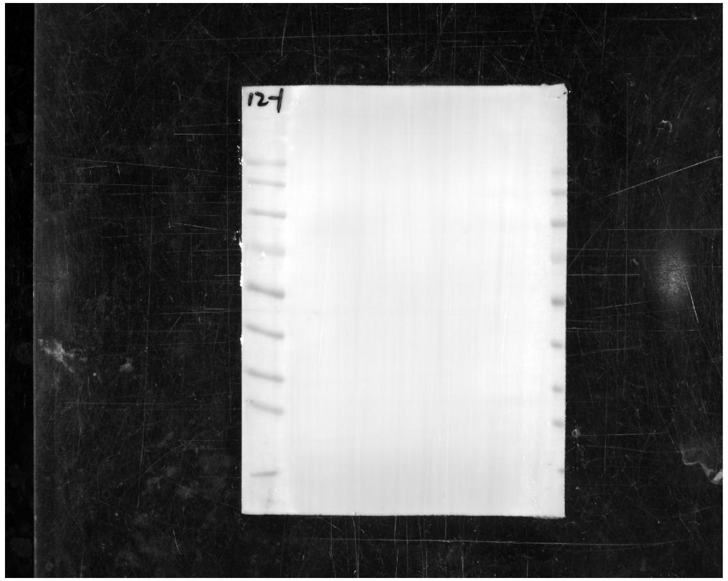

A549 CDK4

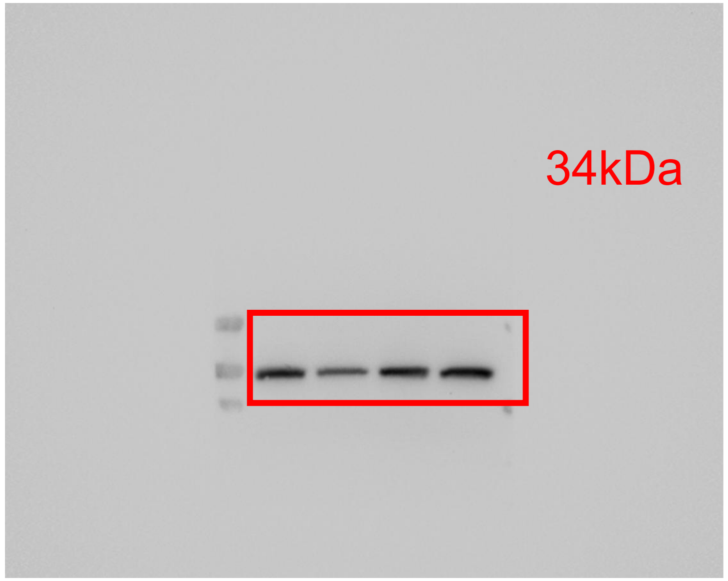

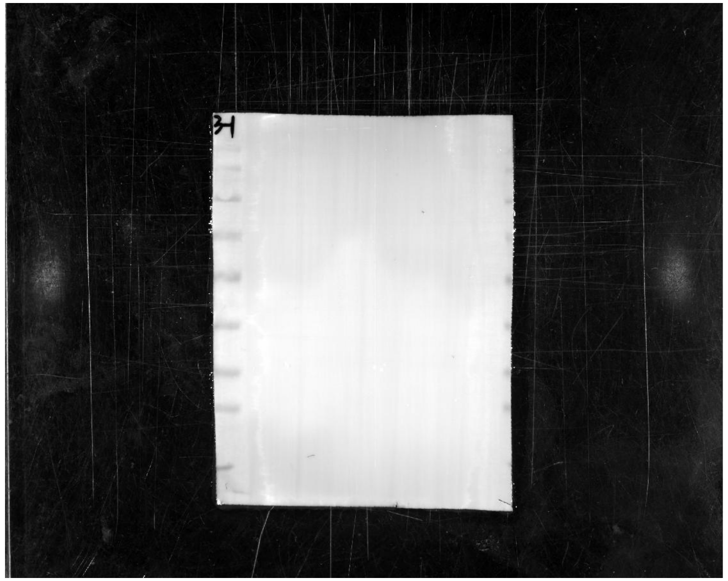

H1299 CDK4

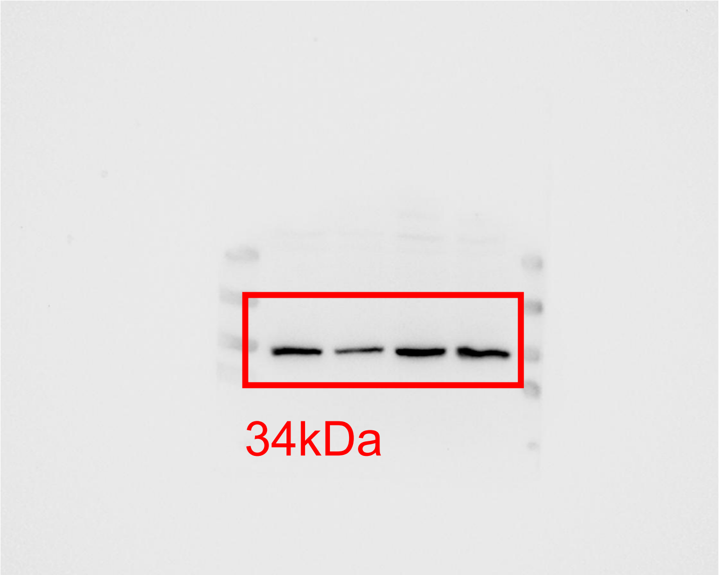

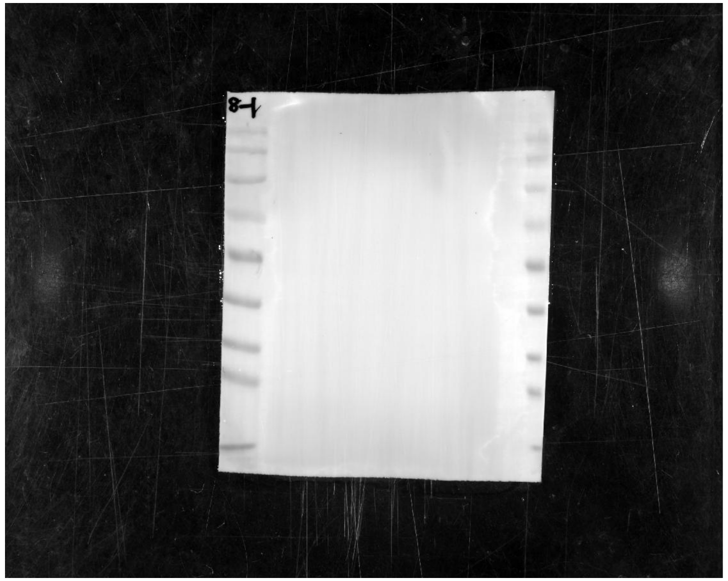

A549 CDK6

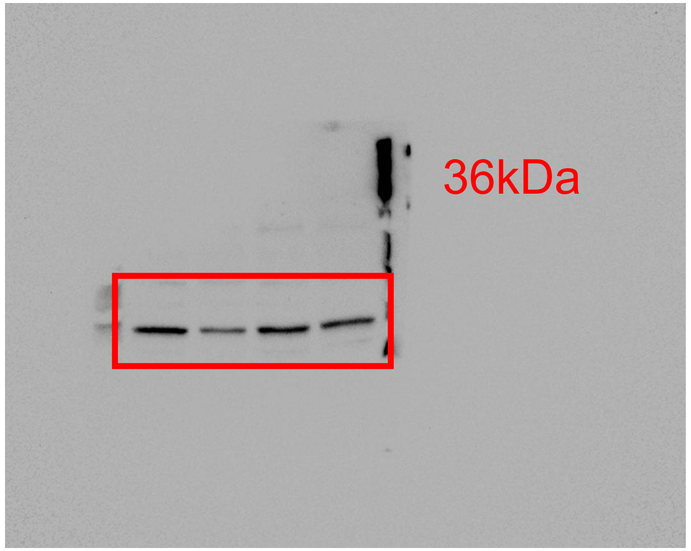

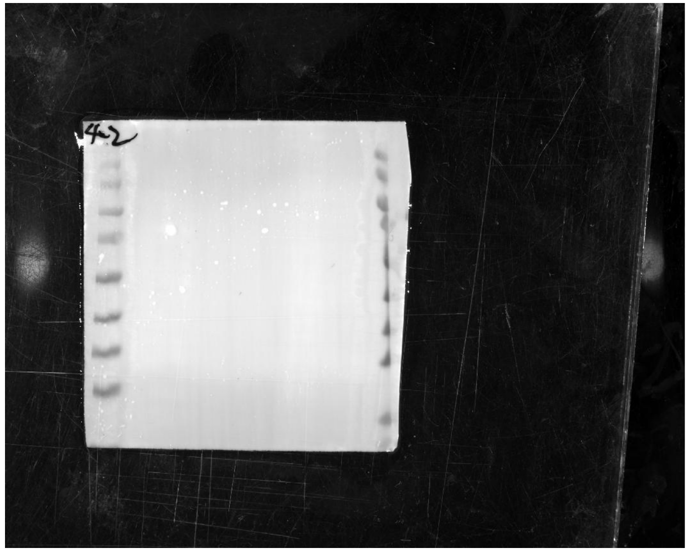

H1299 CDK6

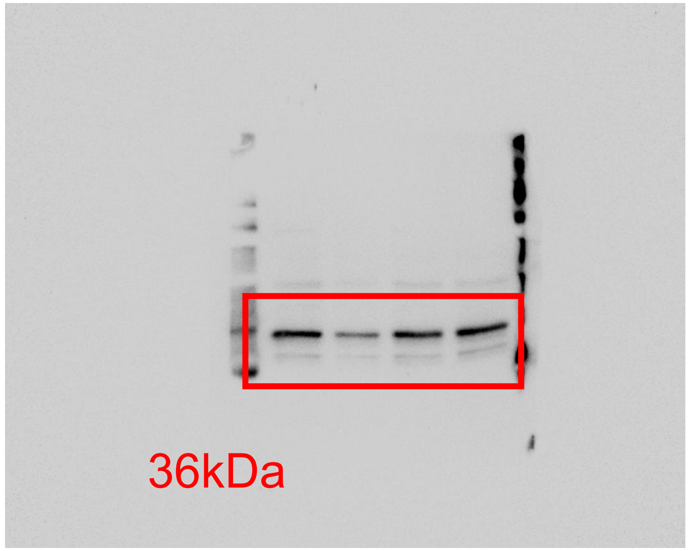

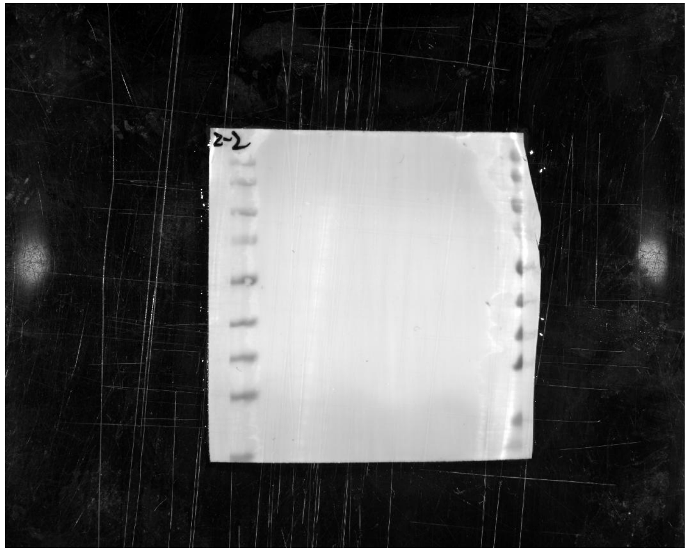

A549 D1

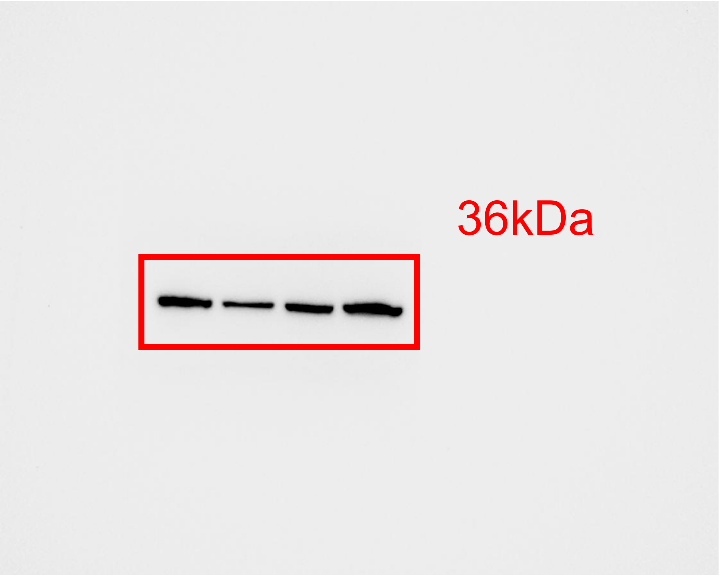

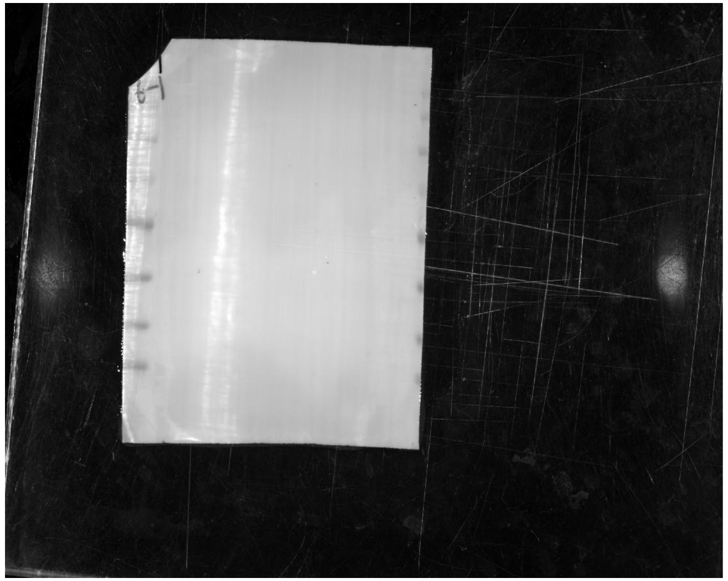

H1299 D1

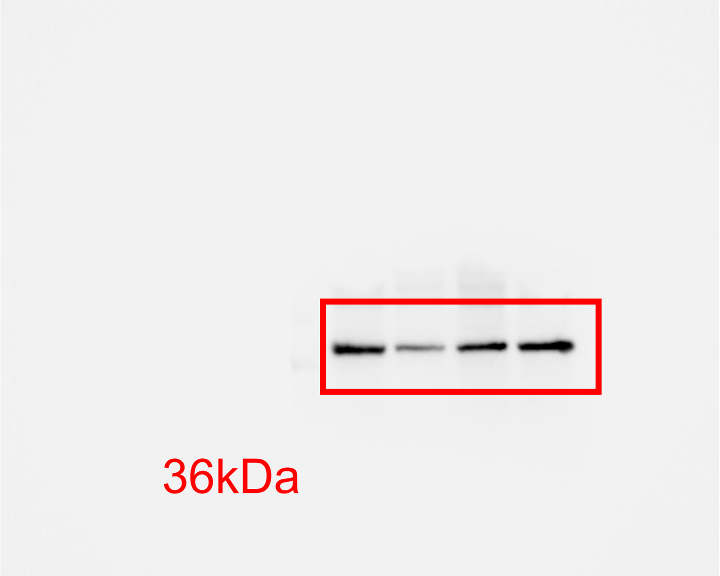

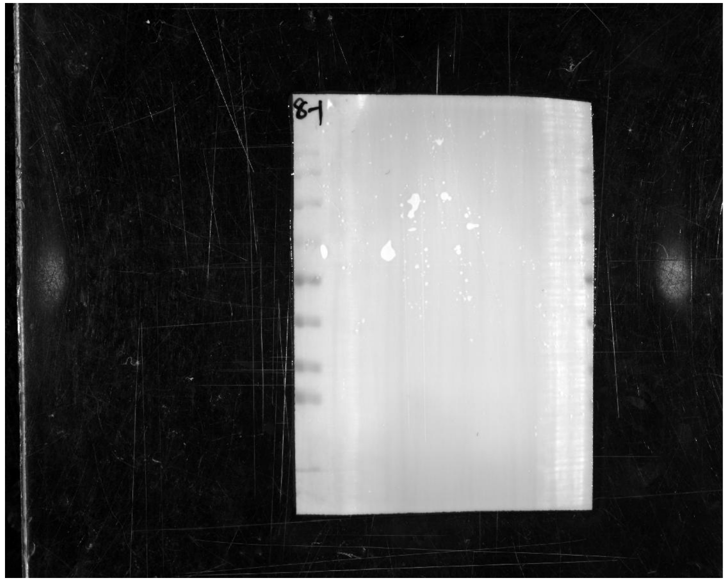

A549 E-CA

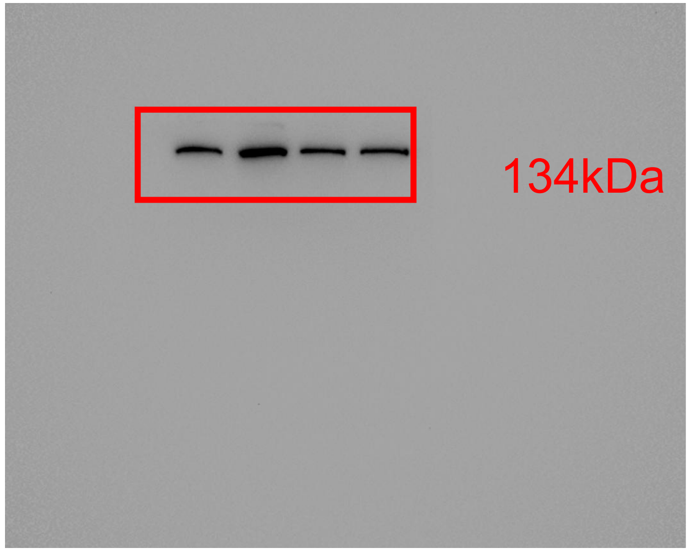

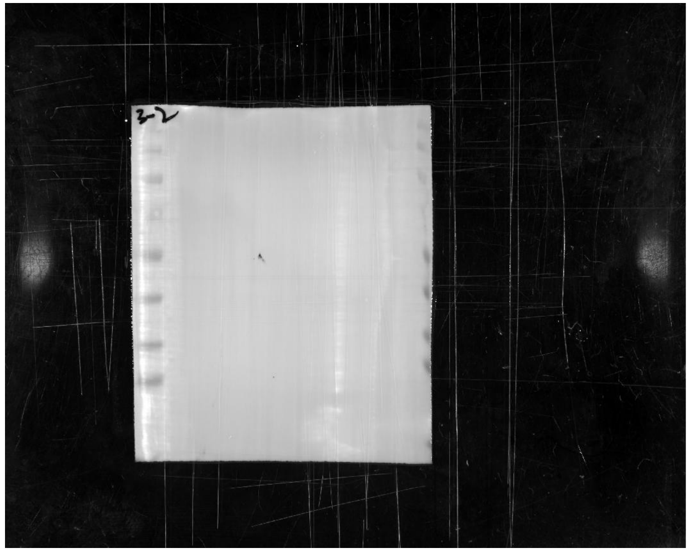

H1299 E-CA

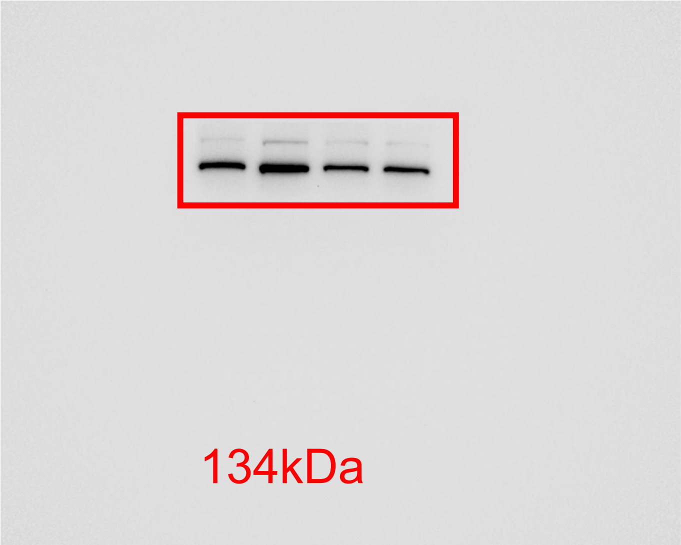

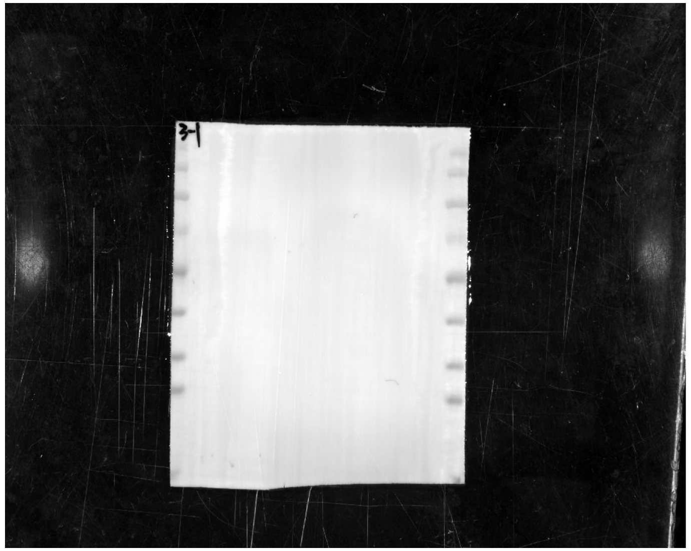

A549 N-CA

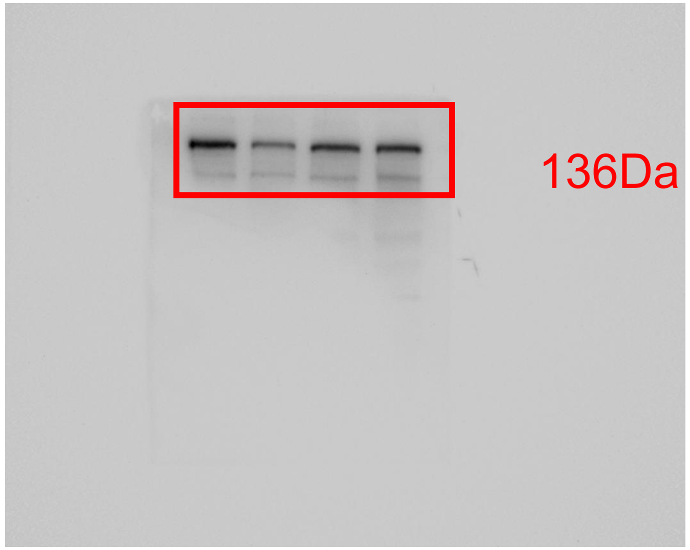

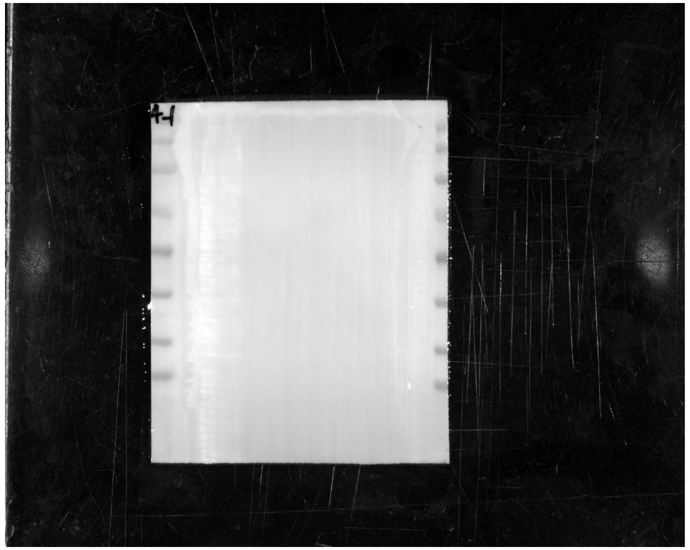

H1299 N-CA

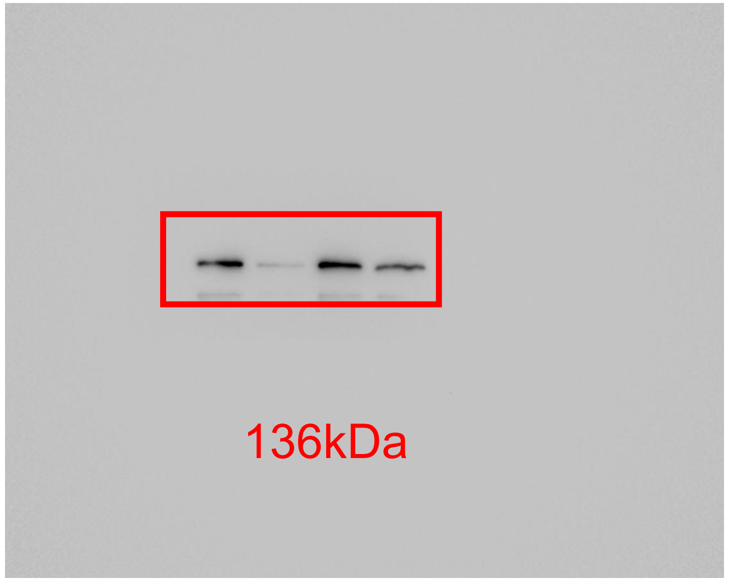

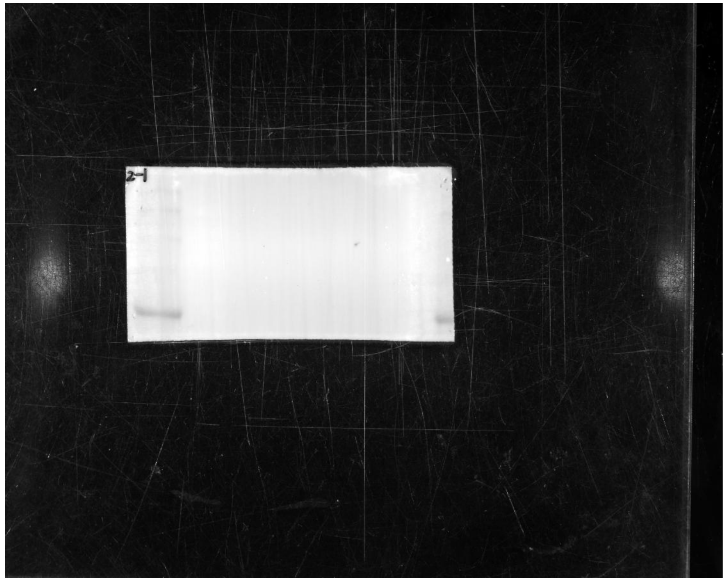

A549 MMP9

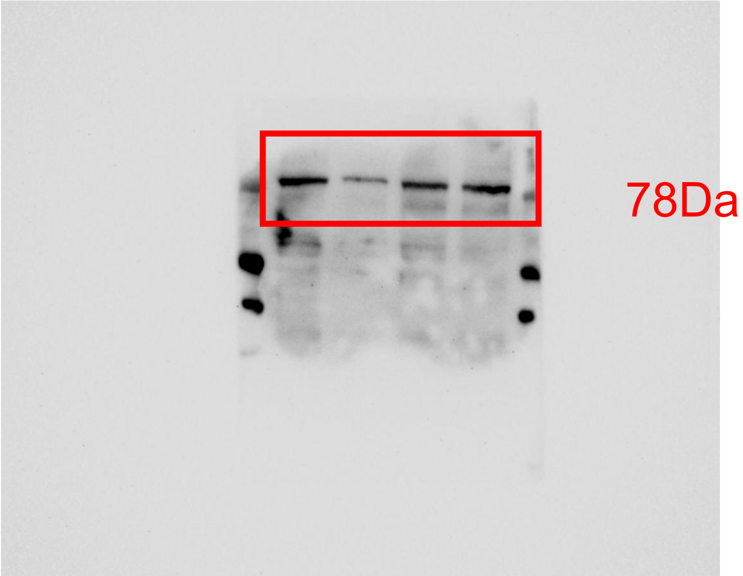

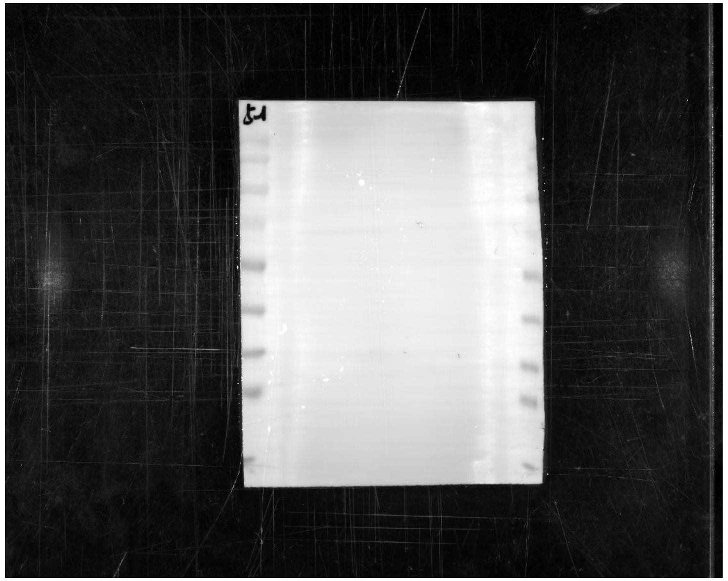

H1299 MMP9

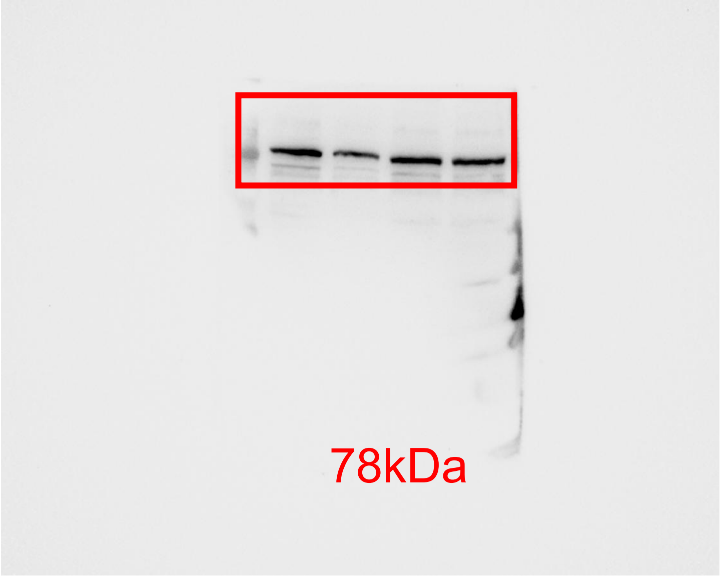

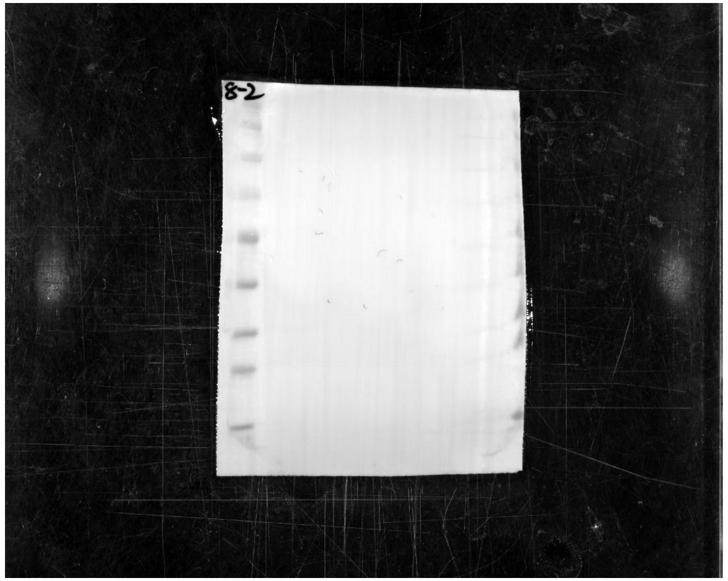

A549 β-actin

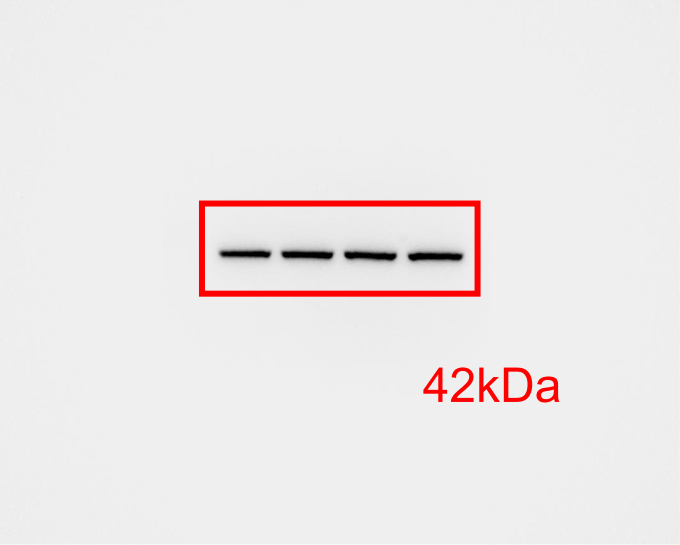

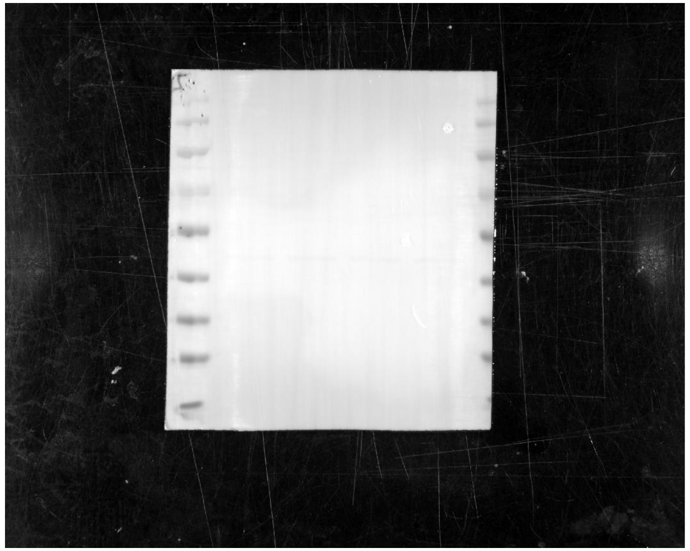

H1299 β-actin

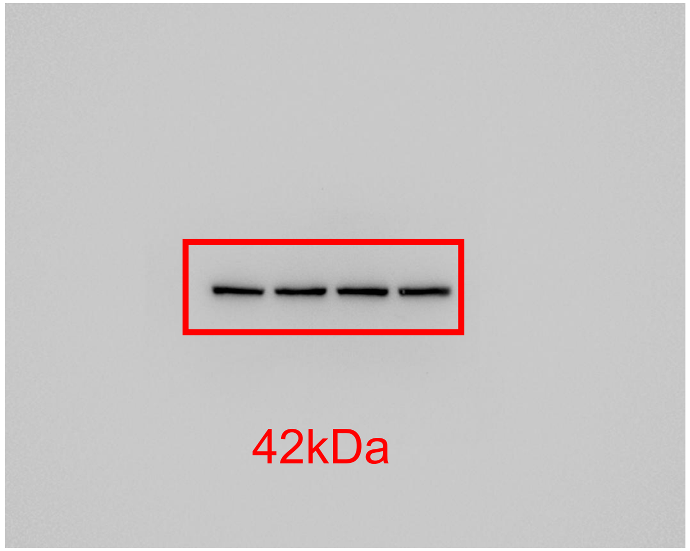

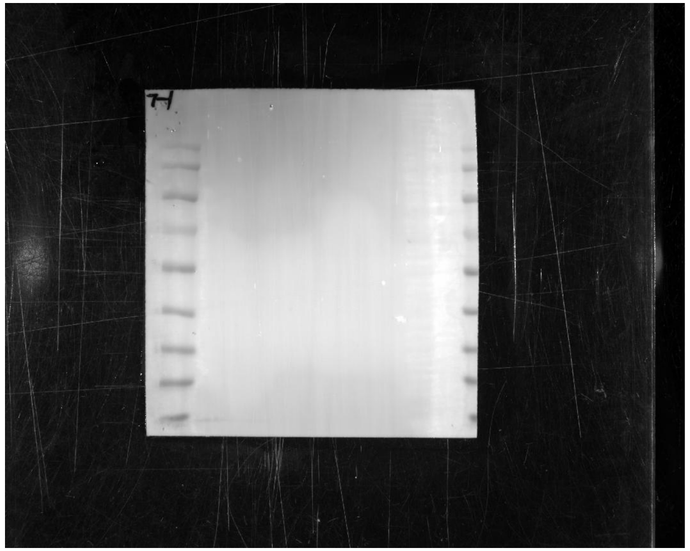

FIG 5

A549 myc

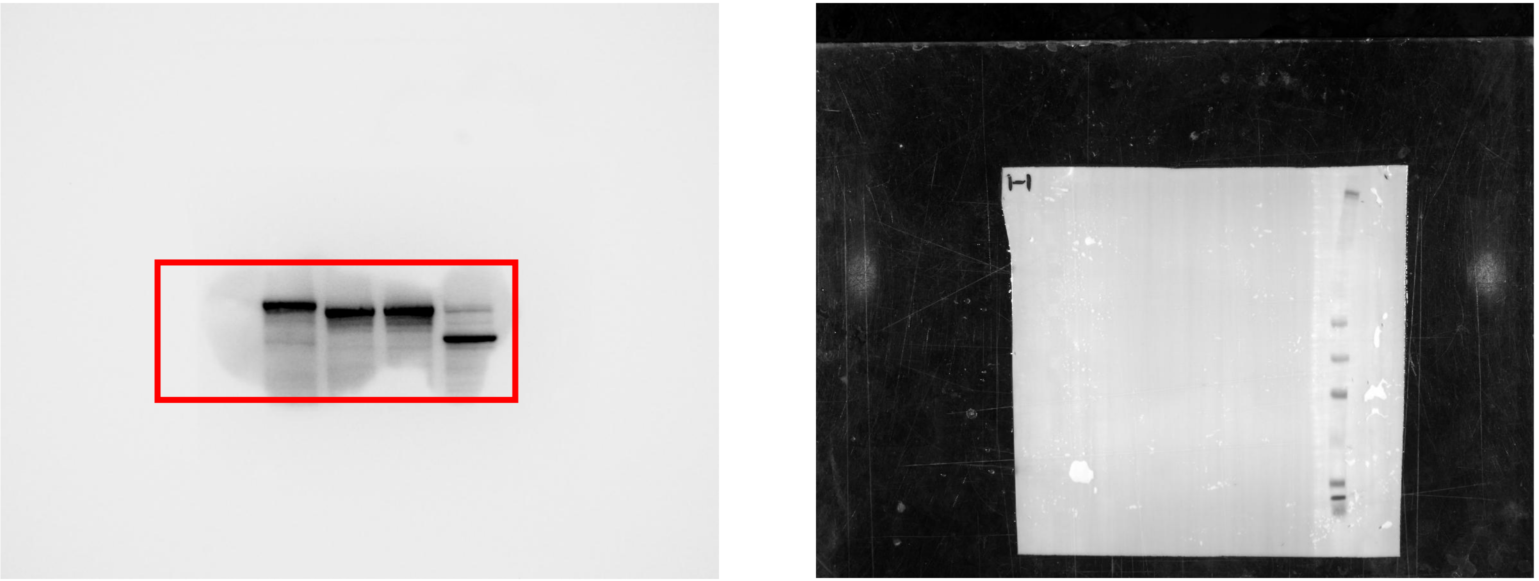

H1299 myc

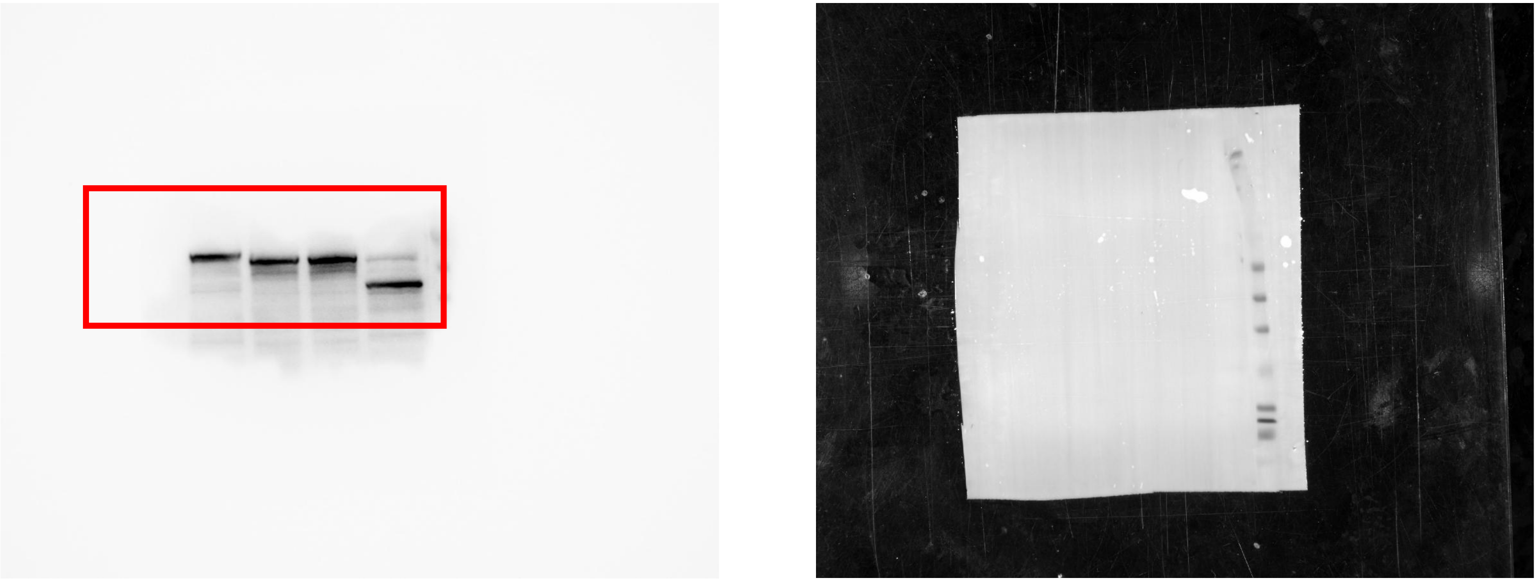

A549 β-actin

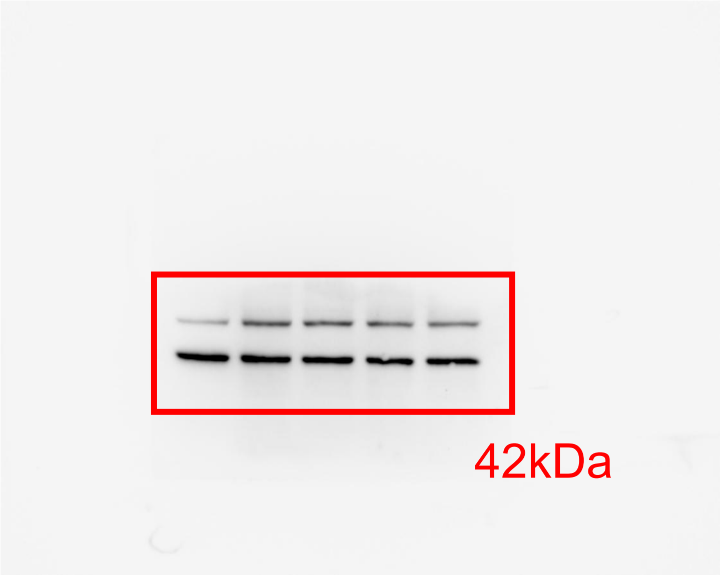

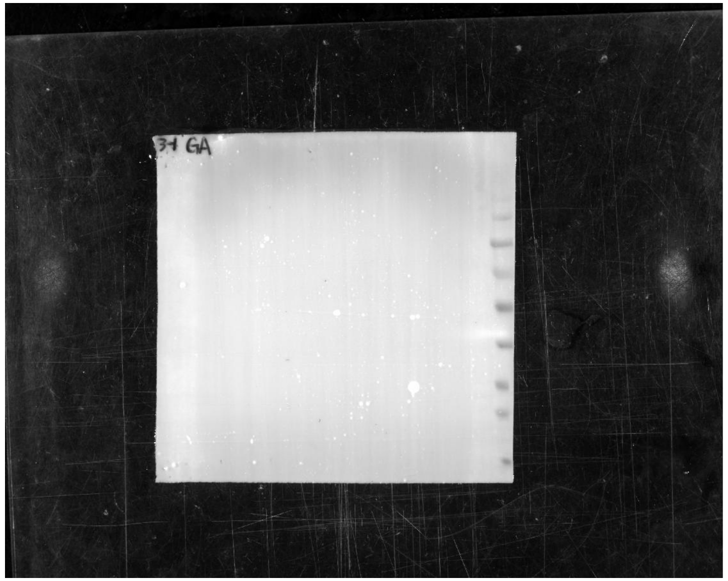

H1299 β-actin

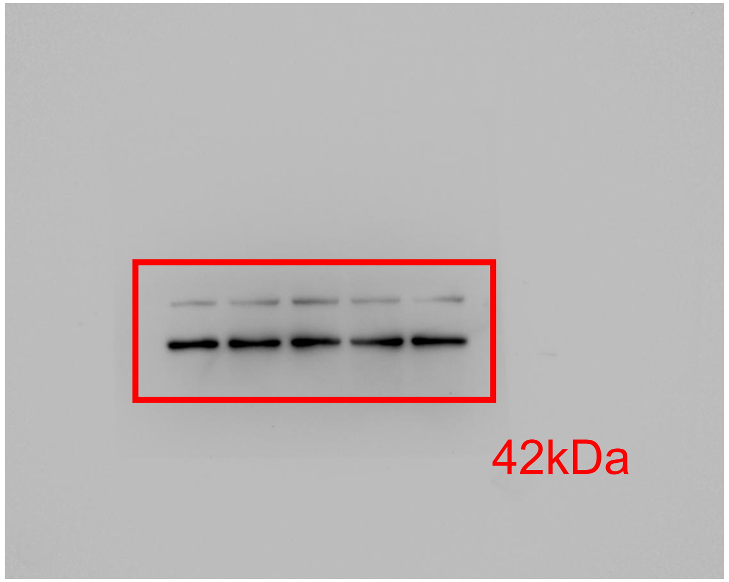

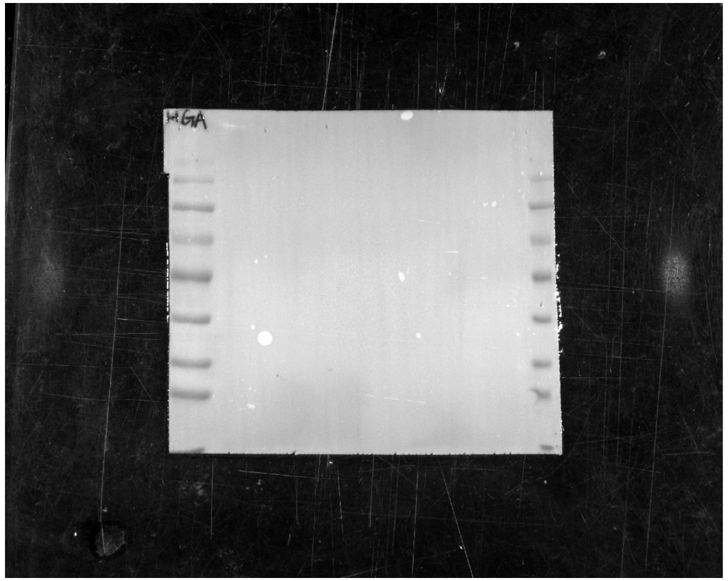

A549 myc

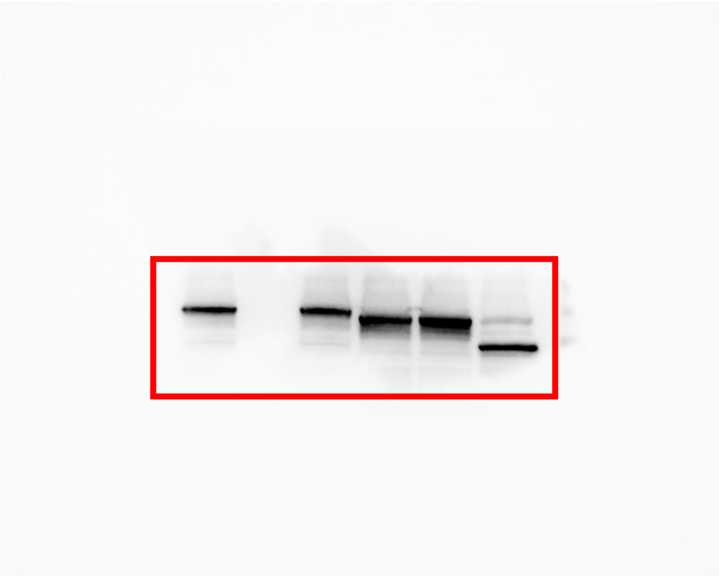

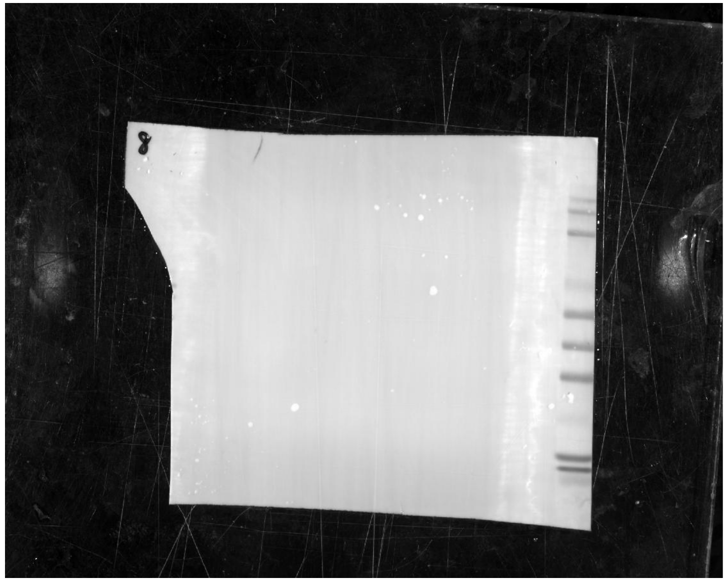

H1299 myc

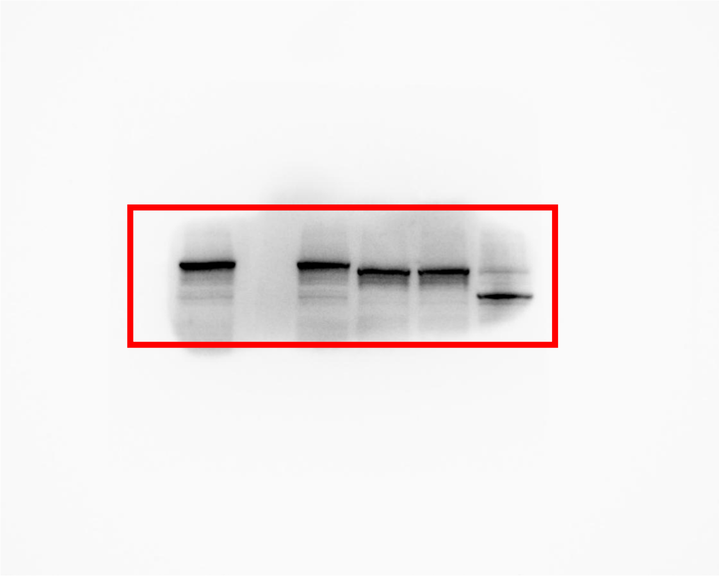

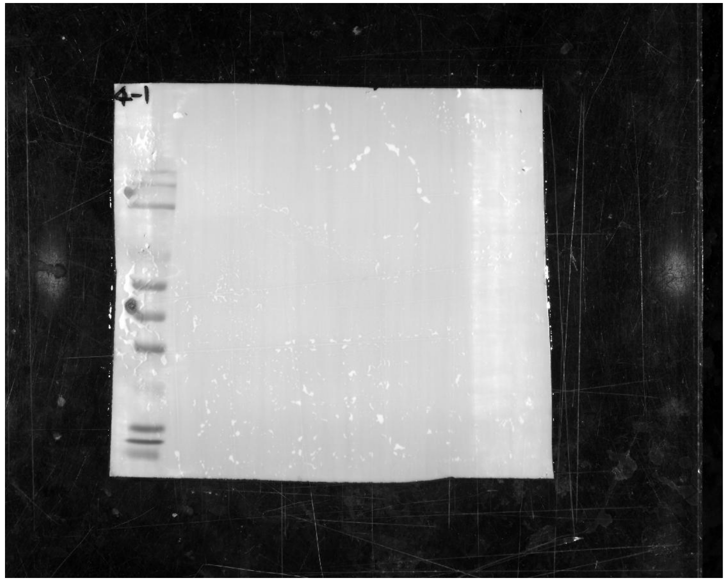

A549 pten

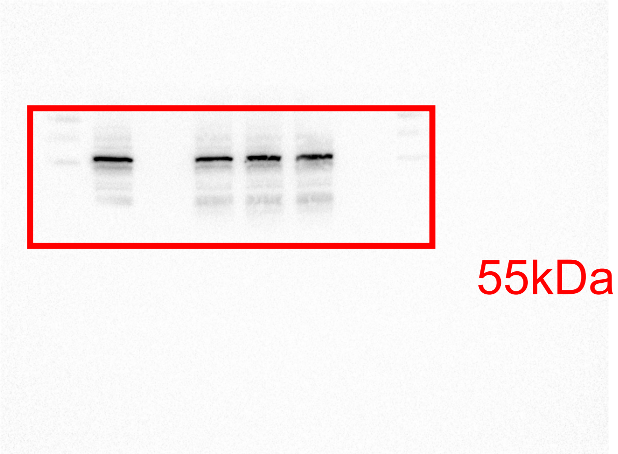

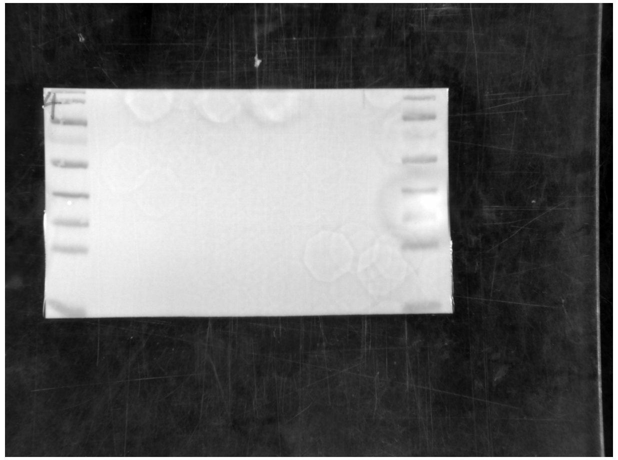

H1299 pten

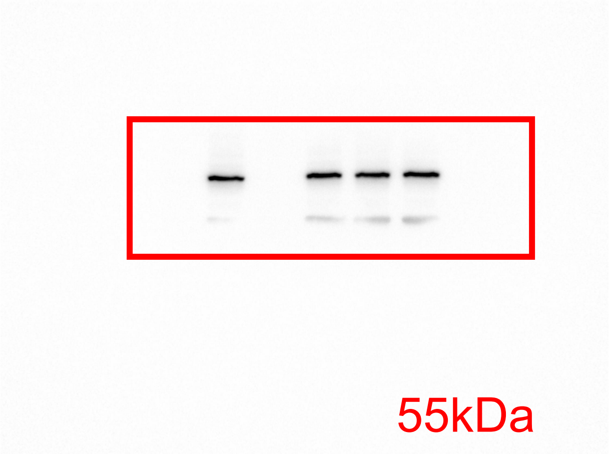

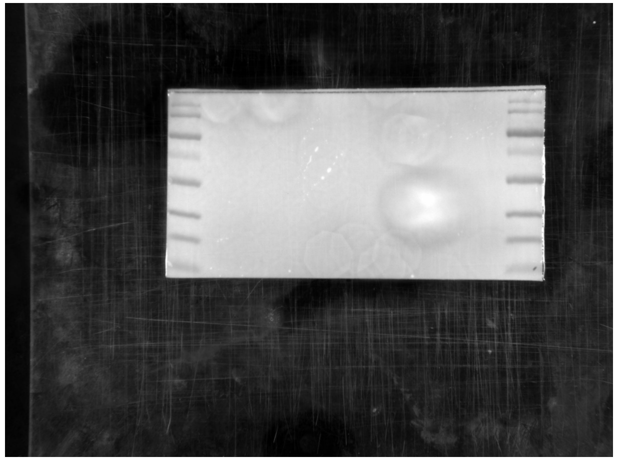

H1299 myc

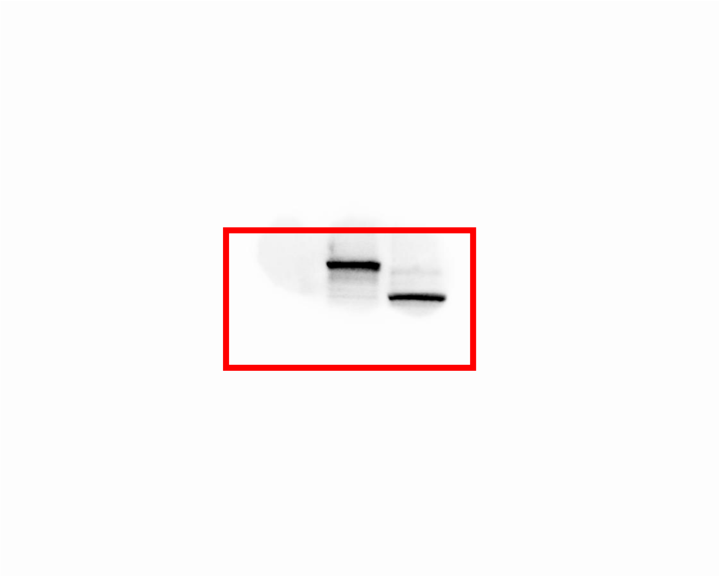

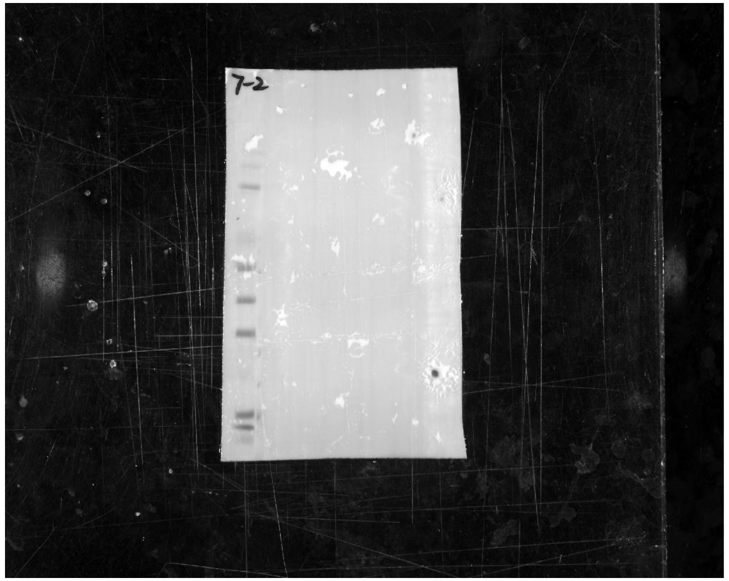

A549 myc

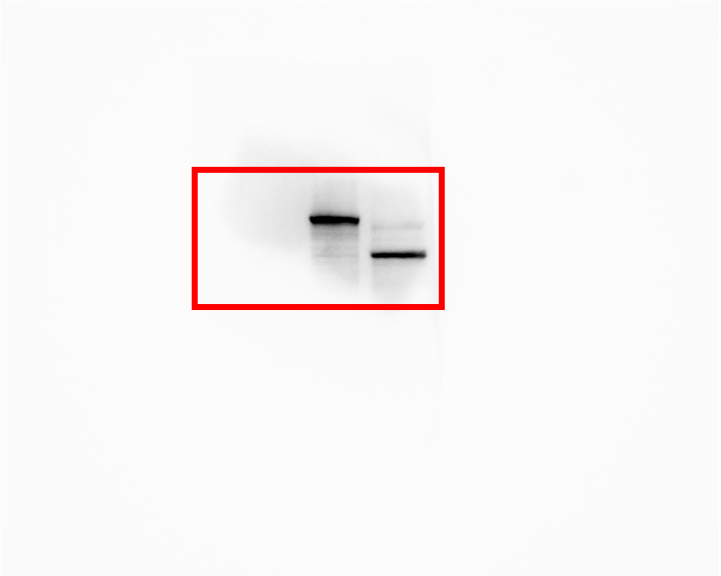

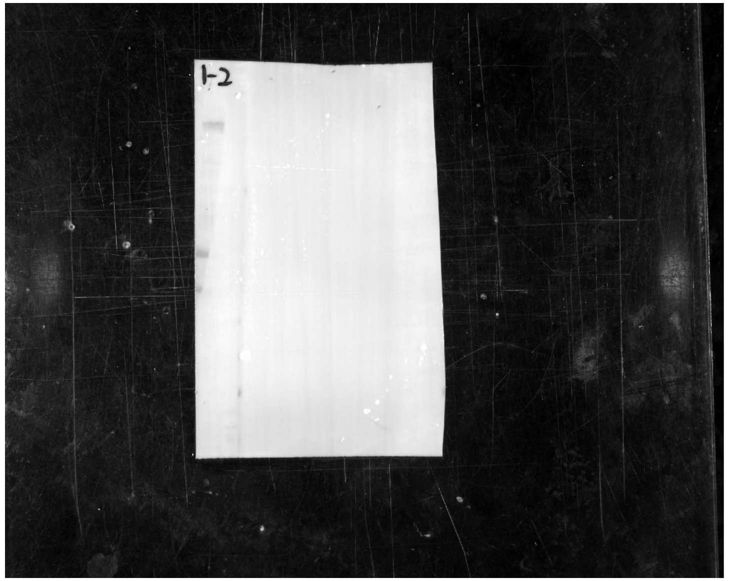

H1299 p-akt

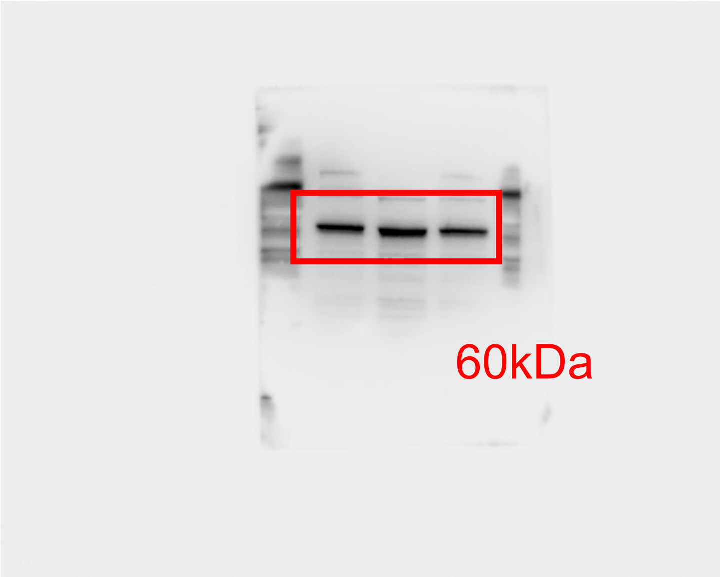

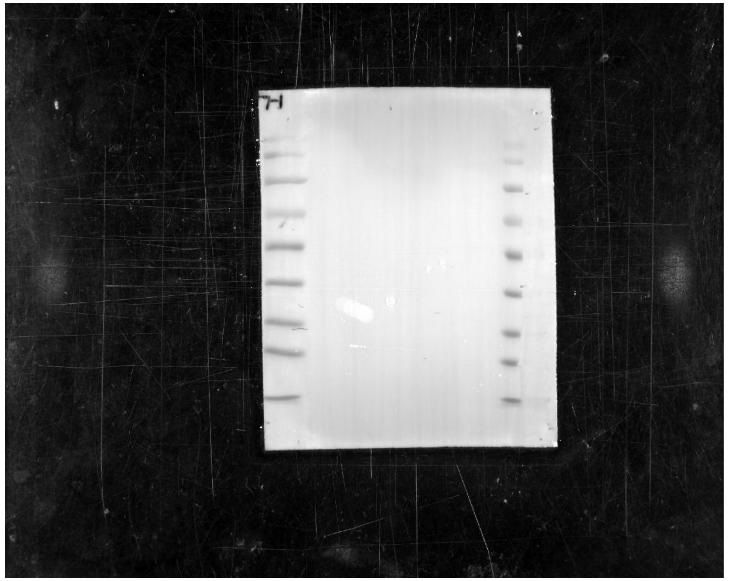

A549 p-akt

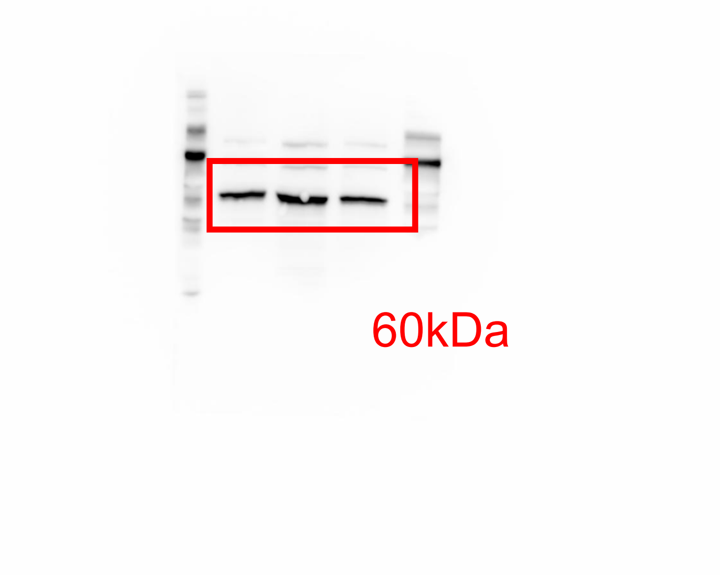

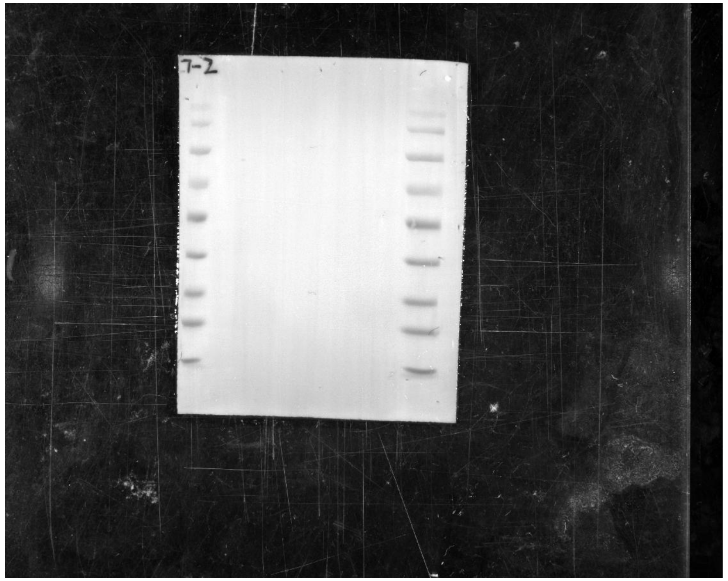

H1299 akt

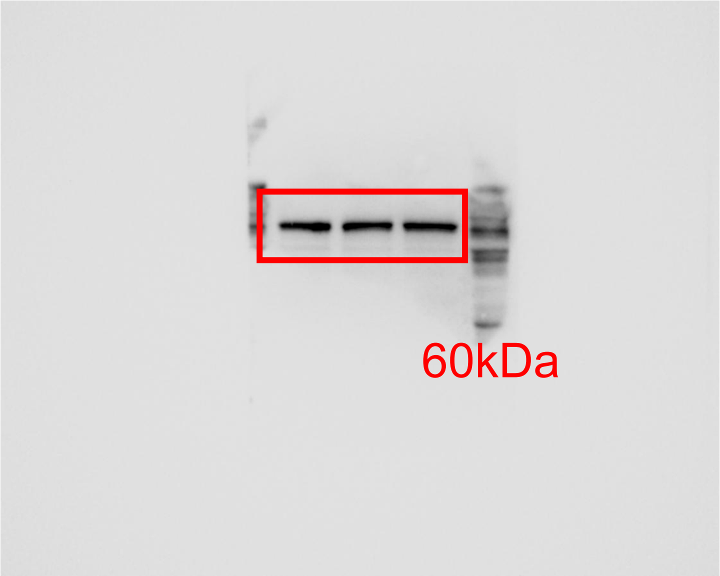

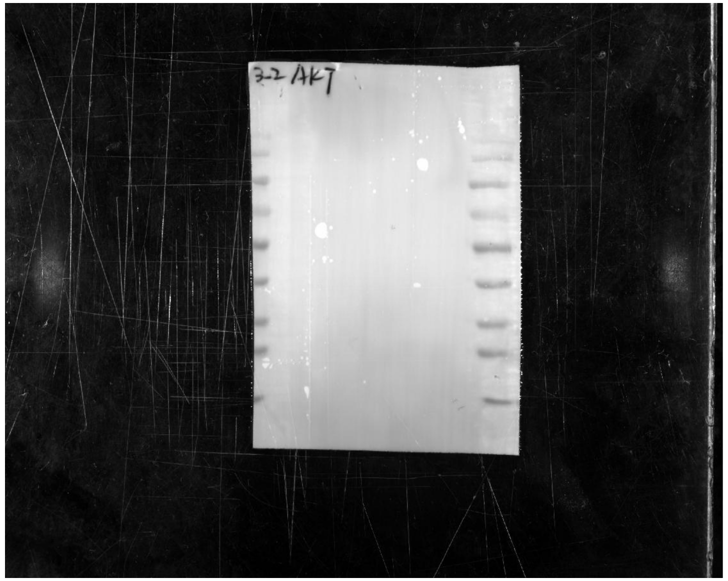

A549 akt

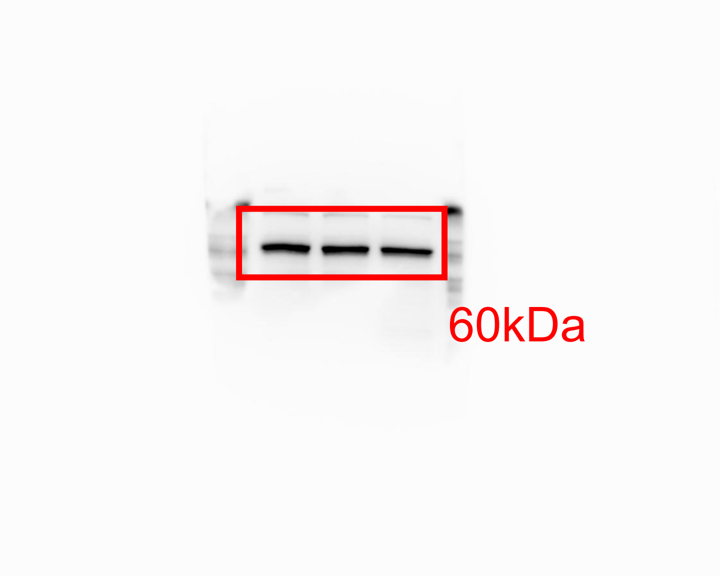

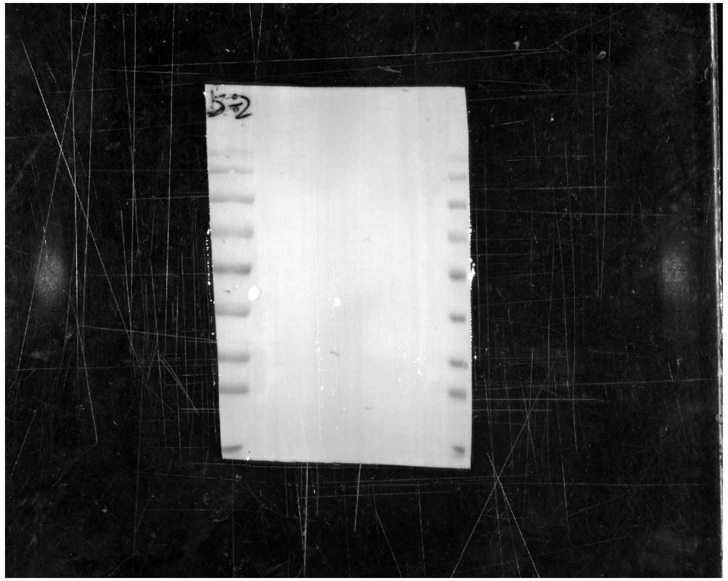

H1299 p-mtor

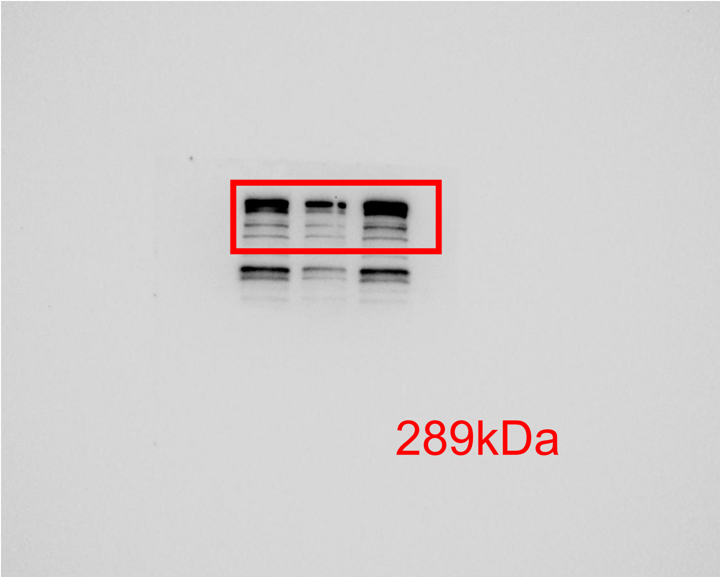

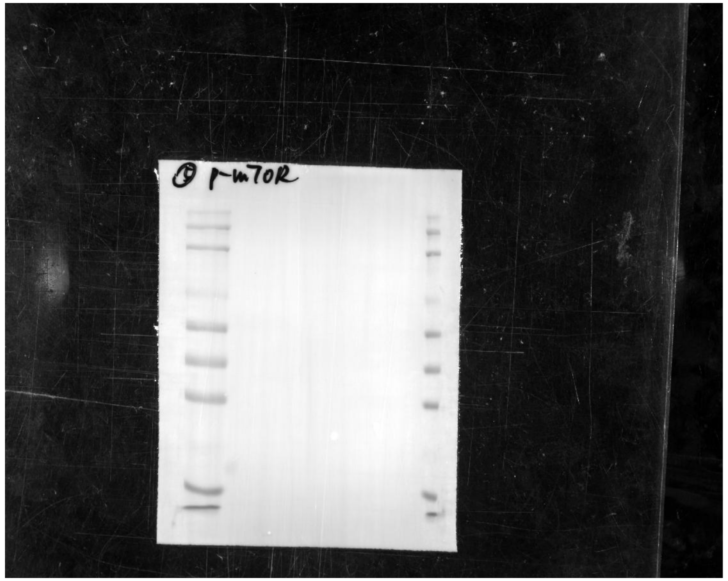

A549 p-mtor

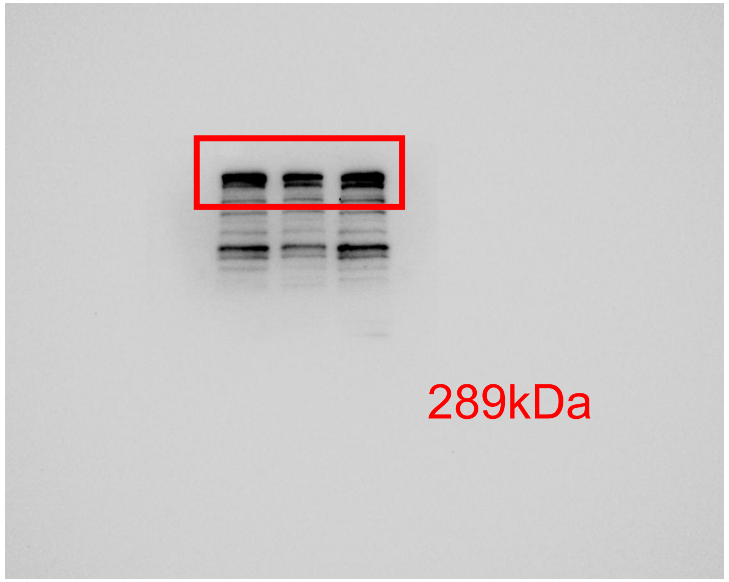

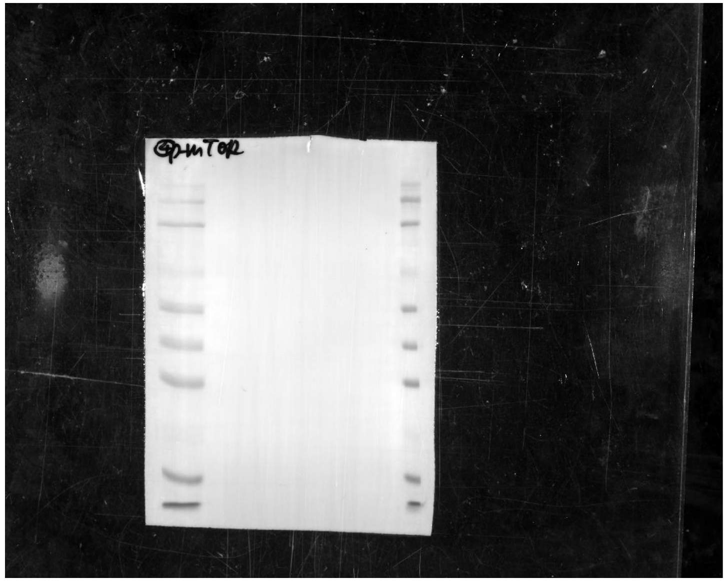

H1299 mtor

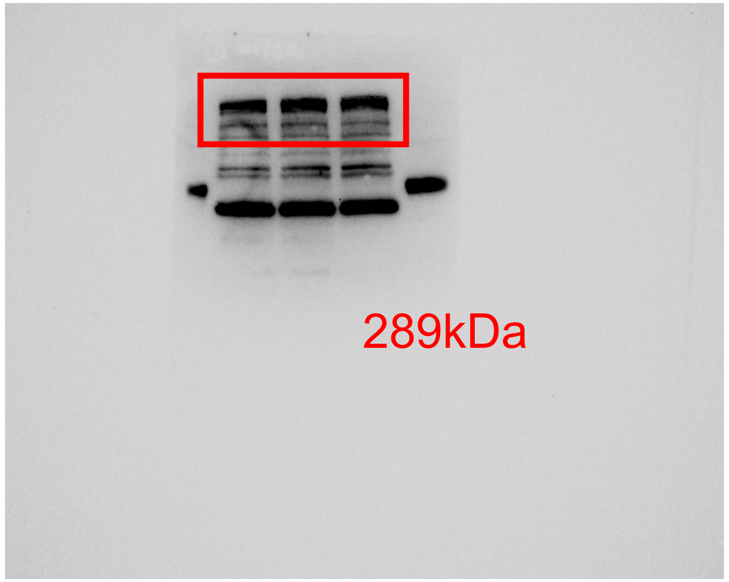

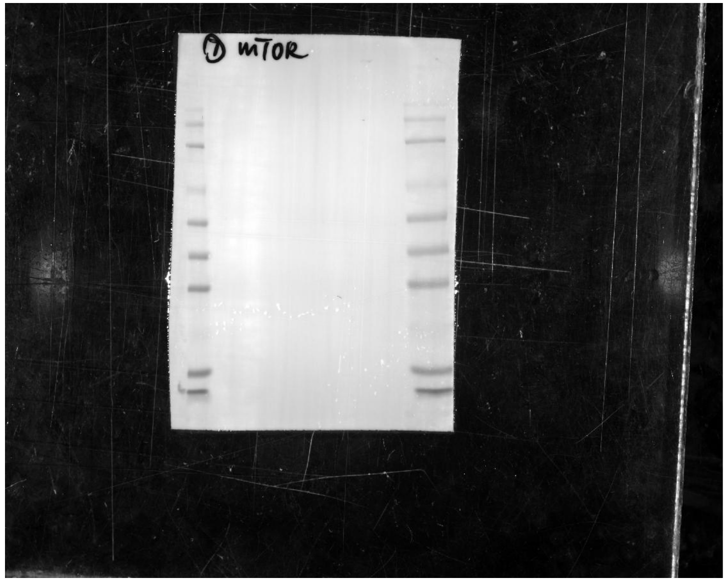

A549 mtor

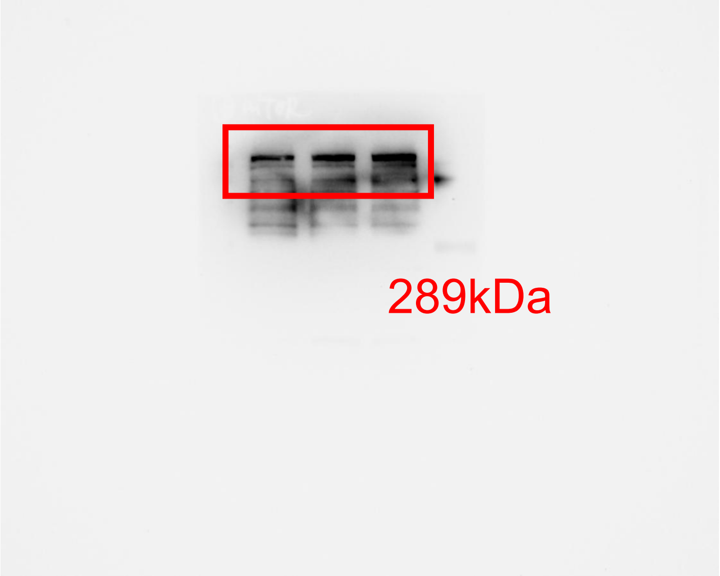

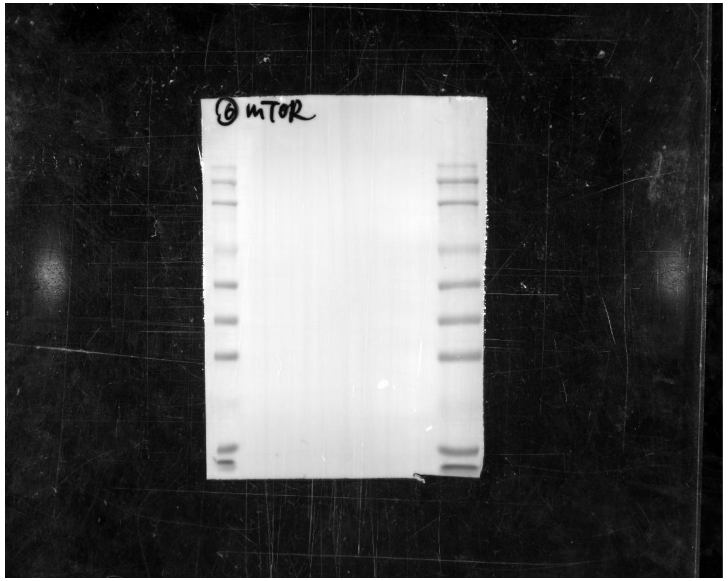

H1299 cdk4

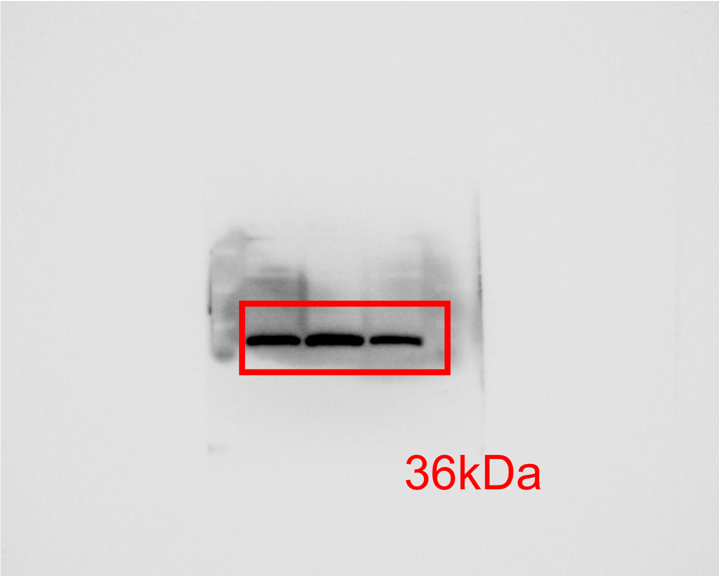

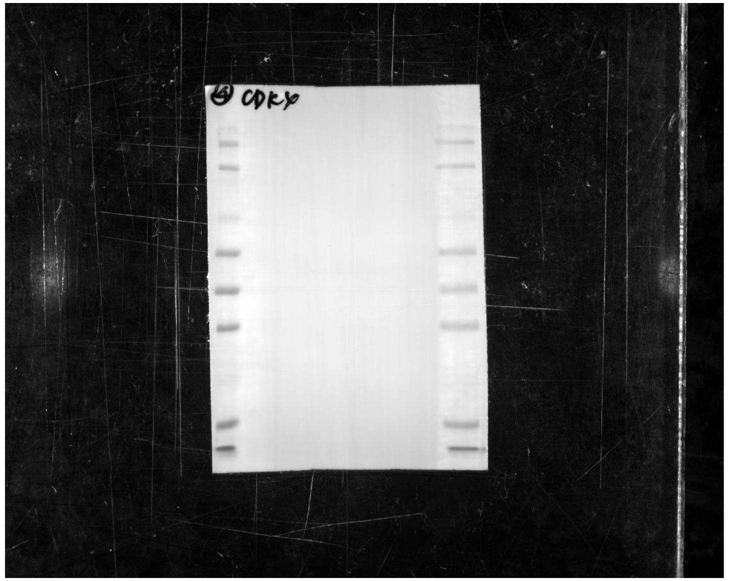

A549 cdk4

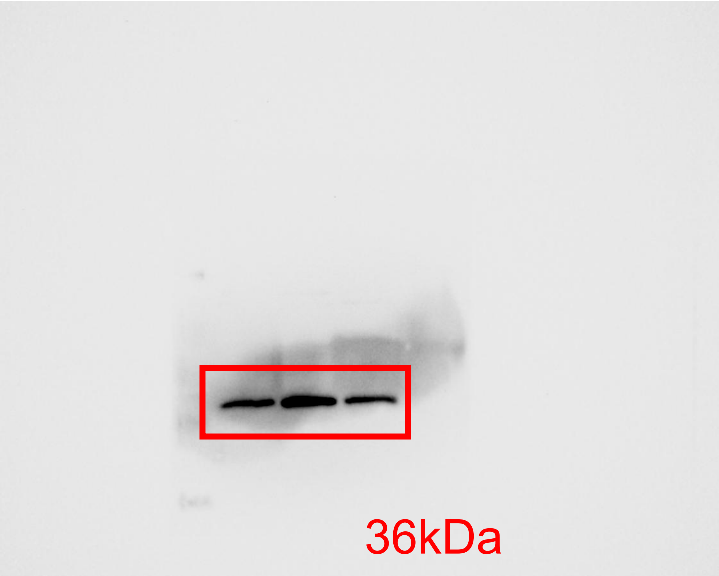

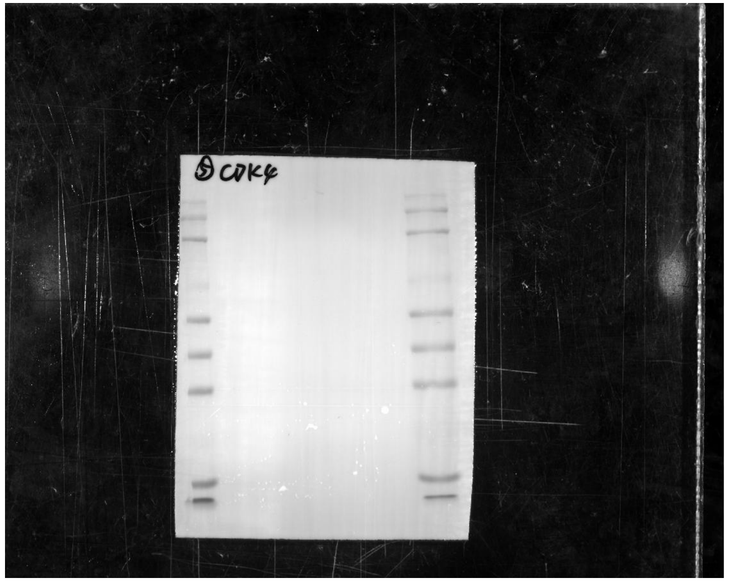

H1299 cdk6

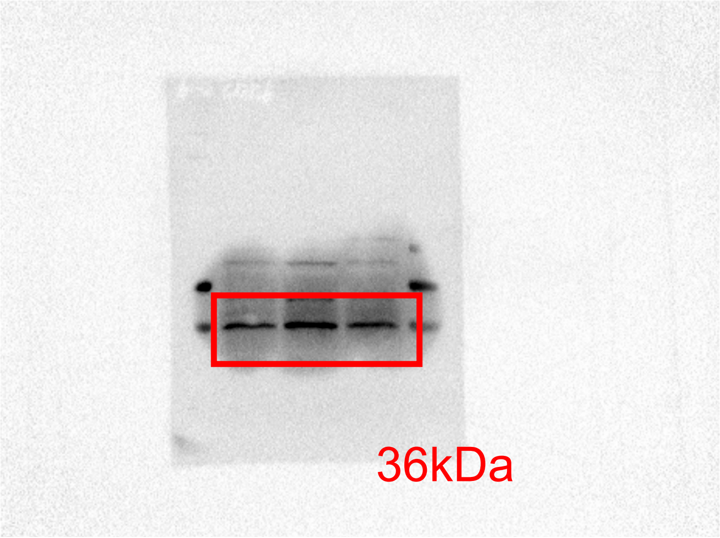

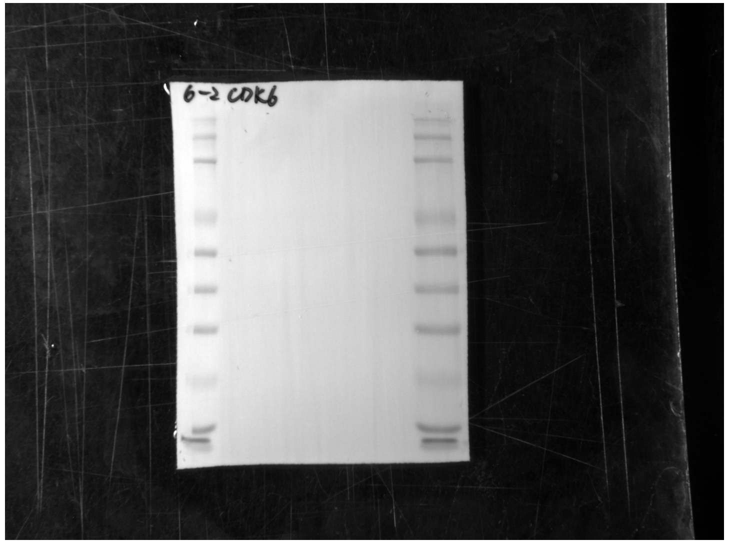

A549 cdk6

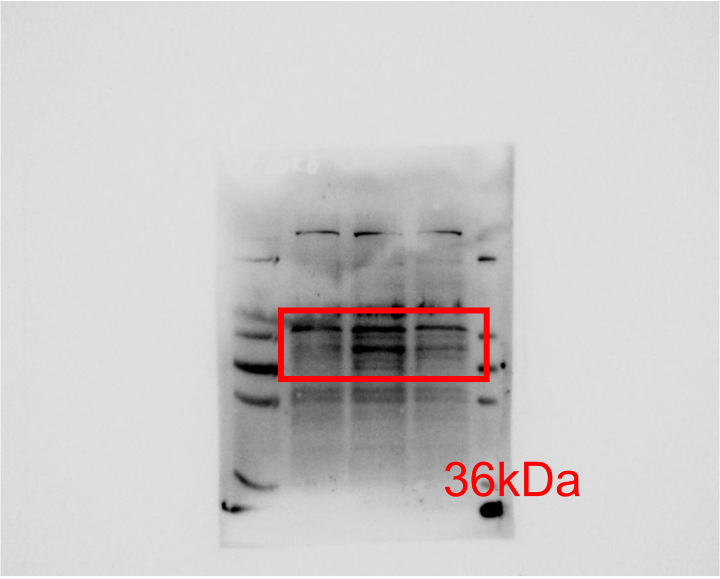

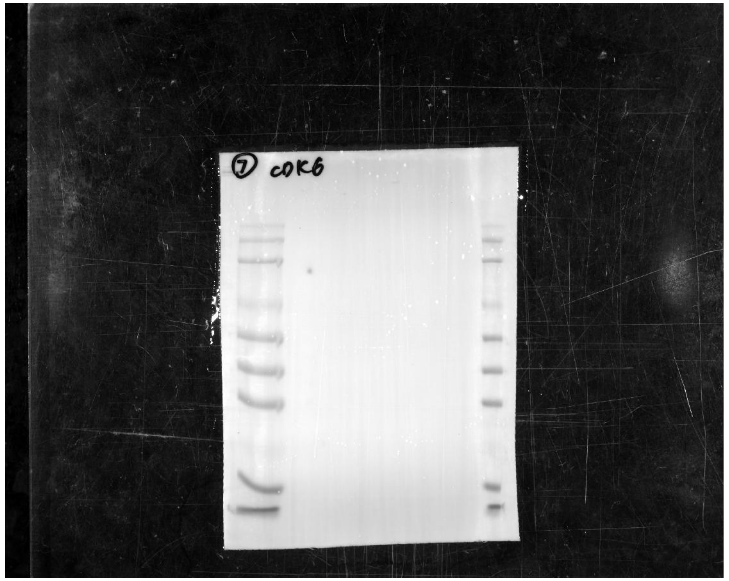

H1299 E-ca

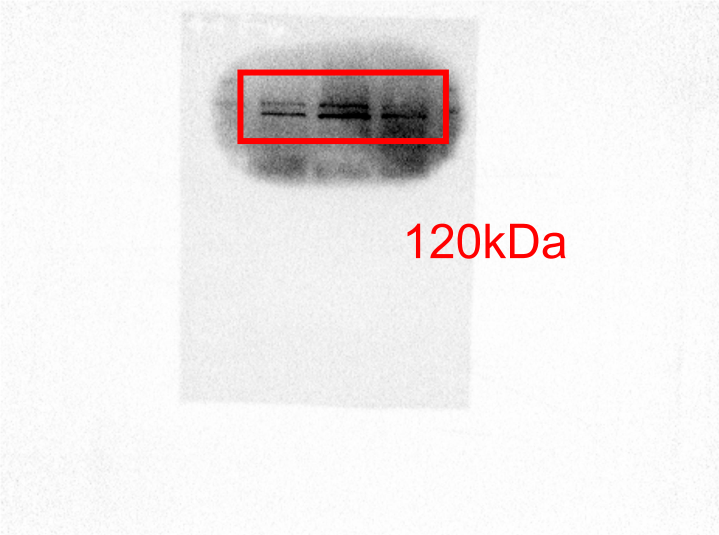

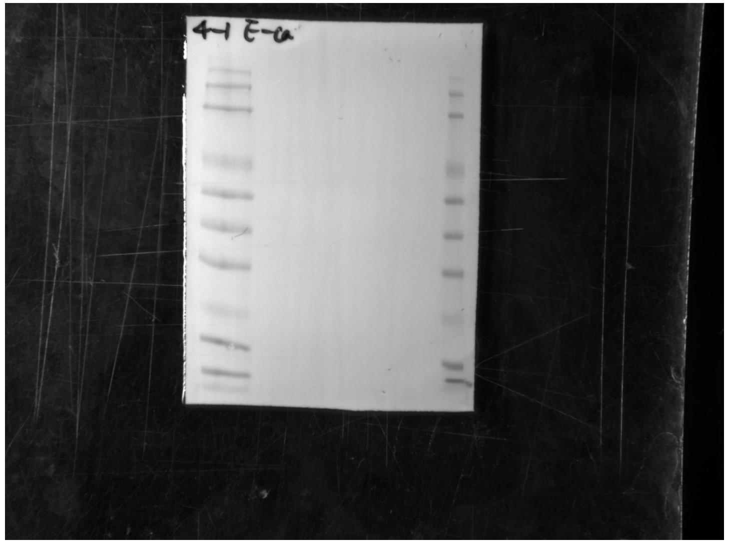

A549 E-ca

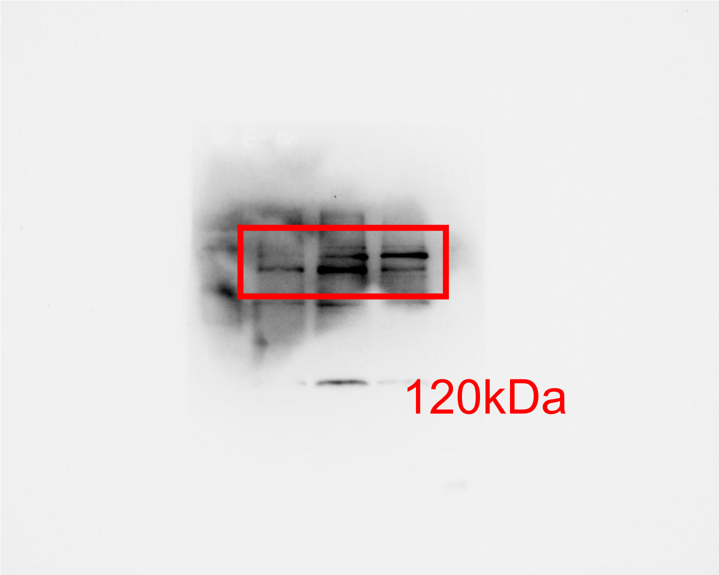

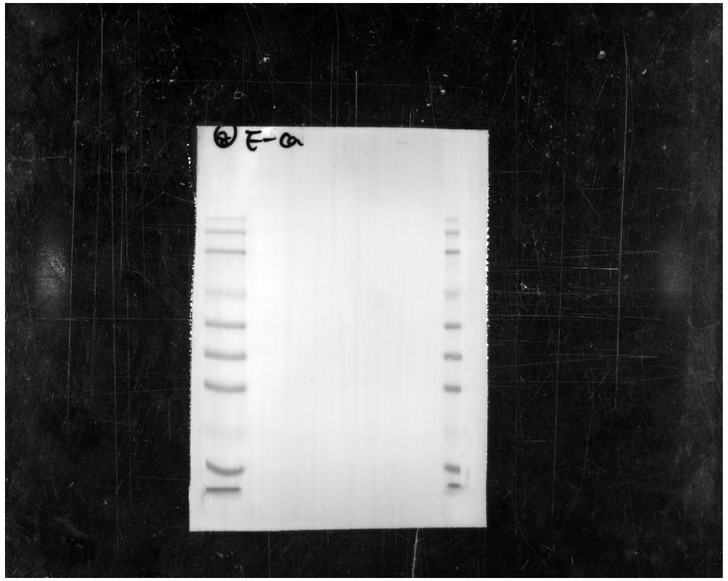

H1299 N-ca

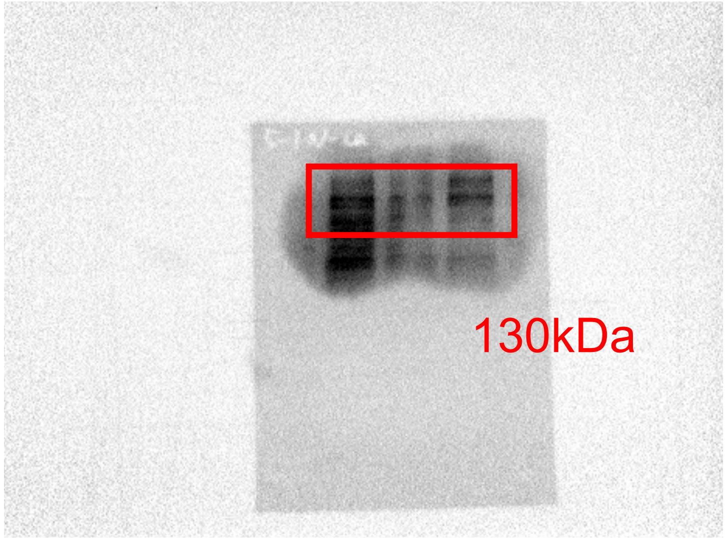

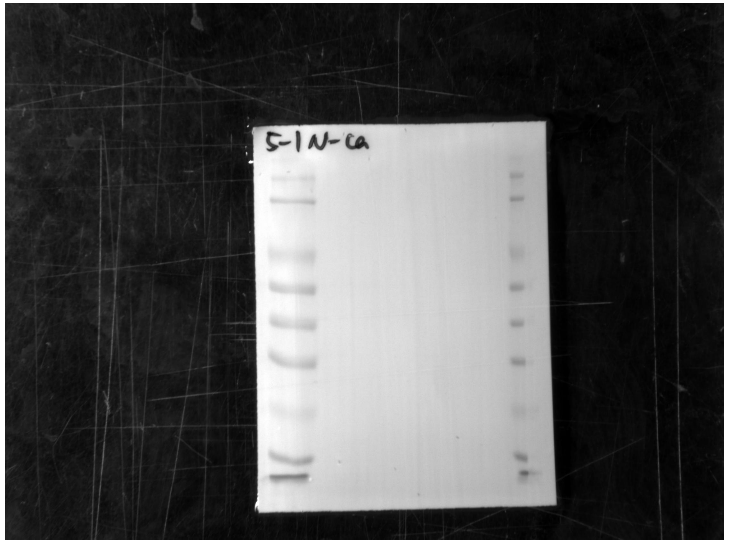

A549 N-ca

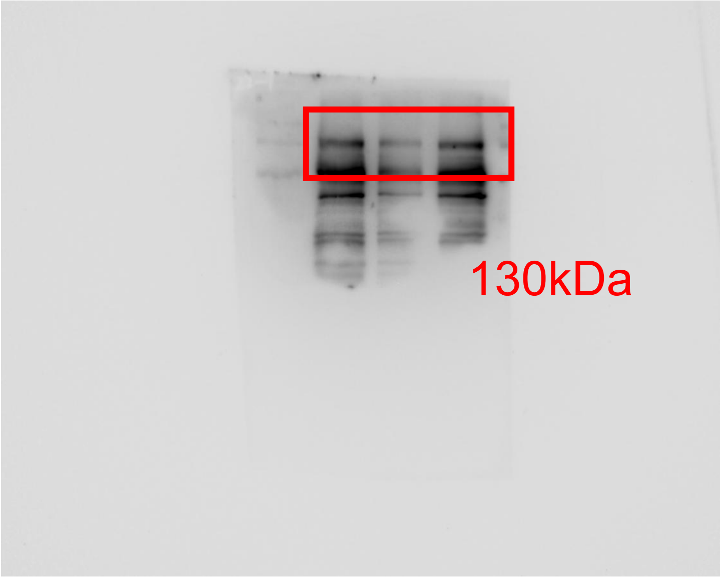

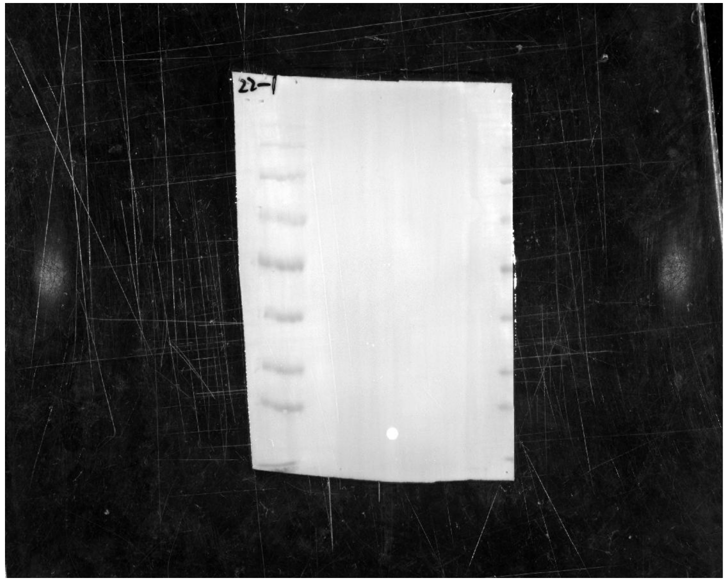

H1299 mmp9

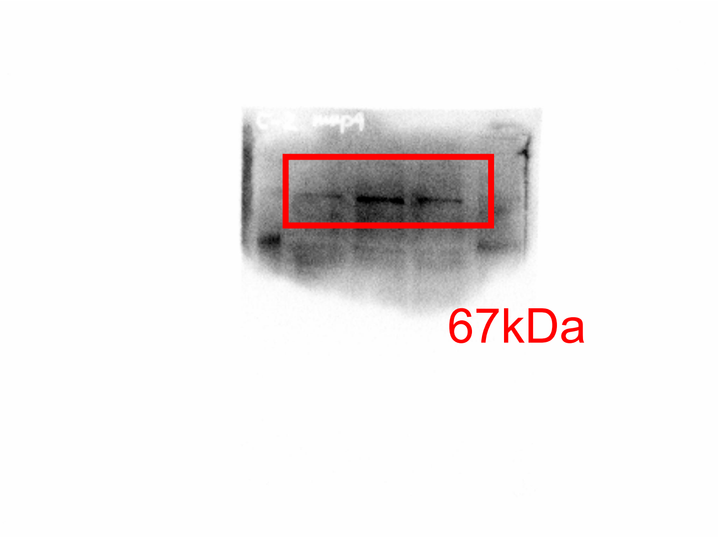

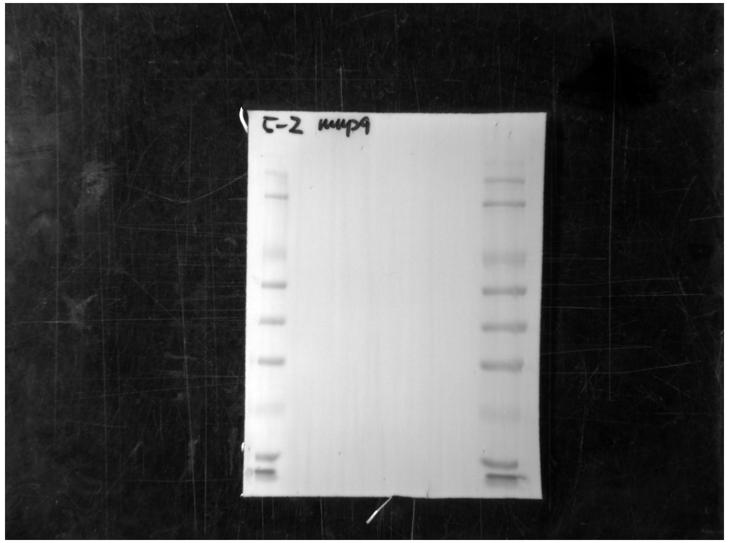

H1299 mmp9

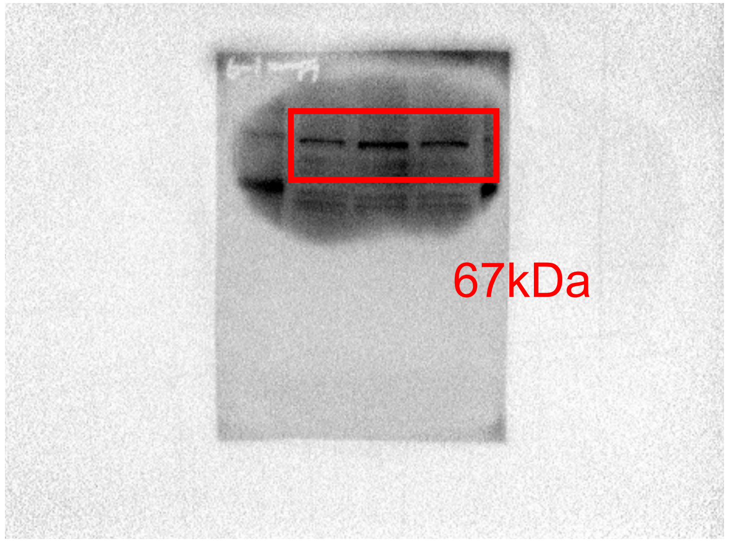

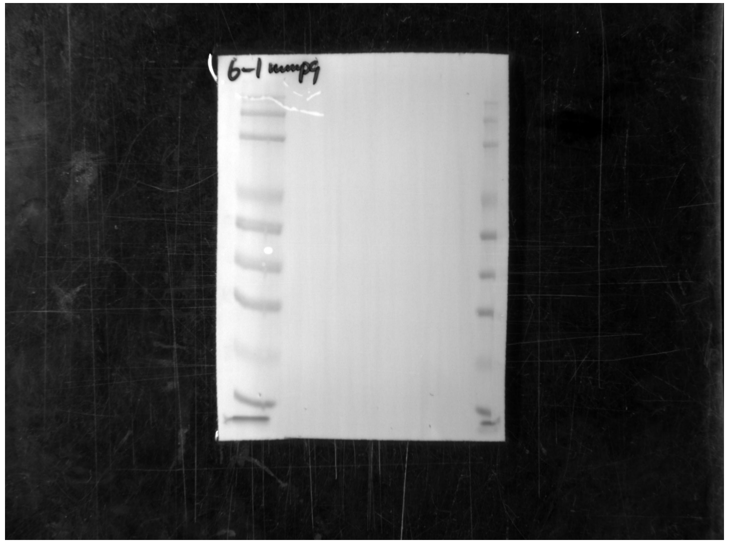

H1299 β-actin

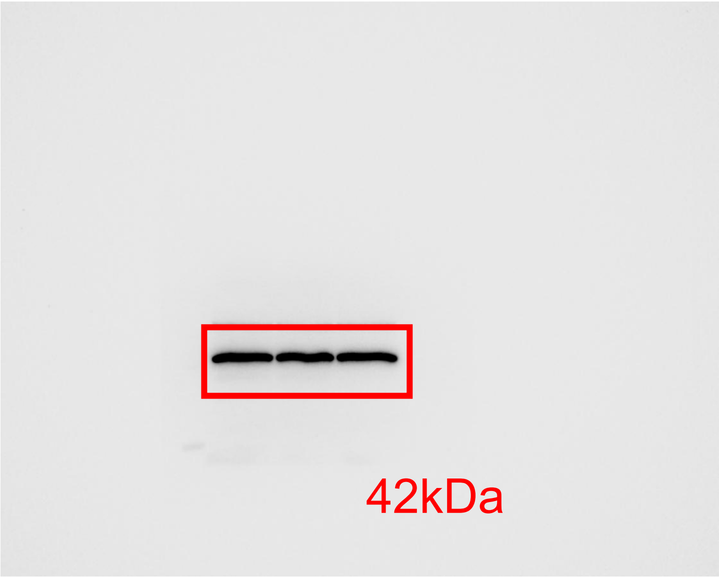

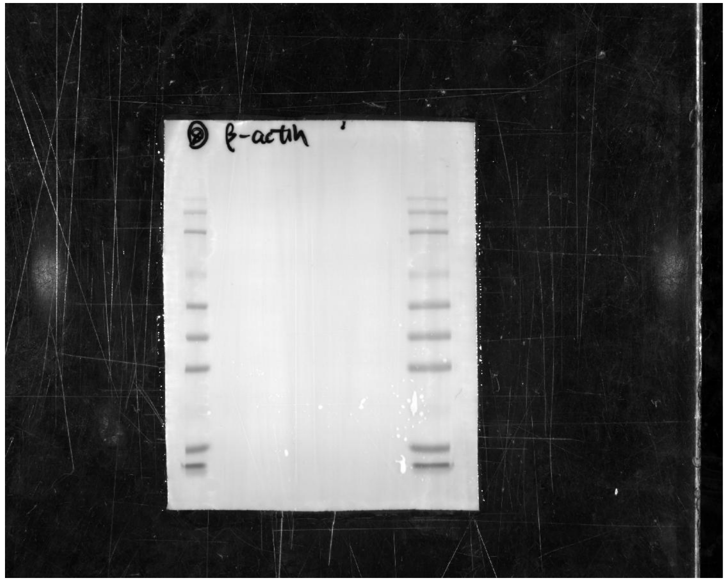

H1299 β-actin

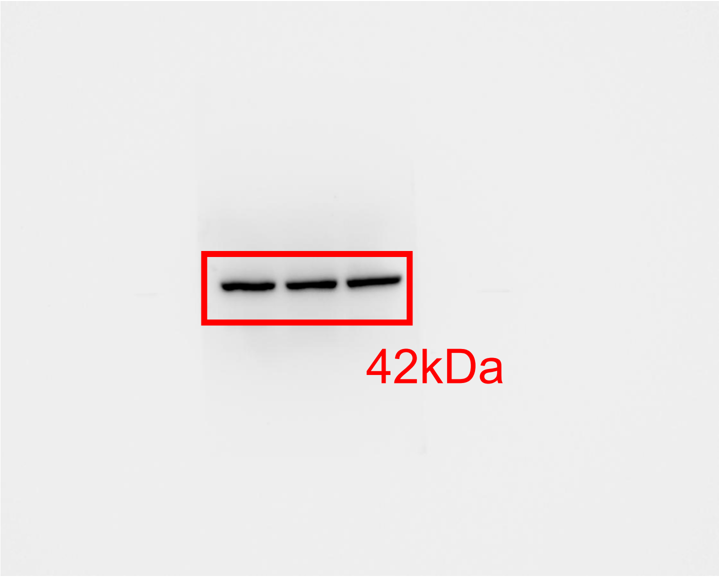

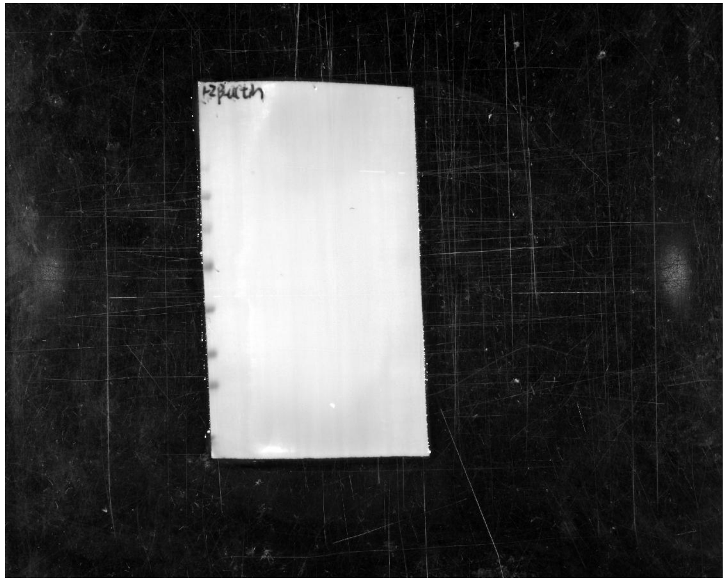

S-FIG 8

A549 ZC3H15

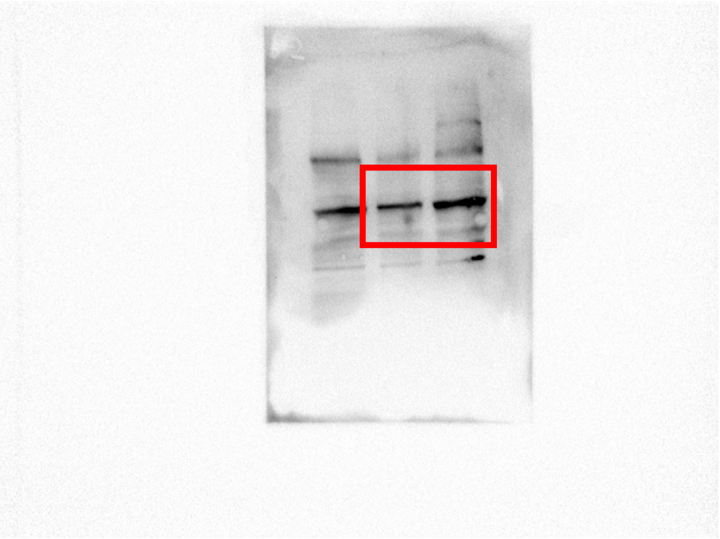

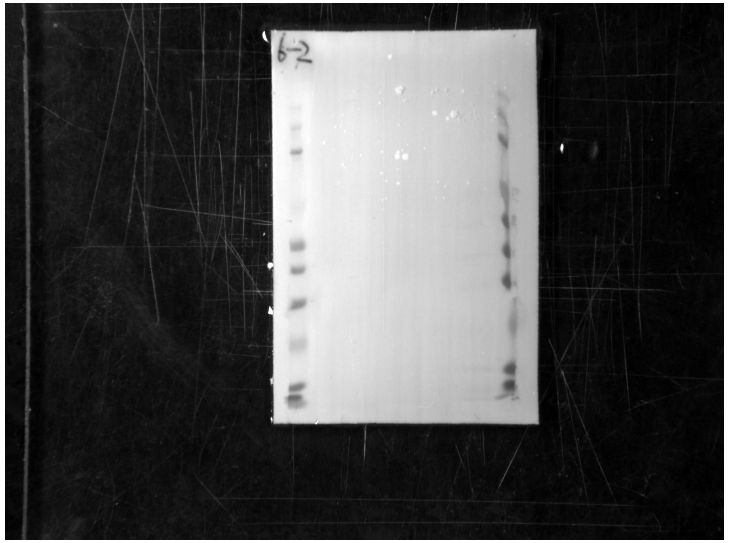

A549 p-pten

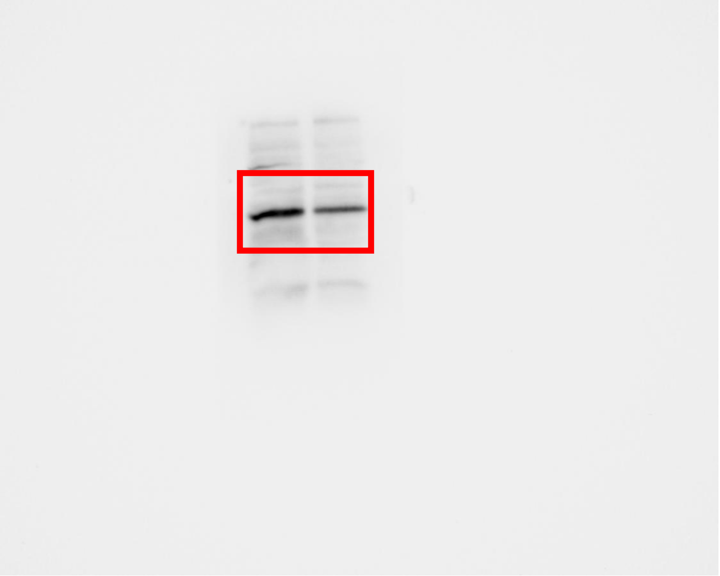

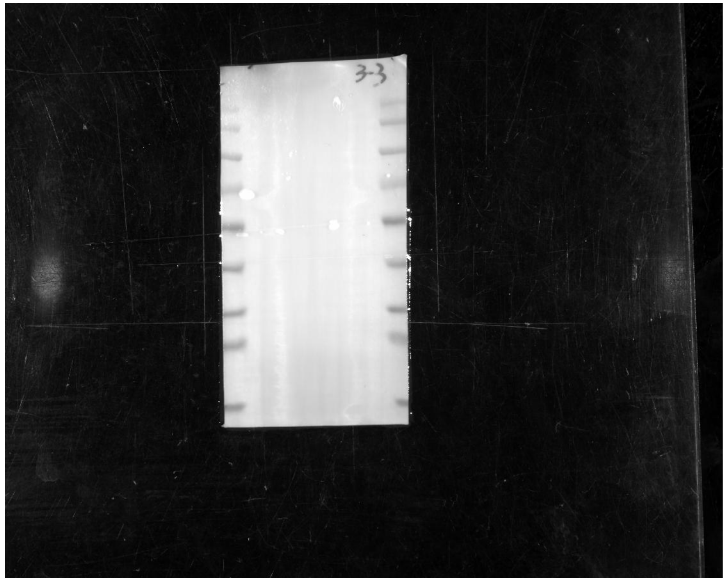

A549 pten

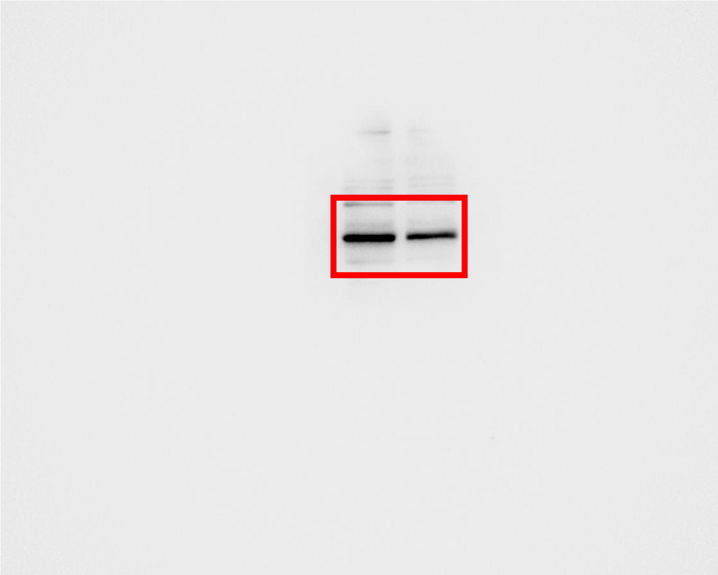

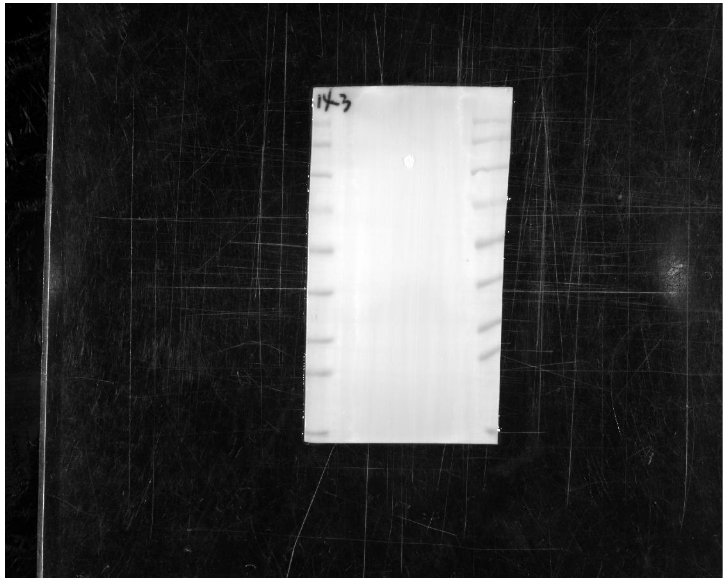

A549 β-actin

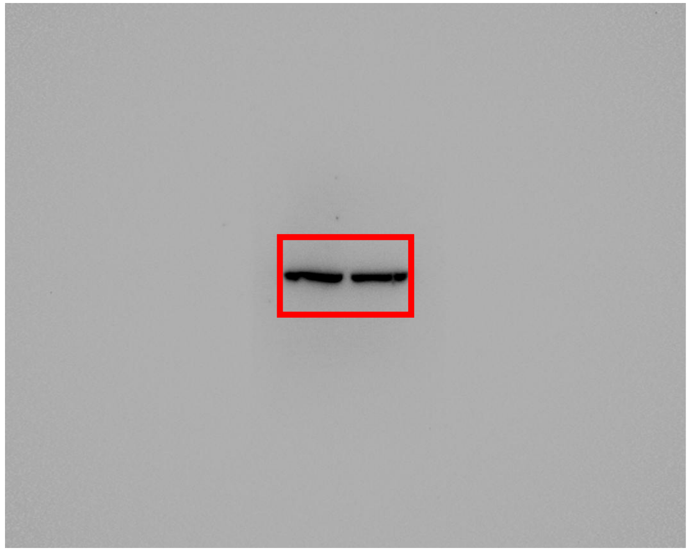

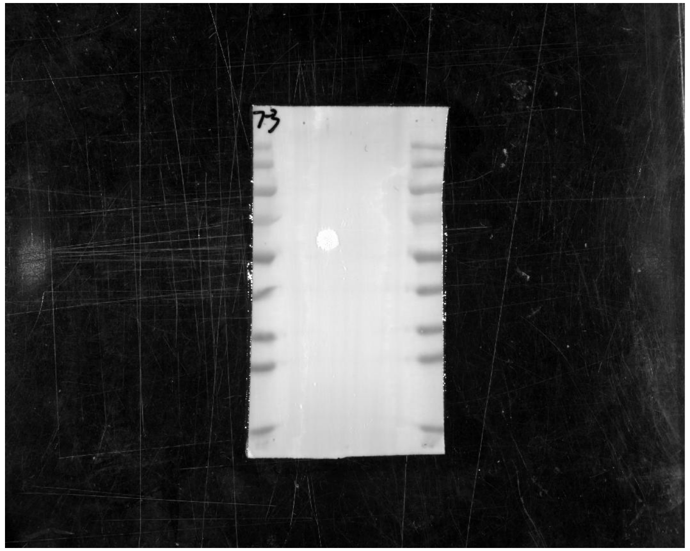

H1299 ZC3H15

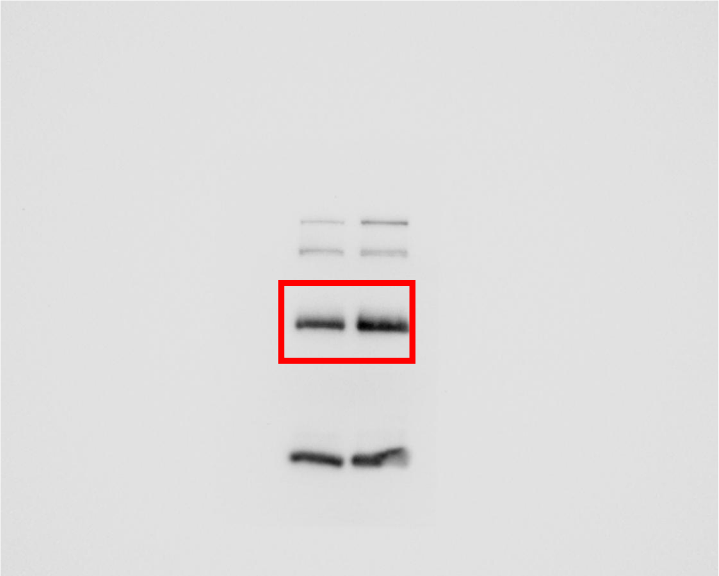

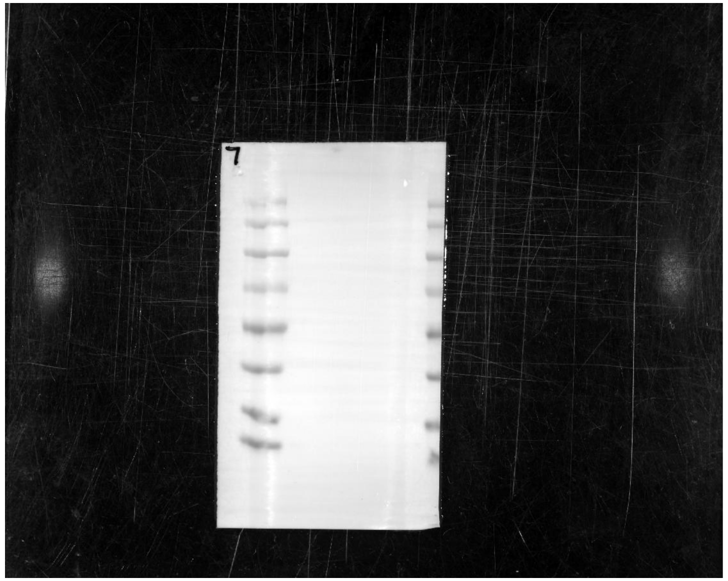

H1299 P-PTEN

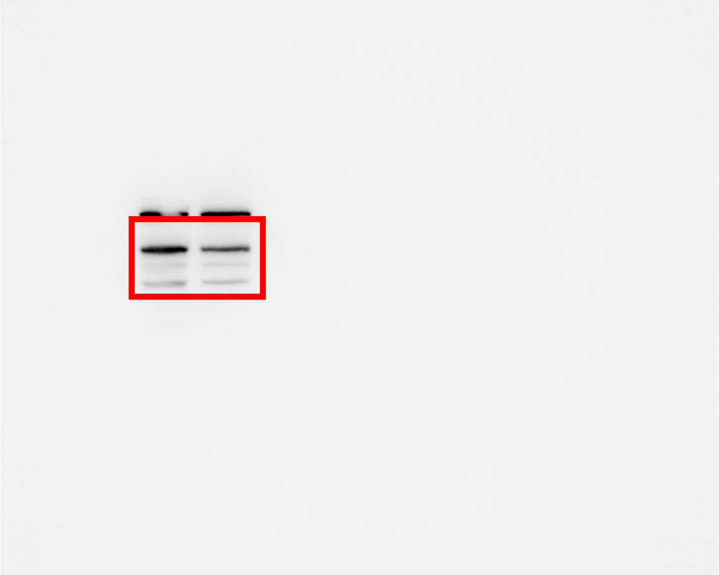

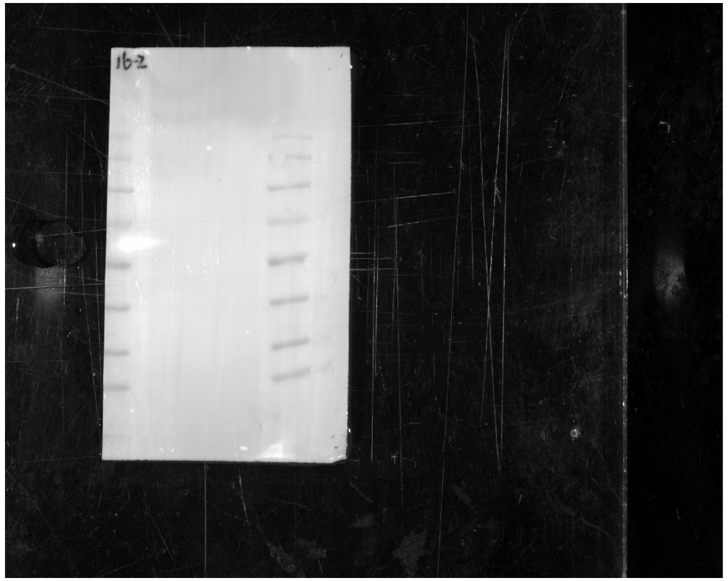

H1299 PTEN

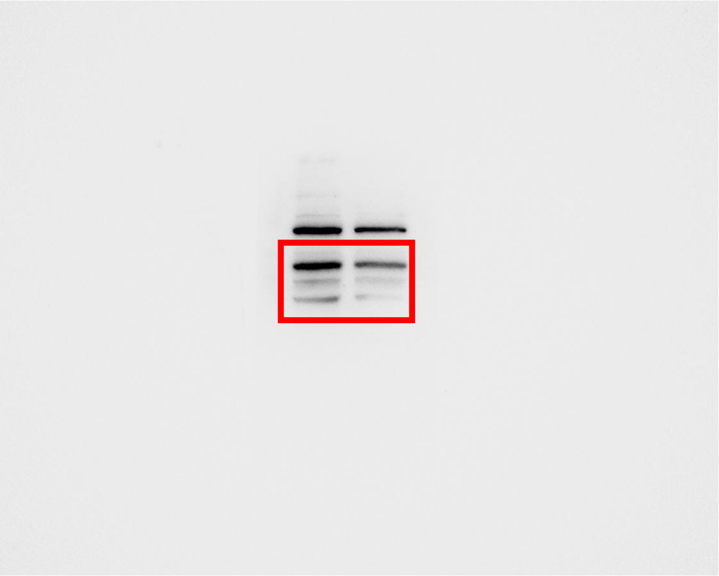

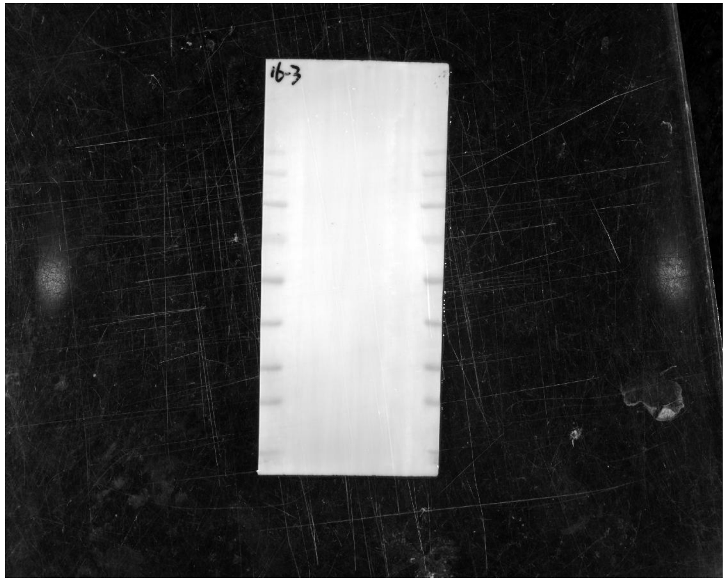

H1299 β-actin

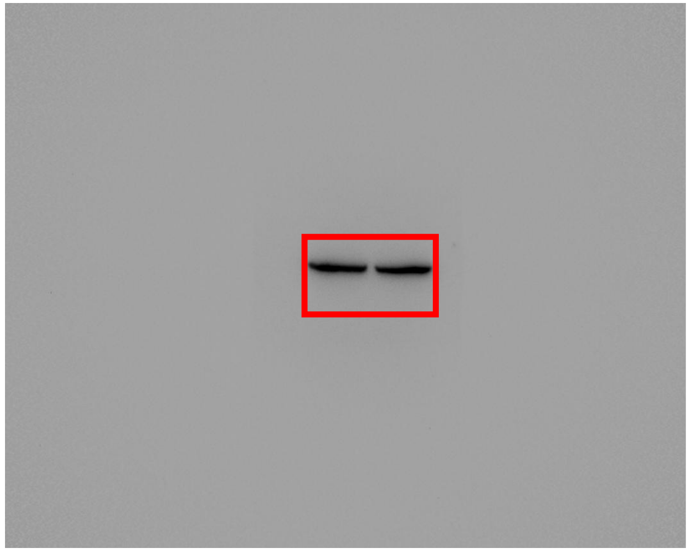

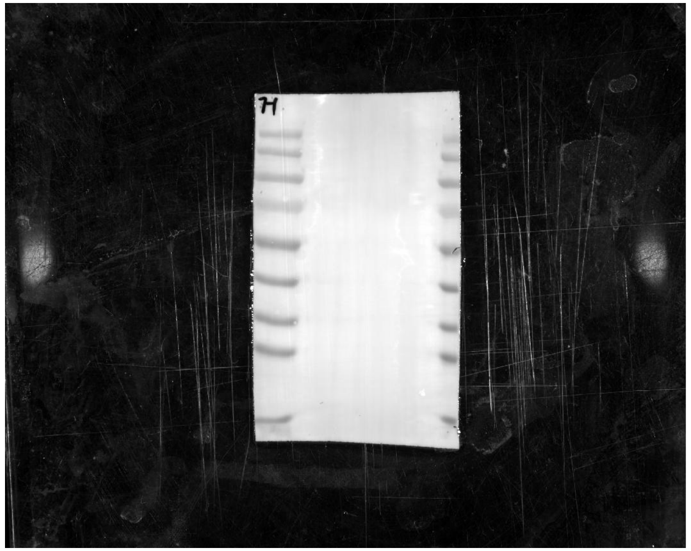

A549 ZC3H15

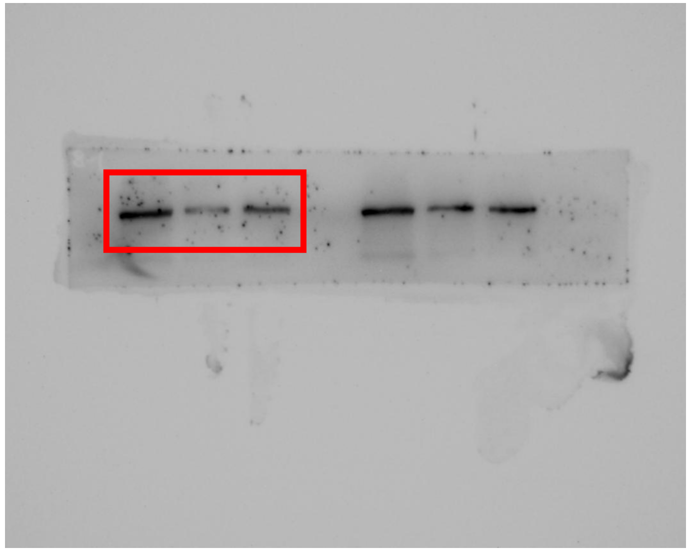

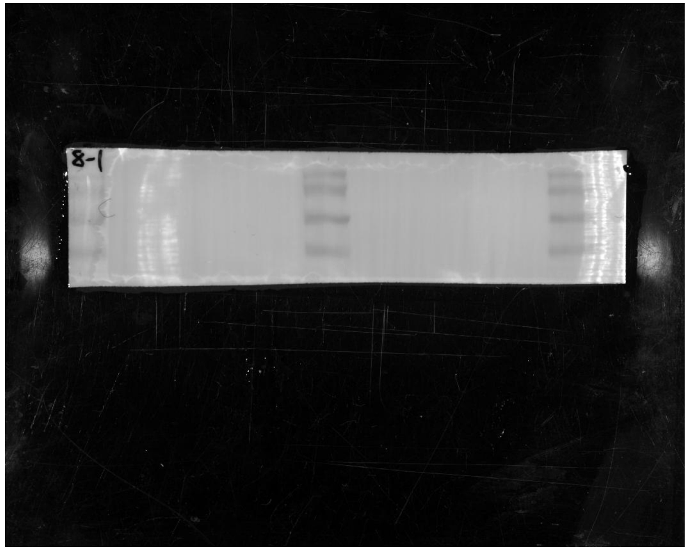

A549 p-pten

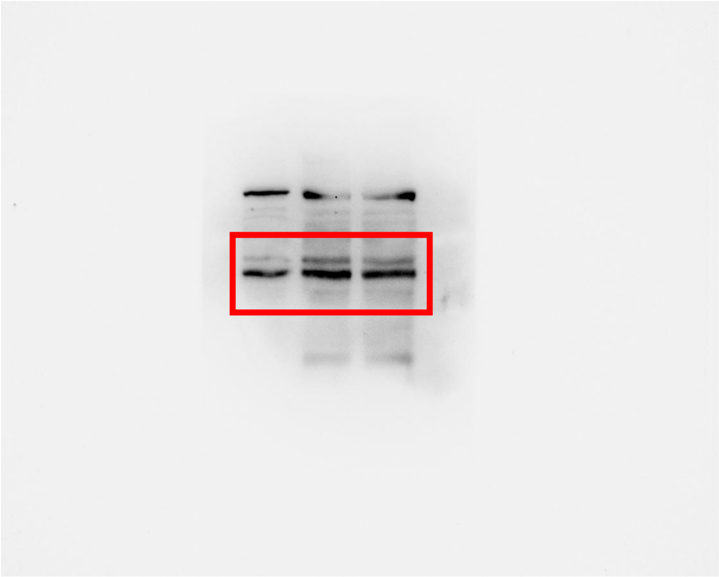

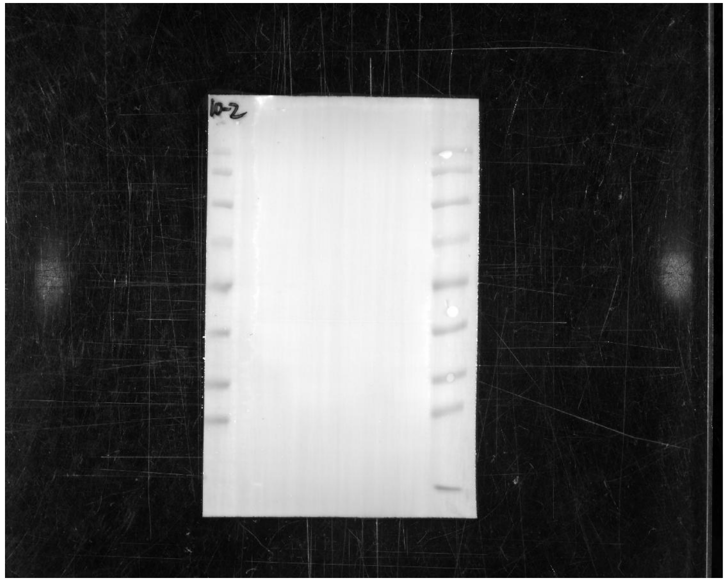

A549 pten

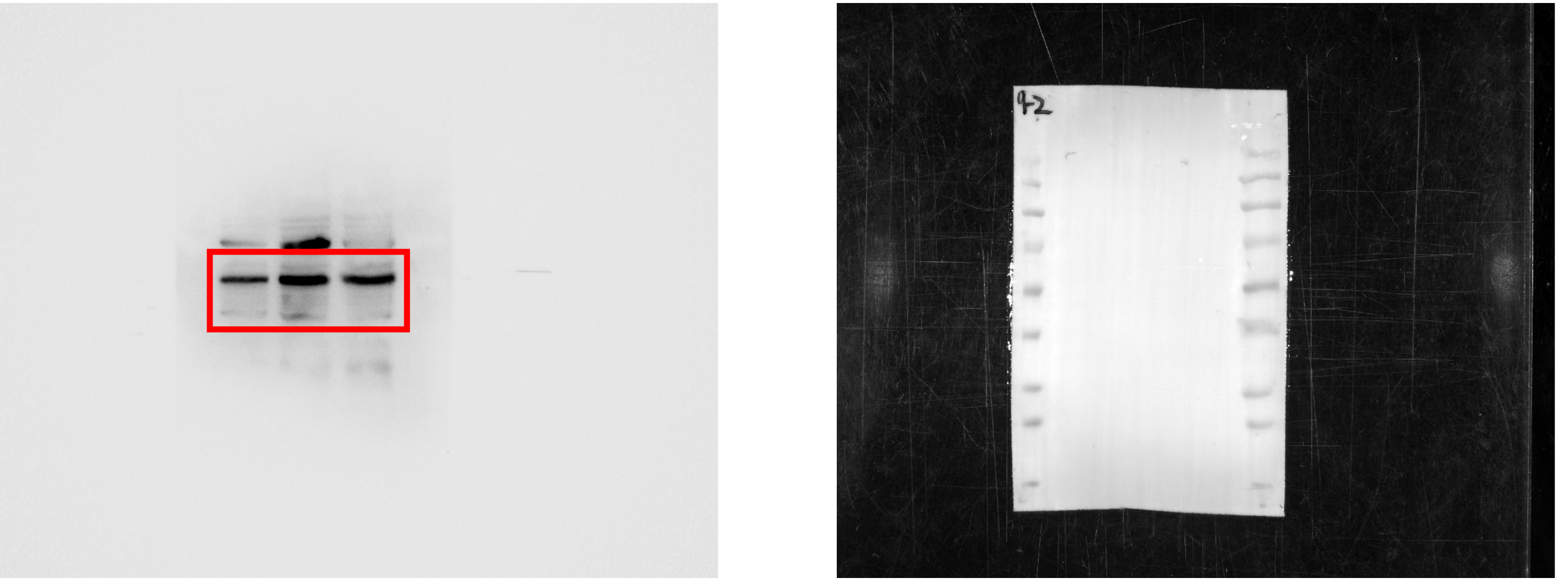

A549 β-actin

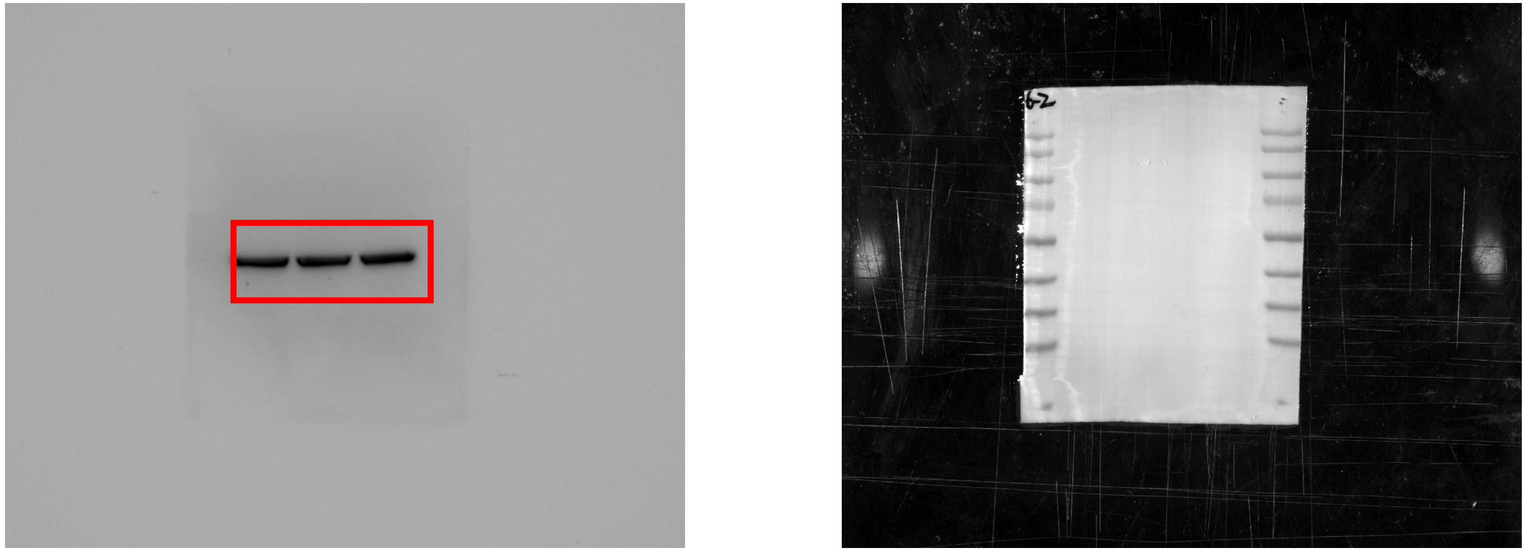

H1299 ZC3H15

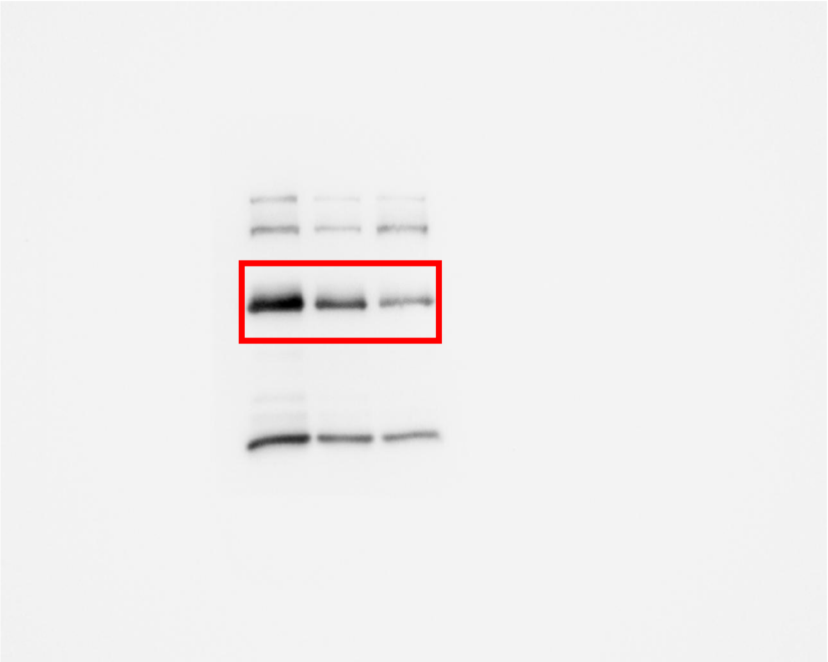

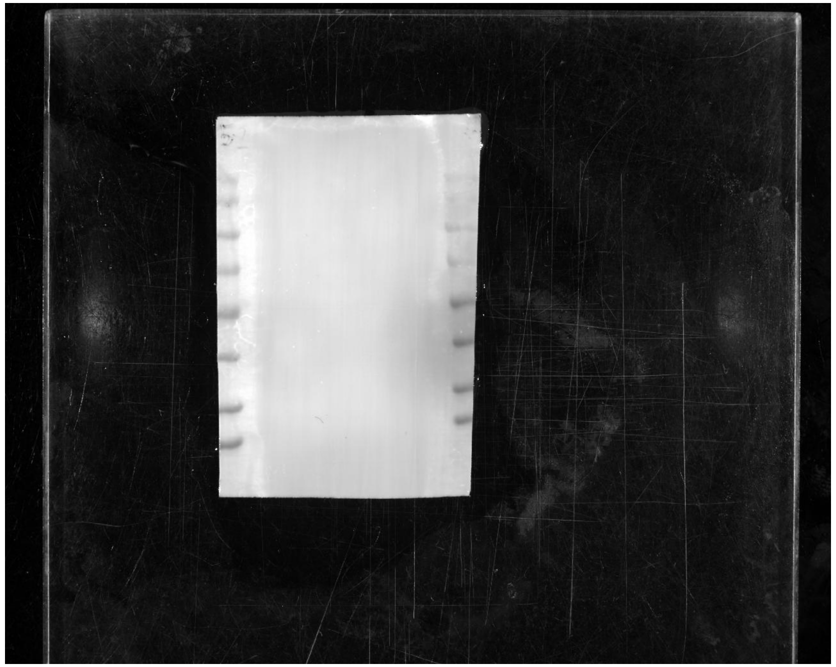

H1299 p-pten

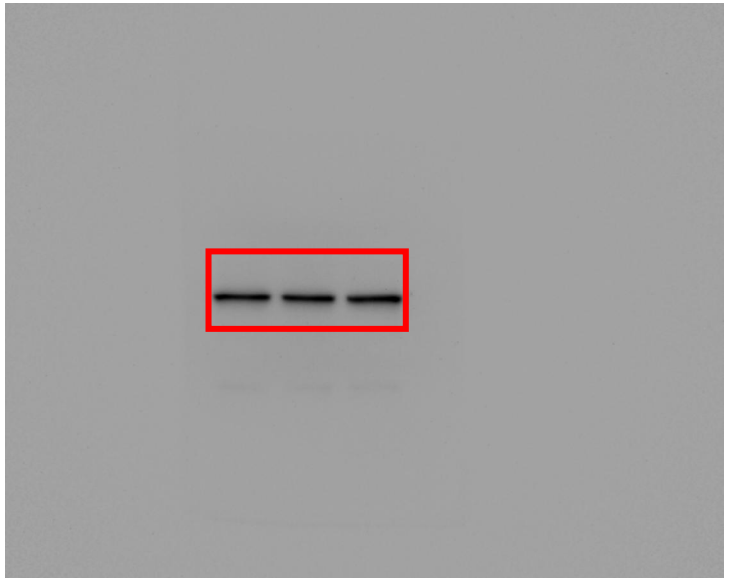

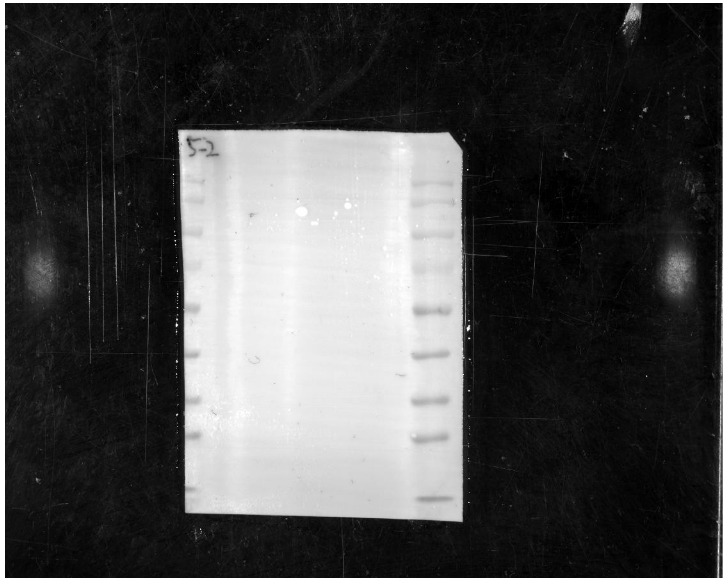

H1299 pten

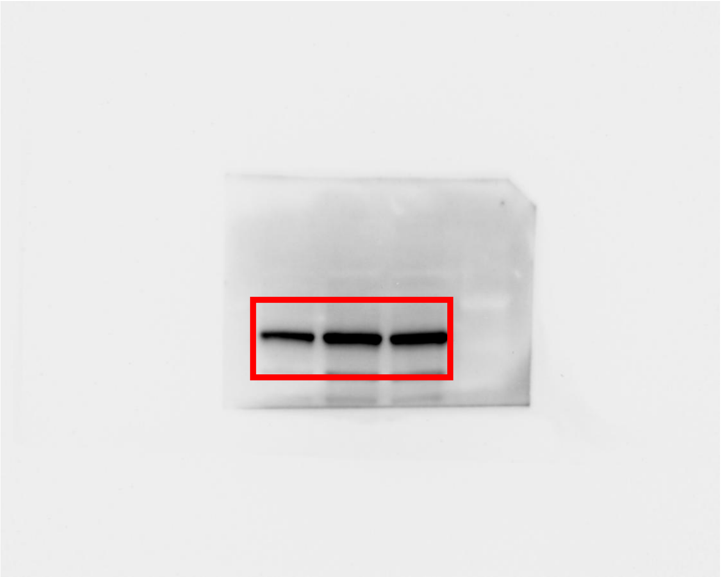

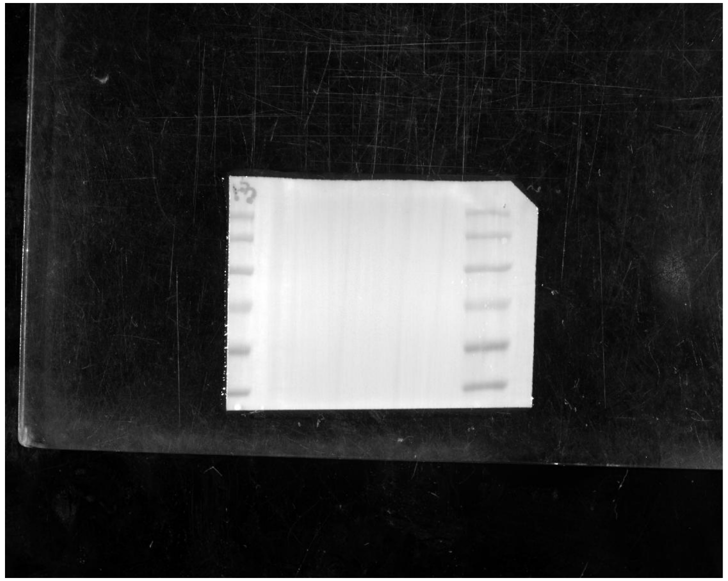

H1299 pten

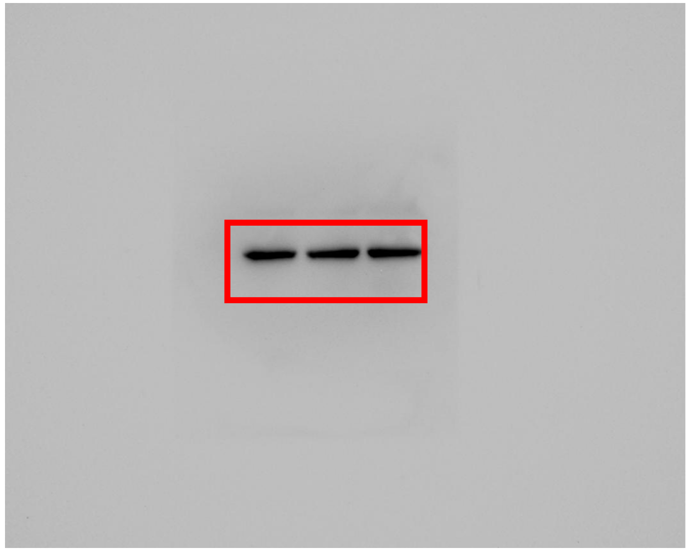

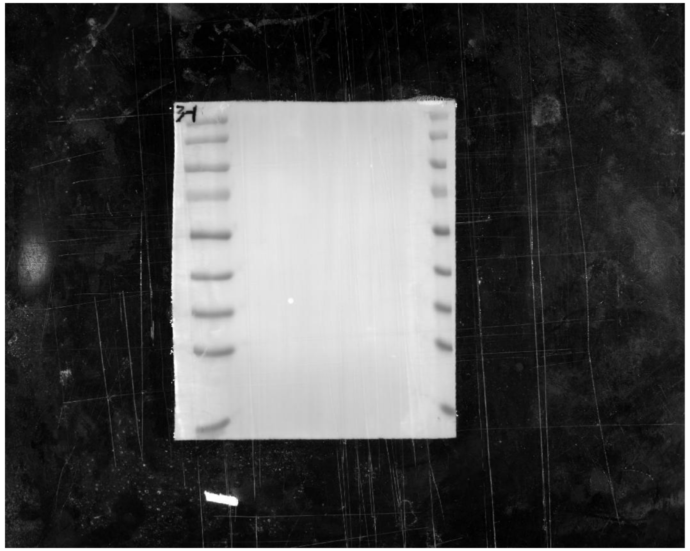

A549 ZC3H15

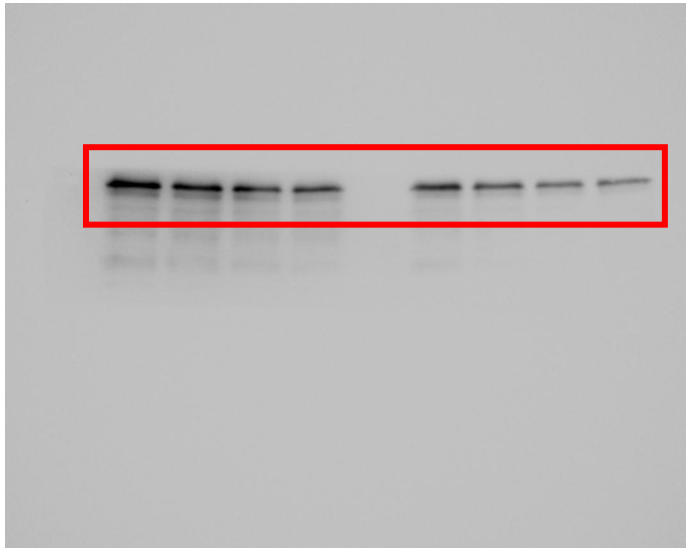

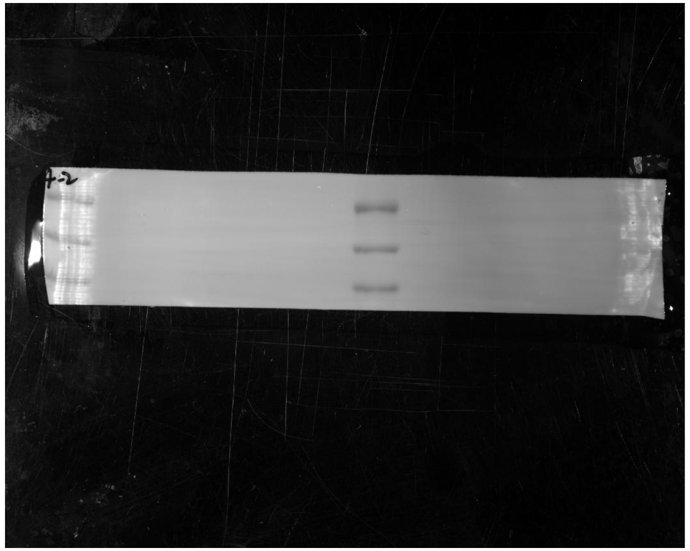

A549 PTEN

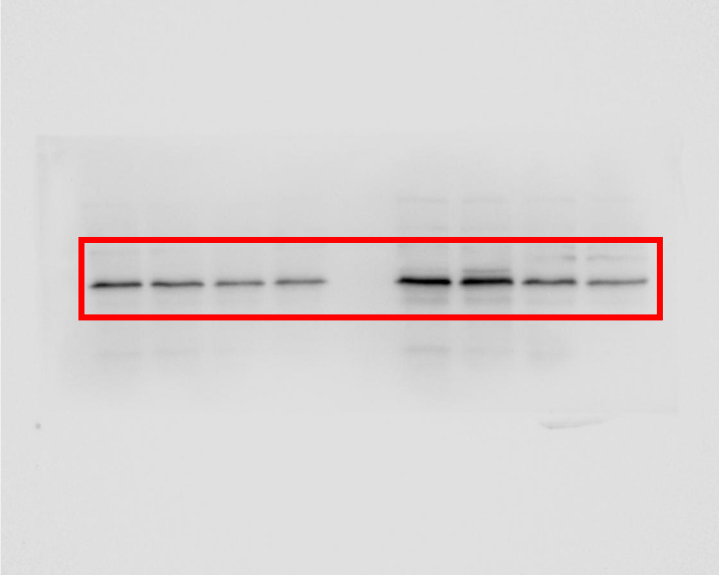

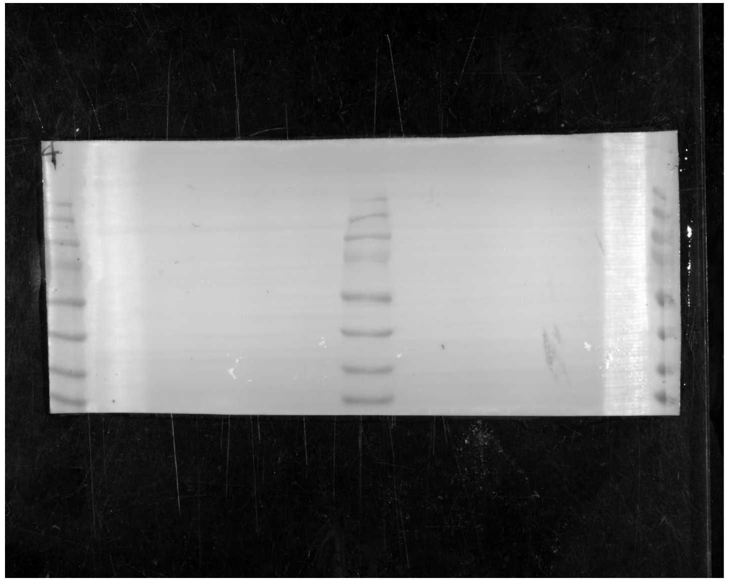

A549 β-actin

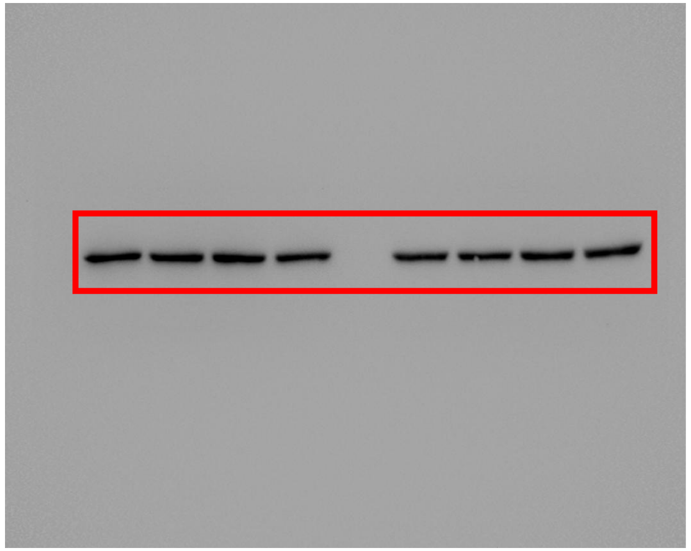

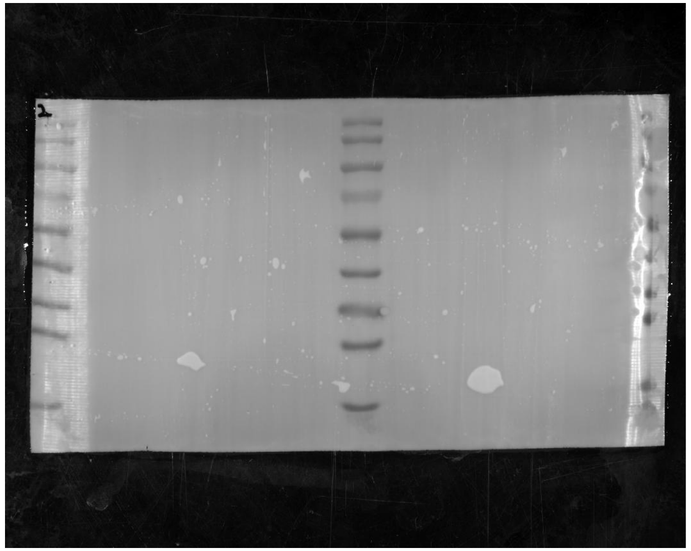

H1299 ZC3H15

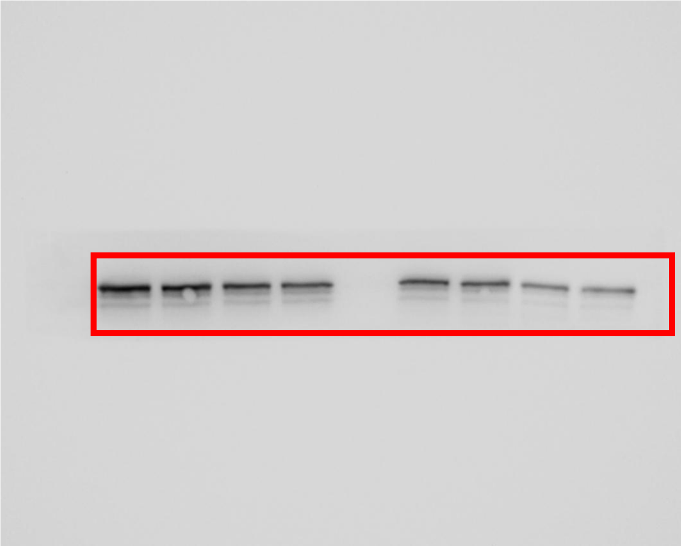

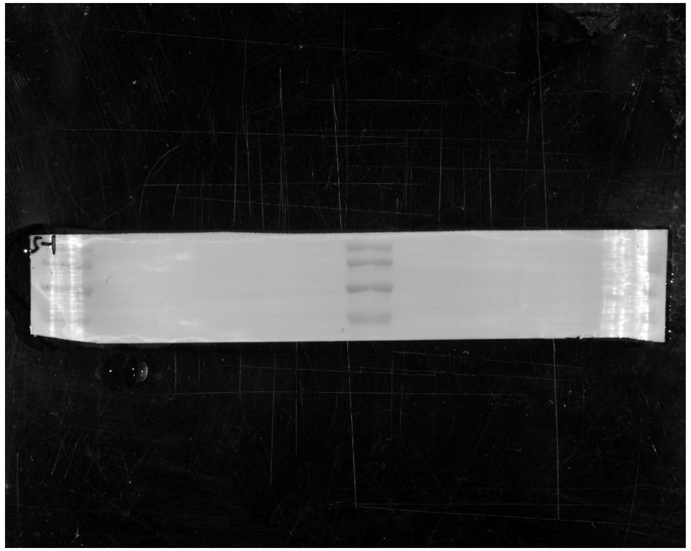

H1299 PTEN

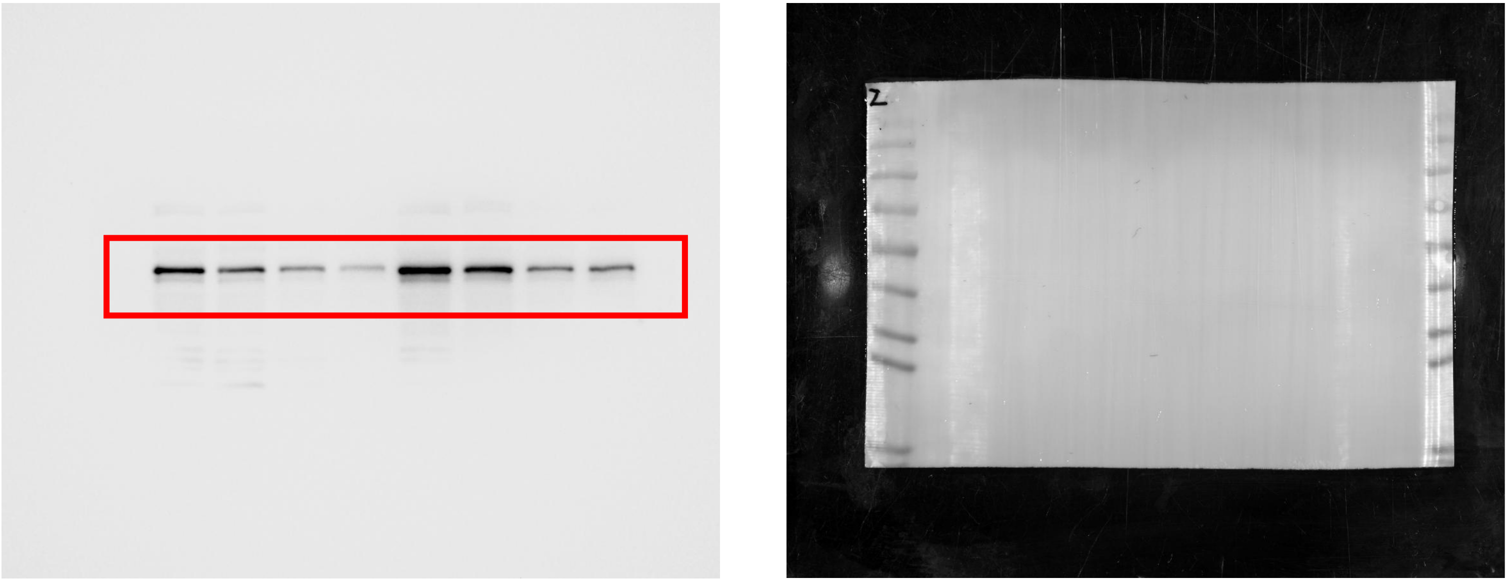

H1299 β-actin

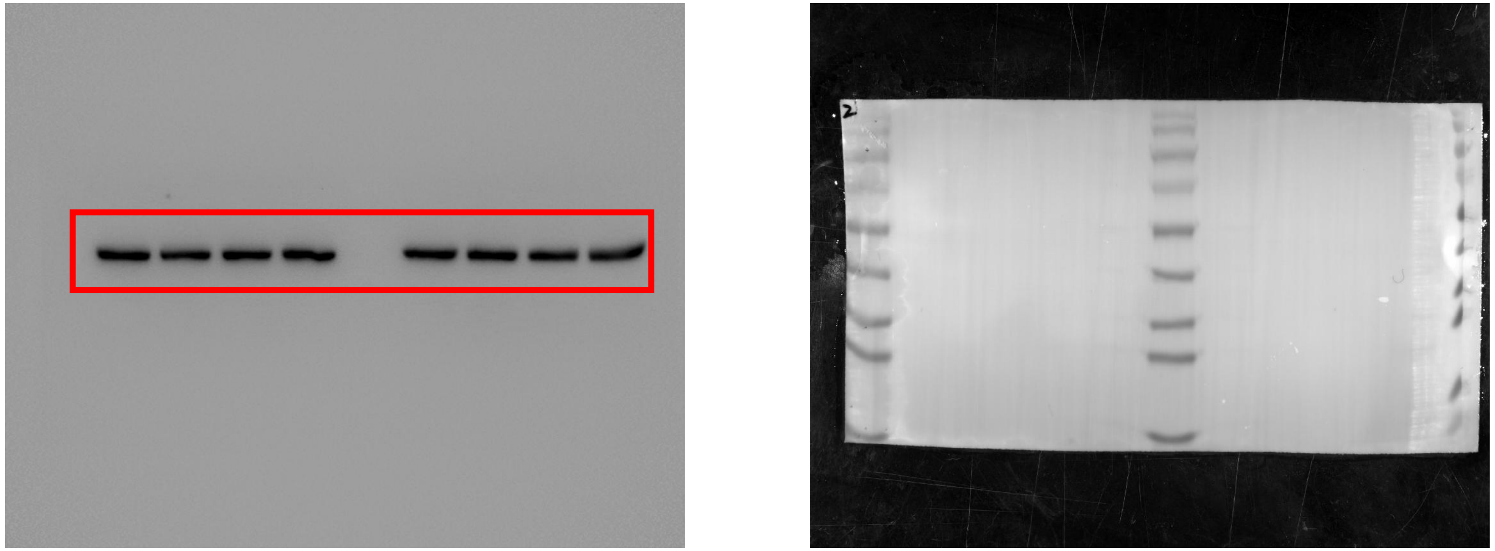

FIG 6

A549 Ub

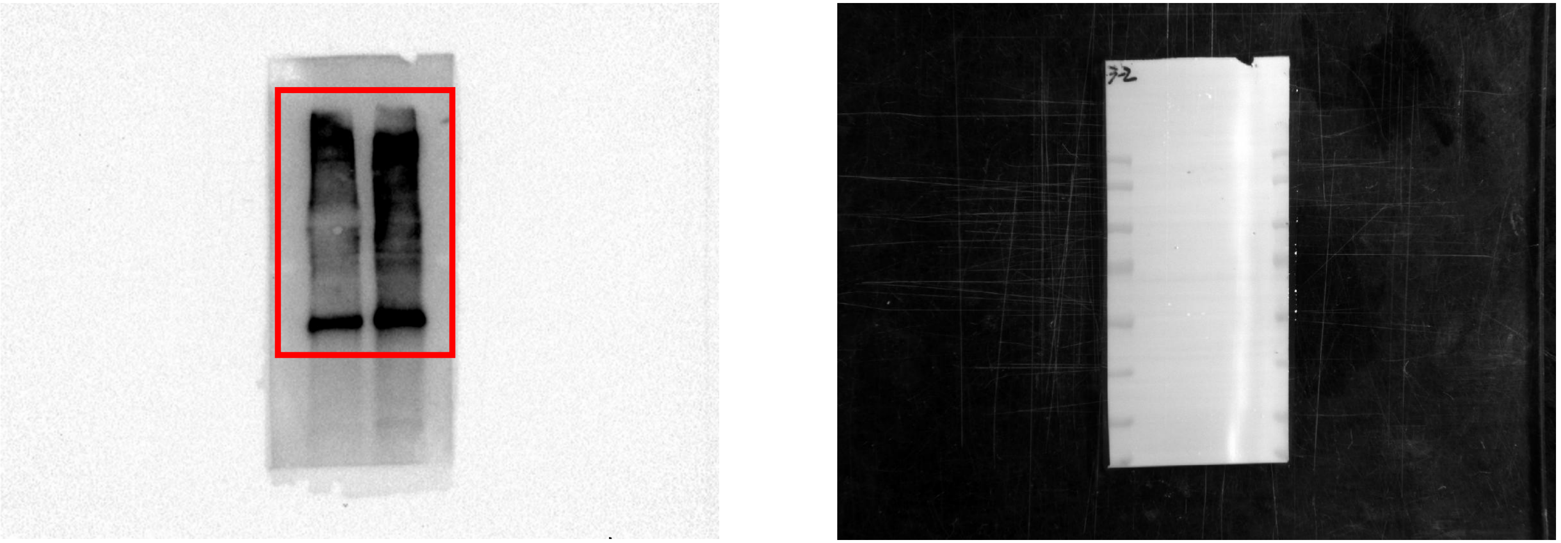

H1299 Ub

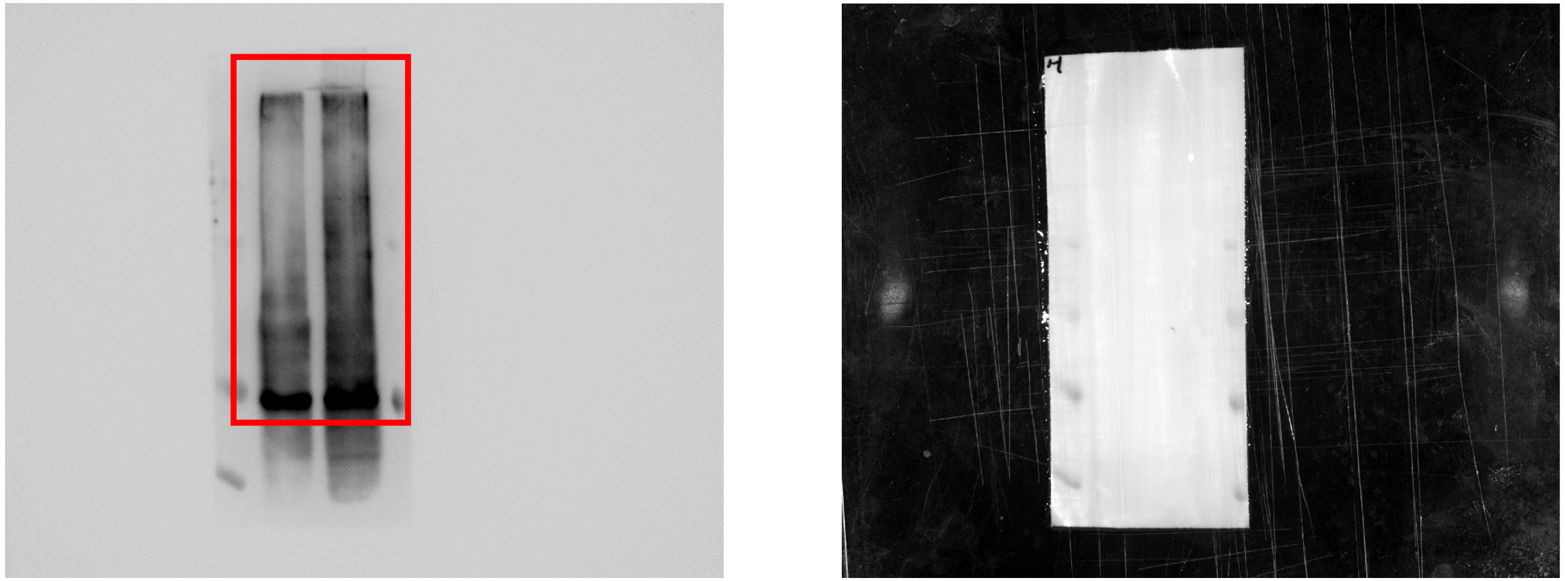

A549 zc-FLAG

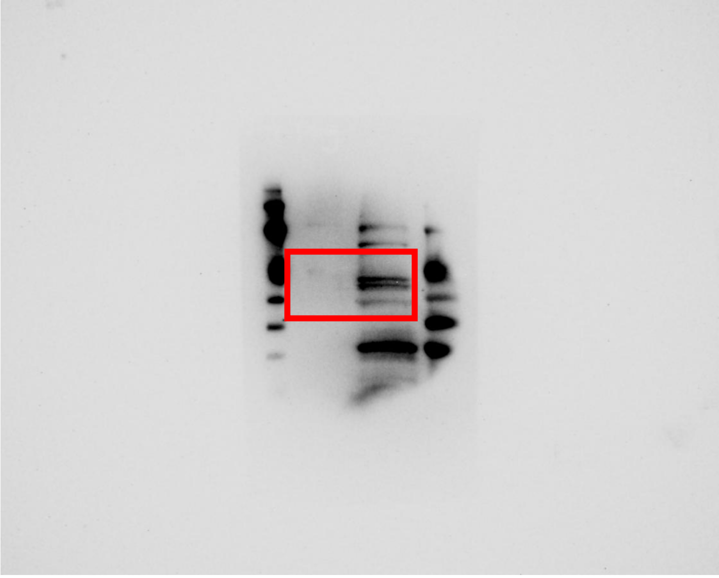

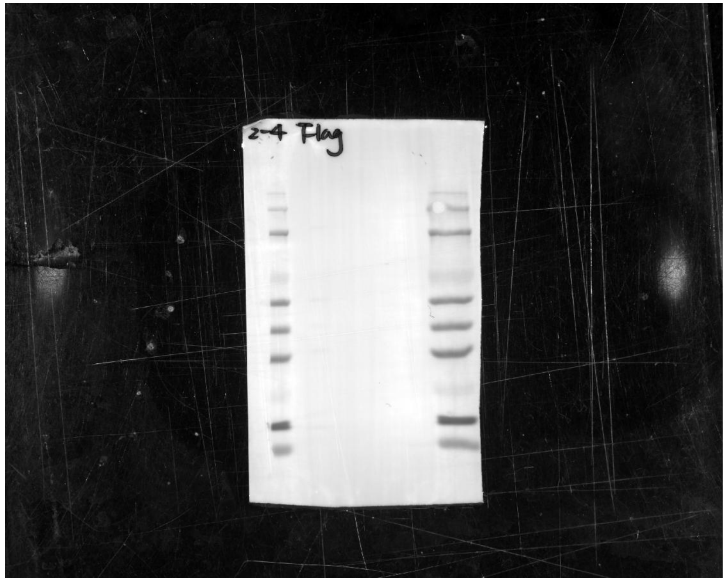

H1299 zc-FLAG

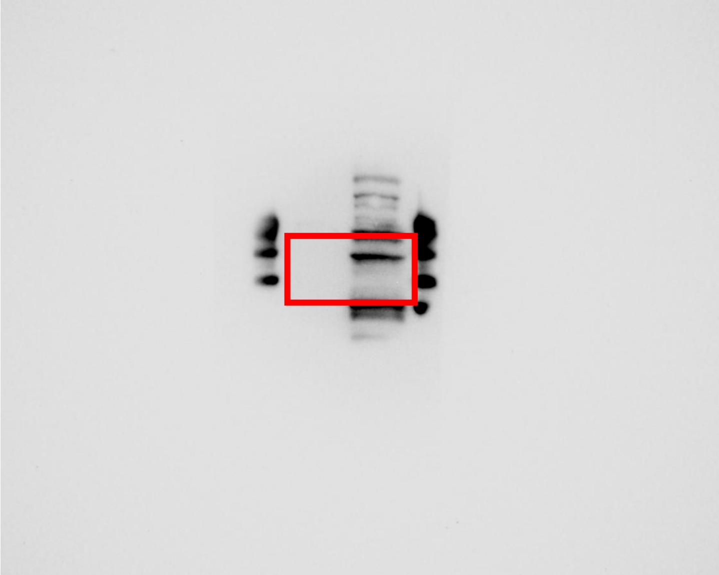

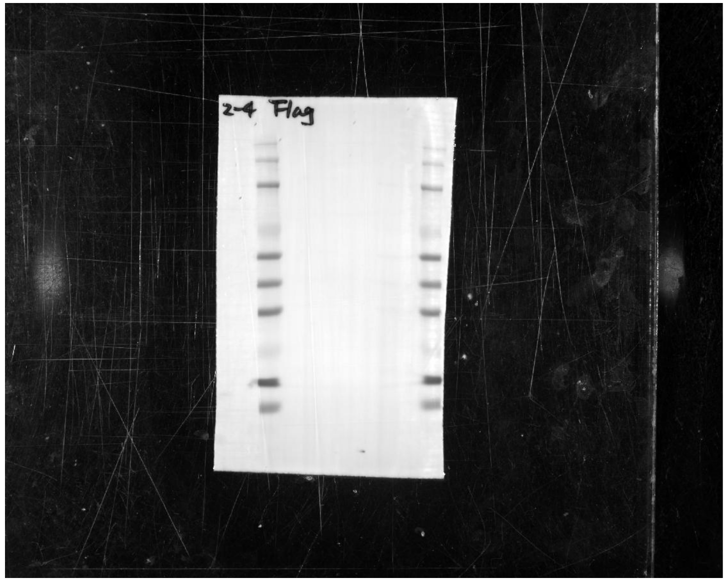

A549 PTEN

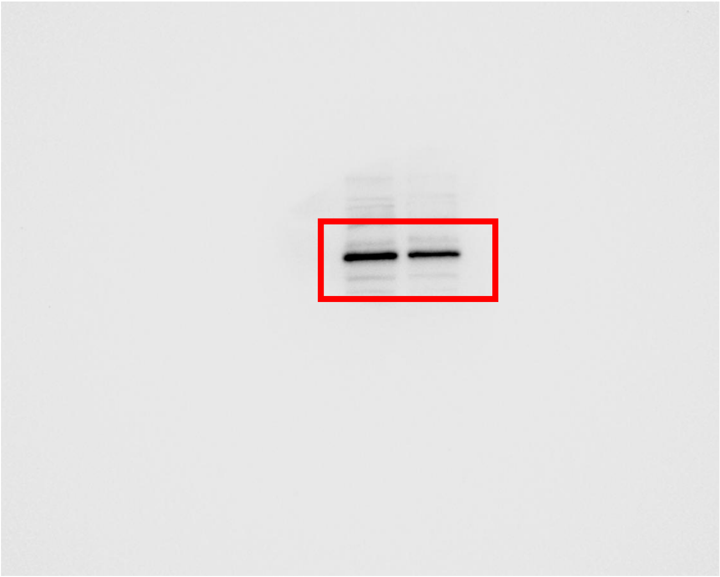

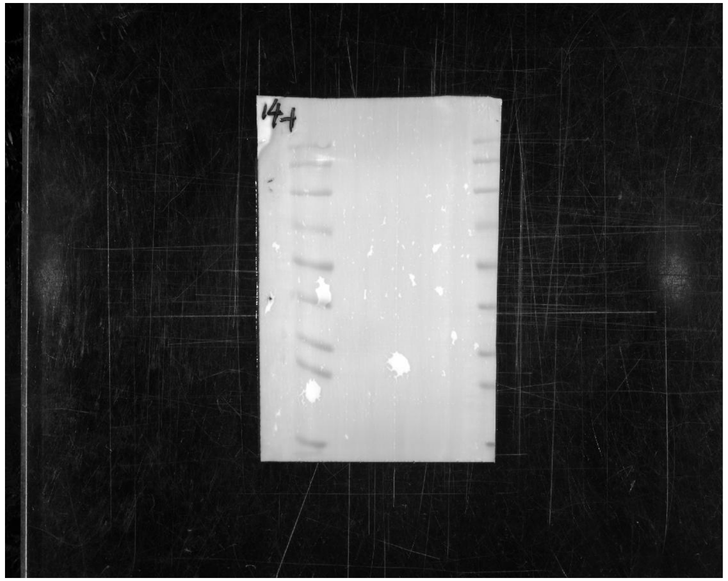

H1299 PTEN

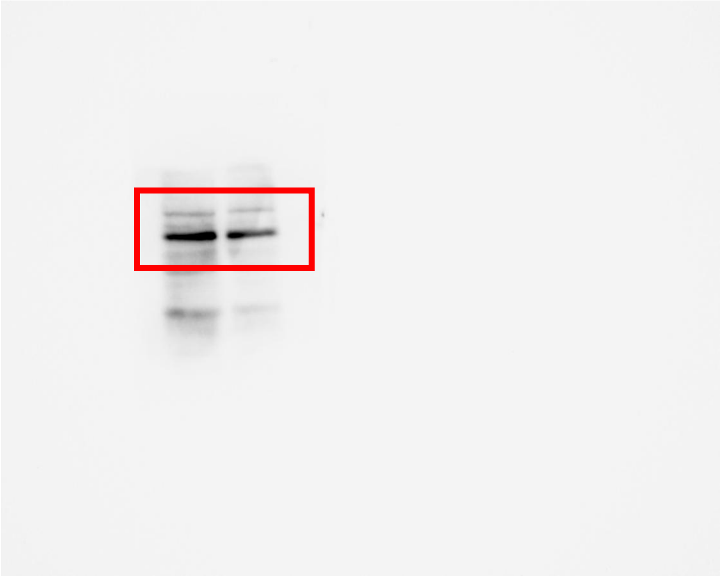

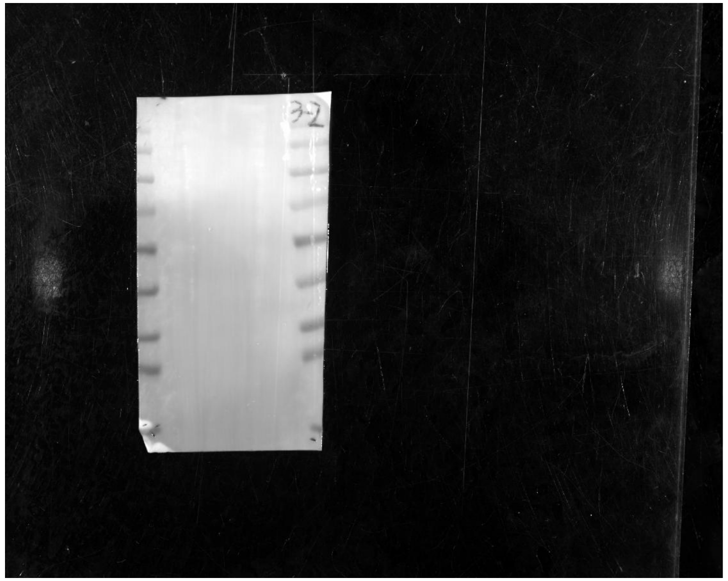

A549 β-actin

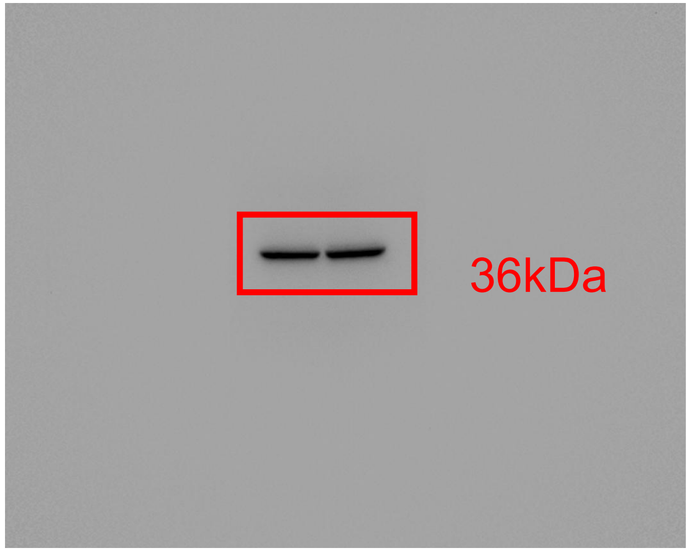

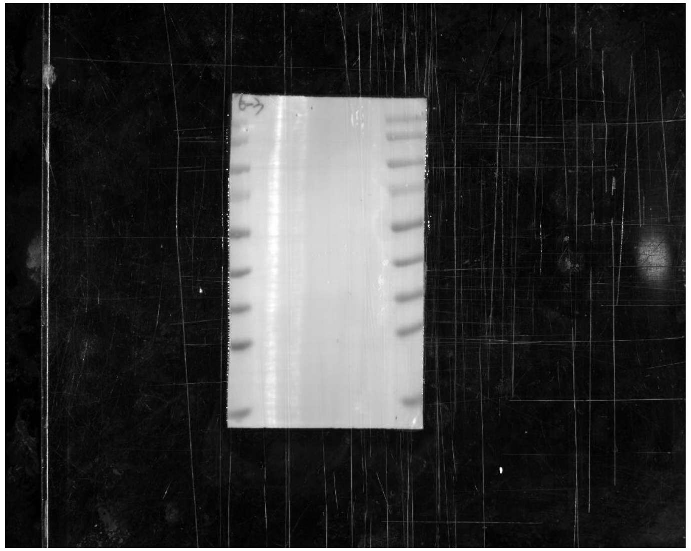

H1299 β-actin

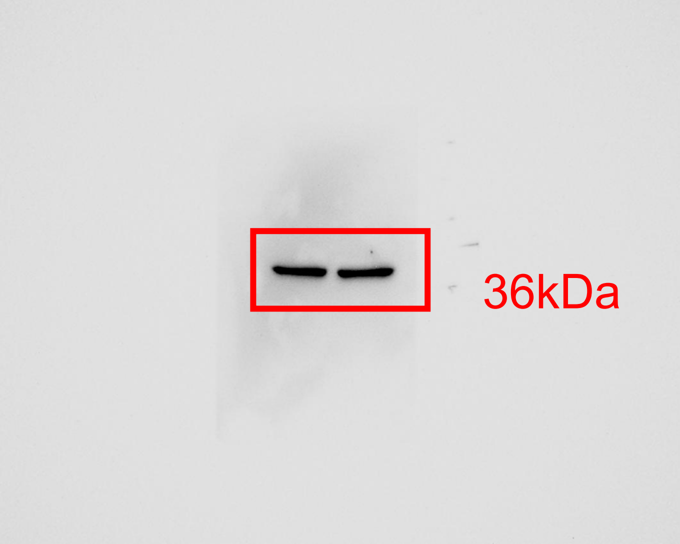

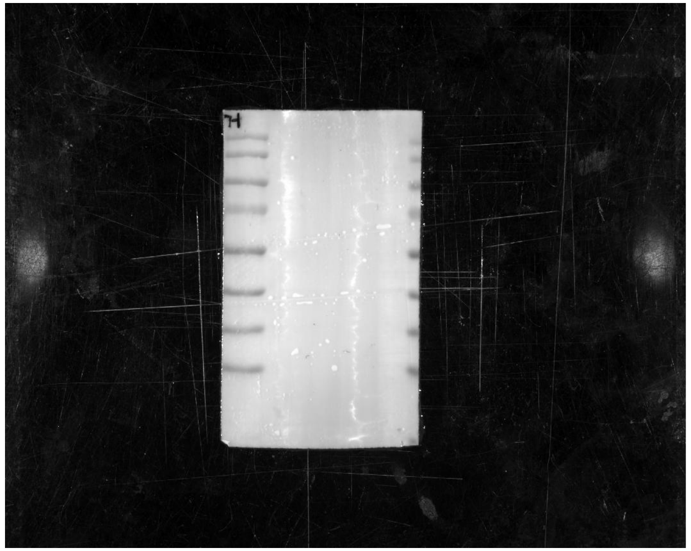

A549 K48 HA

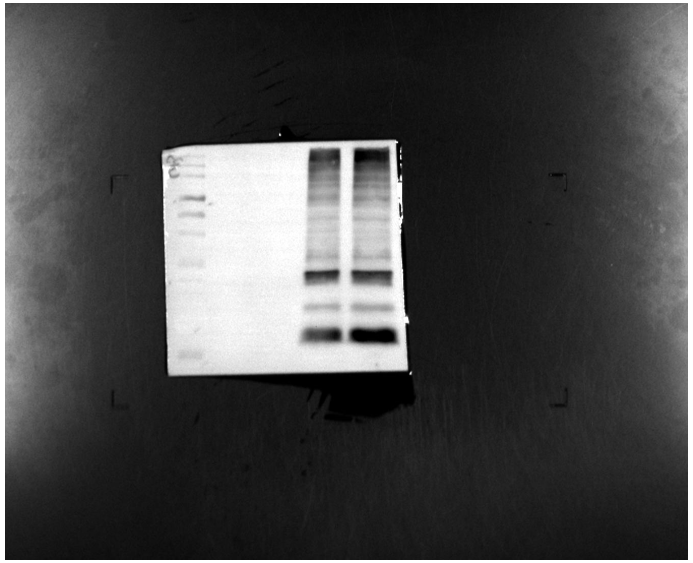

A549 K48 flag

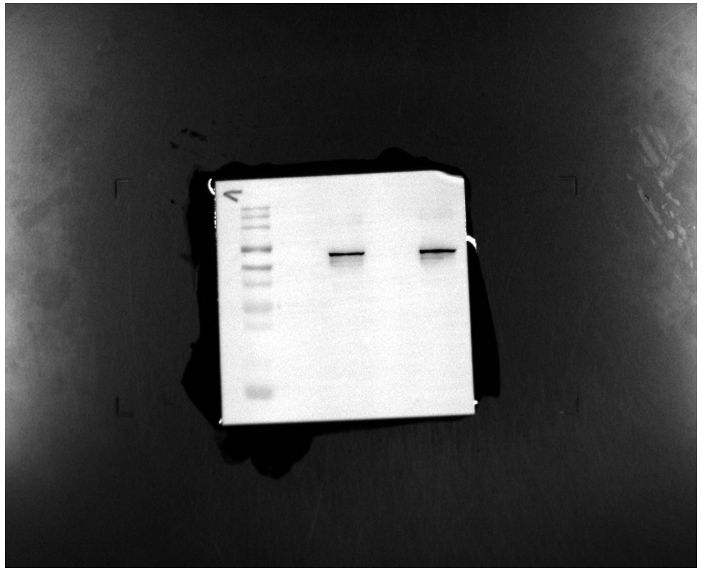

A549 K48 GFP

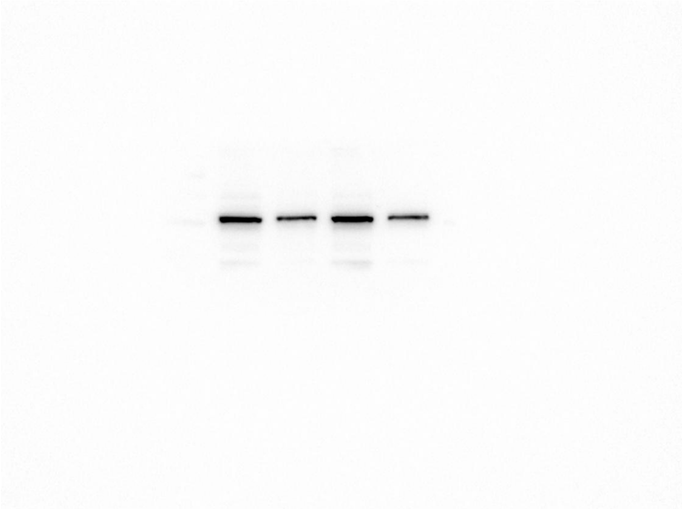

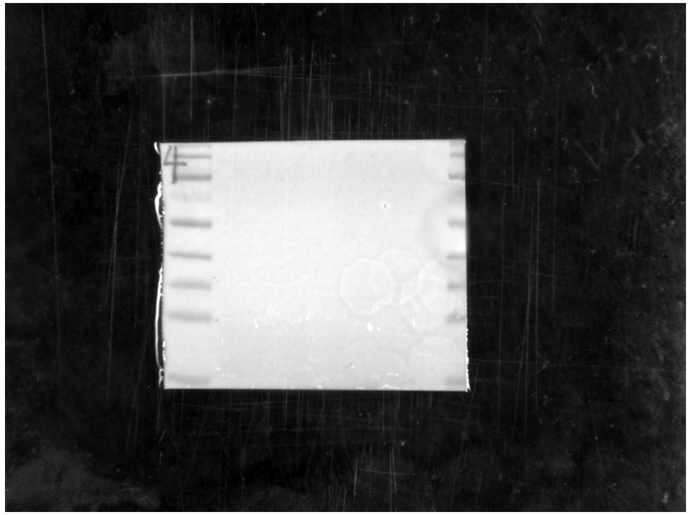

A549 K48
INPUT
GFP

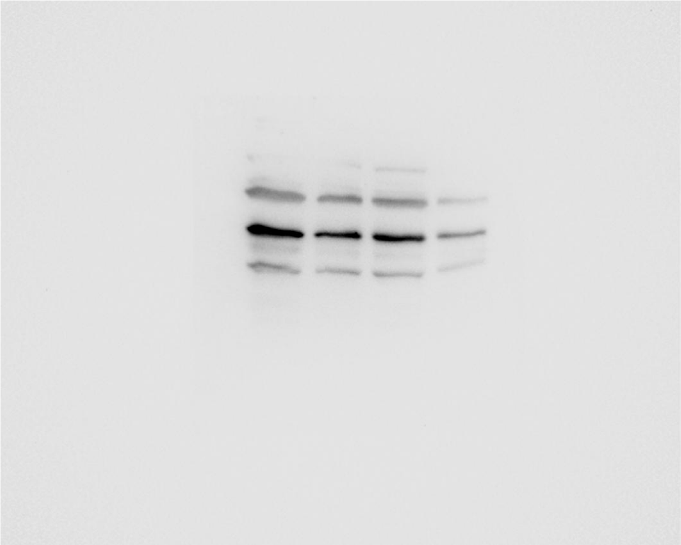

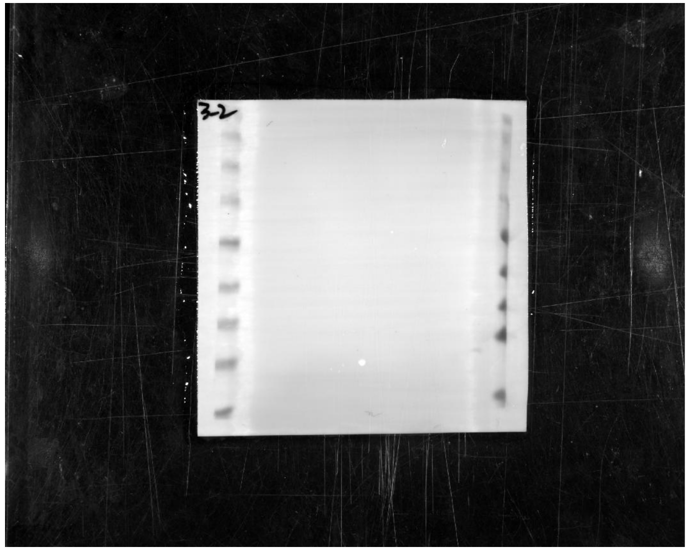

A549 K48
INPUT
β-actin

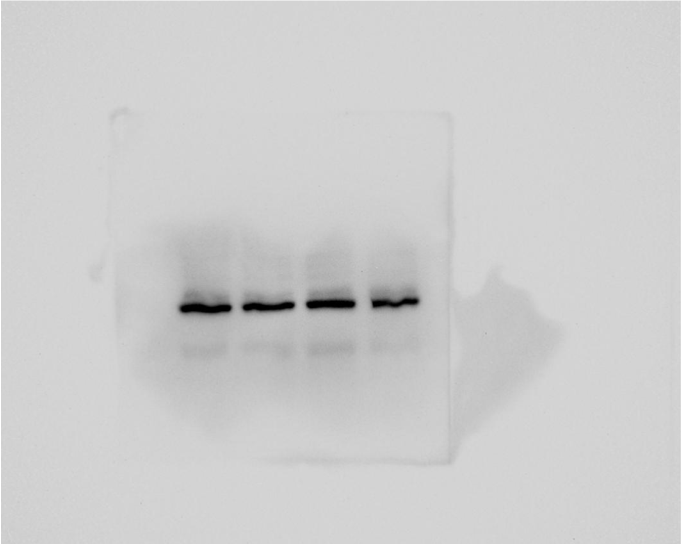

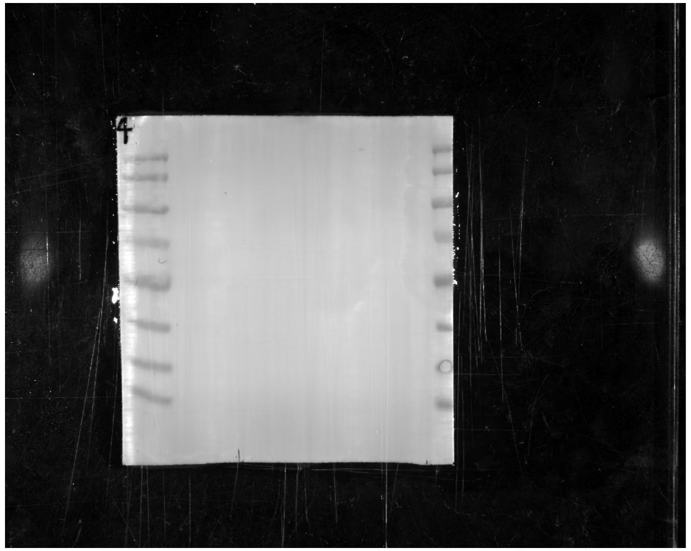

H1299 K48 HA

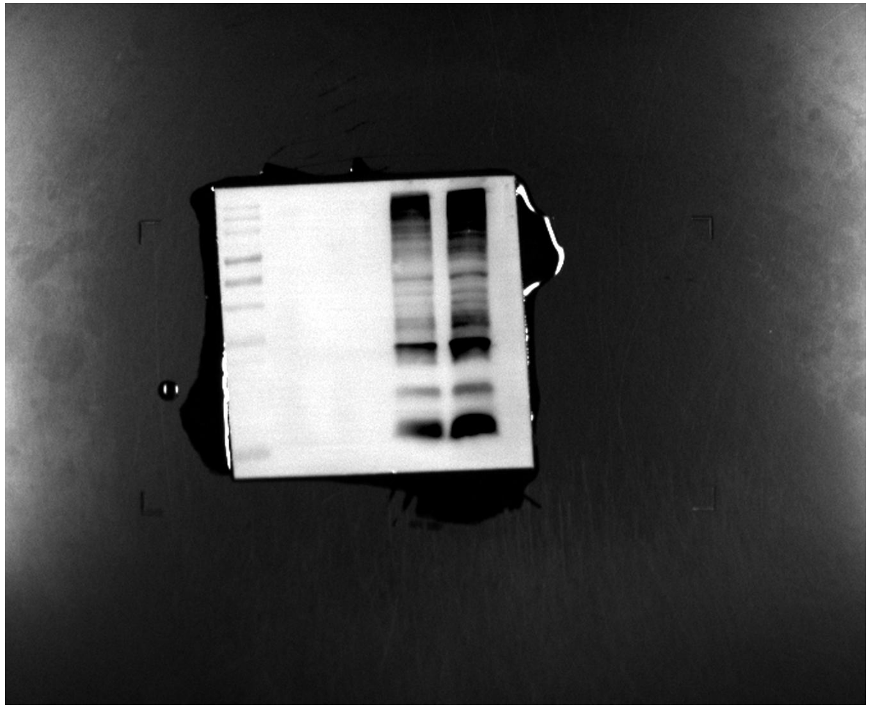

H1299 K48 flag

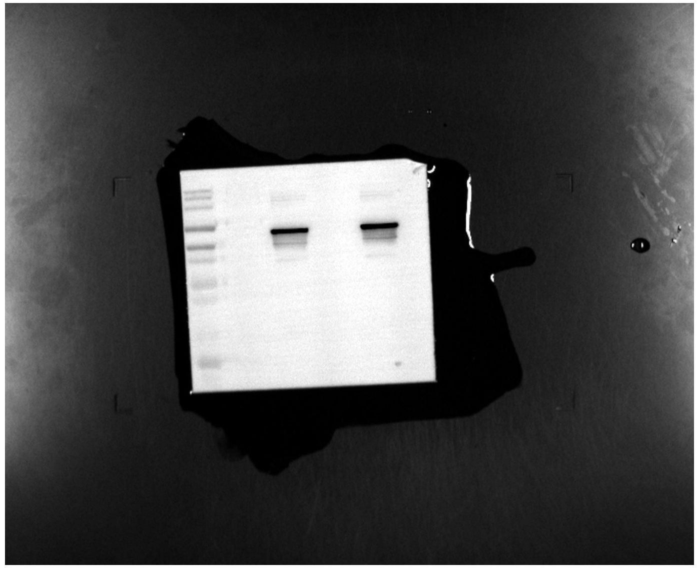

H1299 K48 GFP

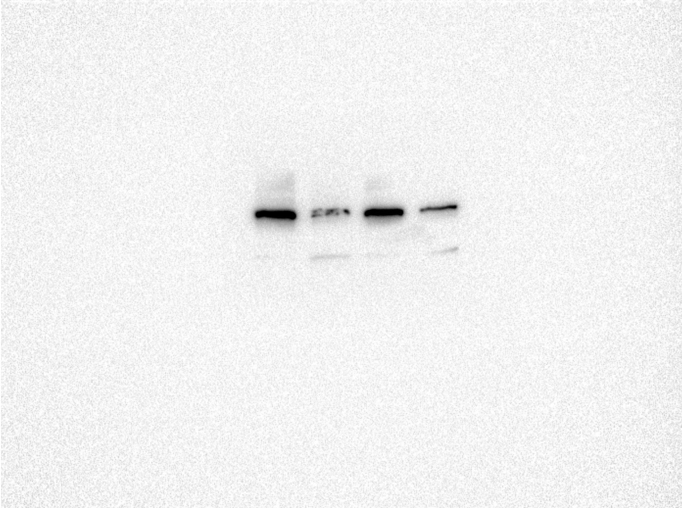

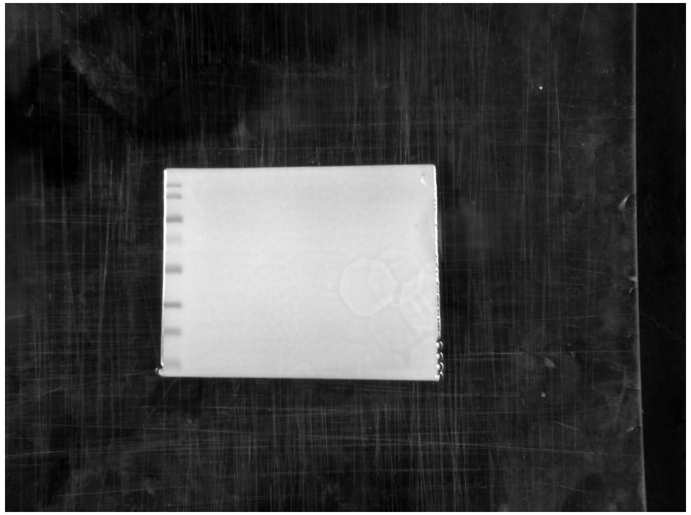

H1299 K48
INPUT
GFP

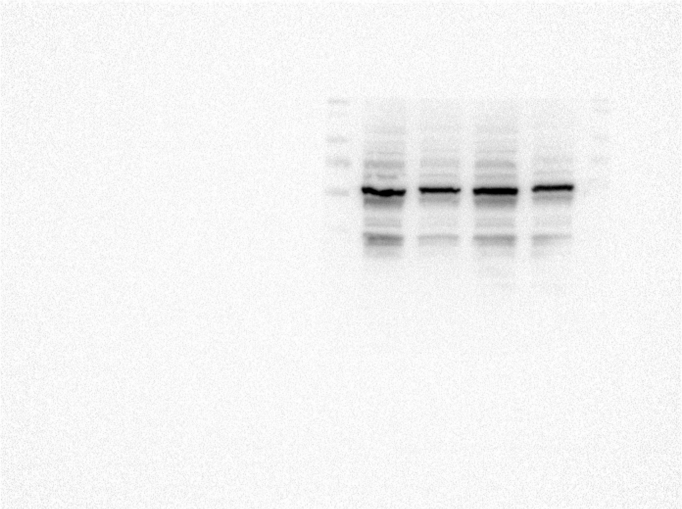

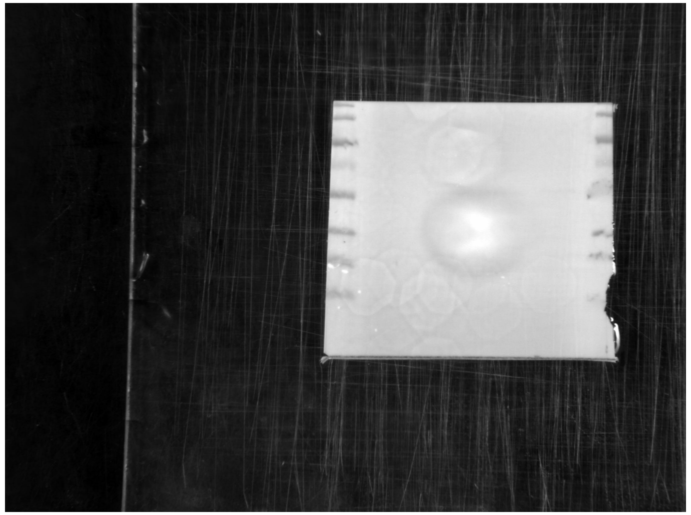

H1299 K48
INPUT
β-actin

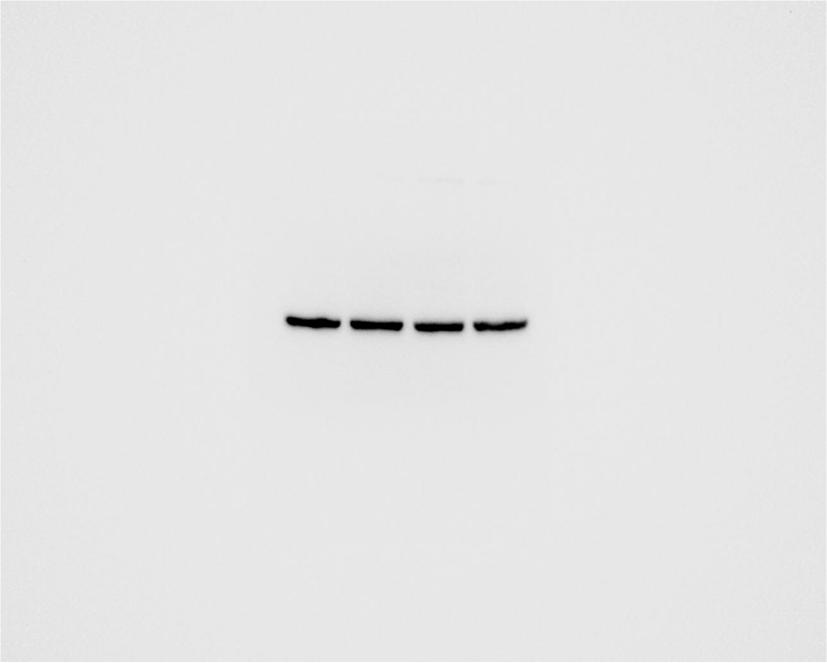

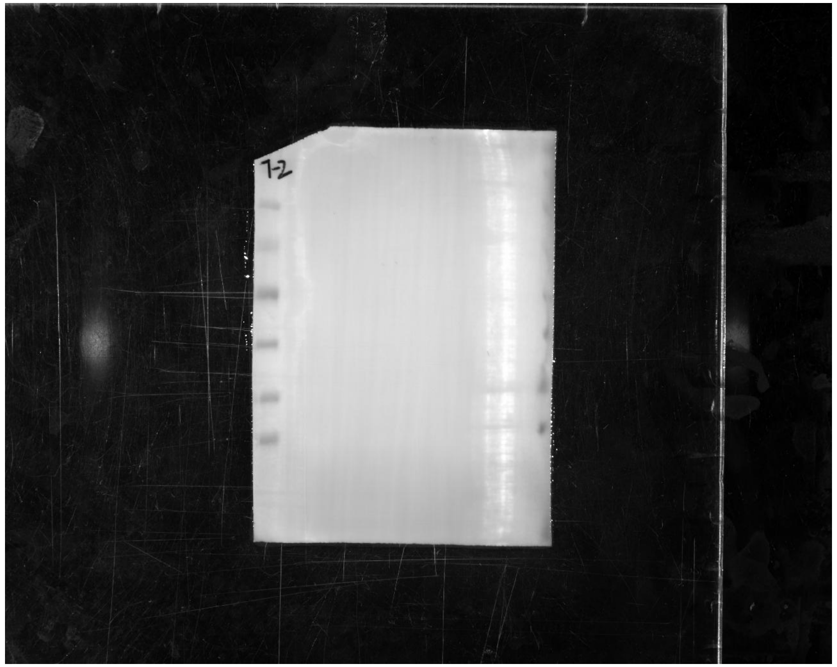

A549 K63 HA

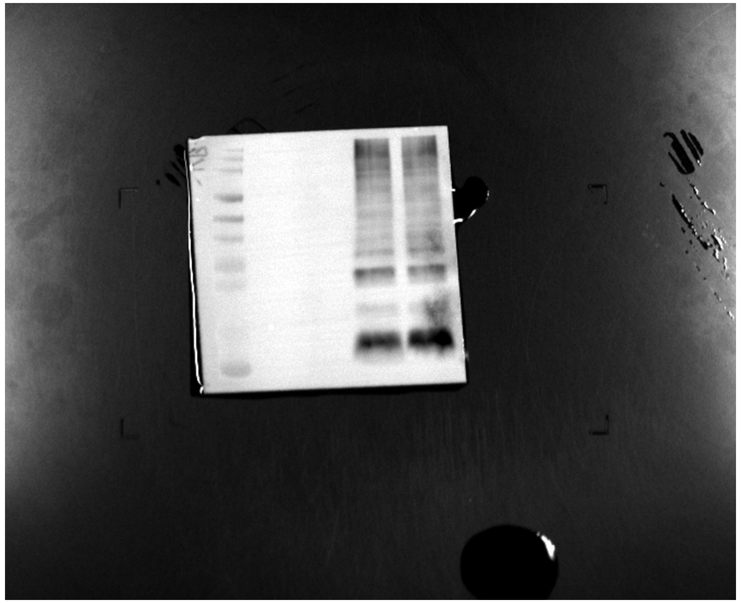

A549 K63 flag

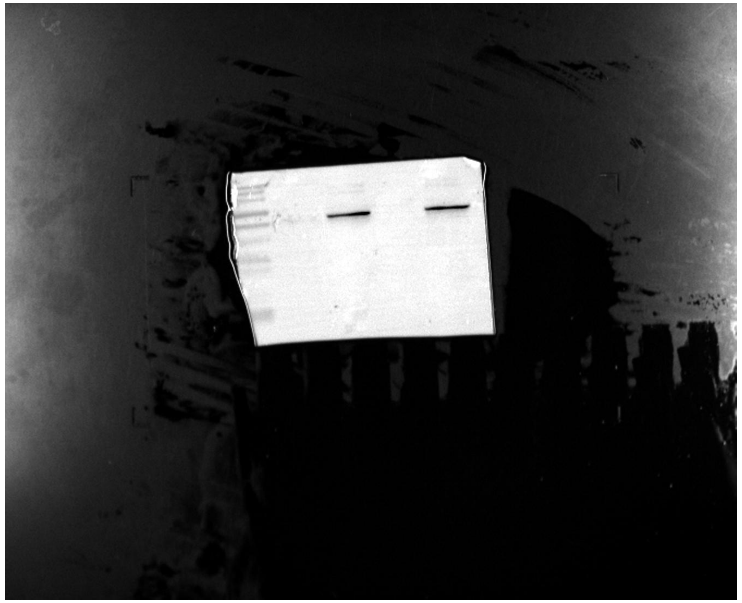

A549 K63 GFP

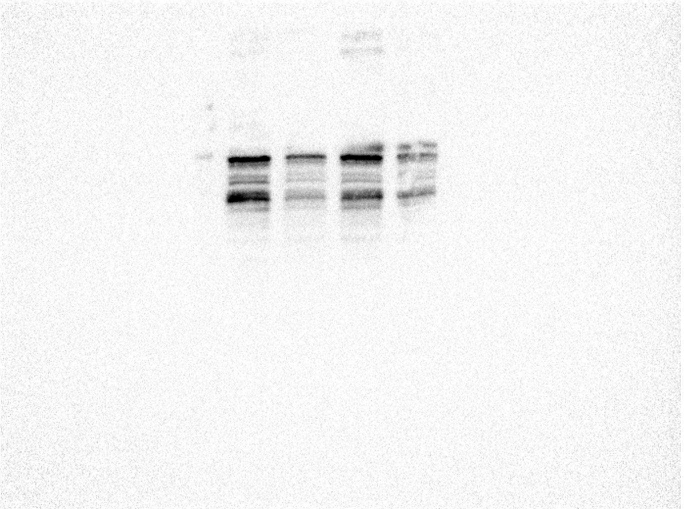

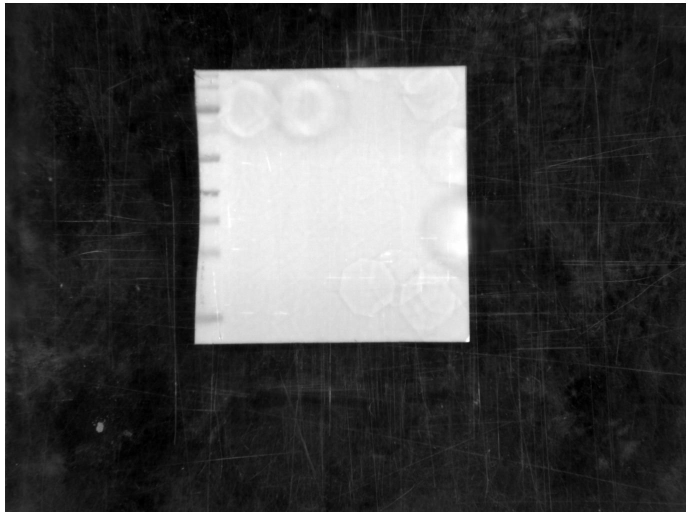

A549 K63
INPUT
GFP

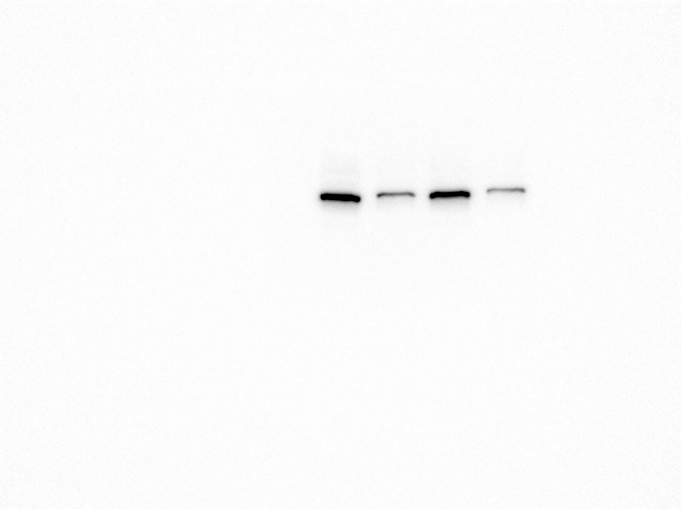

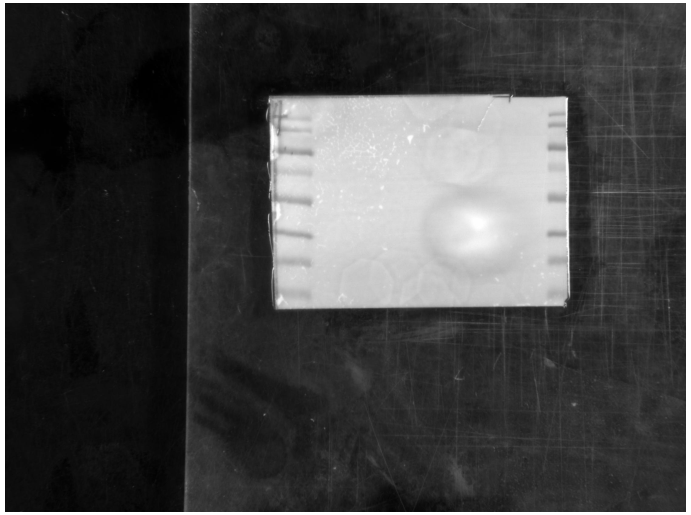

A549 K63
INPUT
β-actin

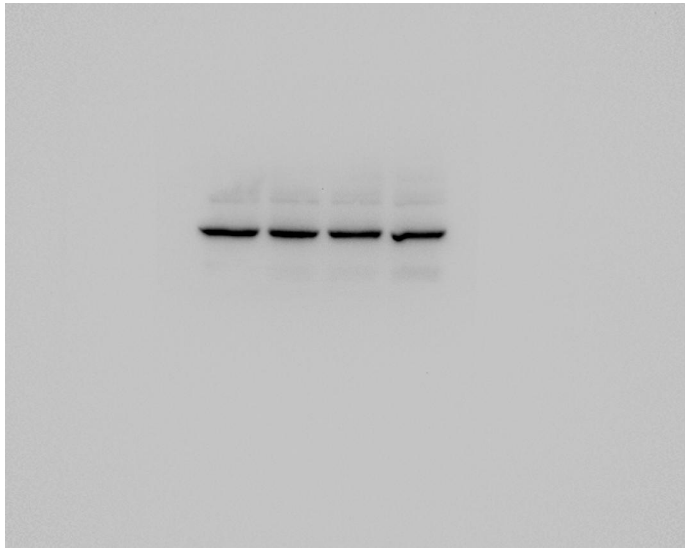

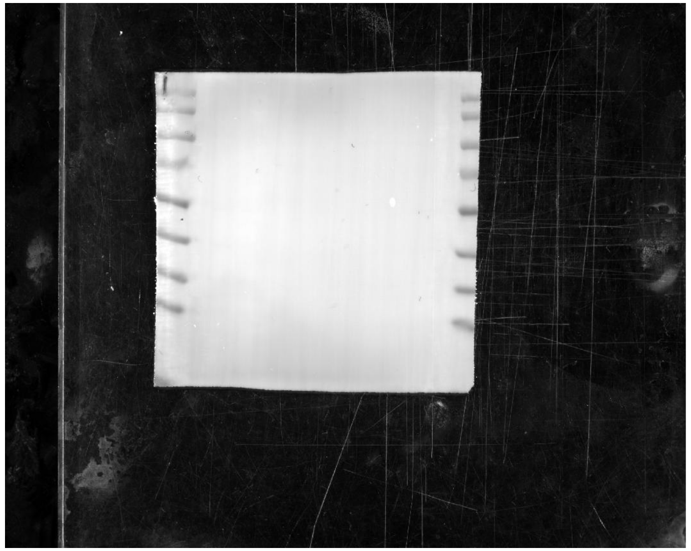

H1299 K63 HA

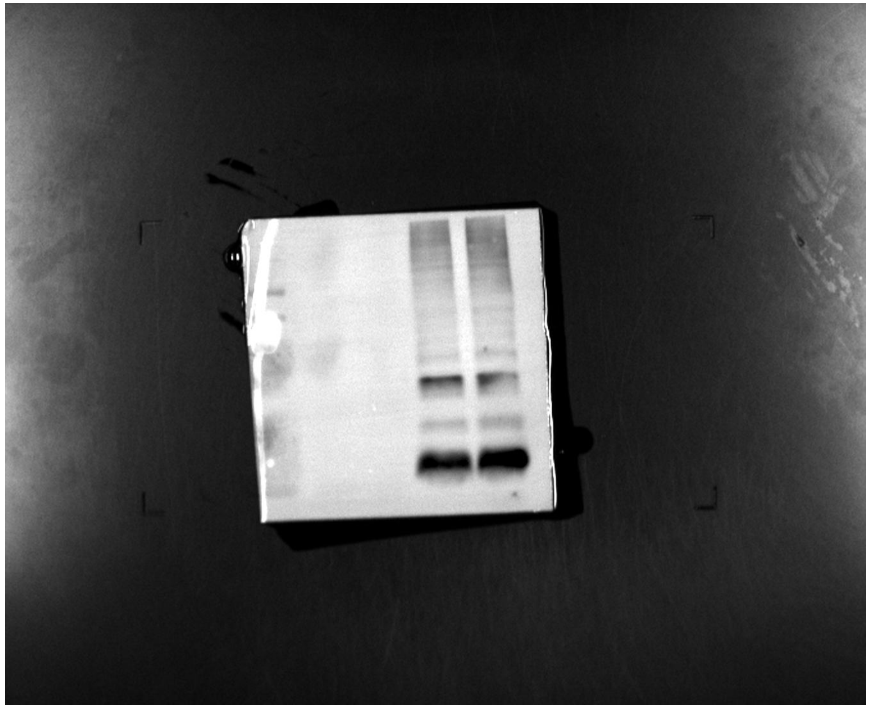

H1299 K63 flag

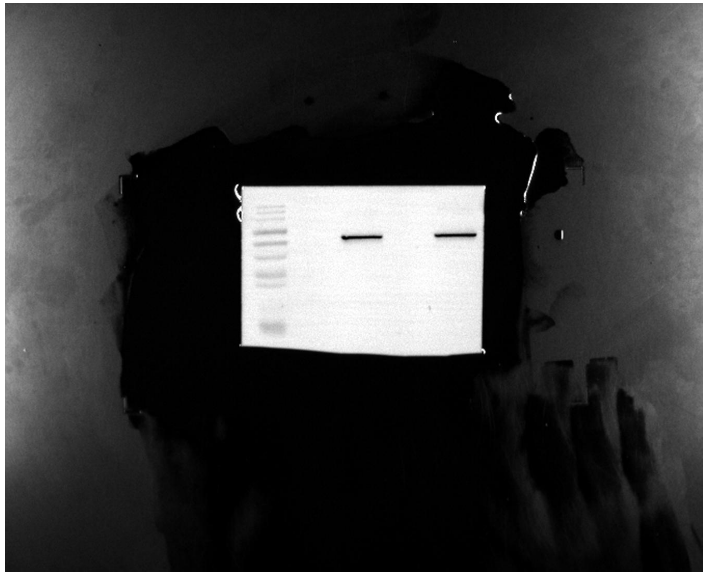

H1299 K63 GFP

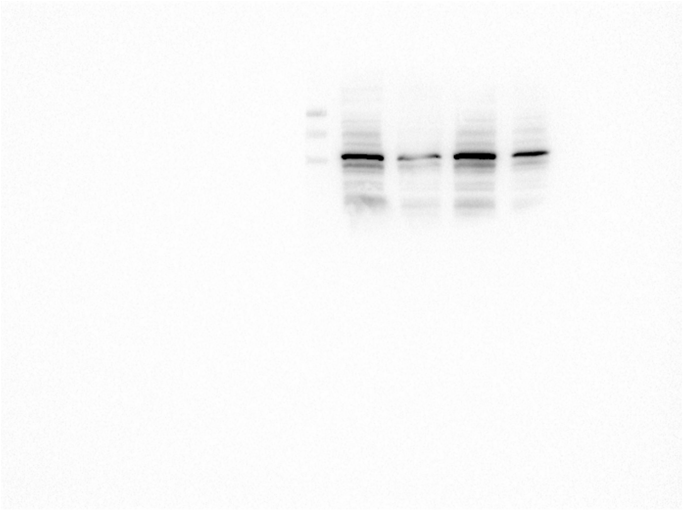

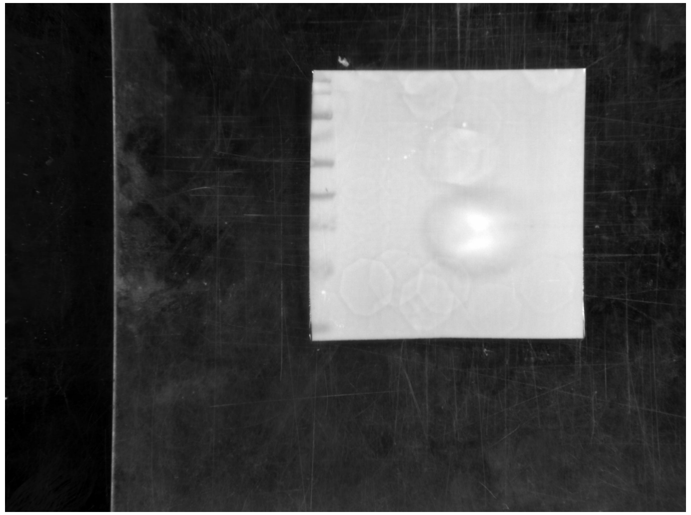

H1299 K63
INPUT
GFP

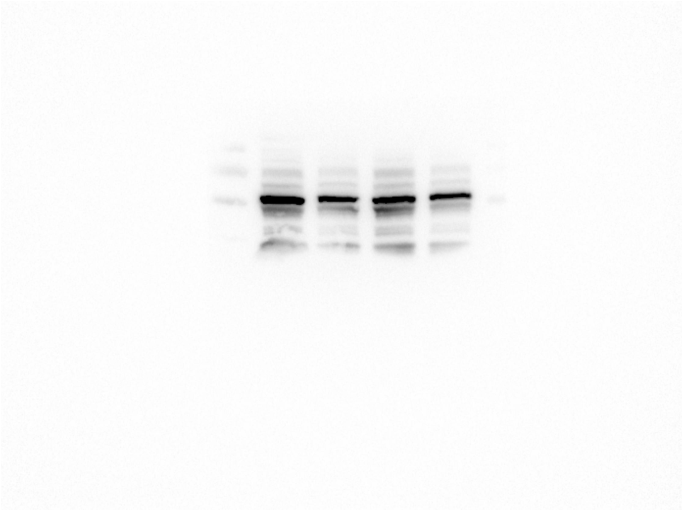

H1299 K63
INPUT
β-actin

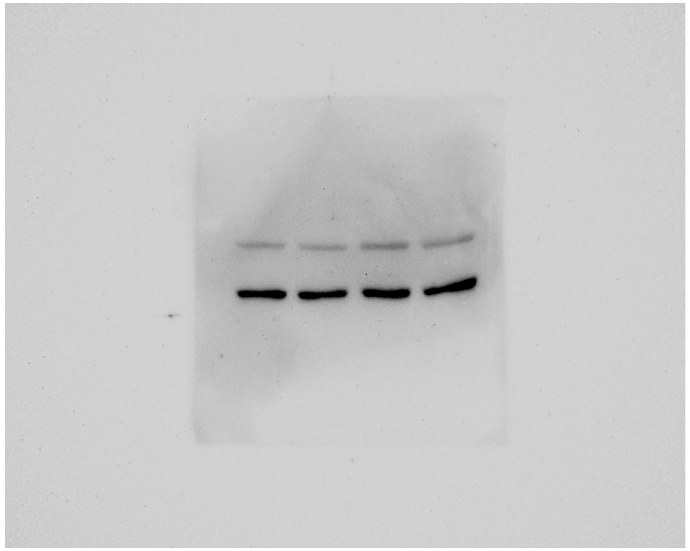

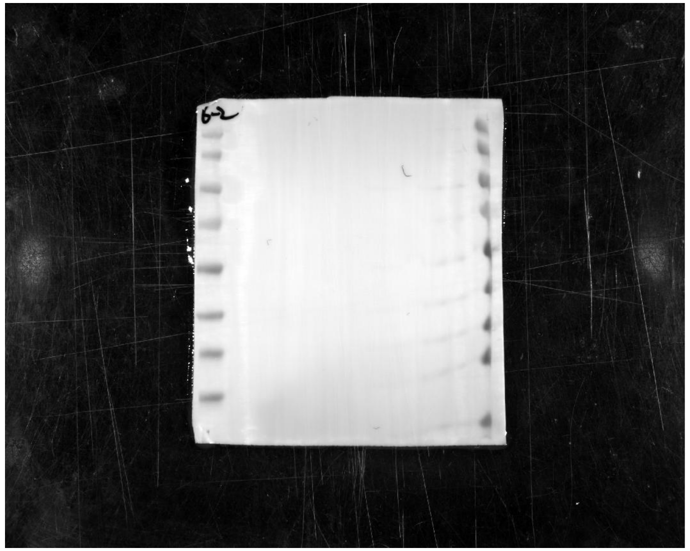

A549
IB:TRIM56

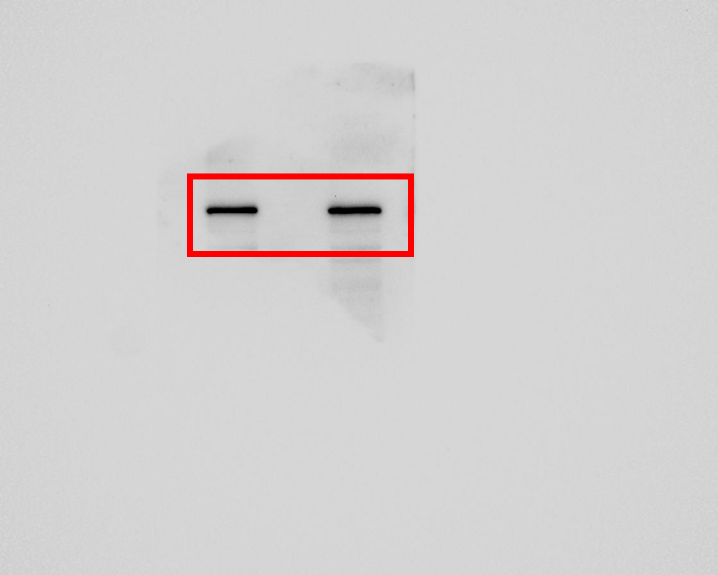

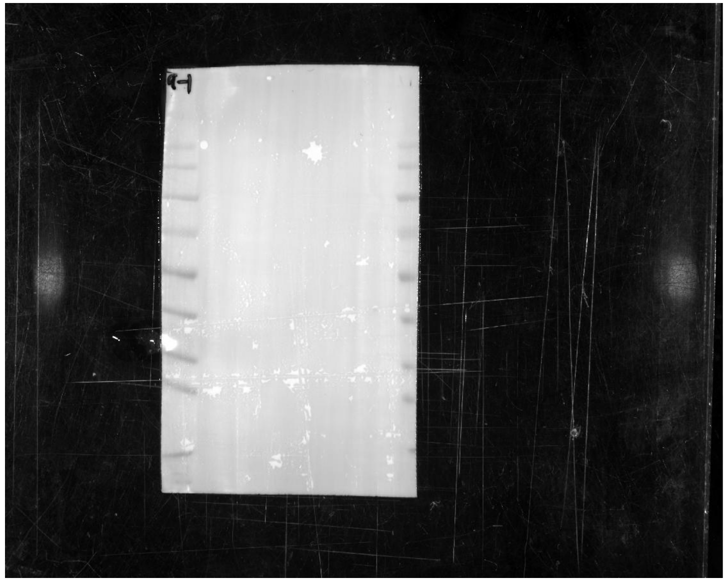

A549 IP:Flag

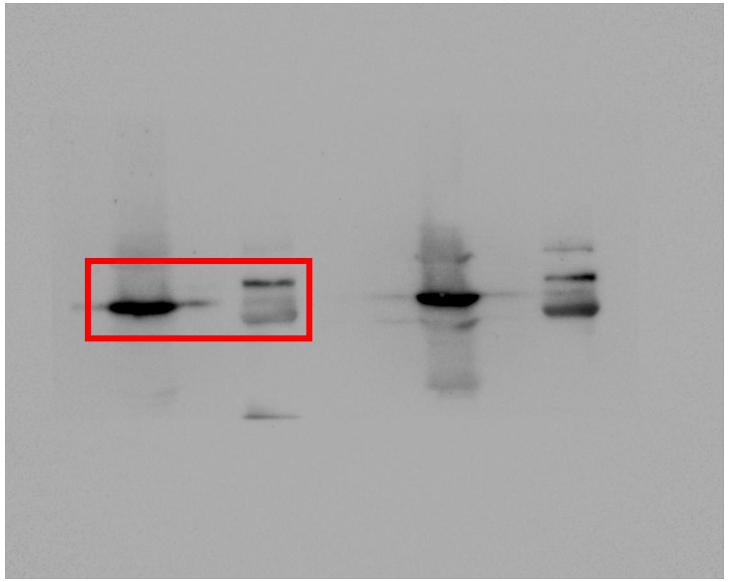

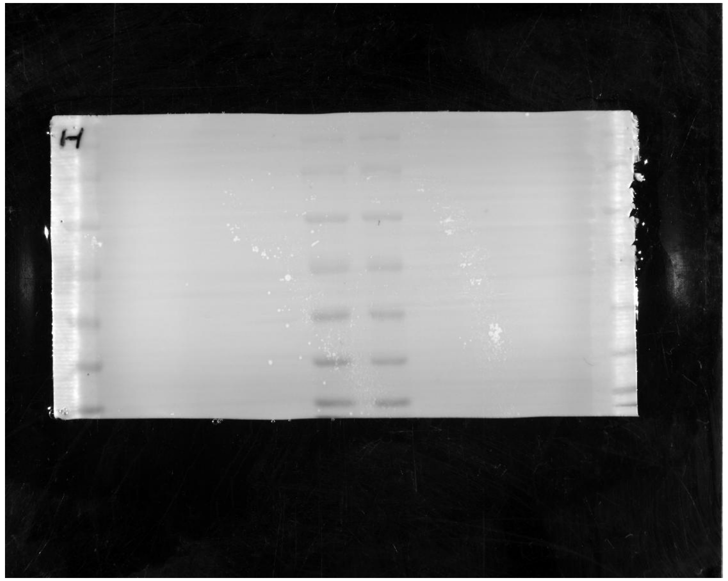

H1299
IB:TRIM56

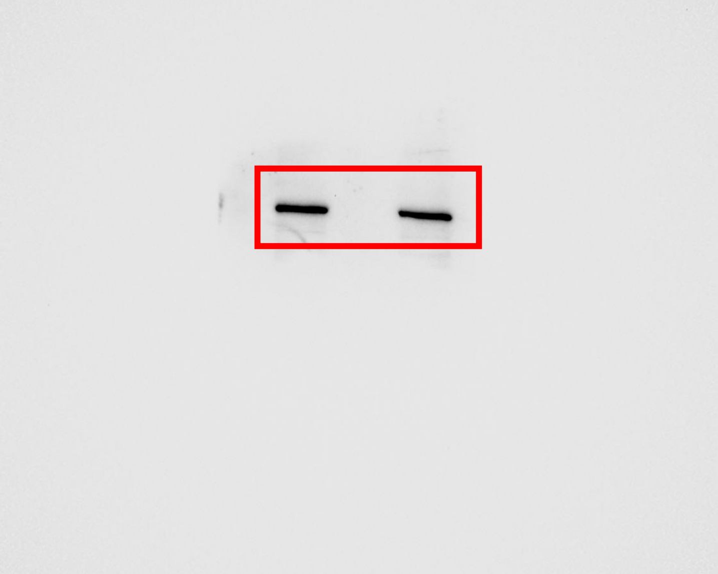

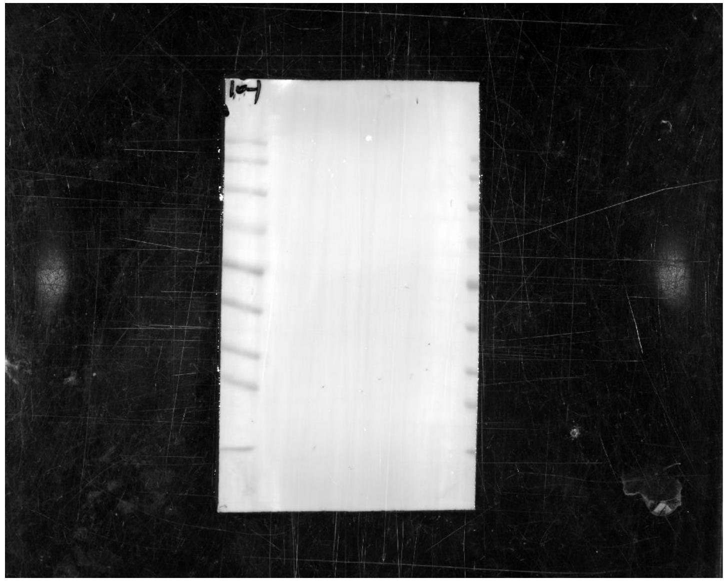

H1299
IP:Flag

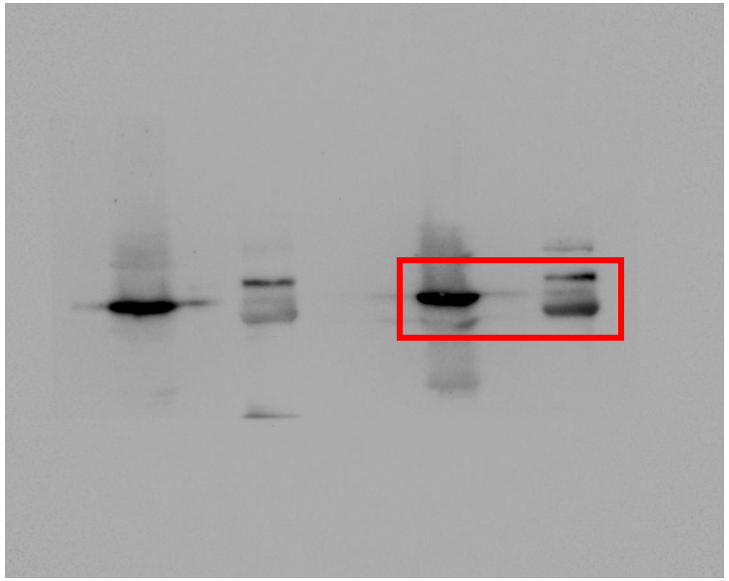

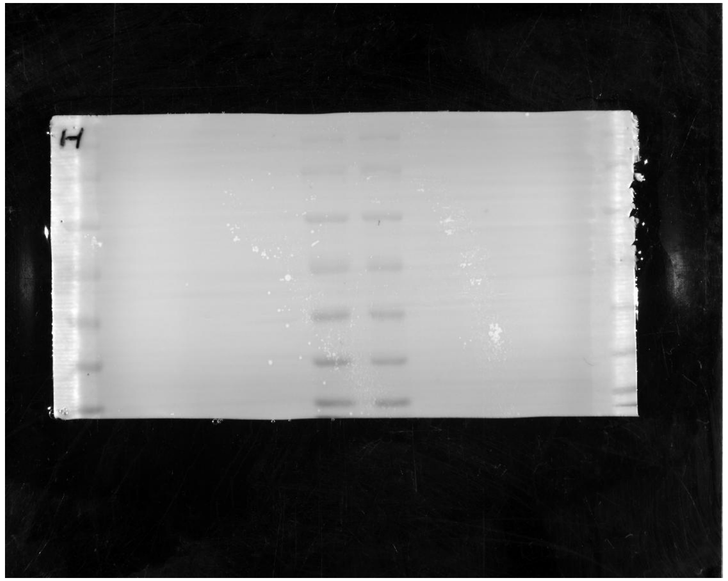

A549
IP:GFP-PTEN

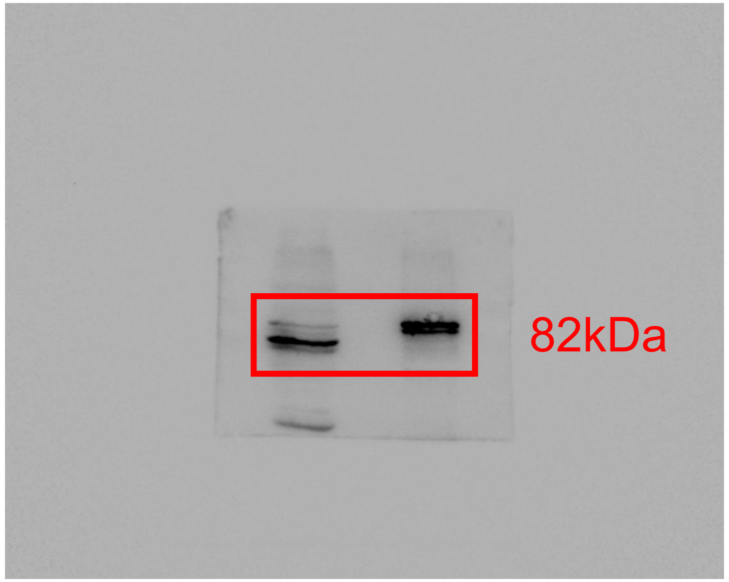

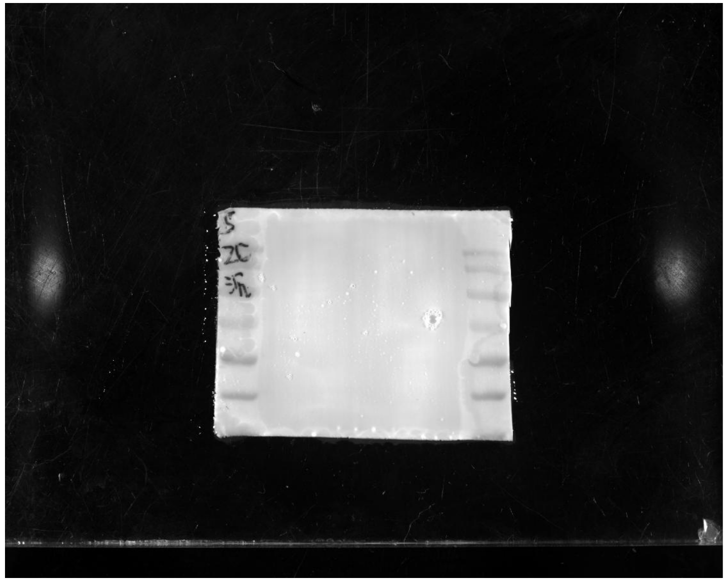

A549
IB:TRIM56

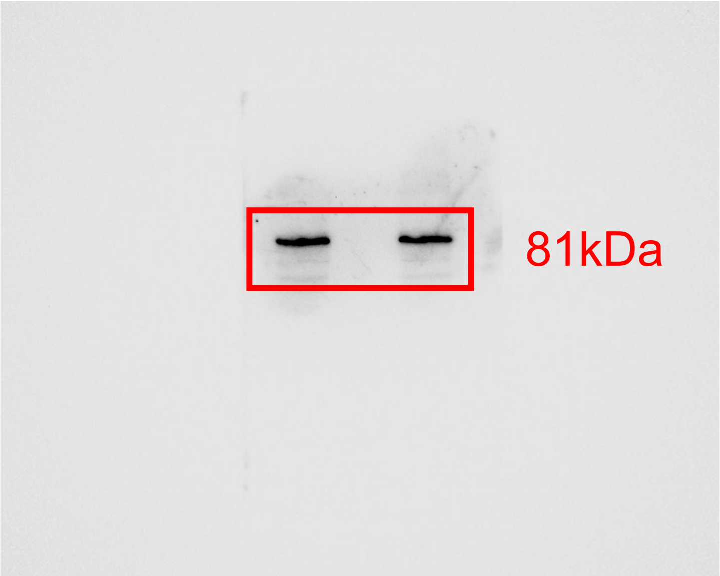

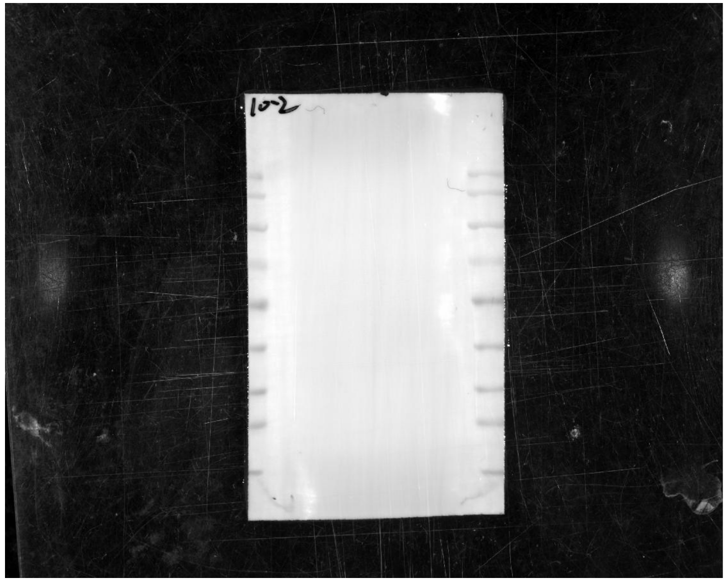

H1299
IP:GFP-PTEN

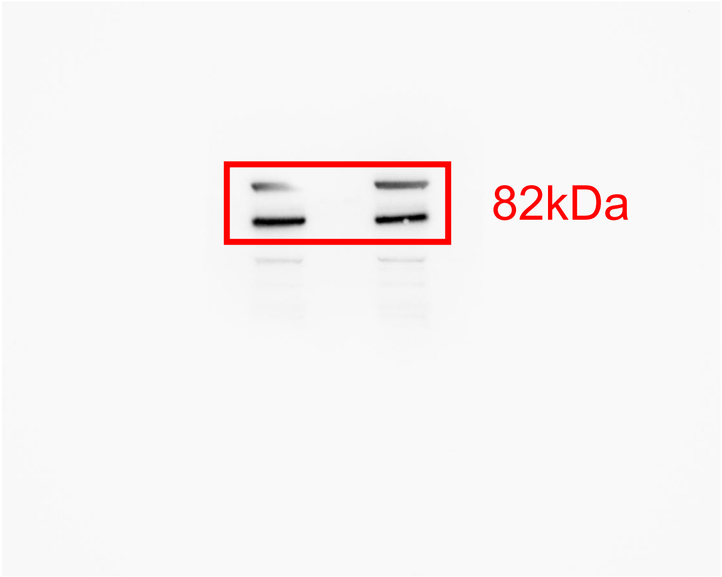

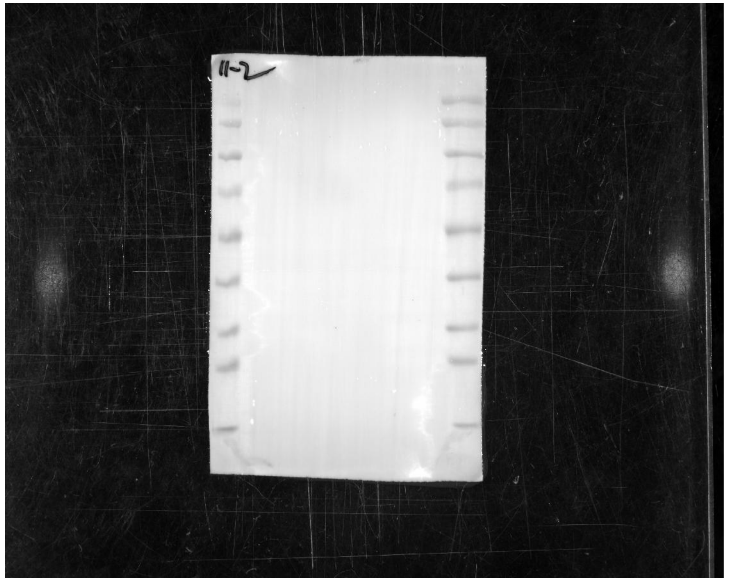

H1299
IB:TRIM56

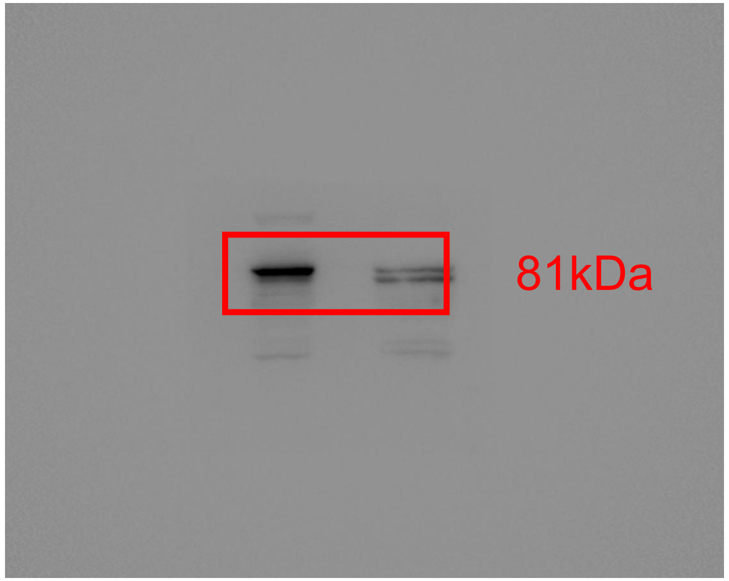

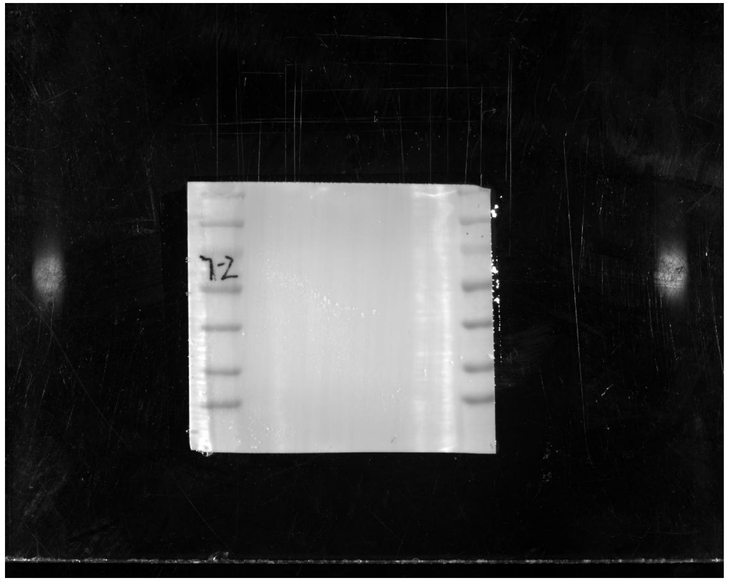

A549 Ub

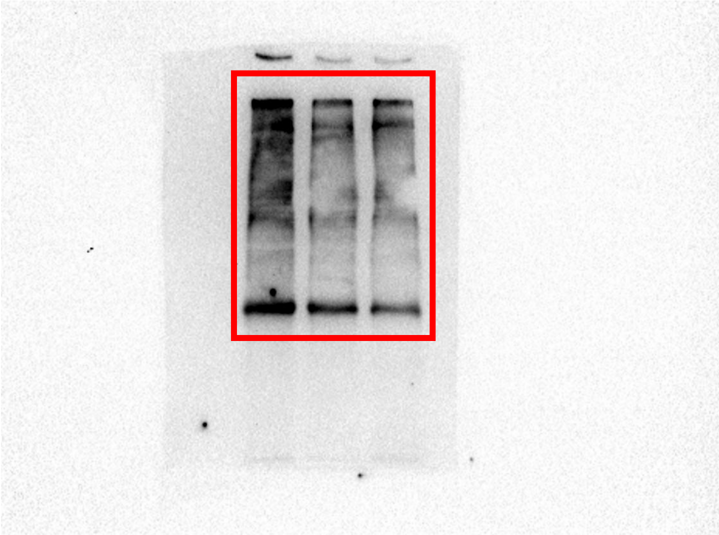

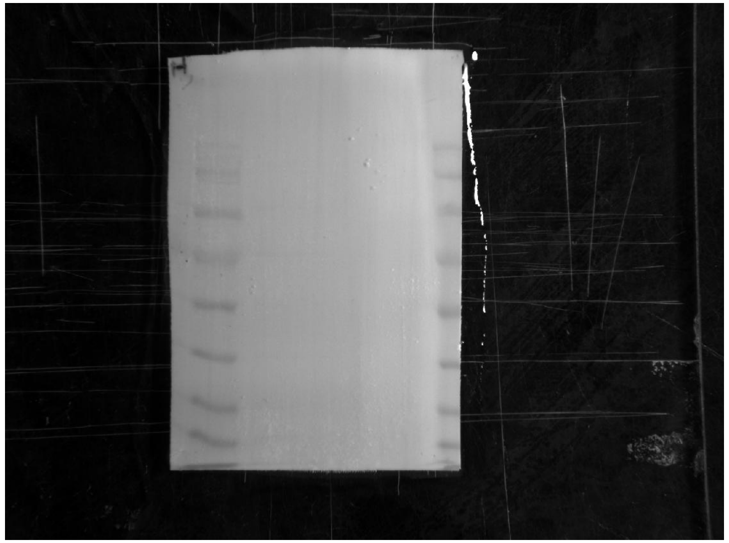

A549 PTEN

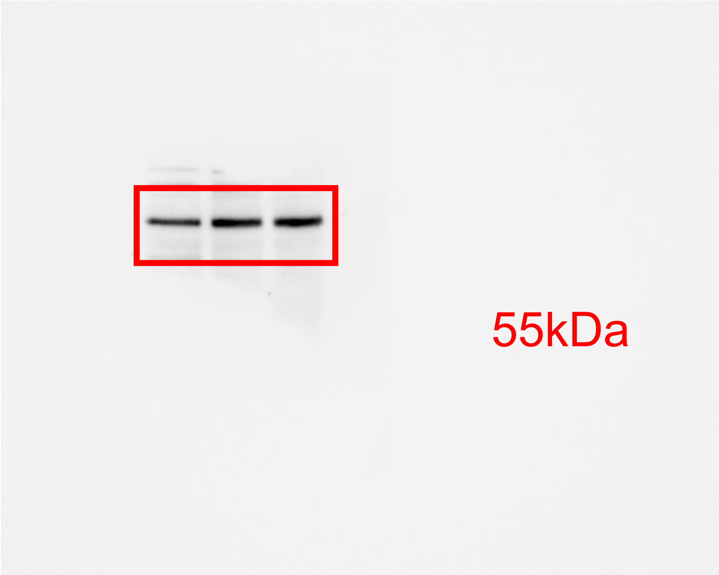

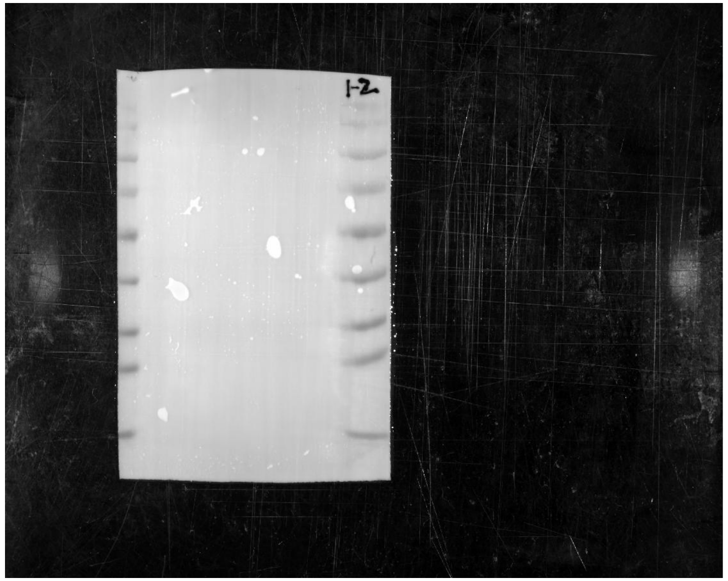

A549 TRIM56

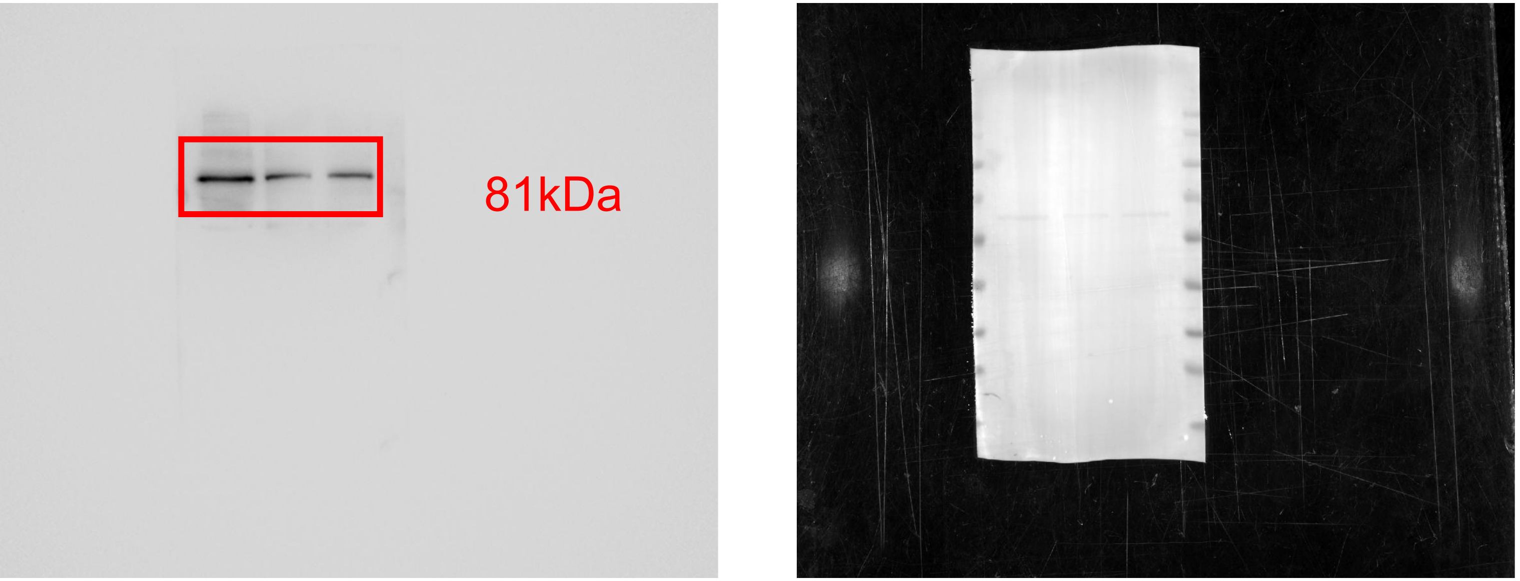

A549 β-actin

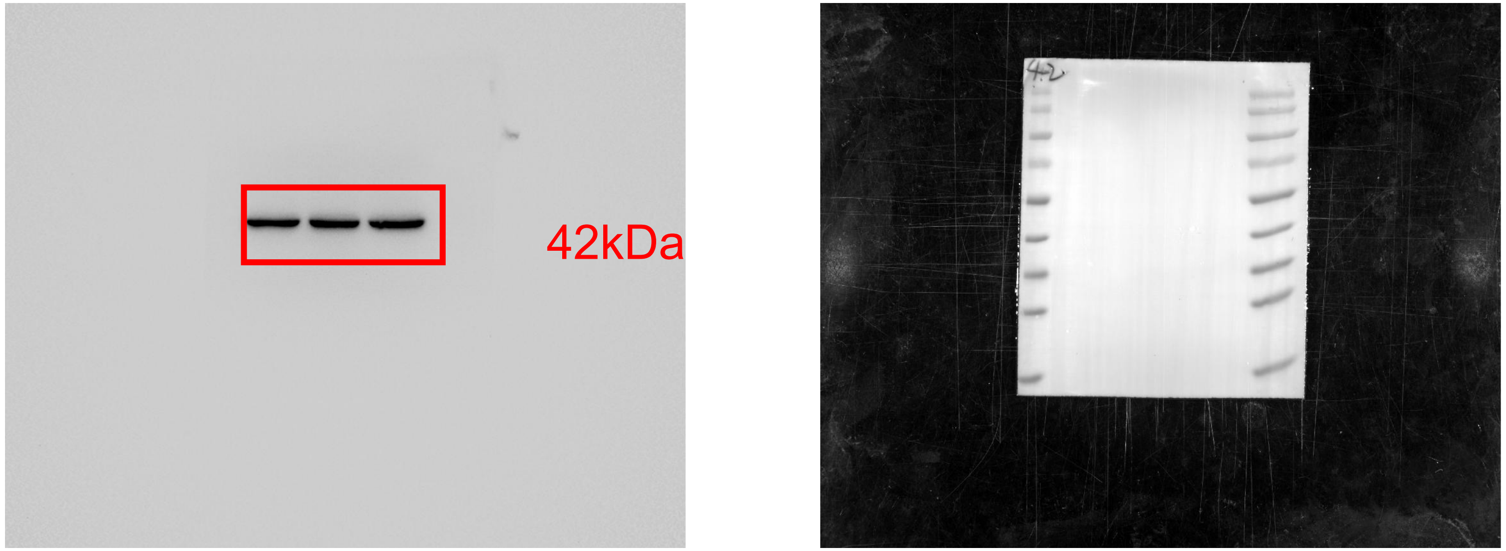

H1299 Ub

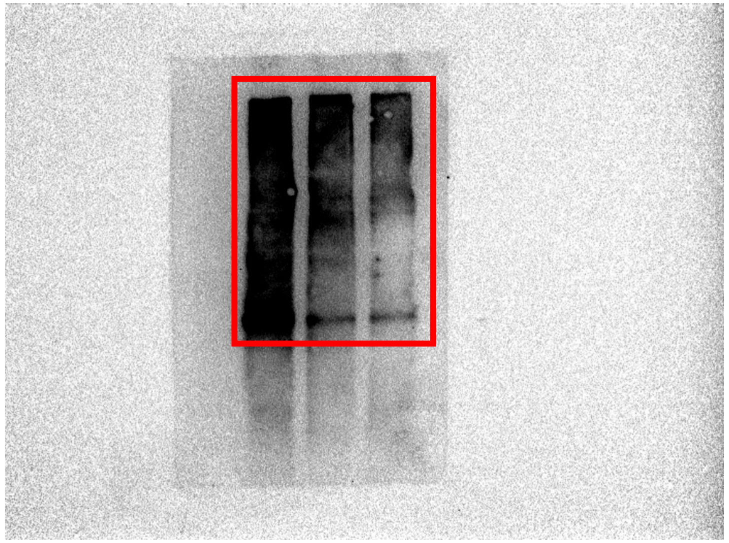

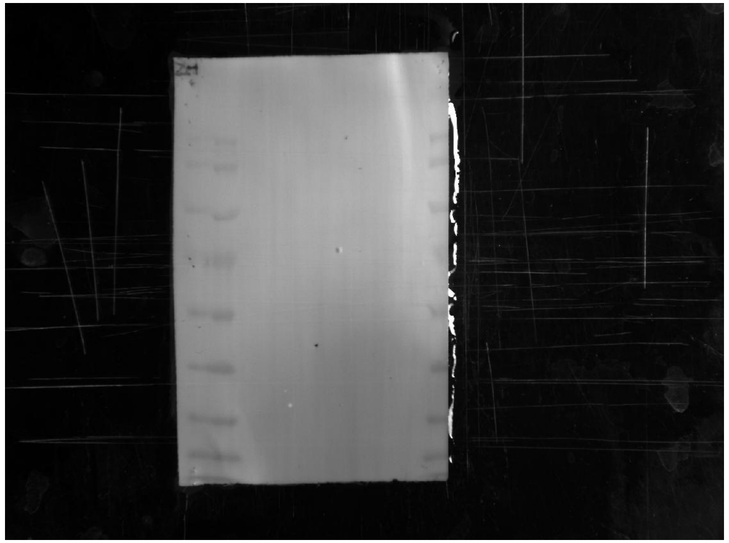

H1299 PTEN

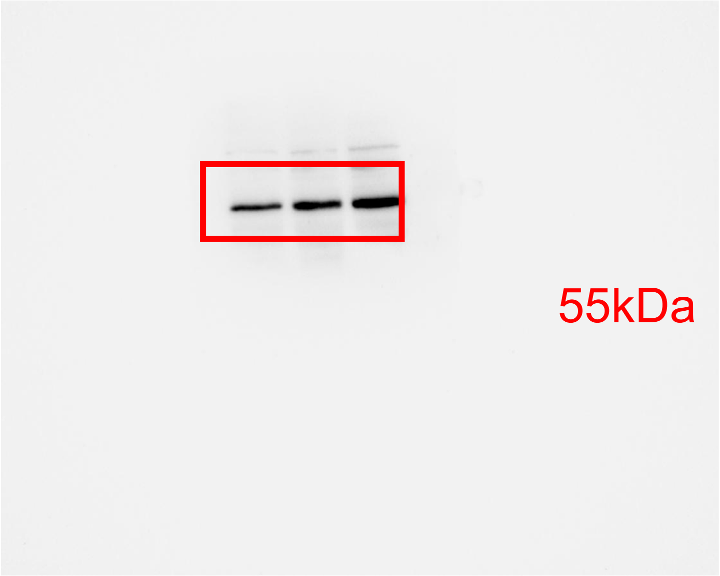

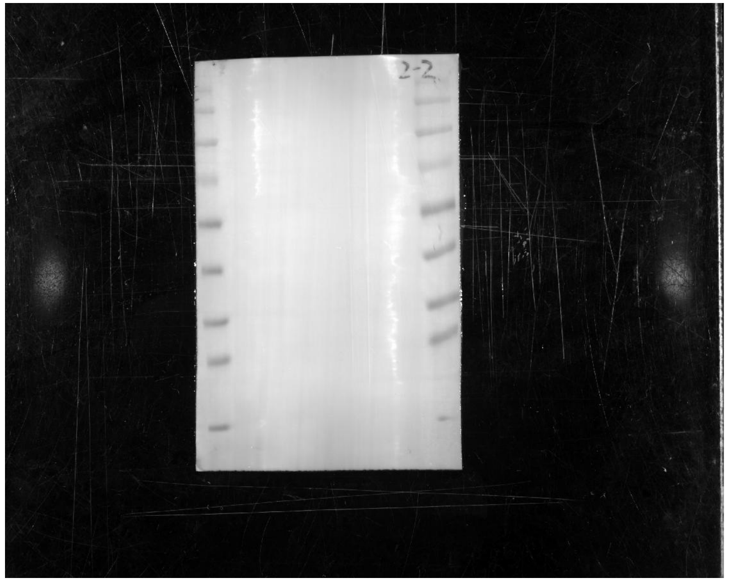

H1299 TRIM56

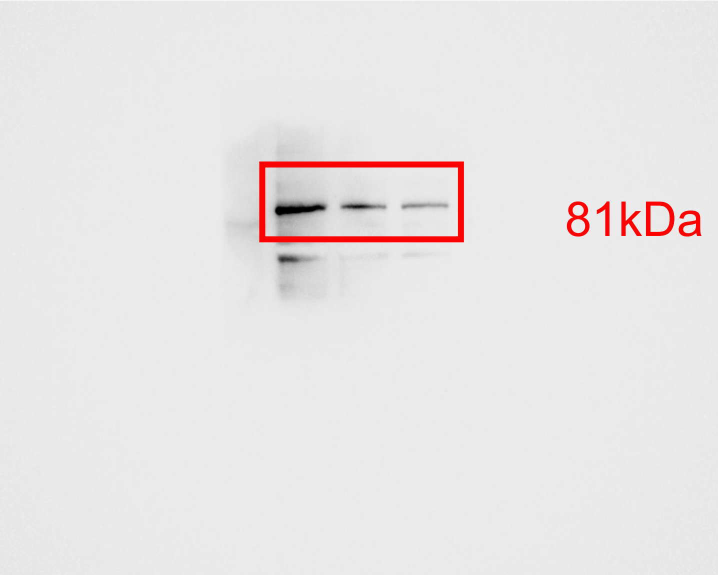

81kDa

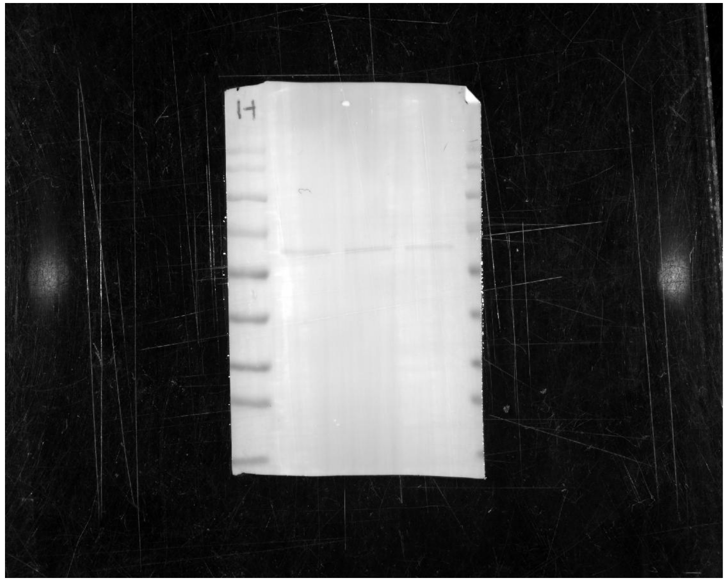

H1299 β-actin

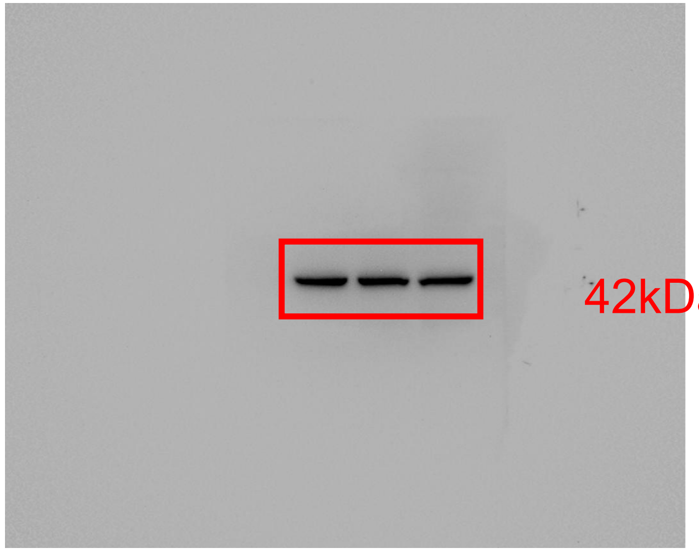

42kDa

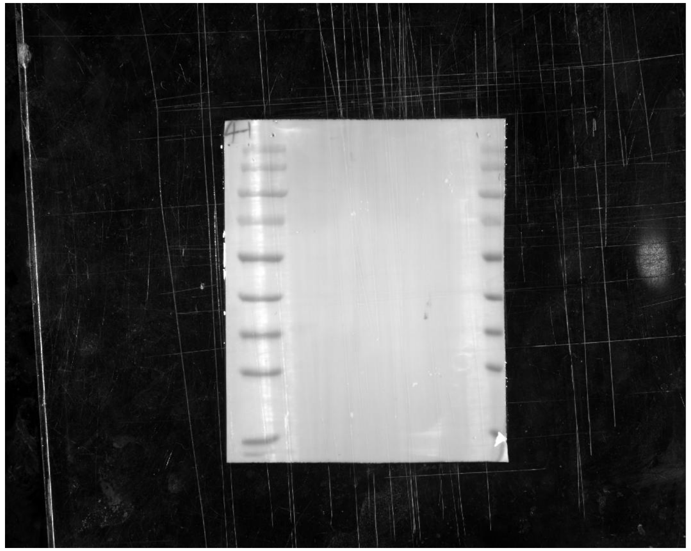

A549 Ub

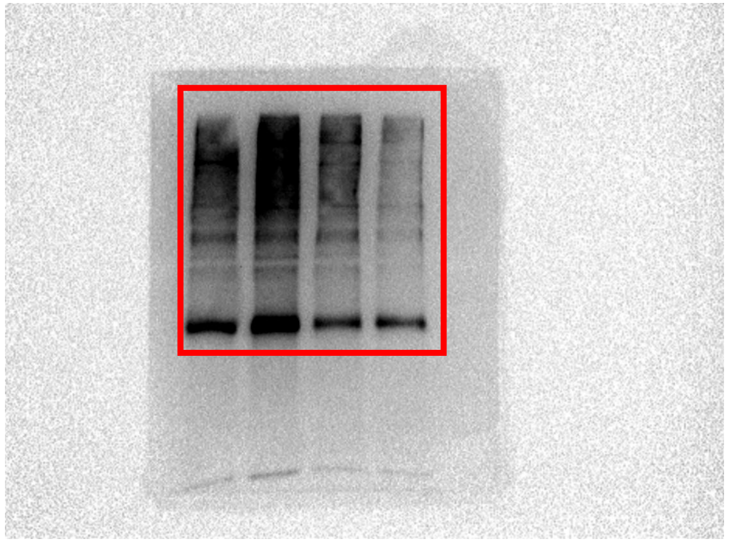

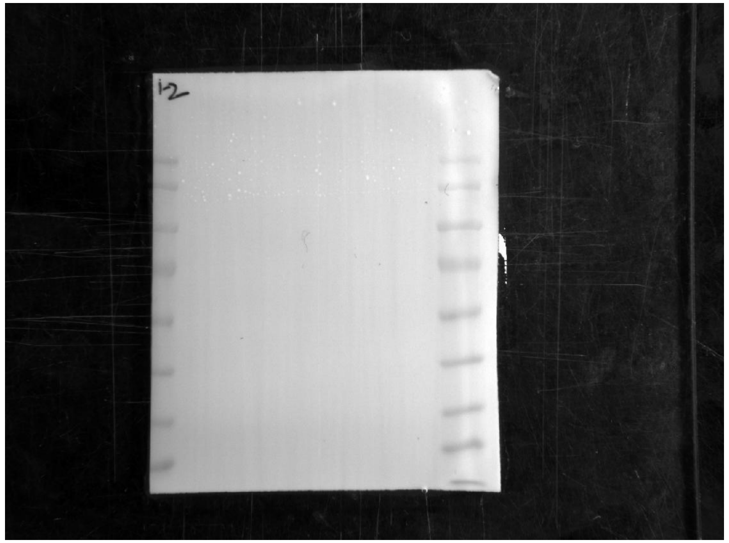

A549 ZC-FLAG

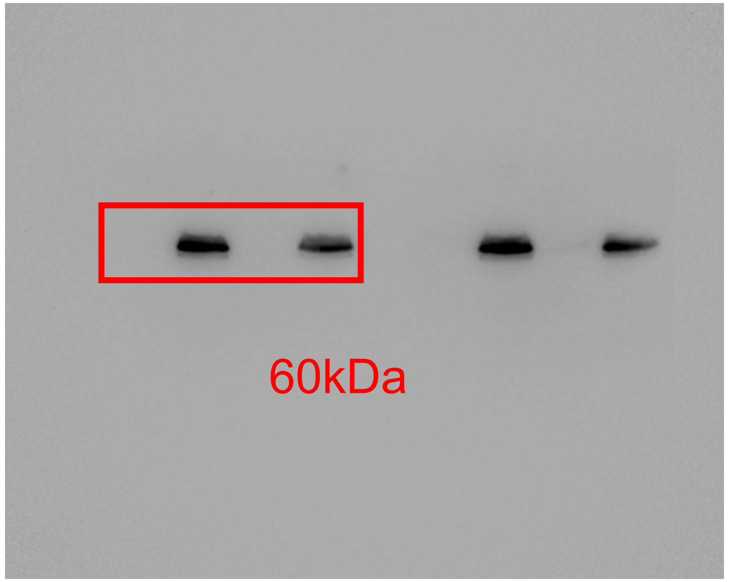

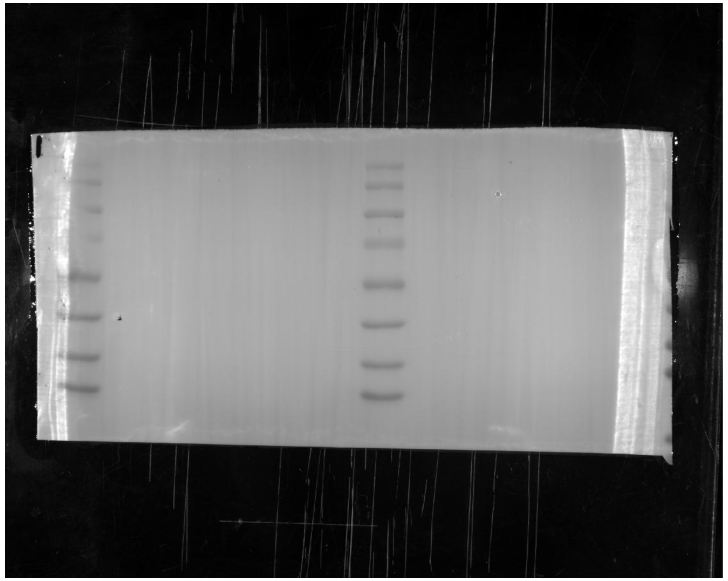

A549 PTEN

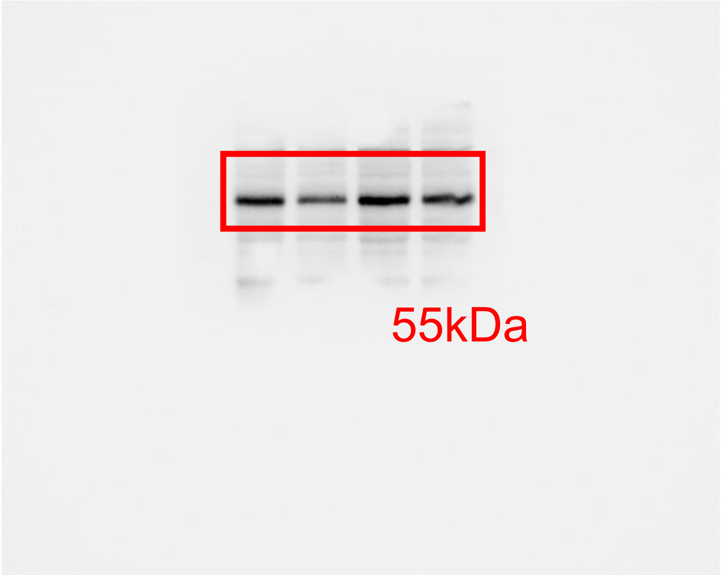

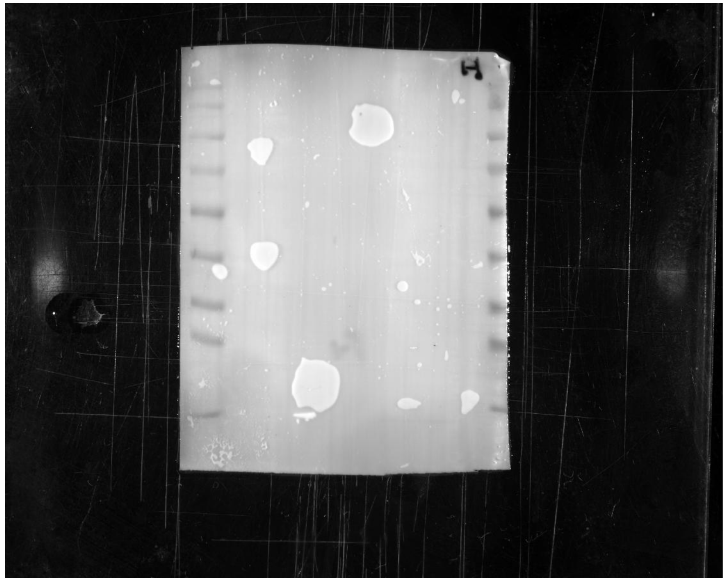

A549 TRIM56

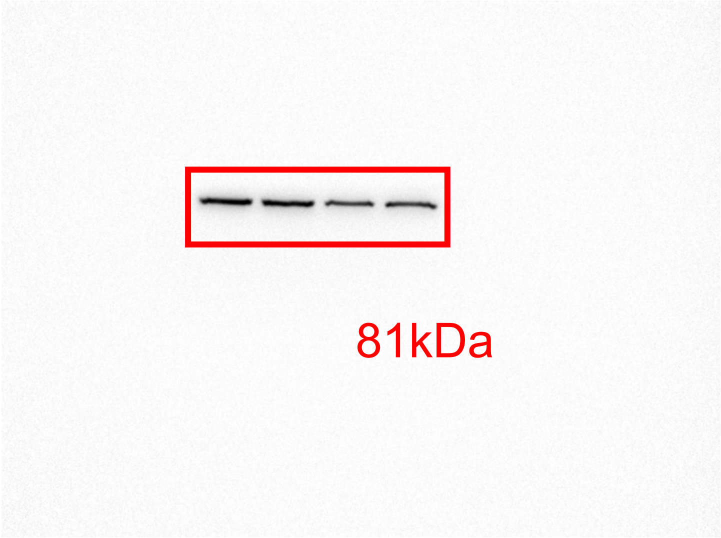

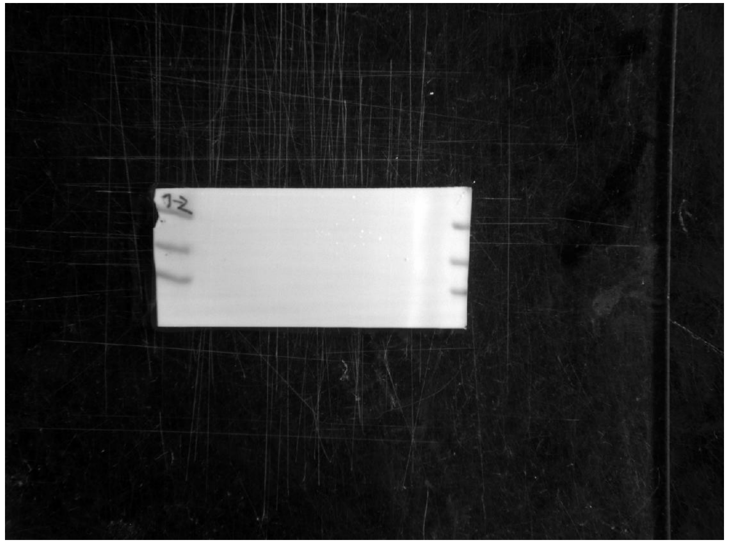

A549 β-actin

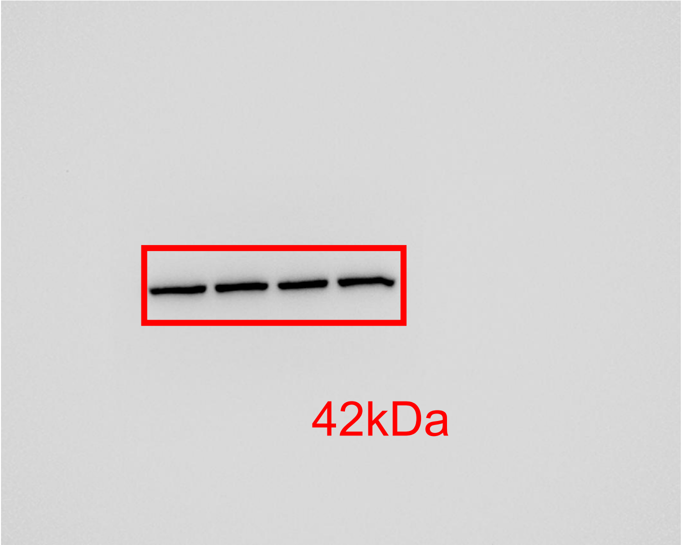

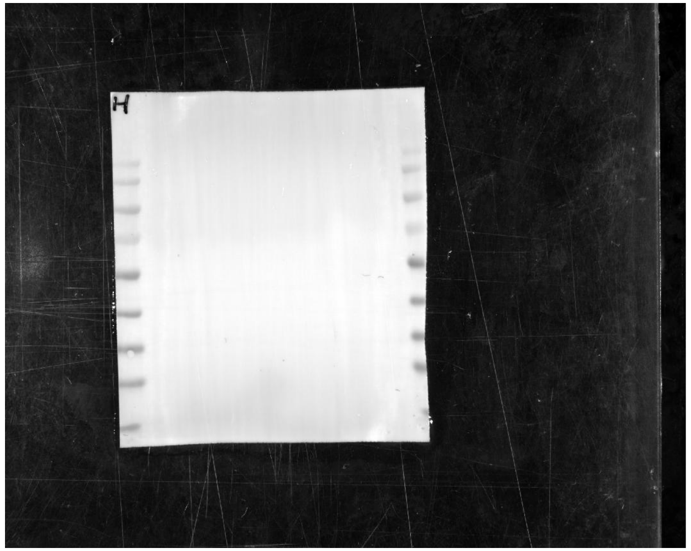

H1299 Ub

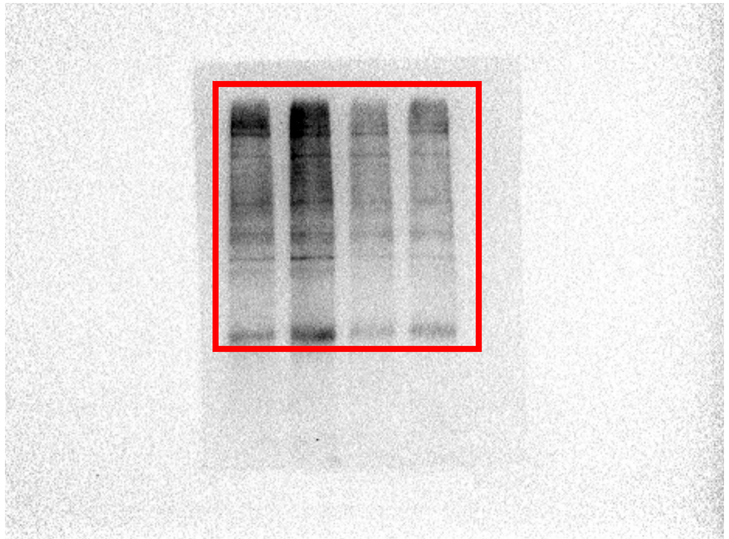

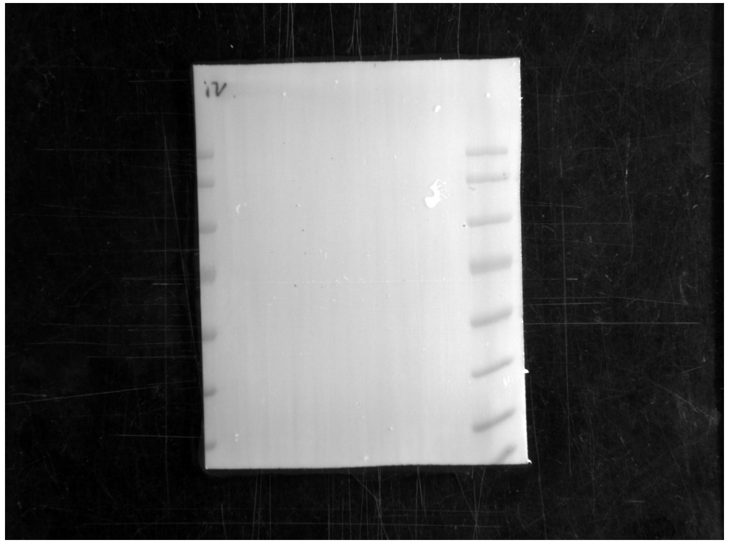

H1299
ZC-FLAG

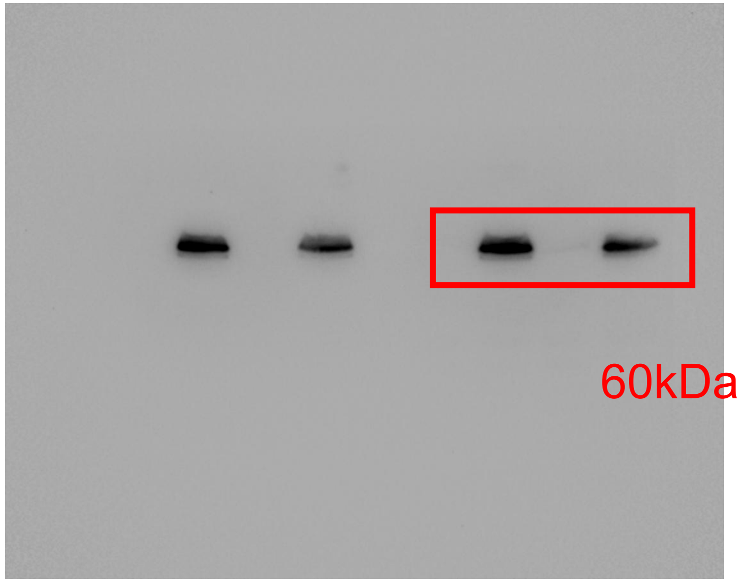

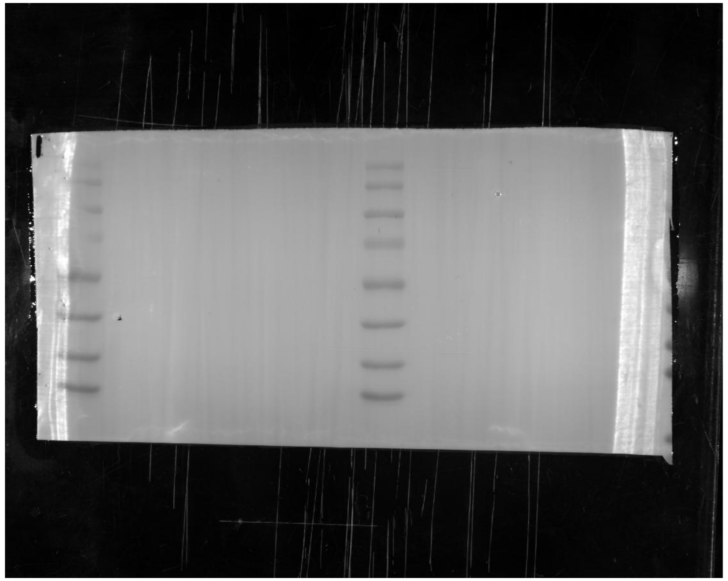

H1299 PTEN

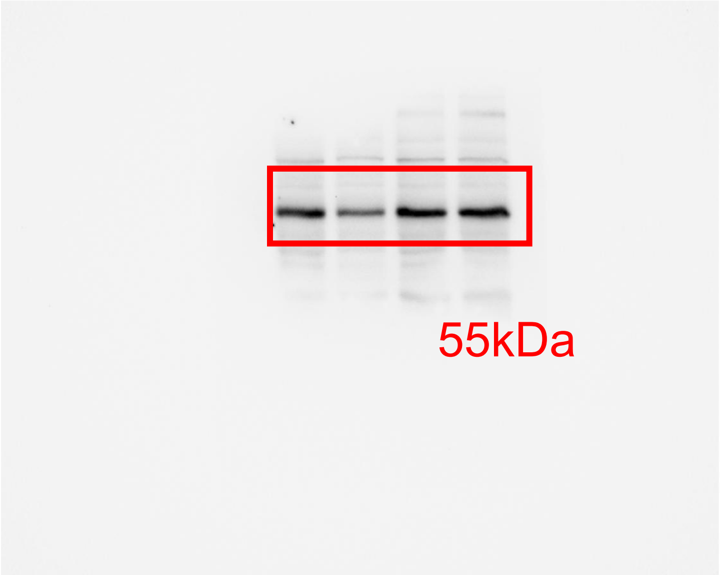

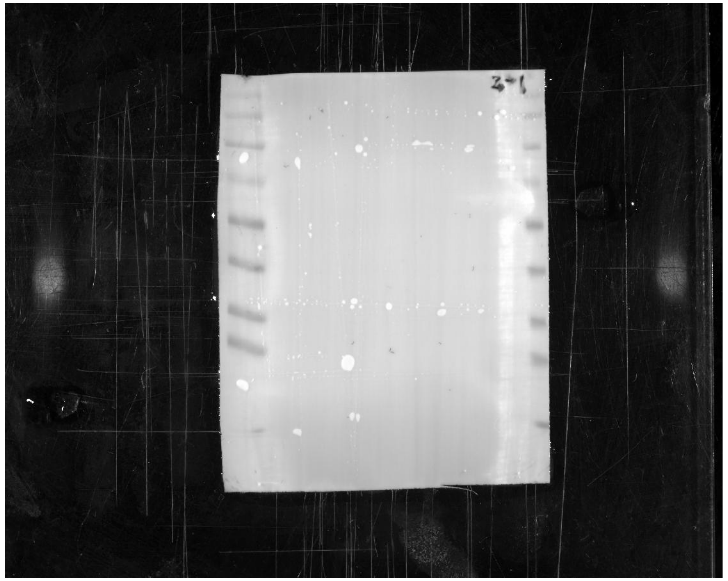

H1299 trim56

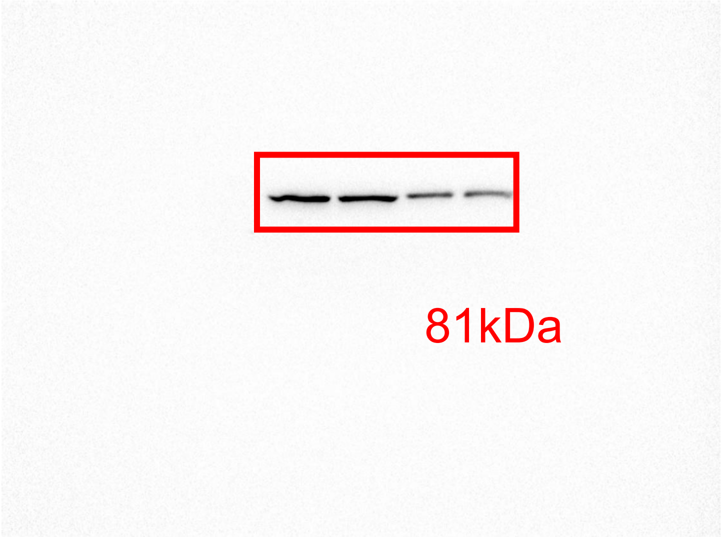

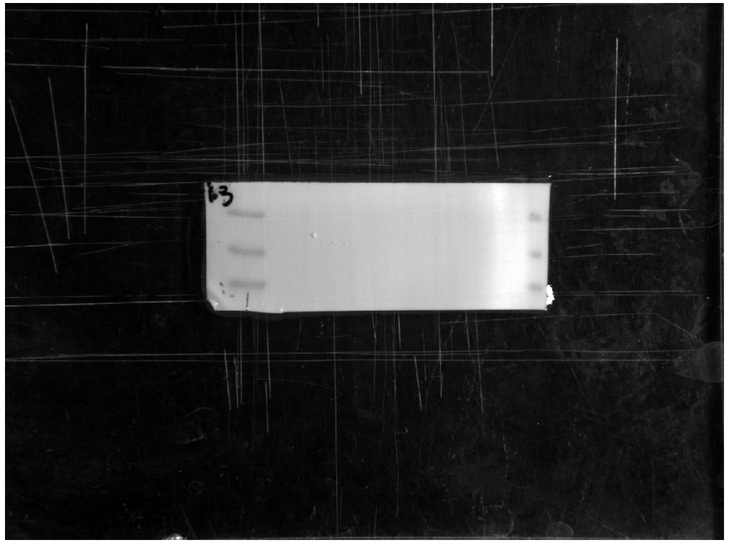

H1299 β-actin

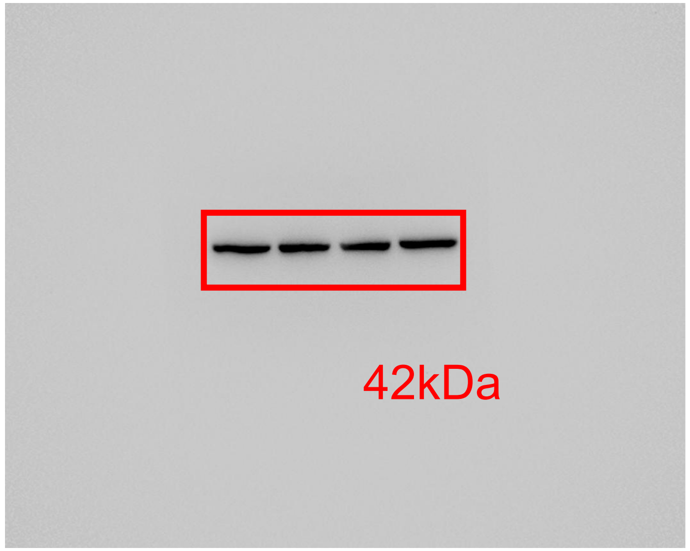

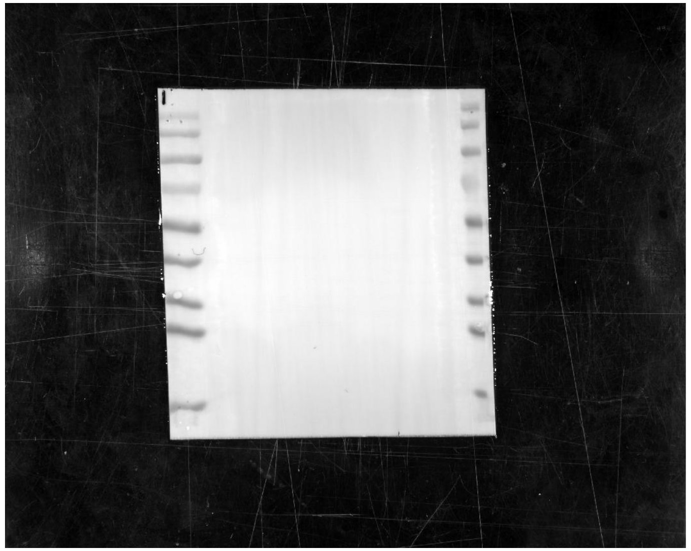

S-FIG 9

A549 ZC3H15

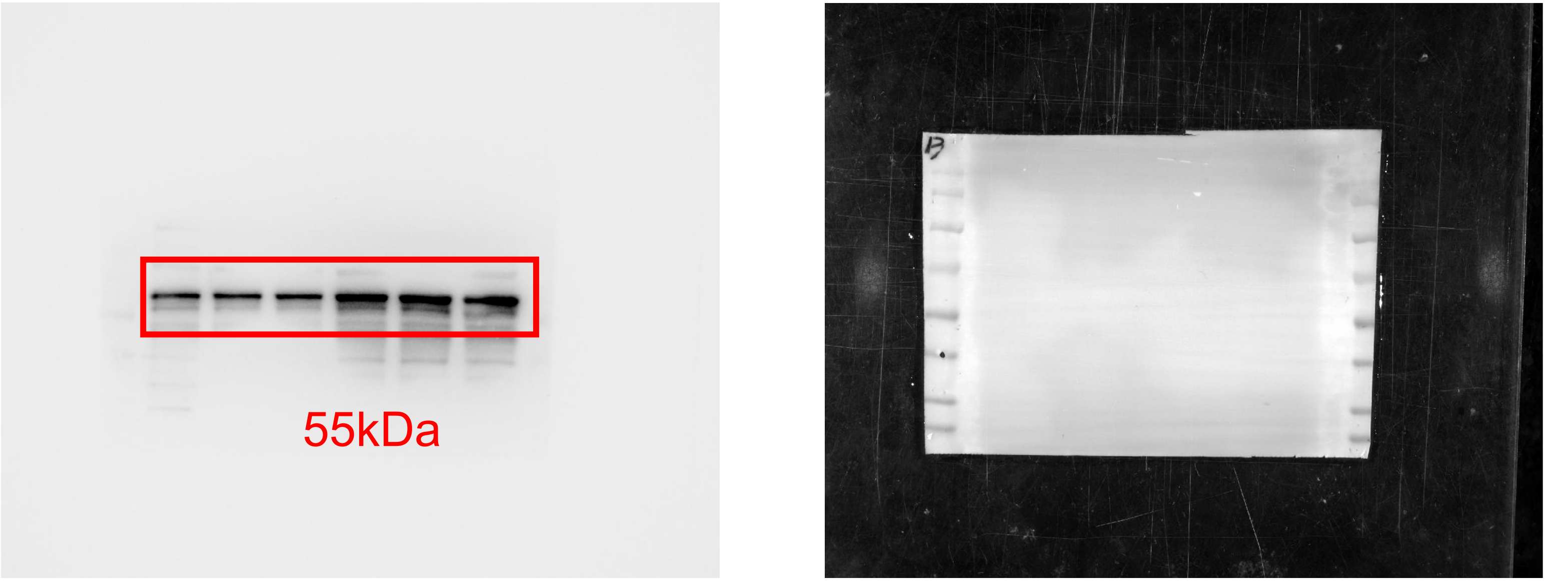

A549 ZC3H15

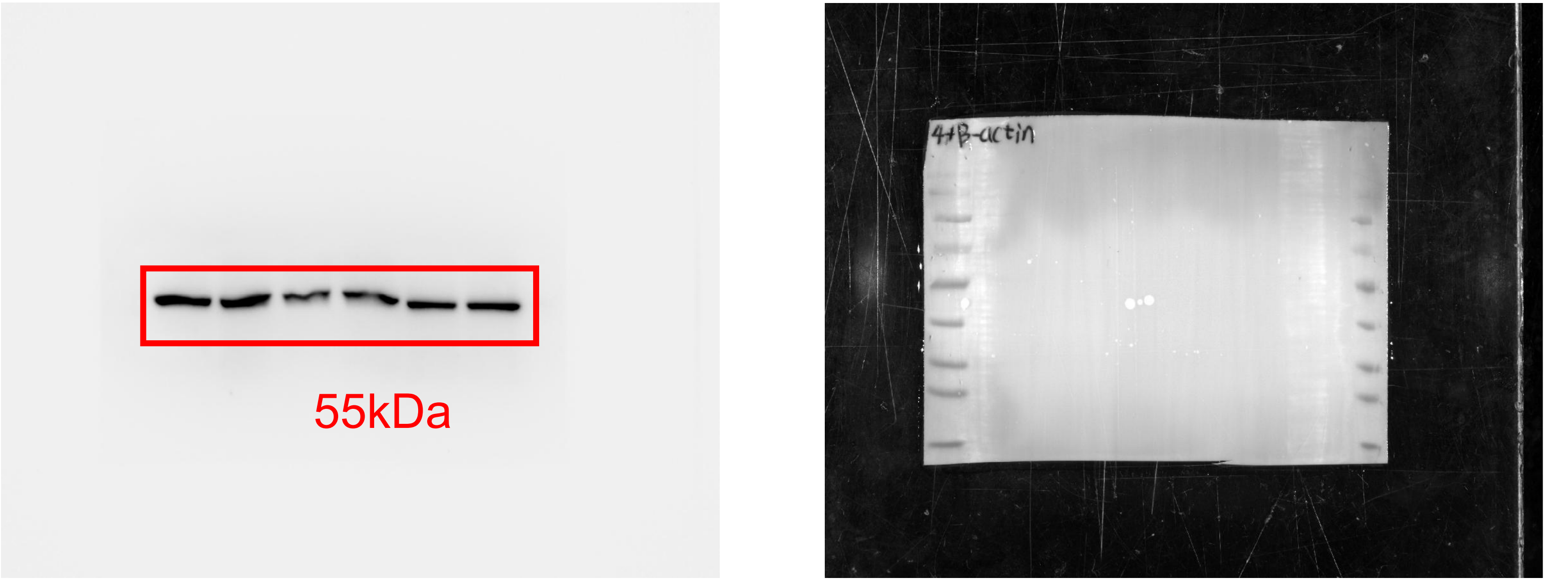

A549 ZC3H15

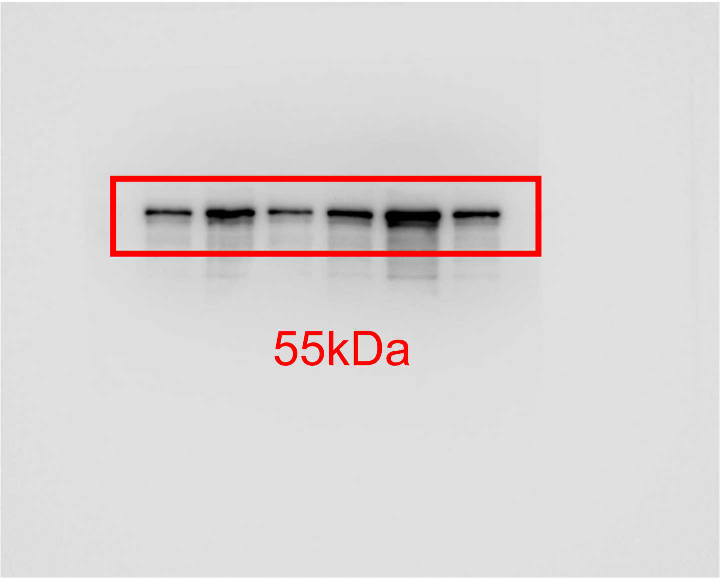

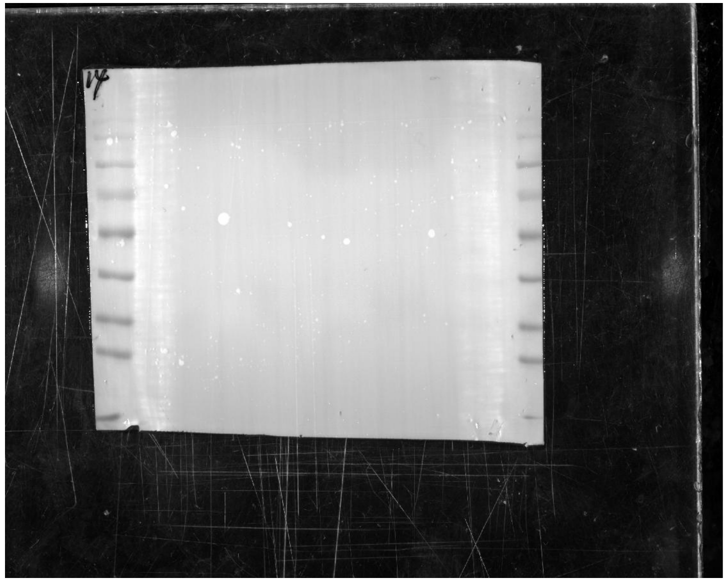

A549 ZC3H15

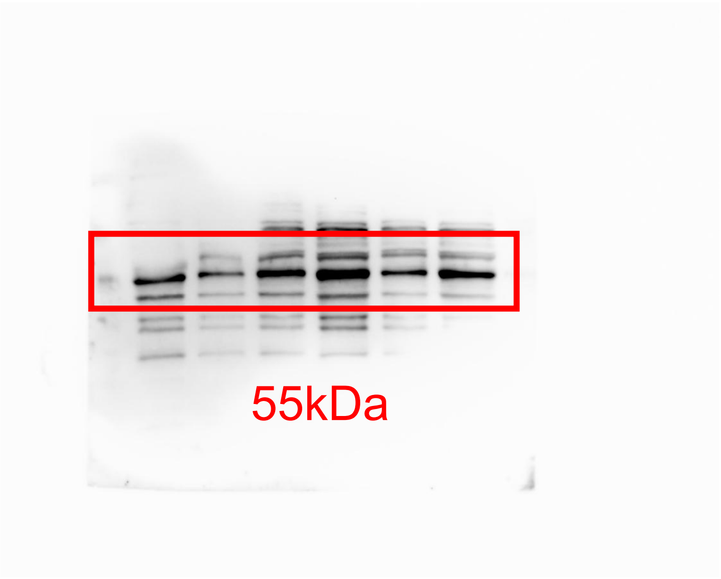

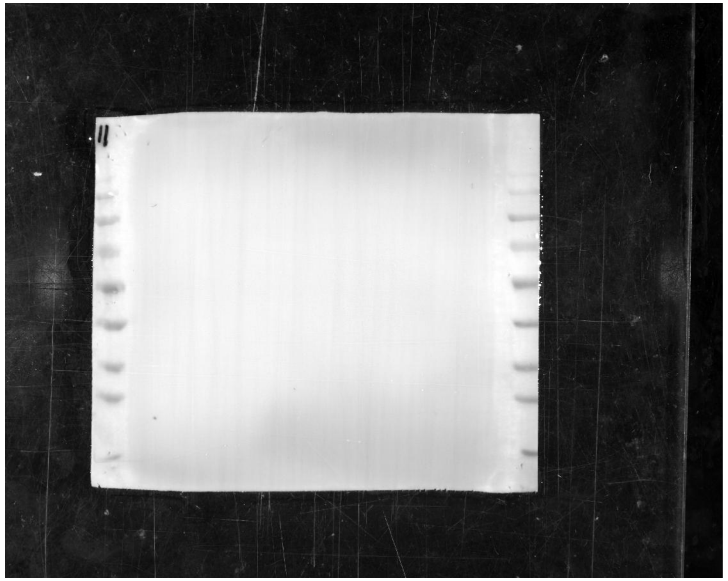

A549 TRIM56

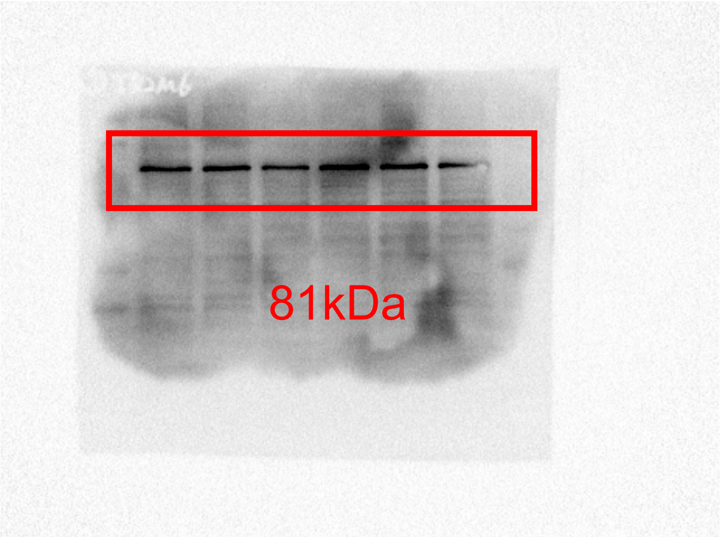

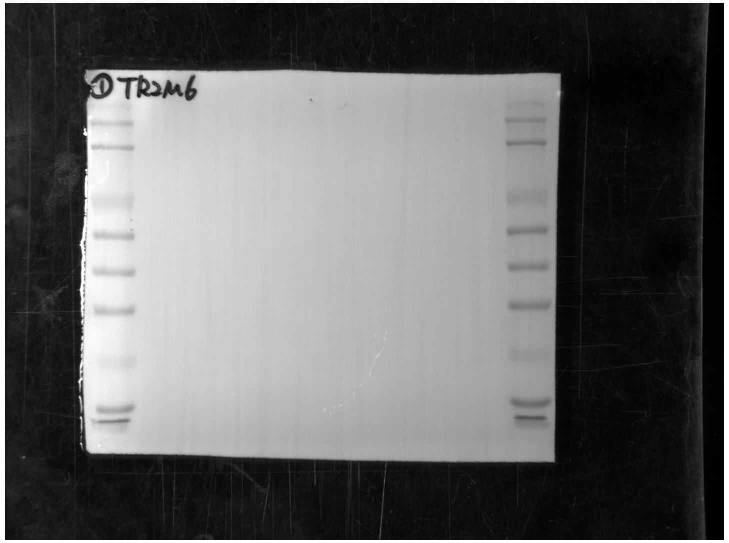

A549 P-AKT

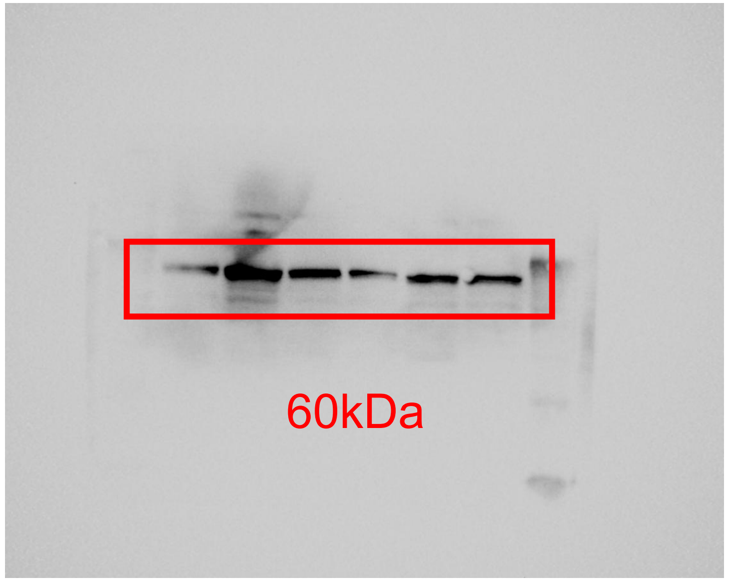

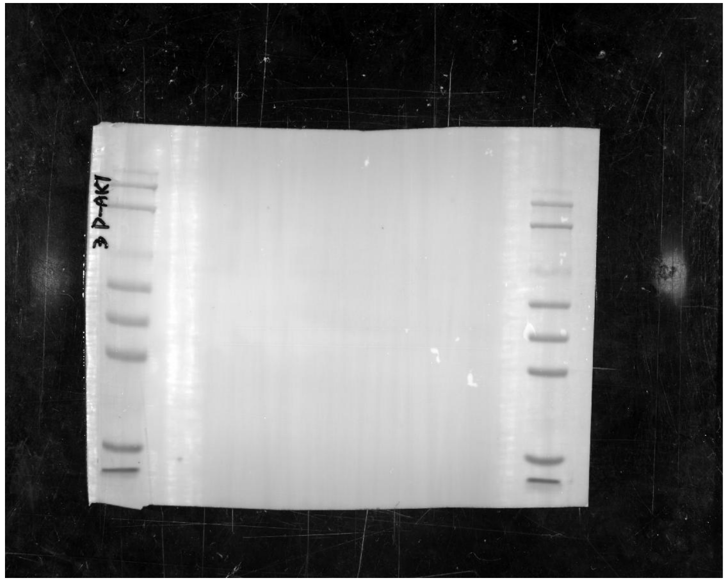

A549 AKT

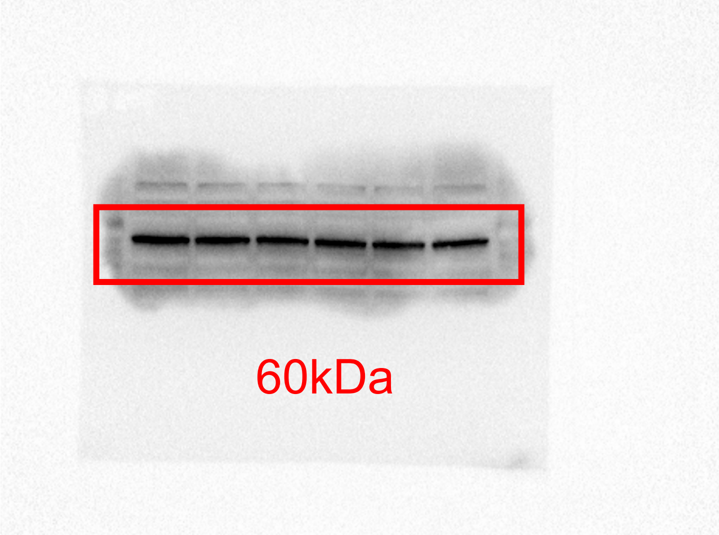

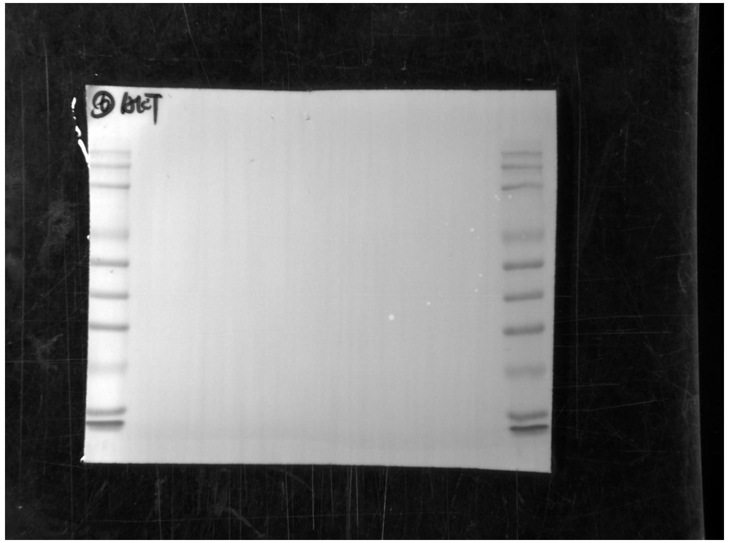

A549 P-MTOR

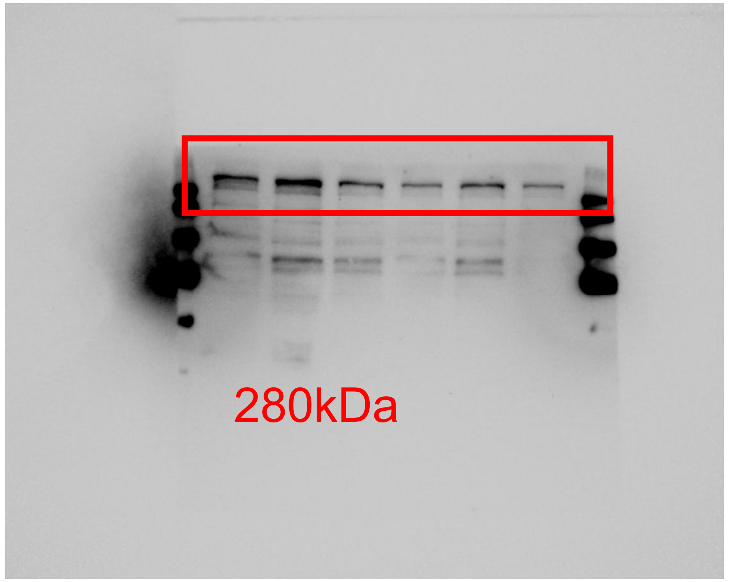

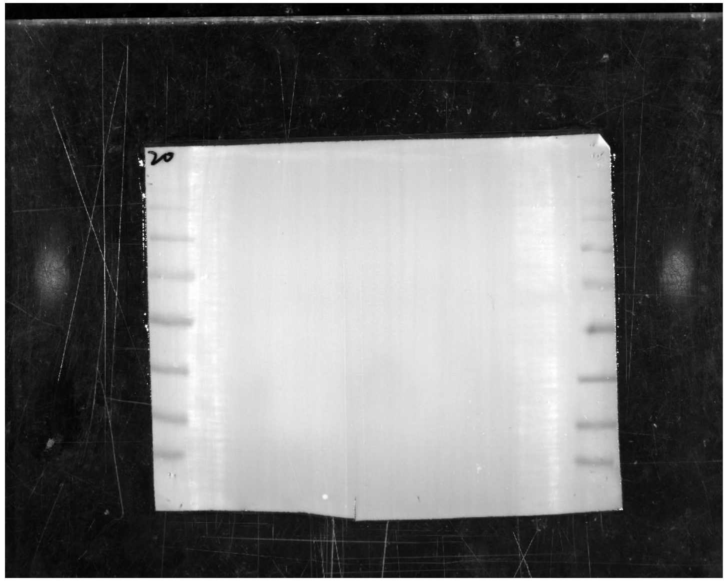

A549 MTOR

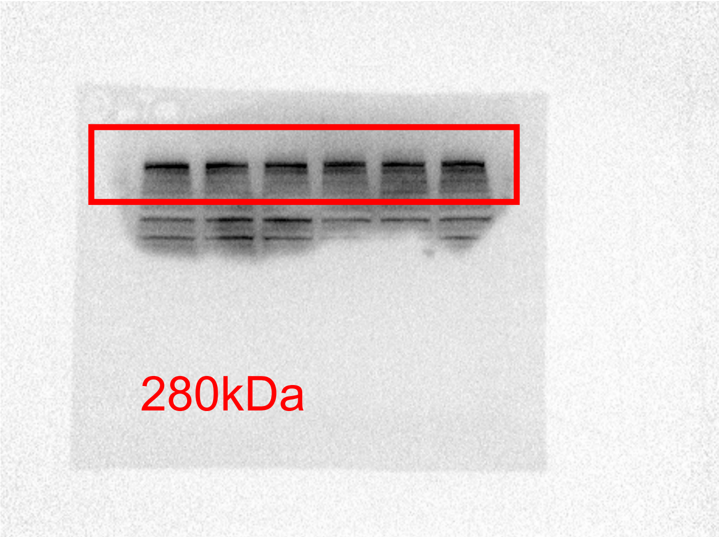

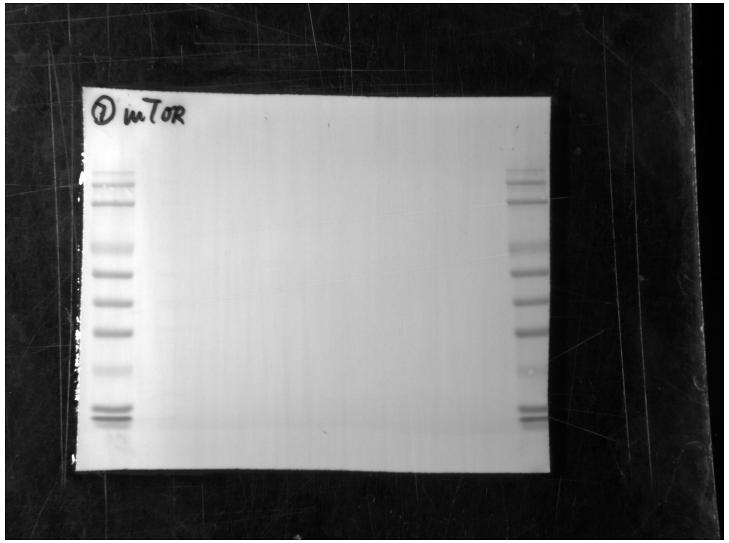

A549 ACTIN

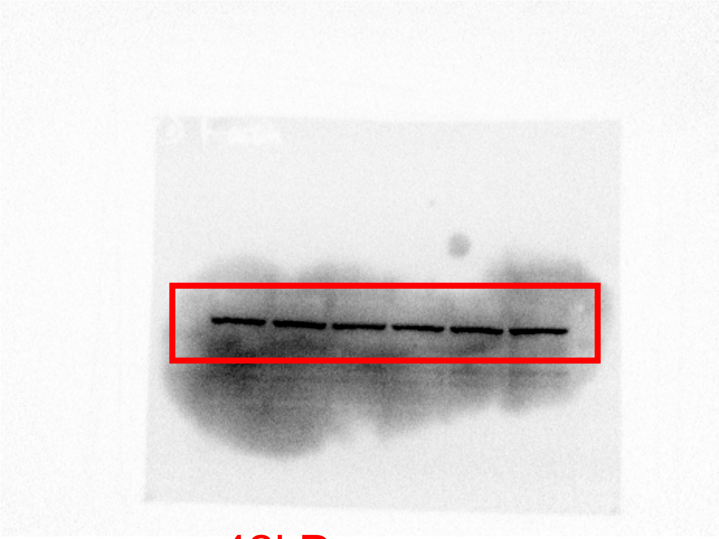

42kDa

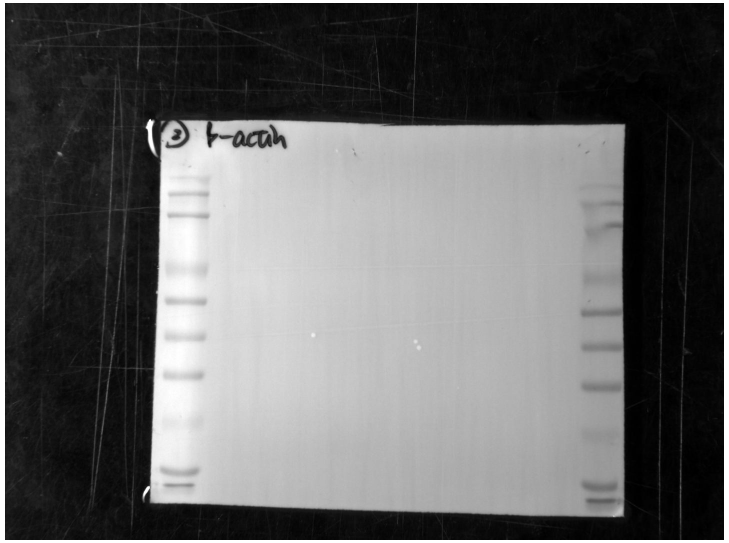

Supplement: Supplementary file 12 — full length blots [file 41419_2025_8138_MOESM12_ESM.pdf]
